# Child maltreatment and resilience in adulthood: a systematic review and meta-analysis

**DOI:** 10.1017/S0033291725001205

**Published:** 2025-06-02

**Authors:** Natalia E. Fares-Otero, Julia Carranza-Neira, Jacqueline S. Womersley, Aniko Stegemann, Inga Schalinski, Eduard Vieta, Georgina Spies, Soraya Seedat

**Affiliations:** 1 Department of Psychiatry and Psychology, Bipolar and Depressive Disorders Unit, Hospital Clínic, Institute of Neurosciences (UBNeuro), Barcelona, Catalonia, Spain; 2 Fundació Clínic per a la Recerca Biomèdica (FCRB), Institut d’Investigacions Biomèdiques August Pi i Sunyer (IDIBAPS), Barcelona, Catalonia, Spain; 3 Department of Medicine, Faculty of Medicine and Health Sciences, University of Barcelona (UB), Barcelona, Catalonia, Spain; 4 Faculty of Health Sciences, School of Medicine, Universidad Peruana de Ciencias Aplicadas (UPC), Lima, Peru; 5 South African PTSD Research Programme of Excellence, Department of Psychiatry, Faculty of Medicine and Health Sciences, Stellenbosch University, Cape Town, South Africa; 6 South African Medical Research Council Genomics of Brain Disorders Research Unit, Department of Psychiatry, Stellenbosch University, Cape Town, South Africa; 7 Department of Psychology, University of Bundeswehr München, Munich, Germany; 8 Network Centre for Biomedical Research in Mental Health (CIBERSAM), Health Institute Carlos III (ISCIII), Barcelona, Spain

**Keywords:** adaptive coping, adults, bullying, childhood trauma, emotion regulation, mental health, neglect, psychological well-being, resilient functioning, self-efficacy, self-esteem

## Abstract

We conducted a systematic review and meta-analysis to quantify associations between overall and subtypes of CM, global/trait resilience, and five resilience domains (coping, self-esteem, emotion regulation, self-efficacy, and well-being) in adults, and to examine moderators and mediators of these associations. A systematic search was undertaken on 12 June 2024 to identify published peer-reviewed articles in five databases (PROSPERO-CRD42023394120). Of 15,262 records, 203 studies were included, comprising 145,317 adults (M*_age_* = 29.62 years; 34.96% males); 183 studies and 557 effect sizes were pooled in random-effect meta-analyses. Overall CM and its subtypes were negatively associated with global/trait resilience and its domains (*r* = −0.081 to −0.330). Emotional abuse/neglect showed the largest magnitude of effect (*r* = −0.213 to −0.321). There was no meta-analytic evidence for an association between sexual abuse and coping, and physical abuse/neglect and self-esteem. Meta-regressions identified age, sample size, and study quality as moderators. Subgroup analyses found that associations between emotional abuse and emotion regulation were stronger, while associations between emotional abuse and self-esteem were weaker, in western *versus* non-western countries. No differences were found in associations between CM and resilience in clinical *versus* non-clinical samples. Narrative synthesis identified several mediators. Associations were of small magnitude and there were a limited number of studies, especially studies assessing CM subtypes, such as physical neglect, bullying, or domestic violence, and resilience domains, such as coping or self-efficacy, in males, and clinical samples. CM exposure negatively impacts resilience in adults, an effect observed across multiple maltreatment types and resilience domains. Interventions focused on resilience in adults with CM histories are needed to improve health and psychosocial outcomes.

## Highlights

- Being exposed to CM, especially emotional abuse and emotional neglect is associated with impaired resilience in adults.
- Age, sample size, study quality, and country/region moderate the association between CM and resilience.
- Self-compassion, self-concept, emotional intelligence, social support, parental/peer relationship quality, attachment style, PTSD, and mood symptoms mediate the association between CM and resilience outcomes.

## Introduction

Child maltreatment (CM), that is, sexual, physical, and emotional abuse, and physical and emotional neglect, including witnessing domestic violence and bullying exposure under 18 years of age (Cowley et al., 2025; Fares-Otero & Seedat, 2024), is one of the most potent and preventable risk factors for the development of physical and mental illnesses throughout the lifespan (Baldwin et al., 2023; Mehta et al., 2023) and is further associated with a multitude of negative psychosocial outcomes in both clinical (Fares-Otero, Alameda et al., 2023; Fares-Otero, De Prisco et al., 2023) and non-clinical populations (Pfaltz et al., 2022). However, outcomes of CM vary widely, and not all individuals exposed to CM experience the same level or range of negative health issues or psychosocial consequences. This suggests resiliency in some individuals exposed to CM.

Resilience is the capacity of an individual to adapt successfully to highly adverse events and, by harnessing resources, maintain healthy functioning (Southwick et al., 2014). Resilience can be defined as a personal characteristic (or trait) captured by personal and psychosocial resources, and it can also be perceived as a process comprising bouncing back and growth (Ayed, Toner, & Priebe, 2019). Resilience may also enhance perceptions about one’s personal qualities, such as self-confidence, adaptability, and the ability to endure stress (Choi et al., 2019). As a dynamic system (Liu & Duan, 2023), resilience refers to the ability to function competently and face future challenges or adversities successfully, and can thus be regarded as both the process of returning to pre-exposure health and well-being and an outcome of one’s reaction to a stressful event (Bhatnagar, 2021).

To date, previous systematic reviews have reported on factors that promote adaptive functioning and positive mental health (Fritz et al., 2018; Meng et al., 2018) but were not able to draw firm conclusions on resilience factors contributing to improved psychosocial outcomes in adults with CM (Latham, Newbury, & Fisher, 2023). One meta-analysis examined associations between violence exposure and protective factors for resilience in children, showing that self-regulation and social support demonstrated significant additive and/or buffering effects in longitudinal studies (Yule, Houston, & Grych, 2019). A multivariate meta-analysis found that trait resilience mediated the association between childhood trauma and depression (Watters, Aloe, & Wojciak, 2023). An umbrella synthesis of meta-analyses on CM antecedents and interventions found that resilient individuals were characterised by lower susceptibility to changes in the environment and that these associations between resilience and susceptibility were moderated by constitutional (e.g. easy temperament) and contextual protective factors (e.g. parent intervention) (van IJzendoorn, Bakermans-Kranenburg, Coughlan, & Reijman, 2020).

Although the association between CM and resilience has been widely recognised, available reviews (Fritz et al., 2018; Latham et al., 2023; Meng et al., 2018) and meta-analyses (van IJzendoorn et al., 2020; Watters et al., 2023; Yule et al., 2019) have focused on broader concepts of childhood adversity and protective factors that promote resilience. It remains unclear whether CM and its specific subtypes are differentially associated with resilience in adulthood using a multi-domain definition and approach for resilience (Fares-Otero, O et al., 2023). Furthermore, analyses of potential moderating (e.g. age, sex, mental condition) or mediating factors (e.g. personality, mood symptoms) in the association between CM and resilience have seldom been undertaken.

This systematic review and meta-analysis sought to address these gaps by determining whether overall CM and its subtypes are associated with global/trait resilience and distinct resilience domains (coping, self-esteem, emotion regulation, self-efficacy, and well-being) in adults. The review also explored potential moderators that may modify the strength and/or direction of the association between CM and resilience, and mediators that may explain the association. Understanding CM-resilience associations can guide clinical decision-making or policy development. Collectively, this information can inform clinical practice guidelines and strategies for improving prediction, early identification, and targeted interventions.

## Methods

### Protocol

The study protocol was registered on PROSPERO (CRD42023394120) and published elsewhere (Fares-Otero, O et al., 2023) before the completion of the study. This review follows the Preferred Reporting Items for Systematic Reviews and Meta-Analyses (PRISMA) (Moher et al., 2009; Page et al., 2021) (see ST1 and ST2 in the Supplement), the Meta-Analysis of Observational Studies in Epidemiology (MOOSE; Stroup et al., 2000) checklist (see ST3 in the Supplement), and the Enhancing the Quality and Transparency of Health Research (EQUATOR) (Altman et al., 2008) reporting guidelines. For a comprehensive glossary of terms used in this work, see SA1 in the Supplement.

### Search strategy and selection criteria

A systematic search using multiple medical subject headings (MeSH), terms, and keywords related to (1) ‘childhood maltreatment’ and ‘resilience’ (domains) using the Boolean operator ‘AND’ adapted according to database thesauruses (see the search strategies and terms appended in SA2 in the Supplement) was implemented on PubMed (Medline), PsycINFO, Embase, Scopus, and Web of Science (core collection) to identify relevant studies on 18 April 2023 and updated on 12 June 2024. No language or date limits were applied. To identify additional eligible studies, references of studies of relevance were cross-referenced manually. This backward and forward citation searching was carried out in PubMed and Google Scholar (NEF-O).

Four independent reviewers (NEF-O, JC-N, JSW, GS) screened the titles and abstracts according to the pre-specified eligibility criteria and discrepancies were resolved through consensus. Articles, that appeared eligible from the abstract, or were of unclear eligibility, were full-text screened (NEF-O, JC-N, JSW, GS). Any disagreements over study eligibility were discussed and an independent senior researcher (SS) was consulted if a consensus could not be reached among the reviewers. Rayyan QCRI software (https://rayyan.qcri.org/) was used to manage citations, remove duplicates, and screen titles and abstracts.

### Inclusion and exclusion criteria

Only original research articles published in peer-reviewed journals were included. Eligible studies reported quantitative associations between at least one CM subtype (exposure variable; i.e. sexual, physical, or emotional abuse; physical or emotional neglect, domestic violence, bullying) and at least one resilience domain (outcome variable; i.e. global/trait resilience, coping, self-esteem, emotion regulation, self-efficacy, well-being) in adults (see the definition and operationalisation of exposure and outcome variables in SA3 in the Supplement). When more than one published study used the same subjects and outcomes, the study with the larger sample size was chosen to maximise power.

Studies were excluded if they: (1) were reviews, meta-analyses, clinical case studies, abstracts, conference proceedings, study protocols, letters to the editor not reporting original data, editorials, commentaries, theoretical pieces, books, book chapters, preprints, theses, or grey literature; (2) only included children and/or adolescents; (3) were studies that exclusively assessed trauma experienced in adulthood (≥ 18 years); (4) were qualitative studies; (5) aimed to conduct or evaluate an intervention and/or to assess treatment outcomes and did not provide baseline data.

According to the **PECOS** (Population, Exposure, Comparator, Outcomes, Study design) framework (Morgan, Whaley, Thayer, & Schünemann, 2018), studies were included if they: (1) **(P)** were conducted on human adults (≥ 18 years) with or without current/past mental or any medical condition and who were exposed to CM; (2) **(E)** assessed the presence of CM (*<* 18 years) and measured overall (total) or specific CM subtypes with validated measures or through clinical interviews/reports; (3) **(C)** compared individuals with and without CM within the same sample; (4) **(O)** evaluated resilience with validated instruments; (5) quantitatively examined and reported associations between CM and resilience or data that allowed correlations to be calculated or provided these data on request; (6) **(S)** were cross-sectional, or longitudinal (providing baseline data).

### Study outcomes

The selection of resilience (outcome) domains was based on resilience outcomes examined in the included studies, and categorisations used in the trauma and resilience research fields (Rutten et al., 2013; Southwick et al., 2014). After study selection, we categorised the study outcomes into: (**I) *Global or trait resilience***: conceived as a relatively stable, personal innate characteristic that is marked by psychological hardiness, and ego resilience (Connor & Davidson, 2003); and (**II) Five separate domains of resilience**, including: (**1) *Coping:*** conscious, volitional efforts to regulate emotion, cognition, behaviour, physiology, and the environment in response to stress (Bonanno, Romero, & Klein, 2015; Bonanno, Westphal, & Mancini, 2011); (**2) *Self-esteem:*** one’s overall sense of self-worth or personal value that represents a comprehensive evaluation of oneself, including positive and negative evaluations (Brown, Dutton, & Cook, 2001); (**3) *Emotion regulation:*** the process by which individuals influence the occurrence, timing, nature, experience, and expression of their emotions (Kok, 2020); (**4) *Self-efficacy:*** sense of perceived self-efficacy to cope with daily hassles and stresses and adapt after experiencing all kinds of stressful life events, including a person’s belief in their ability to complete a task or achieve a goal (Bandura, 1982); (**5) *Well-being:*** biological and psychological qualities of well-being and mental health that enable successful adaptation or swift recovery from life adversity, such as optimism, a sense of coherence, the experience of positive emotions, having a purpose in life, and a sense of mastery (Ruggeri et al., 2020; Rutten et al., 2013).

Appendix SA4 in the Supplement provides a complete definition and operationalisation of each outcome domain and ST4 provides a complete overview of assessments of each outcome domain.

### Data extraction and study quality assessment

Data from eligible studies were extracted and tracked in Microsoft Excel by two groups of independent reviewers in the initial (NEF-O, JC-N, JSW, and GS) and updated search (NEF-O, JC-N, JSW, AS, and GS) using a structured coding form.

Descriptive variables extracted comprised demographics, and measurement instruments for CM, and resilience domains (see a detailed description of the extracted variables in SA3 in the Supplement). Correlation coefficients (*r*) were extracted as measures of effect size index. If not reported in the original publication, information was calculated from available statistics using established formulas (Lenhard & Lenhard, 2017; Lipsey & Wilson, 2001) or was requested from the authors.

The included studies were assessed for study quality by two groups of independent reviewers for the initial (JN-C, JSW, and GS) and updated search (JN-C, JSW, AS, IS, and GS) using a modified version of the Newcastle–Ottawa Scale (NOS) for non-randomised studies as used in previous meta-analyses in the field (Fares-Otero, Alameda et al., 2023; Fares-Otero, De Prisco et al., 2023). When using the NOS, studies are rated depending on sample selection, comparability of groups, and assessment of exposure or outcome, and the adapted version contains additional items to assess sample size, confounders, and statistical tests as recommended by the Cochrane Handbook (Higgins et al., 2011) (see ST5 in the Supplement).

Any disagreements over data extraction and/or study quality were discussed, and the lead researcher (NEF-O) was consulted if a consensus could not be reached, with discrepancies resolved through general consensus.

### Statistical analysis

Random-effect meta-analyses were conducted when a minimum of five studies (Jackson & Turner, 2017) were available. If the number of available effect sizes did not allow random effects meta-analysis, study findings were summarised and appraised qualitatively in a narrative synthesis (Popay et al., 2006). For those studies not reporting correlation coefficients, information was transformed from available statistics (e.g. mean and standard deviations between groups comparisons, regression coefficients) (Lenhard & Lenhard, 2017). Pearson correlation coefficients (effect sizes) were Fisher’s *Z* transformed to stabilise the variance and calculate reliable confidence intervals (CIs) and back transformed after pooling to allow for clearer interpretation, as per procedures used in previous meta-analyses (Fares-Otero, Alameda et al., 2023; Fares-Otero, De Prisco et al., 2023). Thus, all pooled effects were reported as correlation coefficients.

For the studies conducting separate analyses for emotion regulation subscales (i.e. acceptance, refocus on planning, positive reappraisal, expressive suppression, rumination, and experiential avoidance) (Güler, Demir, & Yurtseven, 2024; Mohammadpanah Ardakan, Khosravani, Kamali, & Dabiri, 2024; Musella et al., 2024; Peng et al., 2021; Sistad, Simons, Mojallal, & Simons, 2021), results were pooled using correction estimates (Olkin & Pratt, 1958) before inclusion in the meta-analysis.

The heterogeneity of effect estimates was investigated using Cochran’s *Q-*test and *I*^2^ statistics (Higgins, Thompson, Deeks, & Altman, 2003). The between-study variance of the underlying distribution of true effect sizes were reported using the tau square (*τ*^2^) statistic. Alongside the 95% CIs and the mean pooled effect provided, the prediction intervals estimating the extent to which effect sizes vary across studies (Borenstein, 2022b) were displayed as part of the forest plots (marked in red).

Additionally, the heterogeneity and content of studies were qualitatively described and possible reasons for the variability were considered by analysing the characteristics of the studies included. Meta-regressions for pre-defined continuous variables were conducted, including age (mean years), sex (% males), and the influence of sample size and study quality (NOS rating). Individual subgroup analyses were conducted for categorical variables, that is, western (EU and Scandinavian countries, the United Kingdom, Iceland, the United States, Canada, Australia, and New Zealand) *versus* non-western countries (Asia, Africa, Latin America, Eastern Europe, Middle East), clinical samples (the presence of any diagnosis of mental disorders, according to DSM (Bell, 1994; Kübler, 2013) or ICD (World Health Organisation, 2019) criteria, *versus* non-clinical samples (subjects recruited from the community and who were not diagnosed with a disorder). Subgroup analyses used a mixed-effects model (a random-effects model within subgroups and a fixed-effect model across subgroups). Other evidence of confounders and effect moderators and mediators on associations between CM and resilience outcomes was narratively synthesised (Popay et al., 2006).

One-study-removed sensitivity analyses were conducted to determine whether a particular study or a set of studies were contributing to potential heterogeneity and to determine the robustness of the meta-analyses (Higgins & Thompson, 2004).

For any meta-analysis with ≥10 studies, funnel plot asymmetry (Egger, Smith, Schneider, & Minder, 1997) was visually evaluated and possible explanations for the asymmetry were considered (small-study effects, publication bias). Publication bias was also assessed and quantified by Egger’s linear regression asymmetry test (Sterne, Gavaghan, & Egger, 2000). Given that these tests might be underpowered if only a small number of studies are available, the non-parametric trim-and-fill method (Duval & Tweedie, 2000) was used to examine the extent to which publication bias may contribute to the meta-analysis results if the search yielded few studies. Risk of bias analyses used a random-effects model, while a fixed-effect model was used to determine missing studies.

Statistical significance was evaluated two-sided at the 5% threshold (two-tailed). Interpretation of correlation coefficients was based on pre-defined cut-offs as follows: *r* values between 0 and 0.3 indicate small, values between 0.3 and 0.7 indicate moderate, and values above 0.7 indicate strong associations (Ratner, 2009).

All quantitative analyses were performed using Comprehensive Meta-Analysis v4.0 (CMA, version 4-meta-analysis.com) (Borenstein, 2022a) and R version 4.1.2 (RStudio Team, 2020). The figure illustrating the results of the meta-analytic synthesis was created using the *ggplot2* package.

## Results

### Study selection

**Figure 1.**
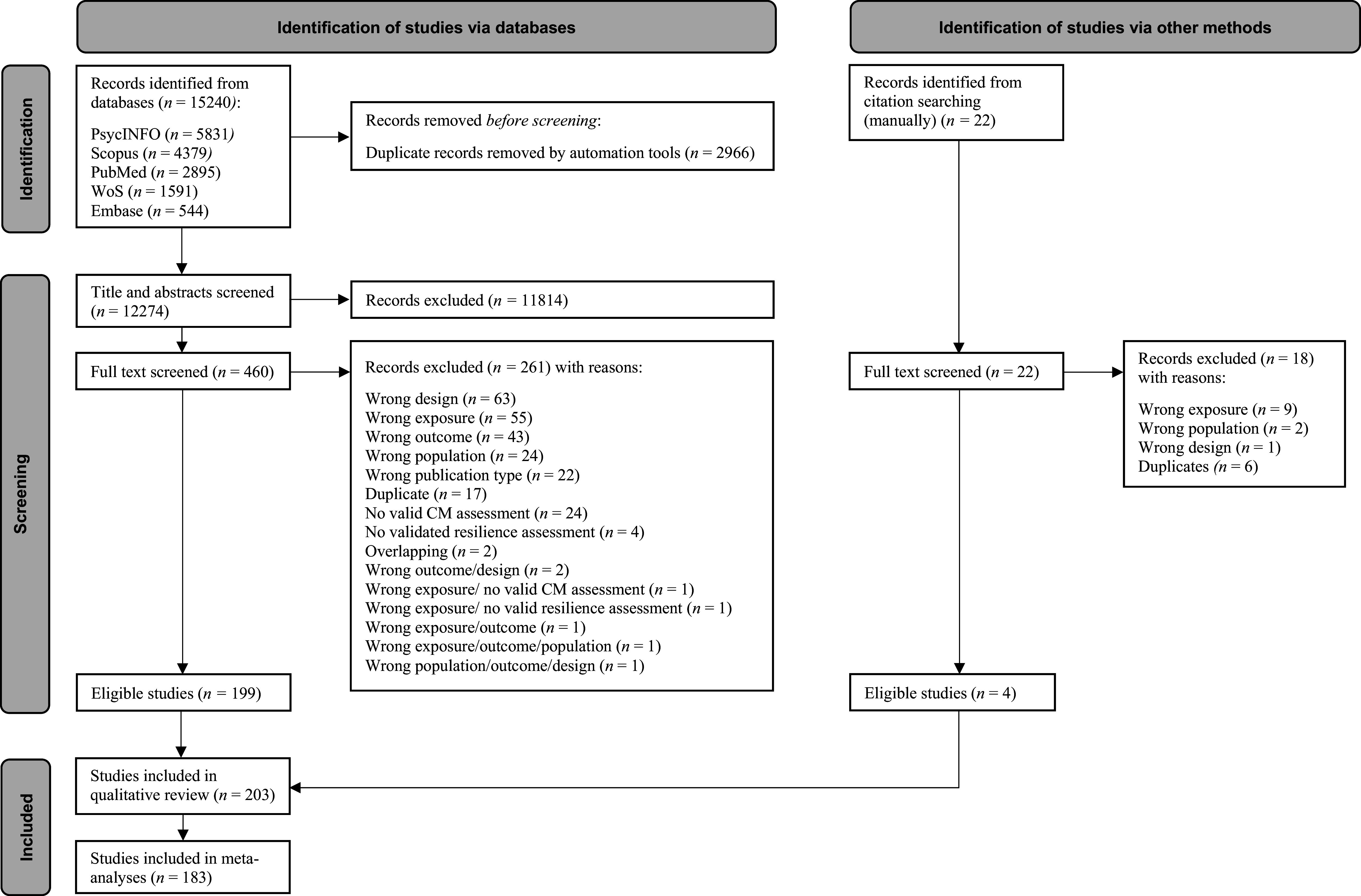
PRISMA 2020 flowchart outlining the study selection process.

From 15,262 identified records (15,240 through databases and 22 studies through manual searches), 482 were full-text screened, and 203 studies were included in the qualitative synthesis, of which 183 were included in the quantitative synthesis, contributing to 557 effect sizes pooled in meta-analyses (see the process of study selection in detail in Figure 1, the full list of included studies in SA5, and the full list of excluded studies with reasons in SA6 in the Supplement).

### Characteristics of the included studies

The 203 included studies were published between 1994 and 2024 and were conducted in North America (*n* = 64), Asia (*n* = 45), Europe (*n* = 39), Turkey (*n* = 22), Middle East (*n* = 14), Oceania (*n* = 5), Latin America (*n* = 4), and Africa (*n* = 1), with a total of 101 (49.75%) studies conducted in western-countries, 93 (45.81%) studies conducted in non-western countries, and nine studies conducted in multiple countries/regions.

Most of the included studies were cross-sectional, except for 15 (7.39%) studies (Armitage et al., 2021; Billen et al., 2023; Chen, Shen, & Dai, 2021; Daniels et al., 2012; Dereboy, Sahin Demirkapi, Sakiroglu, & Safak Ozturk, 2018; ElBarazi, 2023; Guo et al., 2023; Herrenkohl et al., 2012; Jones, Marsland, & Gianaros, 2023; Kong, Homan, & Goldberg, 2024; Kumar et al., 2022; S. Liu et al., 2023; Martin et al., 2023; Salles et al., 2024; Sexton et al., 2015) with a longitudinal design.

The total sample of the included studies comprised 145,317 (range = 30–25,113) adults, of which 34.96% were males. The mean age was 29.62 (range = 18.25–72.24) years. Of the 2023 included studies, 78 (38.42%) studies were carried out in clinical samples, of which 55 (27.09%) reported the presence of any diagnosis of mental disorders according to DSM (Bell, 1994; Kübler, 2013) or ICD (World Health Organisation, 2019) criteria. Three (1.48%) studies were conducted in samples with physical conditions (Artime & Peterson, 2012; Crosta et al., 2018; Kızılkurt, Demirkan, Gıynaş, & Güleç, 2021).

Overall CM was examined in 122 (60.09%) of the included studies, while 91 (44.83%) studies examined emotional abuse, 89 (43.84%) studies examined physical abuse, 97 (47.78%) studies examined sexual abuse, 66 studies (32.51%) examined emotional neglect, while 53 (26.11%) studies examined physical neglect. Bullying (or peer victimisation) was examined in 13 (6.40%) studies, and domestic violence exposure was examined in 11 (5.42%) studies.

Most of the included studies included retrospective assessments of CM. The Childhood Trauma Questionnaire (CTQ) short-form (28 items) was used in 141 (69.46%) studies, including shortened (25 items) or translated versions; while structured clinical interviews were used in seven (3.45%) studies and official case record reviews were used in three (1.48%) studies.

Forty-eight (23.65%) studies controlled for confounders in their analysis with a wide range of confounders being considered, including sex/gender, age, race/ethnicity, household characteristics, health measures, additional traumas, substance abuse, and mood symptoms. See further descriptive characteristics of the included studies in Table 1.

**Table 1.** Sociodemographic and clinical characteristics of the included studies

Authors/publication year	Country/region	Total N	Mean age (SD) in years	% Male	Descriptives n (%)	Instrument to assess CM	Type of CM	Instrument to assess resilience domains	Study outcome: resilience domains	Confounders
Allbaugh et al. (2017)	USA/North America	179	36.65 (10.55)	0	179 with suicide attempts and IPV history	CTQ–28	Overall CM, EA, PA, SA	SRI–25	Global/trait RES (suicide)	NA
Anctil et al. (2007)	USA/North America	564	29.02 (5.80)	49.10	70.4% DSM-IV disorder: 36.1% learning disability, 18.4% ADHD	Foster care case record reviews	EA, PA, SA, neglect	RSES–9	Self-esteem	NA
Armitage et al. (2021)^a^	UK/Europe	1486	23 (NA)	36.50	ALSPAC offsprings, 6.5% had a diagnosis of depression	BFIS–9, ALSPAC Mother’s reports of child’s victimisation	Bullying	BPNSS, MLQ, SHS, SWLS, WEMWBS–14	WB	Depressive symptoms, emotional problems, conduct problems, maternal education, maternal depression, social class, employment, income, sex
Arslan (2015)	Turkey/Europe-Asia	320	24.62 (3.93)	34.10	320 college students	CTQ – Turkish version EA subscale	EA	ARM – Turkish version, BSI	Global/trait RES, self-concept	NA
Arslan and Genç (2022)	Turkey/Europe-Asia	421	20.72 (1.06)	35.00	421 adults college students	PMQ–12	EA	14-MHC-SF – Turkish version	WB	NA
Artime and Peterson (2012)	USA/North America	320	NA (NA)	100	198 (62%) with current/past STI diagnosis, 1 with HIV	CTQ–28	Overall CM, EA, PA, SA, EN, PN	DERS	ER	NA
Babad et al. (2022)	USA/North America	436	19.73 (1.83)	36.20	436 college students	ACE-Q–9	EA, PA, SA, EN, PN, DV	CAP Ego-Strength subscale	Self-Esteem	NA
Berhe et al. (2023)	Germany/Europe	351	24.8 (6.54)	46.15	351 adults from the community	CTS	Overall CM	28-Brief-COPE, GSES, WHO–5	Coping, Self-efficacy, WB	Age, sex, SES, years of education
Berzenski (2019)	USA/North America	500	19.51 (2.29)	30.60	500 college students	CTQ-SF	EA, EN	DERS–36	ER	Race, sex
Berzenski and Yates (2010)	USA/North America	2169	19.15 (1.52)	36.20	2169 college students	CATS, CMIS	EA, PA, SA, DV	DERS–36	ER	NA
Billen et al. (2023)	Belgium and The Netherlands/Europe	94	42.67 (10.46)	100	Forensic psychiatric patients: 31.9% PD; 26.6% SUD; 13.8% paraphilic disorder; 8.5% psychotic disorder; 8.5% developmental disorders; 17.1% other	CTQ–28	Overall CM	BSCS–13, DERS–16, UPPS-P–20	ER	NA
Blood and Blood (2016)	USA/North America	72	21.9 (3.40)	86.11	36 with stuttering; 36 without stuttering	RBQ–46 adapted to include cyberbullying	Bullying	RSES–10, SWLS–5	Self-esteem, WB	NA
Bouchard and Sonier (2023)^a^	Canada/North America	200	20.22 (2.29)	31.00	200 young adults and their mothers	SRQ–10	(Sibling) Bullying	25-SPSI-R	Social problem solving	NA
Bradley, Schwartz, and Kaslow (2005)	USA/North America	134	34.6 (9.37)	0	134 (100%) with IPV history, suicidal behaviour, PTSD symptoms	CTQ–28	Overall CM	14-Brief RCOPE, TSEI–16	Coping, Self-esteem	NA
Brodski and Hutz (2012)	Brazil/South America	293	20.7 (2.70)	34.60	293 college students	CTQ–28 – Brazilian version	EA	SWLS, PANAS, RSES–10 – Brazilian version	Self-esteem, WB	NA
Broekhof et al. (2015)	The Netherlands/Europe	2104	46 (13.10)	34.20	79.4% affective disorders: 643 (30.6%) current affective disorders, 1027 (48.8%) lifetime affective disorders, 434 (20.6%) healthy controls	CTI	Emotional maltreatment (EA and EN), PA, SA	LOT-R–10	WB	Gender, age, years of education, physical activity, severity of depressive symptoms, depressive/anxiety disorders
Bungert et al. (2015)	Germany/Europe	167	BPD (acute): 28.3 (6.3); BPD (remitted): 29.2 (4.7); HCs: 26.8 (6.6)	0	77 with acute BPD, 15 with remitted BPD, 75 HCs	CTQ–28	Overall CM	RSES	Self-esteem	NA
Burns et al. (2010)	USA/North America	912	19 (1.63)	0	912 college students	CTQ–28	EA, PA, SA	DERS–36	ER	NA
Costa et al. (2024)	Portugal/Europe	302	35.82 (10.13)	0	302 from primary health care: 58.1% with risk for depression, 8.3% with probable PTSD diagnosis	CTQ–11-Portuguese abbreviated version	Abuse	ERQ–10 – Portuguese version	ER	Employment status, yearly income
Cantón-Cortés et al. (2012)	Spain/Europe	182	21.11 (4.61)	12.64	182 college students	CSAQ	SA	RSES–10	Self-esteem	NA
Cao, H. et al. (2022)	China/Asia	740	NA (NA)	31.50	740 college students	CTQ–28 – Chinese version	Overall CM	CD-RISC–25, SCSQ–20 – Chinese version	Global/trait RES, Coping	Gender
Cao, Q. et al. (2023)	China/Asia	971	24.5 (6.40)	NA	971 transgender, of them 505 with non-suicidal self-injury	CAQ – Mandarin version	Overall CM, EA, PA, SA, EN, PN	DERS – Mandarin version	ER	NA
Carvalho Fernando et al. (2014)	Germany/Europe	160	31.09 (9.48)	30.63	49 with BPD, 48 with MDD, 63 HCs	CTQ-German version	EA, PA, SA, EN, PN	DERS, ERQ – German version	ER	Age, gender
Cecen and Gümüş (2024)	Turkey/Europe-Asia	528	26.32 (9.73)	35.04	528 young adults from the community	CTQ-SF–28	EA	SCS-SF–12, SCRS–10, 3S–31	WB	Age, gender
Çelik and Odaci (2020)	Turkey/Europe-Asia	636	20.47 (1.88)	25.00	636 college students	CTQ–40 – Turkish version	Overall CM	SLCS–16 – Turkish version	Self-esteem	NA
Chang et al. (2023)	Taiwan/Asia	108	22.92 (2.43)	52.77	108 young adults from the community	ACE-IQ–24	Bullying	RSA–29 – Chinese version	Global/trait RES	Gender
Chaturvedi and Arya (2023)	India/Asia	104	21.4 (1.97)	35.58	104 healthy young adults	CTQ-SF–28	Overall CM	RSES–10	Self-esteem	NA
Chen et al. (2023)	China/Asia	433	18.92 (1.41)	89.80	149 (34.41%) college students with depression	CTQ–28	Overall CM, EA, PA, SA, EN, PN	CD-RISC–25 – Chinese version	Global/trait RES	NA
Cheng and Langevin (2023)	Canada/North America	428	21.15 (2.08)	10.50	573 emerging adults from the community	ETISR-SF, ICAST-R neglect subscale, CTs–2	Overall CM, EA, PA, SA, neglect (physical neglect), DV	DERS-SF–18	ER	Gender, ethnicity
Chi et al. (2021)^a^	China/Asia	2038	20.56 (1.9)	37.05	2038 college students	ACE-Q	DV	PTGI–21, SCS–26-C, CD-RISC SF–10 – Chinese version	Global/trait RES, Post-traumatic growth, self-compassion	Age, gender, subjective SES, family structure
Choe et al. (2021)	USA/North America	290	23.54 (5.36)	47.90	290 college students	RBQ–44	Bullying	RSES–10	Self-esteem	Gender, race
Choi et al. (2014)	South Korea/Asia	162	40.2 (15.44)	44.40	75 with MDD/DD-NOS; 37 with anxiety disorder; 9 with somatoform disorder; 14 with PTSD/ASD; 6 with adjustment disorder; 5 with SUD; 4 with mixed anxiety and depressive disorder; 12 with other diagnosis	CTQ-Korean version	EA, PA, SA	DERS–36	ER	Adulthood trauma
Christ et al. (2019)	The Netherlands/Europe	276	21.70 (2.38)	0	276 college students: 30.1% mild depressive symptoms; 8 moderate depressive symptoms; 2.1% severe depressive symptoms	CTQ-SF	EA, PA, SA	DERS–36	ER	NA
Clark et al. (2021)	USA/North America	213	36.79 (11.23)	0	213 (100%) clinical sample history of IPV and suicide attempt(s): 72.3% MDD; 44.5% BD; 8.5% PTSD	CTQ-SF–28	Overall CM, EA, PA, SA, EN, PN	BSE–18	Self-esteem	IPV, suicide attempt(s)
Cloitre et al. (2008)	USA/North America	109	35.61 (10.79)	0	78% with PTSD; 33% MDD; 28% GAD; 23% Dysthymia; 22% social phobia	CMIS	Overall CM	NMR	ER	NA
Crosta et al. (2018)	Italy/Europe	153	46.14 (14.61)	47.71	77 psoriatic patients, 76 HCs	CTQ-SF–28	Overall CM, EA, PA, SA, EN, PN	CD-RISC–25	Global/trait RES	NA
Daniels et al. (2012)	Canada/North America	70	36.4 (12.60)	41.40	19 of 55 participants (34.5%) with ASD; 12 of 64 participants (18.7%) with PTSD at 5 to 6 weeks; 5 of 44 participants (11.4%) with PTSD at 3 months	CTQ-SF–25	Overall CM	CD-RISC–25	Global/trait RES	NA
Daruy-Filho et al. (2013)	Brazil/South America	30	43.77 (12.36)	0	30 (100%) with BD Type 1	CTQ–28-Brazilian-Portuguese version	Overall CM, EA, PA, SA, EN, PN	WCQ–45, Brief-COPE–28-Brazilian-Portuguese version	Coping	NA
Davies et al. (2004)	USA/North America	142	23.57 (8.25)	0	142 college students	CMIS-SF	PA, SA, DV	RSES–10	Self-esteem	Non-physical forms of family conflict
Dawson et al. (2022)	Australia/Oceania	461	41.42 (16.68)	23.20	461 adults from the community	CTQ-SF–28	EA, PA, SA, EN, PN	ERQ–10	ER	NA
Demir et al. (2020)	Germany and Jordan/Europe-Asia	89	34 (10.18)	46.60	89 Syrian refugees: 21.3% mild depression, 29.2% moderate depression, 30.3% moderately severe depression, 18% severe depression; 27% mild anxiety, 36% moderate anxiety, 34.8% severe anxiety; 30.3% with PTSD	CTQ–28	Overall CM	CERQ–36	ER	NA
Dereboy et al. (2018)	Turkey/Europe-Asia	69	20.93 (NA)	33.30	33.3% with SCID I, 28.9% with SCID II psychiatric diagnoses	CTQ-SF–28	EA, SA	DERS–36	ER	NA
Di Nicola et al. (2024)	Italy/Europe	226	44 (11.7)	67.70	163 (72.1%) with SUD, 63 (27.9%) with SUD and suicide attempts, 46.6% with psychiatric comorbidities	CTQ-SF–28-Italian version	EA, PA, SA, EN, PN	DERS–36-Italian version	ER	Age, gender
Ekinci and Kandemir (2015)	Turkey/Europe-Asia	95	SUD 26.64 (5.47), HCs 25.56 (6.92)	90.53	50 adults with SUD: 13 (26%) MDD, 6 (12%) PTSD, 5 (10%) GAD, 6 (12%) dysthymic disorder, 1 (2%) OCD, 1 (2%) social phobia; 45 HCs	CTQ-Turkish version	Overall CM, EA/EN, PA, SA	RSES–10-Turkish version	Self-esteem	Gender
ElBarazi (2023)	Egypt/Africa	319	19.03 (0.46)	23.50	319 college students, 206 (64.58%) with CM, 113 (35.42%) without CM, 24 (7.5%) with any medical illness	CTQ	Overall CM, EA, PA, SA, EN, PN	DERS–36	ER	NA
Endo et al. (2024)	Japan/Asia	404	42.3 (11.9)	54.46	18 (4.46%) with a history of psychiatric treatment	CATS–38-Japanese version	Overall CM	RSES–10-Japanese version	Self-esteem	NA
Erol and Inozu (2023)^a^	Turkey/Europe-Asia	397	20.84 (2.22)	26.40	397 college students	CTQ–25 – Turkish version	EN	SCS–24, DTS–15, SDS-R–22-Turkish version	Distress tolerance, self-compassion, self-disgust	NA
Feinauer et al. (1996)^a^	USA/North America	255	NA (NA)	0	255 non-clinical sample	SAS	SA	PVS	Adjustment, hardiness	NA
Fereidooni et al. (2023)	The Netherlands and New Zealand/Europe, Oceania	2156	19.94 (2.89)	0	2156 college students	CTQ-SF	Overall CM	CD-RISC, CSI, DERS, MEMS, PTGI	Global/trait RES, Coping, ER, WB	NA
Festinger and Baker (2009)	USA/North America	253	41.5 (NA)	1.58	253 child welfare staff	CTQ	EA, EN	SWLS–5, RSES–10	WB, self-esteem	NA
Fitzgerald and Barton (2022)^a^	USA/North America	183	28.67 (10.23)	8.30	183 college students	CTQ-SF–28	Overall CM	TSS–25	Self-qualities (e.g. compassion), Self-leadership qualities	NA
Fitzgerlad and Esplin (2023)	USA/North America	1345	50.42 (13.66)	54.30	1345 college students	CTs	EA, PA	Validated questionnaire developed by authors for WB, MPQ	ER, WB	Gender, race, education, physical health, living with an alcoholic as a child
Fleming et al. (1999)	Australia/Oceania	710	38.6 (10.6)	0	124 women with alcohol problem, 586 women without an alcohol problem	Authors questionnaire based on WSHQ	SA	RSES–10	Self-esteem	NA
Fossati et al. (2015)	Italy/Europe	354	34.29 (14.88)	41.50	354 community-dwelling adults	CATS–38-Italian version	EA, PA, SA	DERS–36-Italian version	ER	Age, gender
Fosse and Holen (2007)	Norway/Europe	160	32.6 (9.52)	33.00	160 psychiatric outpatients	Olwesus (1991) Inventory for school children; CTQ–21	SA, EN, Bullying	RSES–10, LOC–17	Self-esteem	Age, gender
Fox and Gilbert (1994)	USA/North America	253	19.33 (2.9)	0	253 college students	FCVQ	PA, SA	RSES	Self-esteem	Social desirability (Crowne-Marlowe score)
Galea et al. (2007)	Malta/Europe	312	20.45 (2.37)	31.40	312 college students	CTQ–28-Maltese version	Overall CM	ABS–10-Maltese version, SWLS–5-Maltese version, STS–24-Maltese version, RPS	WB	NA
Gambaro et al. (2020)	Italy/Europe	119	29.4 (10.52)	85.70	119 migrants: 64 with depressive symptoms; 69 with anxiety symptoms; 63 (53.39%) with PTSD symptoms, 84 (70.59%) with insomnia, 13 (10.92%) with a lifetime history of suicide attempts, 30 (25.21%) with a current medical diagnosis	CTQ–28	Overall CM	CD-RISC–25	Global/trait RES	NA
Garcia and Berzenski (2023)^a^	USA/North America	405	19.44 (2.12)	30.60	405 college students	CTQ-SF–28	EN, PN	RSA–33, ATQ-SF–77, RLOC–29	Sociability, locus of control, social competence	NA
Garofalo et al. (2024)	The Netherlands/Europe	521	35.27 (15.99)	40.10	521 individuals from the general community	CTQ-SF–28-Dutch version	Overall CM	CD-RISC–10-Dutch version	Global/trait RES	NA
Goldbach et al. (2023)	Germany/Europe	187	29.84 (8.21)	0	121 (65%) with BPD, 22 (12%) with dysthymia, 8 (4%) with substance misuse, 7 (4%) with OCD, 17 (9%) with panic disorder, 36 (19%) with social phobia, 65 (35%) with PTSD, 143 (76%) currently in treatment, 26 (14%) without mental disorder	CTQ–28-German version	EA, PA, SA, EN, PN	DERS–36-German version	ER	NA
Goldstein et al. (2013)	Canada/North America	93	19.46 (1.27)	23.70	93 (100%) from child welfare	CTQ-SF–25	EA, PA, SA, EN	CD-RISC–25	Global/trait RES	Age, gender
Goodboy et al. (2016)^a^	USA/North America	149	18.25 (0.87)	48.32	149 college students	PECK–32	Bullying	AMS–28, SACQ–67	Motivation, adjustment	NA
Griffing et al. (2006)	USA/North America	219	26.77 (6.23)	0	86 women with a history of child SA, 133 without a history of child SA	CTQ–28	SA	CSI-SF–32, RSES–10	Coping (with DV), Self-esteem	NA
Güler et al. (2023)	Turkey/Europe-Asia	395	35 (10)	48.90	395 adults from the community	CTQ–28-Turkish version	Overall CM, EA, PA, SA, EN, PN	CERQ–36-Turkish version, CD-RISC–25-Turkish version.	Global/trait RES, ER	NA
Guo et al. (2022)	China/Asia	447	20.05 (1.61)	23.94	447 college students: 149 with CM, 298 without CM	CTQ-SF–25-Chinese version	Overall CM	SWLS–5, SCSQ–20, RSES–10, THS	Coping, Self-esteem, WB	Age, gender, family structure (intact or non-intact)
Haj-Yahia et al. (2021)	Israel/Middle East	516	24.9 (2.70)	9.30	516 college students	CTs	EA, PA, DV, Abuse without SA	TSES	Self-efficacy	NA
He et al. (2022)	China/Asia	937	28.51 (11.1)	41.60	459 (48.99%) with psychoactive substance abuse or dependence, 478 (51.01%) HCs 734 with CM, 203 without CM	ACE-Q–10	Abuse, neglect	CD-RISC–25	Global/trait RES	NA
Hengartner et al. (2013)	Switzerland/Europe	511	NA (NA)	NA	511 individuals from the general population	CTQ–28 – German version	EA, PA, SA, EN, PN	Brief-COPE	Coping	NA
Herrenkohl et al. (2012)	USA/North America	357	NA (NA)	52.10	357 from child welfare agencies	Official records of CM, parent reports of PA, observers ratings of EN and PN in parent–child interactions	Overall CM, PA, neglect	Validated questionnaire from the MIDUS study, RSES–10	Self-esteem, WB	Childhood SES, gender
Heshmati et al. (2021)	Iran/Middle East-Asia	250	24.72 (4.37)	39.20	250 college students	CASRS–38	Overall CM without EN, EA, PA, SA, PN	PANAS–20	WB	NA
Higgins and McCabe (1994)	Australia/Oceania	199	20.95 (NA)	0	199 college students, 47 with CM	FSHQ	SA	RSES–10	Self-esteem	NA
Hu et al. (2024)	China/Asia	4302	19.92 (1.42)	41.10	4302 college students: 1814 with PLEs, 2488 with no-PLEs	CTQ–28-Chinese version	Overall CM	CD-RISC–10-Chinese version	Global/trait RES	NA
Ion et al. (2023)	Romania/Europe	118	19.65 (NA)	17.24	118 healthy volunteers	CTQ-SF–25	Overall CM	Experience sampling questionnaire adapted from PANAS/ERQ/RSQ	ER, WB	Mean strategy endorsement
Janiri et al. (2021)	Italy/Europe	500	NA (NA)	40.40	148 (29.6%) with lifetime history of chronic diseases, 190 with COVID–19-related psychological distress	CTQ-SF–28	EA, PA, SA, EN, PN	DERS–36	ER	Age and sex
Jennissen et al. (2016)	Germany/Europe	701	27.82 (9.94)	23.40	434 (61.9%) with at least one type of CM, 26% with a current mental disorder, 32.4% with a past mental disorder	CTQ–28 – German version	Overall CM, EA, PA, SA, EN, PN	DERS–36 – German version	ER	Negative affect
Johnson (2001)	USA/North America	120	NA (NA)	0	60 with CM, 60 without CM, 57 (95%) with depressive symptoms, 41 (68%) with thoughts about death, 39 (65%) with suicidal thoughts	Research standardised inventory interview	SA	CFSEI–2–60	Self-esteem	NA
Jones et al. (2023)	USA/North America	331	40.24 (6.24)	49.50	331 adults from the community	CTQ–28	Overall CM, abuse (EA, PA, SA), neglect (EN, PN)	ERQ–10	ER	Baseline levels of systemic inflammation, age, sex, race
Jonzon and Lindblad (2006)	Sweden/Europe	152	41 (9.4)	0	152 from a non-clinical group	Research standardised questionnaire	PA, SA	CW, SES	Coping, Self-esteem	Health measures, lifestyle variables, and additional trauma (bullying)
Kanai et al. (2016)	Japan/Asia	415	42.3 (12)	53.50	415 general nonclinical adult population	CATS–38-Japanese version	Overall CM, neglect, abuse	SUBI–40-Japanese version	WB	NA
Kanj et al. (2023)	Lebanon/Middle East-Asia	411	32.86 (11.98)	24.60	411 adults from the community	CTQ-SF–28-Arabic version	EA, PA, SA, EN, PN	DERS–16-Arabic version	ER	NA
Kapoor et al. (2018)^a^	USA/North America	121	36.07 (11.03)	0	121 (100%) with a history of IPV and suicide attempt	CTQ-SF–25	Abuse	SRI–25, SWBS–20, SESBW–12	(Suicide) Global/trait RES, Self-efficacy, WB	Intrapersonal strengths
Karagöz and Dağ (2015)	Turkey/Europe-Asia	79	41.7 (10.50)	100	28 SUD with self-mutilation, 51 SUD without self-mutilation	CTQ-Turkish version	EA-EN, PA, SA	DERS–36 – Turkish version	ER	NA
Karakaş and Çingöl (2022)^a^	Turkey/Europe-Asia	359	20.42 (1.85)	15.30	359 college students	CTQ–40-Turkish version	Overall CM, EA-EN, PA, SA	SOCS–13 – Turkish version	Sense of coherence	NA
Kazan Kizilkurt et al. (2021)	Turkey/Europe-Asia	80	31.9 (4.0)	0	80 adults with fibromyalgia	CTQ–28-Turkish version	EA, PA, SA, EN, PN	RSA–33 – Turkish version	Global/trait RES	NA
Kesebir et al. (2015)	Turkey/Europe-Asia	100	32.7 (13.2)	46.00	35 (35%) with CM, 100 (100%) with BD type 1	CTQ-Turkish version	EA, PA, SA, EN, PN	RSA–33 – Turkish version	Global/trait RES	NA
Khosravani et al. (2019)	Iran/Middle East-Asia	329	33.45 (8.69)	100	329 (100%) with AUD, 120 (36.5%) with comorbid psychiatric disorders: 45 (13.7%) MDD, 35 (10.6%) BD, 21 (6.4%) PTSD, 19 (5.8%) anxiety disorders.	CTQ-SF–28-Persian version	Overall CM	CERQ-Short–18 – Persian version	ER	Depression, age of onset of alcohol use, duration of alcohol use
Kim E. Y., et al. (2016)	Korea/Asia	183	40.1 (11.8)	58.47	107 with CM, 100% adult probationers, 60 (56.1%) with at least one psychiatric diagnosis	CTQ–28-Korean version	Overall CM	CD-RISC–25, DERS–36 – Korean version	Global/trait RES, ER	NA
Kim, M., et al. (2021)^a^	South Korea/Asia	212	39.9 (13.3)	17.92	212 crime victims with PTSD	CTQ	Abuse, neglect	CD-RISC, Brief COPE	Global/trait RES, Coping	NA
Kiziltepe et al. (2023)	Turkey/Europe-Asia	421	21.16 (1.79)	23.30	421 college students	CTQ-SF–28-Turkish version	EA, PA, SA, EN, PN	RSES–10 – Turkish version	Self-esteem	Perceived SES, sex, age, SA, PA, EN, PN
Koçak and Çağatay (2024)	Turkey/Europe-Asia	400	42 (6.91)	35.00	400 adults from the community	CTQ–33-Turkish version	overall CM	DERS–36, RSES–10 – Turkish version	Self-esteem, ER	NA
Kong et al. (2024)	USA/North America	4736	54 (NA)	47.23	4736 random sample of individuals from the Wisconsin Longitudinal Study	CTs	Overall CM (without SA, PN)	Ryff’s scales of psychological WB	WB	NA
Krause-Utz et al. (2023)	Multi-country: Asia, Europe, Middle East, North America, South America, Other	445	25.29 (10.22)	29.00	16 from Asia, 366 from Europe 38 from Middle East, 6 from North America, 5 from South America, 14 from other countries, 100% with a history of IPV, 50 (11.2%) with BPD features, 50 (11.2%) with trait dissociation	CTQ–25	Overall CM	BERQ–25, CERQ–36	ER	Before versus after the start of the pandemic
Krvavac and Jansson (2021)	Norway/Europe	133	27.81 (12.99)	42.86	133 college students and staff with alexithymia	CTQ	Overall CM, EA, PA, SA, EN, PN	DERS–36	ER	NA
Kumar et al. (2022)	USA/North America	491	21.74 (2.23)	0	491 from a multi-wave, multi-site community setting: 186 (37.9%) mild to severe CM	CTQ-SF–28	SA	DERS–36, FFMQ–39	ER, WB	NA
Kuo et al. (2015)	Canada/North America	243	20.1 (4.74)	14.40	243 college students (psychology), including individuals ranging in BPD severity	CTQ-SF–28	EA, PA, SA	DERS–36	ER	NA
Kurtuluş and Elemo (2023)	Turkey/Europe-Asia	385	NA (NA)	37.40	385 college students	CTQ–28-Turkish version	EN	MPLS–17-Turkish version	WB	NA
Lacelle et al. (2012)	Canada/North America	889	21.2 (NA)	0	889 adults from the community, 280 with CM, 609 without CM	ACE-Q–5, SVCQ	SA	HOPES–20, CISS–48 – French version	Coping, WB	NA
Laghaei et al. (2023)	Iran/Middle East-Asia	372	20.75 (2.25)	42.70	372 college students	CTQ-SF–28-Iranian version	Overall CM, EA, PA, SA, EN, PN	S-DERS–21-Iranian version	ER	NA
Lassri et al. (2023)^a^	Israel/Middle East	65	25.59 (3.89)	0	65 high-functioning young adults: 35 with CM, 30 without CM	CTQ–28, SES-SFV, PDS	SA	SCC–12	Self-concept clarity	NA
Latzer et al. (2020)	Israel/Middle East	426	35.56 (12.91) eating disorder, 33.63 (10.27) HCs	0	158 with eating disorder, 268 HCs	CTQ–28	EA, PA, SA, EN, PN	RSES–10	Self-esteem	NA
Lewis et al. (2006)	USA/North America	102	27.17 (6.63)	0	102 (100%) residents from emergency DV shelters	CTQ	EA, EN	RSES–10	Self-esteem	NA
Li, B., et al. (2020)	China/Asia	1622	20.02 (1.96)	36.10	1622 healthy college students	CTQ-SF–28 – Chinese version	Overall CM	RSES–10 – Chinese version	Self-esteem	NA
Li, Chao, et al. (2023)	China/Asia	217	33.08 (8.32)	54.00	101 with MDD: of them 57 with CM; 116 HCs: of them 55 with CM	CTQ–28 – Chinese version	Overall CM, EA, PA, SA, EN, PN	CD-RISC–25 – Chinese version	Global/trait RES	Age, sex, education, HDRS score, Hamilton anxiety rating scale score, MDD total history, MDD episodes
Li, Chengcheng, et al. (2023)	China/Asia	349	Discovery sample: 20.48 (1.53), Replication sample: 20.43 (1.94)	Discovery sample: 16.67, Replication sample: 18.34	349 emerging adults: 120 from the discovery sample, 229 from the replication sample	CTQ-SF	Overall CM (without SA)	RSES–10, SWLS, SPANE	Self-esteem, WB	Sex, age, SES
Li, Cun, et al. (2023)	China/Asia	6057	34 (NA)	60.01	6507 individuals recruited across China the internet	CTQ–28 – Chinese version	Overall CM	ERQ–10, RSES–10 – Chinese version	Self-esteem, ER	Age, sex
Li, W., et al. (2023)	China/Asia	1069	20.57 (1.24)	53.60	1069 college students	CTQ-SF–28 – Chinese version	Overall CM	GSES–10-Chinese version	Self-efficacy	NA
Liu, J., et al. (2024)	Singapore/Asia	200	36.5 (12.5)	46.00	144 (72%) MDD, 56 (28%) BD, 27 (13.5%) psychiatric comorbidity	CTQ-SF–28	Overall CM	DERS-SF–18	ER	NA
Liu, S. et al. (2023)	China/Asia	1929	18.49 (0.80)	36.90	1929 youth participants	CTQ-SF–28	EA, PA, SA, EN, PN	ERQ–10 – Chinese version	ER	NA
Lu, Wen, Deng, and Tang (2017)	China/Asia	816	34.59 (8.53)	67.40	816 drug addicts	CTQ-SF–28-Chinese version	Overall CM, EA, PA, SA, EN, PN	GSES–10 – Chinese version, TSCS–70 – Taiwan version	Self-efficacy, Self-concept	Age, gender
Maftei and Nițu (2024)	Romania/Europe	178	22.5 (6.74)	19.66	178 adults from the community	CTQ-SF–12	EA, PA, SA	ERQ–10	ER	NA
Mandavia et al. (2016)	USA/North America	2014	39.84 (12.4)	28.10	2014 low socioeconomic, primarily African American urban population	CTQ–25	EA, PA, SA	EDS–12	ER	NA
Martin et al. (2023)	USA/North America	241	NA (NA)	0	241 mother and adolescent child dyads	CTQ–28	Maternal Overall CM	DERS–36	(maternal) ER	NA
Martínez et al. (2023)	Chile/Latin America	178	36.9 (13.7)	30.40	178 (100%) with MDD: 46.7% severe MDD	CTQ-SF–28-Chilean version	Overall CM	DERS–36-Chilean version	ER	Sex, age
Martxueta and Etxeberria (2014)	Spain/Europe	119	37.9 (8.24)	71.40	96.6% homosexuals: of them 29.41% with anxiety symptoms, 28.57% with depressive symptoms, 51.3% with bullying related to emotional-sexual orientation	OBVQ–12- adapted for high school students	Bullying	RSES–10, PANAS – Spanish version	Self-esteem, WB	NA
Maxwell and Huprich (2014)	USA/North America	599	22.32 (6.10)	23.54	599 undergraduate students	CTQ–28	Overall CM, EA, PA, SA, EN, PN	RSES–10	Self-esteem	Gender
Merians and Frazier (2024)	USA/North America	312	20.28 (2.47)	20.00	312 undergraduate students (psychology)	CTQ-SF–28	Overall CM	DERS–36, MLQ–5, Ryff (1989) Scales of Psychological WB’s autonomy subscale–9	ER, WB	NA
Mohammadpanah Ardakan et al. (2024)	Iran/Middle East-Asia	300	30.22 (6.25)	36.00	300 (100%) with OCD, 115 (38.3%) with anxiety and MDD	CTQ-SF–28	EA, EN	TCAQ–25, AAQ-II–7, ERS–10	ER	NA
Mohammadzadeh et al. (2019)	Iran/Middle East-Asia	310	34.58 (9.6)	100	310 with SUD, 10 with psychotic disorder, 80 with MDD, 40 with BD, 35 with anxiety disorder, 10 with BPD	CTQ-SF–28-Persian version	Overall CM	DERS–36-Persian version, CERQ-Short–18-Persian version	ER	NA
Mondolin et al. (2024)	Finland/Europe	4950	Pregnant mothers 30.4 (4.5), Fathers 32.1 (5.3)	39.07	3016 pregnant mothers, 1934 fathers	TADS–43	Overall CM	CD-RISC–10	Global/trait RES	NA
Moreira et al. (2024)	Portugal, Brazil/Europe, Latinamerica	846	30.9 (0.49)	29.31	846 adult participants from the general population	ACE-Q-Portuguese version, CTQ	EA, PA, SA, EN, PN	DERS–36 – Portuguese version	ER	NA
Musella et al. (2024)	USA/North America	193	19.5 (NA)	22.00	193 college students with social anxiety symptoms: 35 (17.8%) mild, 37 (19.2%) moderate, 18 (9.4%) severe, 11 (5.8%) very severe	CTQ–28	Overall CM	ERQ–10, AAQ-II–7	ER	NA
Naderzadeh et al. (2023)^a^	Iran/Middle East-Asia	237	69.23 (6.87)	60.30	237 community-dwelling older adults	CTQ neglect subscale, CTs EA-PA subscale	neglect, abuse without SA	SOCS–13-Persian version	Sense of coherence	Sex, age, marital status, educational level, income
Naughton et al. (2020)^a^	Ireland/Europe	355	20.07 (2.08)	29.40	355 college students	CEDV	DV	GHQ–12	WB	NA
Newman et al. (2011)^a^	USA/North America	1339	18.8 (1.8)	33.00	1339 college students	OBVQ	Bullying	COPE	Coping	NA
Nimphy et al. (2024)	The Netherlands/Europe	250	51.3 (13.7)	41.20	100% with experienced and perpetrated abuse from three generations families	CTs-PC	EA, PA	CERQ	ER	NA
Ozakar Akca et al. (2021)	Turkey/Europe-Asia	3602	NA (NA)	NA	3602 college students	CTQ–28-Turkish version	Overall CM, EA, PA, SA, EN, PN	RSES–10-Turkish version	Self-esteem	NA
Pabian, Dehue, Völlink, and Vandebosch (2022)	Belgium and The Netherlands/Europe	1660	21.73 (2.24) Belgium, 21.61 (2.33) The Netherlands	42.2 Flemish-Belgium, 21.39 The Netherlands	1010 from Flemish-Belgium: of them 664 with CM; 650 from The Netherlands: of them 317 with CM	Authors questionnaire adapted from OBVQ	Bullying	Questionnaire by Przybylski et al. (2013), RSES–10-Dutch version	Self-esteem, WB	NA
Park et al. (2023)	Korea/Asia	1521	36.29 (11.65)	37.50	787 (51.74%) psychiatric patients: 247 MDD, 120 BD type 1, 420 BD type 2; 734 individuals from the general population	CTQ- SF–28	Overall CM, EA, EN	CD-RISC–25	Global/trait RES	Age, sex, education, employment, marital status, smoking status, alcohol use status, psychiatric family history
Peng et al. (2020)	China/Asia	619	24.96 (11.19)	43.78	175 (28.27%) MDD; 138 (22.29%) anxiety disorder; 113 (18.26%) personality disorder: of them 43 (38.05%) BPD; 193 (31.18%) other psychiatric disorders	CTQ–28	EA, PA, SA, EN, PN	CERQ–36	ER	Depression, anxiety, age, subjective family status, subjective social status
Pourshahriar et al. (2018)	Iran/Middle East-Asia	312	22.9 (3.1)	41.02	312 college students	CTQ–45-Persian version	EA-EN	DERS–36-Persian version	ER	NA
Qin et al. (2024)	China/Asia	1272	19.71 (1.93)	39.15	1272 college students: 544 with depressive symptoms, 728 without depressive symptoms	CTQ–28-Chinese version	Overall CM	CERQ–36-Chinese version	ER	NA
Racine and Wildes (2015)	USA/North America	188	26.44 (10.03)	4.30	188 (100%) with anorexia nervosa: 105 (55.9%) with AN-binge/purge, 83 (44.1) with anorexia nervosa-restricting	CTQ-SF–28	EA, PA, SA	DERS–36	ER	NA
Richardson et al. (2023)	UK/Europe	189	30.97 (13.83)	23.30	21 (11.11%) with MDD, 31 (16.4%) with anxiety, 8 (4.23%) with PTSD, 46 (24.3%) with BD, 7 (3.7%) with OCD, 2 (1%) eating disorder	CTQ–28	Overall CM	DERS–16	ER	NA
Rodriguez et al. (2021)	USA/North America	110	30.81 (6.08)	0	110 mothers from a community sample	CTQ–28	Overall CM	DERS–36	ER	NA
Romans et al. (1995)	New Zealand/Oceania	320	NA (NA)	0	138 (43.13) with CM: 20 (14.5%) with depression, 1 (0.7%) with anxiety, 10 (7.2%) with phobia, 1 with mania	Validated questionnaire from The Otago Women’s Health Survey Child SA study	SA	Robson Self-esteem Questionnaire–30	Self-esteem	NA
Rong et al. (2023)	China/Asia	1040	23.72 (2.49)	67.12	1040 (100%) juvenile prisoners: 139 (13.4%) with NSSI	CTQ-SF–28-Chinese version	Overall CM, EA, PA, SA, EN, PN	RSES–10-Chinese version	Self-esteem	NA
Rostami et al. (2023)	Iran/Middle East-Asia	331	28.75 (7.73)	20.50	331 healthy adults	CTQ–28-Iranian version	EA, PA, SA, EN, PN	DERS–36-Iranian version, LOCS–22-Iranian version	ER	NA
Sachs-Ericsson et al. (2011)^a^	USA/North America	1396	67.1 (10.2)	42.30	Adults aged 50 and over: 6.4% with CM, of them: 65% physically disabled	CIDI PTSD module	Abuse	PMS	Self-efficacy	NA
Salles et al. (2023)	France/Europe	220	52.6 (13.1)	40.00	139 (63%) with CM, 82 (37%) without CM, 220 (100%) with TRD, 78 (35%) with a history of suicide attempts	CTQ	Overall CM	RSES	Self-esteem	NA
Schulz et al. (2014)	Germany/Europe	2046	56 (13.9)	NA	2046 from a community based sample: 1167 (57%) with CM, 262 (12.8%) with lifetime MDD	CTQ–28-German version	Overall CM	RS–25-German version	Global/trait RES	Sex, age
See Mey et al. (2022)	Malaysia/Asia	360	33.34 (7.25)	100	360 (100%) with SUD	CTQ-SF–28-Malay version	Overall CM, EA, PA, SA, EN, PN	GSES–10, HFS–18	Self-efficacy	NA
Sehlikoğlu et al. (2022)	Turkey/Europe-Asia	146	28.23 (6.7)	100	73 with SUD: 15 (20.5%) with severe MDD, 32 (43.8%) with PD, 28 (87.5%) with antisocial personality disorder, 38 (52.1%) with self-mutilation, 22 (30.1%) with suicide attempt, 33 (45.2%) with history of psychiatric treatment; 73 HCs: 3 (4.1%) with self-mutilation	CTQ-SF–28-Turkish version	EA, PA, SA, EN, PN	RSES–63	Self-esteem	NA
Sexton et al. (2015)	USA/North America	214	28.2 (5.7)	0	214 4-month postpartum mothers	CTQ–28	Overall CM	CD-RISC–25	Global/trait RES	NA
Sezer Katar et al. (2023)	Finland/Europe	95	31.4 (6.28)	91.60	95 patients with OUD, 83 HCs	CTQ–33 – Turkish version	Overall CM, EA, PA, SA, EN, PN	CD-RISC–25 – Turkish version	Global/trait RES	NA
Shen (2009)	Taiwan/Asia	1924	20.5 (NA)	48.60	1924 college students, 116 (6%) with PA only, 370 (19.2%) with DV only	CTs-PC, CTs Form-R -Taiwanese version	PA, DV, PA-DV	RSES–10-Chinese version	Self-esteem	Sex, age, family income, parents divorced, self-blame, other family risks, Chinese traditional beliefs
Shen and Soloski (2022)	USA/North America	767	33.16 (13.03)	24.10	767 adults: 427 (55.67%) with CM, 340, 44.33% without CM	SEQ-modified version	SA	RSES–10	Self-esteem	Age, gender, race
Shin and Brunton (2024)^a^	Australia/Oceania	316	Study 1: 35.9 (13.6); Study 2: 34.8 (11.4)	54.70	316 college students: 176 participants in the Study 1; 140 participants in the Study 2	CCMS, ACE-Q	Abuse, neglect	BRS–6	Global/trait RES	NA
Simeon et al. (2007)	USA/North America	54	33.2 (11)	53.70	54 healthy adults	CTQ-SF–25	Overall CM, EA, PA, SA, EN, PN	DSQ	Global/trait RES	Age, gender
Simon et al. (2009)	USA/North America	103	36.69 (14.1)	69.90	103 (100%) with GSAD, 27 (26.21%) with GAD, 8 (7.77%) with panic disorder, 2 (1.94%) with PTSD, 21 (20.39%) with MDD	CTQ–28	Overall CM	CD-RISC–25	Global/trait RES	Age, gender
Sistad et al. (2021)	USA/North America	586	19.58 (1.57)	29.30	586 college students	CATS–38	Overall CM	ERQ–10, PANAS–20	ER, WB	Gender
Soffer et al. (2008)	Israel/Middle East	203	23.6 (1.86)	15.27	203 college students	CTQ–28	EA, PA, SA, EN, PN	GSES–10, PSI, DEQ-SC	Self-efficacy	NA
Șoflău et al. (2023)	Romania/Europe	419	27.32 (8.98)	11.90	419 from a community sample	CTQ–28	Overall CM	BRS–6	Global/trait RES	NA
Somers, Ibrahim, and Luecken (2017)	USA/North America	150	19.7 (2.1)	39.33	150 college students	CTQ–25	Overall CM	PANAS–10	WB	Sex
Stevens et al. (2013)	USA/North America	139	28.46 (7.76)	0	44.6% with at least one type of CM, 12% with PTSD symptoms	CTQ–28	Abuse	DERS–36	ER	NA
Su et al. (2022)	Canada/North America	25113	NA (NA)	45.20	1642 (65.4%) with chronic conditions	CEVQ–18, validated questionnaire adapted from CCHS-MH–2002	PA, SA, DV, PA-SA-DV	CCHS-MH–2012	Coping	NA
Sun Yujing et al. (2023)	China/Asia	300	39.6 (8.6)	56.30	300 with schizophrenia, 242 (80.67%) with CM	CTQ-SF–28 – Chinese version	Overall CM, EA, PA, SA, EN, PN	CD-RISC–25-Chinese version, RSES–10-Chinese version	Global/trait RES, Self-esteem	NA
Suresh and Tipandjan (2012)	India/Asia	95	NA (NA)	65.26	95 college students	RBQ	Bullying	CAs self-esteem subscale	Self-esteem	NA
Švecová et al. (2023)	Slovak Republic/Europe	1018	46.24 (NA)	48.70	1018 adults from a representative sample of the population	CTQ–25, ACE-IQ – Slovak version	Overall CM, EA, PA, SA, EN, PN, Bullying	BRS–6	Global/trait RES	NA
Swaminath et al. (2023)	USA/North America	603	19.62 (1.59)	29.35	603 college students	CATS–38	Overall CM	PANAS–20	WB	Sex and negative affect
Talmon et al. (2022)^a^	Israel/Middle East	316	72.24 (8.12)	32.30	316 older adults	ICES–12	EA	PMS–7	Mastery	Age, gender, relational status, education
Tarber et al. (2016)	USA/North America	182	26.51 (11.04)	100	182 adults from the community, 68 (37.36%) with CM	Research questionnaire–5	Overall CM	TSPWB–54, SCS-SF–12	WB	NA
Theran and Han (2013)	USA/North America	257	19.74 (2.11)	0	257 college students	CTQ–28	Physical abuse (PA-PN), emotional abuse (EA-EN)	RSES–10	Self-esteem	NA
Thoma et al. (2021)	Switzerland/Europe	257	70.72 (11.08)	53.70	132 with a history of placements from child welfare: 56.8% with a current mental disorder, 84.1% with lifetime mental disorder, 125 HCs	CTQ–28 – German version	EA, PA, SA, EN, PN	RSES–10, SCS-SF–12-German version	Self-esteem	Age, gender
Tinajero et al. (2020)	USA/North America	79	27 (6.50)	32.00	79 healthy adults, 46 with at least some CM	CTQ	Abuse, neglect	DERS–41	ER	Age, sex, years of education
Toker et al. (2011)	Turkey/Europe-Asia	82	SUD 34.8 (10.51); HCs 38.9 (8.74)	100	41 with SUD: 5 with MDD, 3 with PTSD, 1 with dysthymia, 2 with GAD; 41 HCs	CTQ–40 – Turkey version	Overall CM without PN, emotional maltreatment (EA-EN), PA, SA	COPE-Turkish version, RSES–63-Turkish version	Coping, self-esteem	NA
Upenieks et al. (2024)	USA/North America	858	61.19 (8.84)	51.28	858 adults from the US South Asians cohort, 28 (3.26%) with anti-depressive medication use	CTQ–28 items	EA, EN, PN, Overall CM (EA-EN-PN)	SSSH	Coping	Gender, income, education, marital status, employment status, language spoken at home, self-rated health, anti-depressant medication, percent life in the USA, childhood parent home ownership, and religious affiliation
Ustuner Top and Cam (2021)	Turkey/Europe-Asia	626	20.88 (1.86)	17.40	626 college students, 272 with CM	CTQ-SF–28 – Turkish version	Overall CM	DUKE–17 – Turkish version	Self-esteem	NA
Valencia and De la Rosa-Gómez (2024)	Mexico (North America)	375	22.03 (2.62)	22.90	375 adult participants from the community	EAIA–14 – Mexican version	EA, PA, SA	ERQ-CA–9 – Mexican version	ER	NA
van Schie et al. (2024)	Multi-country: Europe, America, Asia, Middle East	374	24.04 (7.45)	32.00	76 (20%) with BPD features, 80 (21%) with current treatment for mental health, 287 (77%) with intentional use of self-harm, 36 (10%) with previous suicide attempt, 75 (20%) with dissociative symptoms	CTQ-SF–28	EA, PA, SA, EN, PN	CERQ-Short–18	ER	Age, gender
Vancappel et al. (2023)	France/Europe	90	36.17 (13.71)	15.56	90 (100%) PTSD, 28.9% MDD, 10% BPD, 4.4% bulimia, 1.10% schizophrenia spectrum disorder, 3.3% adjustment disorder, 1.1% autism spectrum disorder, 2.2% social anxiety, 3.3% GAD, 2.2% non-epileptic psychogenic crisis, 2.2% AUD, 1.1% panic disorder, 1.1% BD, 1.1% dissociative identity disorder	CTQ–28-French version	Overall CM	FFMQ–39-French version, Difficulties in DERS–36-French version	ER	NA
Vettese, Dyer, Li, and Wekerle (2011)	Canada/North America	81	19.49 (2.32)	65.40	81 (100%) with SUD, 87.7% poly-substance users, 29.6% in the criminal justice system	CTQ-SF–28	Overall CM	DERS–36, SCS–26	ER	NA
Volgenau et al. (2022)	USA/North America	2094	Study 1: 54.55 (11.73), Study 2: 50.79 (13.41)	Study 1: 43.5, Study 2: 47.8	Study 1: 1239 adult participants; Study 2: 855 participants	CTQ–25	EA, PA, SA, EN, PN	MASQ, SWS	WB	NA
Wadji et al. (2023)	Multi-country: Cameroon, Canada, Germany, Japan	478	Cameroon 35.65 (8.34), Canada 34.39 (10.81), Germany 28.86 (9.75), Japan 52.45 (14.13)	34.22	Multi-country study: 478 general population sample	ICAST-R–5, ETISR-SF, CTs–2-English, French, German, Japanese versions	Neglect, EA, PA, SA, DV	BRS–6, PTGI-SF–10; PTGI-SF–21 French, German, Japanese versions	Global/trait RES, Post-traumatic growth	NA
Walker et al. (2023)	USA/North America	744	21.48 (4.12)	19.10	744 college students, 56% with CM	LSC-R–30	Overall CM	DERS-SF–18	ER	Recruitment site, income, age, sex, race
Walsh et al. (2011)	USA/North America	160	35.4 (9.3)	0	160 incarcerated women	CTQ–28	SA	DERS–36	ER	NA
Wang, Z., et al. (2023)	China/Asia	809	37.39 (8.81)	100	767 male prisoners	CTQ–28 – Chinese version	EA, PA, SA, EN, PN	SCSQ–20, RSES–10-Chinese version	Coping, Self-esteem	NA
Wang, Z., et al. (2022)	China/Asia	767	20.58 (1.7)	42.10	176 (22.9%) with suicidal risk state	CTQ-SF – Chinese version	Overall CM	MLQ–5 – Chinese version	WB	NA
Whittington (2023)	USA/North America	318	19.16 (1.73)	17.00	318 college students	ACE-Q–10	Overall CM without SA	DERS-SF–18	ER	NA
Wind and Silvern (1994)^a^	USA/North America	259	40.7 (NA)	0	259 female university staff	CTs	DV, PA-SA	CSEI	Self-esteem	NA
Wolff et al. (2016)	Germany/Europe	159	37.93 (11.65)	52.83	105 with SUD, 54 HCs	CTQ-SF – German version	Overall CM, EA, PA, SA, EN, PN	DERS–36-German version	ER	NA
Wong et al (2024)	USA/North America	853	22.43 (4.93)	23.80	853 college students, 68 (8%) with a history of suicide attempt, 31.5% with a high risk of suicidality	CTQ–28	Overall CM	DERS–36	ER	Depression symptoms, race/ethnicity
Wu, C. et al. (2023)	China/Asia	1350	18.64 (1.06)	39.48	1350 college students	CTQ–28 – Chinese version	Overall CM, EA, PA, SA, EN, PN	RESE–17-Chinese version	Self-efficacy, ER	NA
Wu, Q., et al. (2022)	China/Asia	358	19.18 (1.46)	36.87	358 college students	CTQ-SF–28 – Chinese version	EA, PA, SA, EN, PN	SWLS–5, RSES–10, SCS–26	Self-esteem, WB	Age, gender, PA, SA, EN, PN
Xiang Y., et al. (2020)+	China/Asia	811	19.54 (1.86)	26.76	811 college students	CTQ–23 – Chinese version	Overall CM without SA	CD-RISC–10-Chinese version	Global/trait RES	NA
Xiang Y., et al. (2021)^b^	China/Asia	811	19.54 (1.86)	26.76	811 college students	CTQ–23 – Chinese version	Overall CM without SA	SWLS–5, PANAS–20 – Chinese version	WB	NA
Xiang, Y., et al. (2018)	China/Asia	426	20.63 (1.85)	33.33	426 college students	CAS–23 – Chinese version	Overall CM without SA	RSES–10-Chinese version	Self-esteem	NA
Xiao et al. (2023)	China, UK/Asia, Europe	1133	NA (NA)	China: 36.1, UK: 35.3	1133 participants from the general community (n = 544 China; n = 589 UK)	PMR–30 – Chinese version	EA, EN	RSES–10-Chinese version	Self-esteem	NA
Xie et al. (2023)	China/Asia	620	19.69 (NA)	51.45	620 college students	CTQ-SF–28 – Chinese version	Overall CM	RSES–10, SCC–12-Chinese version	Self-esteem	NA
Xu and Zheng (2022)	China/Asia	835	19.44 (1.28)	35.10	835 college students	CTQ-SF–28	EA	RSES–10	Self-esteem	NA
Xu et al. (2023)	China/Asia	47	19.1 (0.79)	48.94	47 healthy subjects, 21 (44.68%) with neglect, 26 (55.32%) without neglect	CTQ-SF–28 – Chinese version	EN	ERQ–10 – Chinese version	ER	NA
Yao et al. (2023)	China/Asia	742	24.01 (2.02)	39.89	164 adults with depressive symptoms, 130 with anxiety symptoms, 58 (7.8%) with Suicide risk	CTQ-SF – Chinese version	Overall CM, EA, PA, SA, EN, PN	CD-RISC–25 – Chinese version	Global/trait RES	NA
Yaroslavsky et al. (2022)	USA/North America	142	26.63 (10.81)	29.00	32 (23%) with CM, 71 (50%) with lifetime depressive disorder, 23% GAD, 14% social anxiety, 12% panic disorder, 12% specific phobia, 6% PTSD	Clinical interview	SA	FAM–54	ER	NA
Yilmaz and Satici (2023)	Turkey/Europe-Asia	330	25.65 (8.88)	27.30	330 participants recruited from the community	PMQ–12 – Turkish version	EA	ERQ–10, SWBS–5-Turkish version	ER, WB	Gender
Yöyen and Bozacı (2023)	Turkey/Europe-Asia	423	NA (NA)	26.00	423 healthy adult participants, 48 (11.3%) with psychological illness	CTS–33	EA, PA, SA, EN, PN	ERDS–16, SPRS–6-Turkish version	Global/trait RES, ER	NA
Yöyen and Çaylak (2023)	Turkey/Europe-Asia	451	NA (NA)	29.00	451 participants from the community	CTQ–28-Turkish version	Overall CM	ERPS–28 – Turkish version	ER	NA
Yrondi et al. (2021)	France/Europe	96	67.2 (5.7)	37.50	96 (100%) geriatric population with TRD: 50 (52.1%) with early onset MDD, 25 (26%) late-onset MDD	CTQ–28	Overall CM, EA, PA, SA, EN, PN	RSES	Self-esteem	Age, sex
Yubero et al. (2021)^a^	Spain/Europe	1122	20.82 (2.26)	21.20	1122 college students	Instrument to assess bully/victim interaction at school (Rigby & Bagshaw, 2003) adapted by Yubero et al. (2017)	Bullying	MHC-SF–3 – Spanish version	WB	NA
Zaorska et al. (2020)	Poland/Europe	165	NA (NA)	88.10	165 (100%) with AUD	CTQ-SF–28-Polish version	Overall CM,EA, PA, SA, EN, PN	DERS–36 – Polish version	ER	NA
Zhang, Rakesh, Cropley, and Whittle (2023)	China/Asia	1105	19.81 (1.34)	41.08	1105 college students	CTQ-SF–28-Chinese version	Overall CM	RESE–12 – Chinese version	Self-efficacy	NA
Zhou and Li (2024)	China/Asia	542	20.79 (1.45)	62.55	542 college students	CTQ-SF–28-Chinese version	EA, PA, SA, EN, PN	RSES–10 – Chinese version	Self-esteem	NA
Zhou, H., et al. (2024)	China/Asia	1266	18.25 (0.79)	38.50	1266 college students	CTQ-SF–25-Chinese version	Overall CM	CD-RISC–10, PTM–26-Chinese version	Global/trait RES	NA
Zhou, J., et al. (2024)	China/Asia	449	28.59 (11.63)	28.73	449 patients with MDD only, 58.34% with anxiety only, 64.17% with MDD comorbid anxiety, 54.25% with BD, 50% with OCD, 65.95% with schizophrenia, 60.63% with schizoaffective disorder, (27.2%) with suicide risk	CTQ–28-Chinese version	Overall CM, EA, PA, SA, EN, PN	RSES–10 – Chinese version	Self-esteem	Gender

*Note:* See the full list and complete publication details of the included studies in SA5 in the Supplementary Material.

Abbreviations: AAQ-II, The Acceptance and Action Questionnaire-II; ABS, The Affect Balance Scale; ACE-Q, Adverse Childhood Experiences Questionnaire; ADHD, Attention-Deficit/Hyperactivity Disorder; ALSPAC, Avon Longitudinal Study of Parents and Children; AMS, Academic Motivation Scale; AnxNOS, Anxiety Disorder Not Otherwise Specified; ARM, Adult Resilience Measure; ASD, Acute Stress Disorder; ATQ, Adult Temperament Questionnaire-Short Form; AUD, Alcohol Use Disorder; BD, Bipolar Disorder; BERQ, Behavioural Emotion Regulation Questionnaire; BFIS-9, Bullying and friendship interview schedule-9; BPD, Borderline Personality Disorder; BPNSS, Basic Psychological Needs Scale; Brief-COPE, The Brief Coping Orientation to Problems Experienced Inventory; Brief RCOPE, The Brief Religious Coping Activities Scale; BRS, The Brief Resilience Scale; BSCS, The Brief Self-Control Scale; BSE, The Beck Self-Esteem Scale; BSI, Brief Symptom Inventory; CAP, Child Abuse Potential Inventory; CAQ, Childhood Abuse Questionnaire; CAs, College Adjustment Scale; CAS, Childhood Abuse Scale; CASRS, The Child Abuse and Self Report Scale; CATS, The Child Abuse and Trauma Scale; CCHS-MH, Canadian Community Health Survey-Mental Health; CCMS, Comprehensive Child Maltreatment Scale; CD-RISC, The Connor–Davidson Resilience Scale; CEDV, Child Exposure to Domestic Violence; CERQ, Cognitive Emotion Regulation Questionnaire; CERQ-Short, Cognitive Emotion Regulation Questionnaire-Short Version; CEVQ, Childhood Experiences of Violence Questionnaire; CFSEI-2, Culture-Free Self-Esteem Inventory; CIDI, Composite International Diagnostic Interview; CISS, Coping Inventory for Stressful Situation; CM, Childhood Maltreatment; CMIS, Childhood Maltreatment Interview Schedule; CMIS-SF, Child Maltreatment Interview Schedule – Short Form; COPE, Coping Orientations to the Problems Experienced; CSAQ, Childhood Sexual Abuse Questionnaire; CSEI, Coopersmith Self-Esteem Inventory; CSI, Coping Strategies Inventory; CSI-SF, Coping Strategies Inventory–Short Form; CTI, Childhood Trauma Interview; CTQ, Childhood Trauma Questionnaire; CTQ-SF, Childhood Trauma Questionnaire-Short Form; CTs, Conflict Tactics Scale; CTS, Childhood Trauma Screener; CTS-33, Childhood Trauma Scale-33; CTs Form-R, Conflict Tactics Scales Form R; CTs-PC, Parent–Child Conflict Tactics Scales; CW, Coping Wheel; DD-NOS, Depressive Disorder Not Otherwise Specified; DEQ-SC, Depressive Experiences Questionnaire Self-Criticism; DERS, Difficulties in Emotion Regulation Scale; DERS-SF, Difficulties in Emotion Regulation Scale–Short Form; DSM, Diagnostic and Statistical Manual of Mental Disorders; DSQ, The Defense Style Questionnaire; DTS, Distress Tolerance Scale; DUKE, The Duke Health Profile; DV, Domestic Violence; EA, Emotional abuse; EAIA, Child Abuse Scale for Adults; EDS, Emotional Dysregulation Scale; EN, Emotional neglect; ERDS, Emotion Regulation Difficulty Scale-Short Form; ER, Emotion Regulation; ERPS, Emotion Regulation Process Scale; ERQ, Emotional Regulation Questionnaire; ERQ-CA, Emotion Regulation Questionnaire-modified version; ERS, Emotion Regulation Scale; ETISR-SF, Early Trauma Inventory Self-Report-Short Form; FAM, Feelings and Me Questionnaire; FCVQ, Finkelhor Childhood Victimisation Questionnaire; FFMQ, Five Facet Mindfulness Questionnaire; FSHQ, Family and Sexual History Questionnaire; GAD, General Anxiety Disorder; GHQ, General Health Questionnaire; GSAD, Generalised social anxiety disorder; GSES, General Self-Efficacy Scale; HCs, Healthy controls; HFS, The Heartland Forgiveness Scale; HIV, Human immunodeficiency virus; HOPES, Hunter Opinions and Personal Expectations Scale; IBS, Impulsive Behaviour Scale; ICAST-R, The ISPCAN Child Abuse Screening Tools Retrospective-Version; ICES, Invalidating Childhood Environments Scale; ID, Identification; IPV, Intimate Partner Violence; LOC, The Locus of Control of Behaviour; LOCS, Levels of Self Criticism Scale; LOT-R, Life Orientation Test-Revised; LSC-R, Life Stressor Checklist-Revised; MASQ, Mood and Symptoms Questionnaire; MDD, Major Depressive Disorder; MEMS, Multidimensional Existential Meaning Scale; MHC-SF, Mental Health Continuum-Short Form; MIDUS, Midlife in the United States study; MLQ, Meaning in Life Questionnaire; MPLS, Meaning and Purpose of Life Scale; MPQ, Multidimensional Personality Questionnaire; NA, Not Available; NMR, General Expectancy for Negative Mood Regulation Scale; OBVQ, Olweus Bully/Victim Questionnaire; OCD, Obsessive-Compulsive Disorder; OCPD, Obsessive-Compulsive Personality Disorder; OUD, Opioid Use Disorder; PA, physical abuse; PANAS, The Positive and Negative Affect Schedule; PD, Personality Disorder; PDS, Post-Traumatic Stress Diagnostic Scale–Part I; PECK, Personal Experiences Checklist; PLEs, Psychotic-like experiences; PMQ, Psychological Maltreatment Questionnaire; PMR, The Psychological Maltreatment Review; PMS, Pearlin Mastery Scale; PN, Physical neglect; PSI, Personal Style Inventory; PTGI, Post-traumatic Growth Inventory; PTGI-SF, Post-traumatic Growth Inventory-Short Form; PTM, Prosocial Tendencies Measure; PTSD, Post-Traumatic Stress Disorder; PVS, Personal View Survey; RBQ, Retrospective Bullying Questionnaire; RES, Resilience; RLOC, Rotter’s Locus of Control Scale; RESE, Regulatory Emotional Self-Efficacy Scale; RPS, Religious Practice Scale; RS, Resilience Scale; RSA, The Resilience Scale for Adults; RSES, Rosenberg Self-Esteem Scale; RSQ, Response Style Questionnaire; SA, Sexual abuse; SACQ, Student Adaptation to College Questionnaire; 3S, Self-Satisfaction Scale; SAS, Severity of Abuse Scale; SCC, Self-Concept Clarity Scale; SCRS, Self-Critical Rumination Scale; SCS, Self-Compassion Scale; SCSQ, The Simplified Coping Style Questionnaire; SCS-SF, The Self-Compassion Scale-Short Form; SD, Standard deviation; S-DERS, State Difficulties in Emotion Regulation Scale; SDS-R, Self-Disgust Scale Revised; SE, Self-esteem; SEQ, Sexual Events Questionnaire; SES, Socioeconomic status; SESBW, Self-Efficacy Scale for Battered Women; SES-SFV, Sexual Experiences Survey–Short Form Victimisation Revised; SHS, Subjective Happiness Scale; SLCS, Self-Liking/Self-Competence Scale; SOCS, Sense of Coherence Scale; SPRS, Short Psychological Resilience Scale; SPSI-R, The Social Problem-Solving Inventory-Revised Short Form; SRI-25, Suicide Resilience Inventory-25; SRQ, Sibling Relations Questionnaire; SSHH, Stress, Spirituality, and Health Questionnaire; STI, Sexually transmitted infection; STS, The Spiritual Transcendence Scale; SUBI, Subjective Well-being Inventory; SUD, Substance use disorder; SVCQ, Sexually Victimised Children Questionnaire; SWBS, Spiritual Well-Being Scale; SWLS, Satisfaction with Life Scale; SWS, Subjective-Well-being Scale; TADS, Trauma Distress Scale; TCAQ, The Cognitive Avoidance Questionnaire; THS, The Hope Scale; TRD, Treatment-resistant depression; TSCS, Tennessee Self-Concept Scale; TSEI, Taylor Self-Esteem Inventory; TSES, The Self-Efficacy Scale; TSPWB, The Scales of Psychological Well-Being; TSS, The Self Scale; UPPS-P, Urgency, Premeditation, Perseverance, Sensation seeking, and Positive urgency; USA, United States of America; WB, Well-being; WCQ, Ways of Coping Questionnaire; WEMWBS, Warwick-Edinburgh Mental Well-Being Scale; WHO-5, The World Health Organisation-Five Well-Being Index; WSHQ, The Wyatt Sexual History Questionnaire.

a Studies with asterisk and row marked in grey signify not included in meta-analysis but fulfilling inclusion criteria and included in the systematic review (see also a description of main results and qualitative synthesis in SA7 in the Supplement).

b Studies with a cross signify carried by same authors and involving the same sample, but assessing different outcomes and included in separated meta-analyses.

Among the 203 studies reviewed, 20 studies were only included in the systematic review. For a description and qualitative synthesis of the main results of CM and resilience domain associations that provided insufficient data for meta-analyses, see SA7 in the Supplement.

### Study quality

The mean quality rating (range = 0–8) of the included studies was 5.48 (range = 4–8). Overall, 52 (25.62%) studies were rated as ‘poor’ (NOS score = 3 or 4), 55 (27.09%) studies were rated as ‘fair’ (NOS score = 5), 45 (22.17%) studies were rated as ‘good’ (NOS score = 6), and 51 (25.12%) studies received a rating considered as ‘high’ (NOS score > 6) (see further details of the study quality assessment in ST5 in the Supplement).

### Meta-analytic results of associations between CM and resilience in adulthood

**Figure 2.**
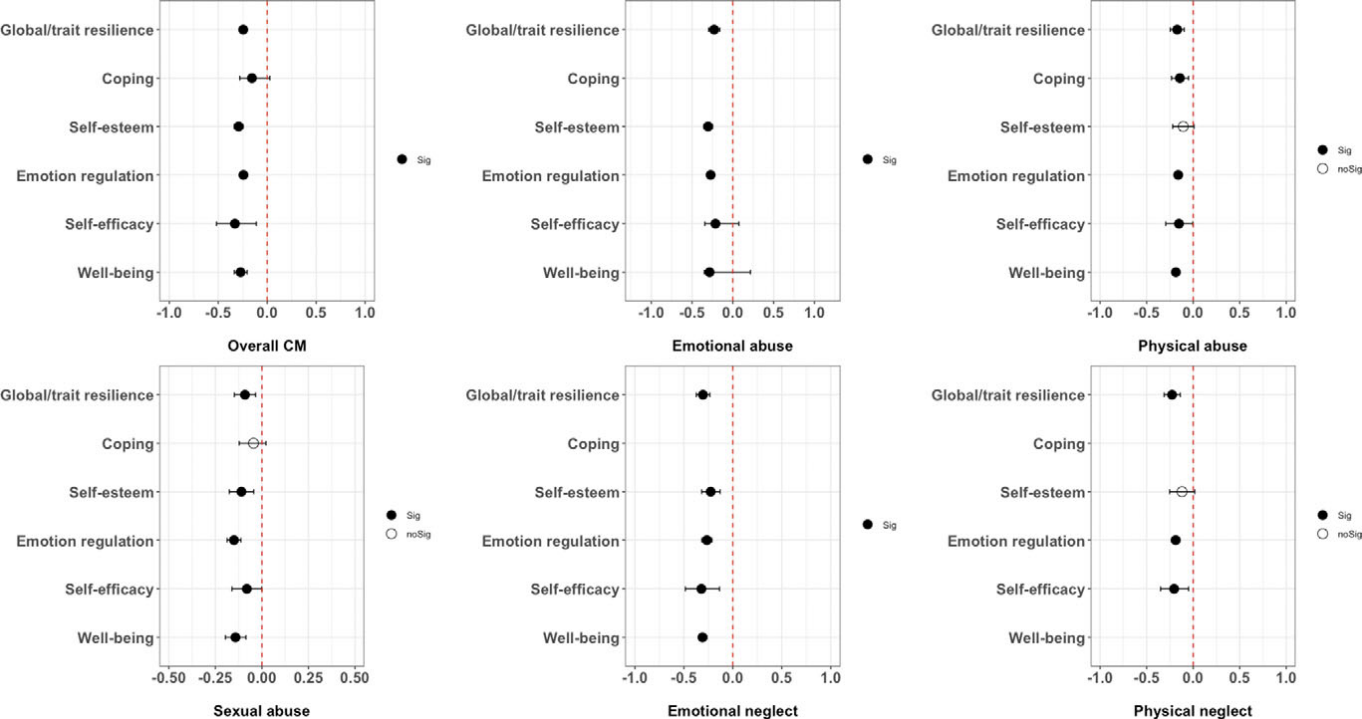
Overall results of the meta-analytic synthesis.

Separate meta-analyses with random-effects estimates were calculated to quantify associations between CM, separated by overall and subtypes, global/trait resilience (*n* = 90, *k* = 98), and five resilience domains: (1) Coping (*n* = 23, *k* = 26), (2) Self-esteem (*n* = 133, *k* = 154), (3) Emotion regulation (*n* = 192, *k* = 192), (4) Self-efficacy (*n* = 34, *k* = 34), and (5) Well-being (*n* = 52, *k* = 53). The main results are presented in Table 2 and illustrated in Figure 2. Forest plots of each analysis can be found in SF1 in the Supplement.

**Table 2.** Meta-analyses of associations between CM and resilience outcomes in adulthood

Childhood maltreatment (CM) total/subtypes	Number of studies (n), effect sizes *(k)*	Pooled sample size	Correlation coefficient			Heterogeneity				Publication bias			
			r	95% CI	*p-*value	*I^2^* (%)	Tau square *(τ2)*	Q test *p-*value	Prediction intervals	Funnel plot asymmetry	Trim & Fill imputed studies	Trim & Fill adjusted *r* coefficient (95% CI)	Egger test *p*-value
	**Global/trait resilience**												
Overall CM	25 (*28*) *	22373	−0.245	−0.282; −0.208	<0.001	86	0.008	<0.001	−0.411; −0.063	Right	5	−0.214 [−0.253; −0.174]	0.316
Emotional abuse	15 (*16*) *	5642	−0.229	−0.296; −0.160	<0.001	85	0.016	<0.001	−0.341; 0.109	Right	3	−0.175 [−0.251; −0.098]	0.258
Physical abuse	14 (*15*) *	5322	−0.172	−0.246; −0.097	<0.001	86	0.018	<0.001	−0.442; 0.126	Right	4	−0.094 [−0.178; −0.009]	0.122
Sexual abuse	13 (*14*) *	5022	−0.091	−0.148; −0.034	0.002	72	0.007	<0.001	–	Right	3	−0.050 [−0.114; −0.014]	0.186
Emotional neglect	12 (*13*) *	4665	−0.305	−0.373; −0.235	<0.001	83	0.014	<0.001	−0.532; 0.038	Right	4	−0.259 [−0.326; −0.189]	0.275
Physical neglect	11 (*12*) *	4572	−0.227	−0.312; −0.139	<0.001	88	0.021	<0.001	−0.514; 0.016	Right	5	−0.113 [−0.213; −0.012]	0.106
	**Resilience domains**												
	**Coping**												
Overall CM	9 (*10*) *	30043	−0.156	−0.280; 0.027	0.018	97	0.037	<0.001	−0.555; 0.301	–	0	–	0.616
Physical abuse	6 (*7*) *	26697	−0.143	−0.233; −0.050	0.003	82	0.009	<0.001	−0.395; 0.130	–	–	–	–
Sexual abuse	8 (*9*) *	27805	−0.045	−0.122; 0.022	0.188	78	0.006	0.076	−0.238; 0.151	–	–	–	–
	**Self-esteem**												
Overall CM	24 (*28*) *	12943	−0.292	−0.338; −0.245	<0.001	89	0.014	<0.001	−0.500; −0.053	Left	10	−0.375 [−0.420; −0.329]	0.005
Emotional abuse	21 (*24*) *	13196	−0.303	−0.357; −0.247	<0.001	91	0.019	<0.001	−0.544; −0.016	Left	5	−0.363 [−0.420; −0.304]	0.107
Physical abuse	24 (*27*) *	13799	−0.107	−0.220; 0.009	0.070	98	0.089	<0.001	−0.626; 0.477	–	0	–	0.385
Sexual abuse	24 (*29*) *	13001	−0.110	−0.175; −0.044	<0.001	92	0.030	<0.001	−0.430; 0.234	Left	12	−0.222 [−0.292; 0.149]	0.093
Emotional neglect	19 (*21*) *	11504	−0.226	−0.318; −0.130	<0.001	96	0.049	<0.001	−0.609; 0.242	Right	7	−0.113 [−0.211; −0.013]	0.109
Physical neglect	15 (*17*) *	10016	−0.120	−0.253; 0.017	0.086	98	0.082	<0.001	−0.630; 0.463	Left	7	−0.283 [−0.418; 0.135]	0.104
Bullying	6 (*8*) *	2396	−0.232	−0.313; −0.148	<0.001	68	0.009	0.002	−0.455; 0.018	–	–	–	–
	**Emotion regulation**												
Overall CM	37 (*37*)	15321	−0.243	−0.279; −0.205	<0.001	85	0.011	<.001	−0.433; 0.031	Right	4	−0.219 [−0.257; −0.179]	0.321
Emotional abuse	38 (*38*)	22561	−0.272	−0.313; −0.231	<0.001	90	0.016	<0.001	−0.494; −0.017	Right	3	−0.257 [−0.298; −0.216]	0.817
Physical abuse	33 (*33*)	19558	−0.161	−0.197; −0.125	<0.001	84	0.009	<0.001	−0.334; 0.033	Right	1	−0.154 [−0.191; −0.117]	0.393
Sexual abuse	38 (*38*)	20103	−0.150	−0.187; −0.113	<0.001	84	0.011	<0.001	−0.348; 0.061	Left	11	−0.202 [−0.242; −0.162]	0.959
Emotional neglect	24 (*24*)	11580	−0.272	−0.327; −0.218	<0.001	89	0.017	<0.001	−0.503; 0.004	Right	6	−0.214 [−0.271; −0.157]	0.036
Physical neglect	22 (*22*)	11870	−0.188	−0.232; −0.143	<0.001	83	0.010	<0.001	−0.379; 0.019	Right	1	−0.175 [−0.223; −0.127]	0.181
	**Self-efficacy**												
Overall CM	7 (*7*)	5446	−0.330	−0.518; −0.111	0.004	99	0.095	<0.001	−0.831; 0.468	–	–	–	–
Emotional abuse	6 (*6*)	3640	−0.213	−0.343; 0.074	<0.001	94	0.029	<0.001	−0.623; 0.290	–	–	–	–
Physical abuse	6 (*6*)	3640	−0.153	−0.295; −0.004	0.044	95	0.033	<0.001	−0.604; 0.372	–	–	–	–
Sexual abuse	5 (*5*)	3124	−0.081	−0.161; −0.001	0.048	77	0.006	<0.001	−0.349; 0.199	–	–	–	–
Emotional neglect	5 (*5*)	3124	−0.321	−0.485; −0.136	0.001	96	0.048	<0.001	−0.800; 0.406	–	–	–	–
Physical neglect	5 (*5*)	3124	−0.205	−0.350; −0.050	0.010	94	0.030	<0.001	−0.673; 0.381	–	–	–	–
	**Well-being**												
Overall CM	20 (*21*)	13691	−0.272	−0.336; −0.205	<0.001	93	0.024	<0.001	−0.546; 0.055	Right	10	−0.146 [−0.223; 0.066]	0.121
Emotional abuse	11 (*11*)	5712	−0.285	−0.350; 0.216	<0.001	86	0.013	<0.001	−0.508; −0.025	Right	2	−0.249 [−0.318; −0.1778]	0.196
Physical abuse	6 (*6*)	3944	−0.186	−0.216; −0.155	<0.001	0	0.000	–	–	–	–	–	–
Sexual abuse	9 (*9*)	6141	−0.142	−0.196; −0.086	<0.001	78	0.005	<0.001	−0.319; 0.045	–	–	–	–
Emotional neglect	6 (*6*)	2930	−0.310	−0.335; −0.264	<0.001	40	0.002	0.139	−0.422; −0.189	–	–	–	–

*Note:* *Different populations from the same study were included in meta-analysis; statistical significance *p* < 0.05.

#### Global/trait resilience

Overall CM and all subtypes were negatively associated with global/trait resilience (*r* = −0.091 to −0.305; *p* = .002 to <.001). Emotional neglect showed the largest magnitude of effect (*n* = 12, *k* = 13, *r* = −0.305, *p* < .001).

#### Resilience domains

##### Coping

Overall CM (*n* = 9, *k* = 10; *r* = −0.156, *p* = .018) and physical abuse (*n* = 6, *k* = 7; *r* = −0.143, *p* = .003) were negatively associated with coping but unrelated to sexual abuse.

##### Self-esteem

Overall CM and most subtypes were negatively associated with self-esteem (*r* = −0.110 to −0.303, *p* < .001), except for physical abuse and physical neglect. Emotional abuse showed the largest magnitude of effect (*n* = 21, *k* = 24; *r* = −0.303, *p* < .001).

##### Emotion regulation

Overall CM and all subtypes were negatively associated with emotion regulation (*r* = −0.150 to −0.272, *p* < .001). Emotional abuse (*n* = 38, *k* = 38; *r* = −0.272, *p* < .001) and emotional neglect showed the largest magnitude of effect (*n* = 24, *k* = 24; *r* = −0.272, *p* < .001).

##### Self-efficacy

Overall CM and all subtypes were negatively associated with self-efficacy (*r* = −0.081 to −0.330, *p =* 0.048 to < .001). Emotional neglect showed the largest magnitude of effect (*n* = 5, *k* = 5; *r* = −0.321, *p* < .001).

##### Well-being

Overall CM and all subtypes were negatively associated with well-being (*r* = −0.142 to −0.310, *p* < .001). Emotional neglect showed the largest magnitude of effect (*n* = 6, *k* = 6; *r* = −0.310, *p* < .001).

### Heterogeneity, meta-regressions

Of the 33 meta-analyses completed, heterogeneity was high for most results (see results on heterogeneity in Table 2).

Meta-regressions were conducted by overall CM and CM subtypes. The following continuous variables were explored: (1) mean age; (2) proportion of males; (3) sample size; and (4) study quality (NOS score).

#### Global/ trait resilience

The magnitude of the association between sexual abuse and global/trait resilience decreased with sample size (*n* = 12, *k* = 12, *B* = −0.000, 95% CI [−0.021; 0.002], *p* = 0.018) and increased with study quality (*n* = 12, *k* = 12, *B* = 0.161, 95% CI [0.073; 0.249], *p* < 0.001).

#### Resilience domains

*Coping:* the magnitude of the association between overall CM and coping increased with sample size (*n* = 7, *k* = 7, *B* = 0.001, 95% CI [0.000; 0.001], *p* < 0.001) and decreased with age (*n* = 7, *k* = 7, *B* = −0.001, 95% CI [−0.000; −0.000], *p* = 0.003) and study quality (*n* = 7, *k* = 7, *B* = −0.091, 95% CI [−0.164; −0.018], *p* = 0.014). The association between physical abuse and coping decreased with age (*n* = 7, *k* = 7, *B* = −0.000, 95% CI [−0.000; −0.000], *p* = 0.002).

*Emotion regulation:* the association between sexual abuse and emotion regulation decreased with study quality (*n* = 33, *k* = 33, *B* = −0.034, 95% CI [−0.063; 0.005], *p* = 0.021). The association between emotional neglect and emotion regulation increased with age (*n* = 20, *k* = 20, *B* = 0.014, 95% CI [0.005; −0.022], *p* = 0.002) and sample size (*n* = 20, *k* = 20, *B* = −0.000, 95% CI [−0.000; −0.000], *p* = 0.003). The association between physical neglect and emotion regulation increased with age (*n* = 6, *k* = 6, *B* = 0.010, 95% CI [0.000; −0.095], *p* = 0.040).

No moderation effects of mean age, percentage of males, sample size, or study quality were found for the associations between overall or any subtype of CM and self-esteem, self-efficacy, or well-being. For a detailed description of meta-regression results see SF2 in the Supplement.

### Subgroup analyses

Subgroup analyses were conducted by overall CM and subtypes. The following categorical variables were explored: (1) western *versus* non-western countries; (2) clinical *versus* non-clinical samples.

#### Global/trait resilience

No differences were found for the associations between overall or any subtype of CM and global/trait resilience in western *versus* non-western countries, or in clinical *versus* non-clinical samples.

#### Resilience domains

The association between emotional abuse and emotion regulation was stronger in western (*n* = 21, *r* = −0.321, [−0.364; −0.277]) *versus* non-western countries (*n* = 16, *r* = −0.215, [−0.282; −0.1545), *p* = 0.010 (see Figure a in the Supplement). The association between emotional abuse and self-esteem was weaker in western (*n* = 9, *r* = −0.213, [−0.321; −0.098]) *versus* non-western countries (*n* = 15, *r* = −0.352, [−0.407; −0.296]), *p* = 0.025 (see Figure b in the Supplement).

No differences were found for the associations between overall or any subtype of CM and any resilience domains in clinical *versus* non-clinical samples. For a detailed description of subgroup analyses results see SF3 (Figures a, b) in the Supplement.

### Sensitivity analysis

To further assess possible causes of heterogeneity and the robustness of findings, a one-study-removed sensitivity analysis (Borenstein, 2022a) was conducted. Removal of single effect sizes did not change the patterns of results with a few exceptions (see SF4 in the Supplement).

### Publication bias

The visual inspection of the funnel plots (see SF5 in the Supplement) and Egger’s test suggested publication bias for the associations between overall CM and self-esteem (*z* = −0.375, *p* = 0.005), and between emotional neglect and emotion regulation (*z* = −0.214, *p* = 0.036). The trim-and-fill corrected random-effect estimate changed relative to the uncorrected estimate, yet both associations remained significant (see Table 2).

### Narrative synthesis of moderators and mediators reported in the included studies

Three (Arslan & Genç, 2022; Shen & Soloski, 2024; Somers, Ibrahim, & Luecken, 2017) of the 203 reviewed studies investigated effect moderation, and 17 studies investigated effect mediation between CM and resilience outcomes.

#### Moderators

One study found that heart rate reactivity moderated the effects of CM on depressive symptoms and positive affect (well-being) in young adults (Somers et al., 2017).

Another study in college students found that positive perception moderated the adverse impact of emotional maltreatment on emotional but not social well-being (Arslan & Genç, 2022).

Childhood attachment significantly predicted adult attachment, psychological distress, and self-esteem in adulthood and moderated the relation between child sexual abuse and anxious adult attachment. In addition, secure attachment at least partially protected against a negative long-term effects of child sexual abuse and fostered intra- and interpersonal adjustment in survivors (Shen & Soloski, 2024).

#### Mediators

Two studies found that intrapersonal strength (Kapoor et al., 2018) and perceived burdensomeness (Allbaugh et al., 2017) mediated the relationship between CM and suicide resilience, especially in African American females. Another study found that resilience and coping strategies mediated the association between childhood abuse and PTSD severity and that lower resilience and dysfunctional coping strategies may accentuate the detrimental effects of childhood abuse on PTSD (Kim et al., 2021).

A study found that negative religious coping related positively to all forms of CM other than emotional neglect, while positive religious coping related negatively only to child physical neglect. Furthermore, PTSD symptoms acted as a mediator between abuse and negative religious coping among low-income, African American women with a history of intimate partner violence and suicidal behaviours (Bradley, Schwartz, & Kaslow, 2005).

Two studies found that parental and peer relationship quality mediated the relationship between dual violence exposure to interparental violence and child physical maltreatment and self-esteem in young adulthood (Shen, 2009), while authenticity in close relationships partially mediated the relation between emotional maltreatment and negative self-esteem in college women (Theran & Han, 2013).

In a cross-national investigation, perceived negative (but not positive) impact of bullying mediated the relationship between adolescent bullying and self-esteem. In addition, perceived negative impact of adolescent bullying victimisation partially mediated, while perceived negative impact of adolescent bullying victimisation fully mediated the relationship between bullying and life satisfaction (Pabian, Dehue, Völlink, & Vandebosch, 2022).

One study found that disorganised attachment, including fear, distrust, and suspicion of attachment figures, as well as odd and disoriented behaviours, mediated the relationship between CM and difficulties in emotion dysregulation above what is captured by anxious and avoidant attachment in emerging adulthood in the context of emerging adult romantic relationships (Whittington, 2024).

In a serial mediation model, one study found that anxiety and emotional dysregulation mediated the effect of childhood emotional abuse on pain resilience among individuals with alcohol use disorder (Zaorska et al., 2020).

Self-concept was shown to mediate the relationship between specific forms of CM and abstinence motivation, and self-concept mediated the relationship between CM and abstinence motivation, as well as self-efficacy among drug addicts (Lu, Wen, Deng, & Tang, 2017).

Self-compassion mediated and mitigated the association between CM severity and later emotion regulation difficulties in individuals with substance use (Vettese, Dyer, Li, & Wekerle, 2011). Another study concluded that self-compassion, while not a full mediator between CM and psychological well-being, served as a partial mediator for male survivors of CM (Tarber et al., 2016). In contrast, researchers using serial mediation analysis found that self-critical rumination was a partial mediator, and self-compassion was not a mediator in the relationship between child emotional maltreatment, and self-satisfaction and well-being (Cecen & Gümüş, 2024).

Another study found that emotional maltreatment was negatively associated with life satisfaction through self-esteem and through the pathway from self-esteem to self-compassion, suggesting that self-processes are more vulnerable to emotional maltreatment than to other maltreatment types in emerging adulthood (Wu et al., 2022).

In a chain mediation model, positive affect, negative affect, and emotional intelligence mediated the link between CM and life satisfaction. In addition, CM influenced life satisfaction through the sequential intermediary of ‘emotional intelligence-positive affect’ and ‘emotional intelligence-negative affect’ (Xiang, Yuan, & Zhao, 2021). Another study, using a two-step structural equation modelling approach, found an association between childhood psychological maltreatment and spiritual well-being, and that this relationship is mediated by both intolerance of uncertainty and emotion regulation in a Turkish sample (Yilmaz & Satici, 2024).

Finally, in a prospective cohort study, although adolescent bullying was a significant risk factor for the onset of depression and poor well-being in adulthood, no mediating or moderating effects of depression were found on the relationship between bullying and well-being (Armitage et al., 2021).

## Discussion

This systematic review and meta-analysis investigated associations between overall and different subtypes of CM, global/trait resilience, and domains of resilience in adults. Across the identified studies, we confirmed overall CM was associated with resilience in adulthood. Specifically, overall CM was associated with poorer global/trait resilience, coping, self-esteem, emotion regulation, self-efficacy, and well-being. We also found associations between different CM subtypes and impairment in both global/trait resilience and most resilience domains. However, overall associations were small in magnitude, and findings differed depending on the subtype of CM and resilience domain considered, suggesting differential and specific effects.

Given the vast evidence that CM increases the likelihood of developing physical and mental health problems (Baldwin et al., 2023; Mehta et al., 2023) and that resilience deficits are a core component of adaptive functioning (Barton et al., 2023), it is possible that a larger effect is being constrained by methodological limitations in the literature. It should also be considered that some of the significant results found in this review may be affected by confounding variables not addressed by most of the included studies (e.g. education level, intelligence, socioeconomic status) and that there could be other, non-causal explanations, such as poverty that may increase risk of CM exposure and impairment in resilience outcomes. Future prospective studies should examine whether a bidirectional relationship between CM and resilient functioning exists.

The associations with CM found in this meta-analysis were weak, suggesting that impairments in resilience in adults are likely influenced by additional biological factors, such as brain structure and functions (Fares-Otero, Verdolini et al., 2024). Future research should explore how the timing of CM (Fares-Otero & Schalinski, 2024), especially during sensitive neurodevelopmental periods affects resilience, and preferably employ multimodal approaches, including neuroimaging and clinical assessments (Demers et al., 2022; Fares-Otero, Halligan, Vieta, & Heilbronner, 2024) to capture the role of neurobiological factors (Ioannidis, Askelund, Kievit, & van Harmelen, 2020; Zhang, Rakesh, Cropley, & Whittle, 2023) and psychosocial influences, such as cognitive reserve (Fares-Otero Borràs et al., 2024). Despite the relevance of CM in health (Lawrence et al., 2023; Telfar et al., 2023), studies examining its effects on resilience outcomes are limited, particularly in those with mental and physical conditions. Further research on the role of CM exposure, especially neglect, on resilience outcomes, including coping abilities, and in larger male samples (Davis et al., 2018; Fares-Otero et al., 2025), is crucial to inform interventions and improve outcomes in adulthood. See also Table 3 for a summary of methodological issues and further recommendations for future studies.

**Table 3.** Methodological problems identified in the included studies and recommendations for future research

Methodological problem	Recommendation
Inconsistencies in the measurement of CM and lack of studies assessing domestic violence or bullying exposure	Studies should report both total score and subscale scores for CM types. Studies should include instruments to assess primary/secondary school bullying(Olweus, 2012), and parental discord/fights or intrafamilial abuse (Bifulco, Bernazzani, Moran, & Jacobs, 2005).
Lack of studies measuring severity and timing of CM exposure	Studies should consider the MSQ (Calheiros, Silva, & Magalhães, 2021) and MACE (Teicher & Parigger, 2015).
Inconsistencies in measurement of well-being and lack of studies assessing coping	Use standardised assessment tools across studies, including objective and subjective approaches for well-being (VanderWeele et al., 2020), and the COPE inventory (Carver, Scheier, & Weintraub, 1989).
Cross-sectional design, which does not allow for causal inference	Longitudinal cohort studies with early life recruitment, where possible. Pooling of longitudinal cohort studies through international collaborations that include researchers from currently underrepresented regions (e.g. Africa, Latin America).
Analyses of multiple outcomes and low statistical power	Use adequately powered sample sizes. Correct for multiple outcomes to avoid type 1 errors.
Effects of other stressful events and traumatic experiences in adulthood not considered	Include a measure of adult-onset trauma such as the ITQ (Cloitre et al., 2018).
Inconsistencies in screening for mental disorders	Screen for psychiatric comorbidities with a brief measure, e.g. the MINI (Sheehan et al., 1998). Consider including PTSD in analyses (Fares-Otero & Seedat, 2024).
Lack of comprehensive reporting of sociodemographic and clinical characteristics	Report gender, SES, education level, social support, physical health conditions.
Lack of studies assessing potential moderators between CM and resilience outcomes	Include moderation/mediation analyses on the association between CM and resilience, involving sex/gender, brain functioning (Fares-Otero, Verdolini et al., 2024), personality traits, social support (Fares-Otero, Sharp, et al., 2024), education level, and SES.

Abbreviations: CM, Childhood Maltreatment; COPE, Coping Orientation to Problems Experienced; ITQ, International Trauma Questionnaire; MACE, Maltreatment and Abuse Chronology of Exposure; MINI, Mini-International Neuropsychiatric Interview; MSQ, Child Maltreatment Severity Questionnaire; PTSD, posttraumatic stress disorder; SES, Socioeconomic status.

Interestingly, the emotional types of CM showed the strongest associations with impaired resilience. This is in line with previous meta-analysis on CM and social functioning (Fares-Otero De Prisco et al., 2023) and a substantial body of evidence demonstrating that emotional maltreatment may be more strongly associated with high levels of affective instability (Palmier-Claus et al., 2025) and depressive symptoms (Hutson et al., 2024), factors that may mediate the relationship between CM and resilience outcomes. Taken together, our findings indicate that emotional abuse and emotional neglect represent an important potential (early) intervention target for adults.

### Clinical implications

Clinically, our findings of poorer resilience in people with CM histories align with and inform a growing body of research suggesting that CM should be routinely considered during assessment, diagnosis, and treatment. Assessing CM and resilience systematically in clinical and community settings could support early intervention, mitigate detrimental effects on resilience, and may even contribute to more accurate diagnoses. While some institutions already include CM in standard assessments, broader adoption of this practice across mental health settings would strengthen preventive and supportive care, particularly by addressing impairment in CM-related resilience early in the illness.

Our findings suggest that early interventions promoting resilience, such as trauma-focused cognitive behavioural therapy-based resilience training (Zalta et al., 2016), therapeutic processes that encourage social ties and therapeutic alliance (Burton, Cooper, Feeny, & Zoellner, 2015; Snijders et al., 2018), and psychotherapy founded on the Trauma Resiliency Model (Grabbe & Miller-Karas, 2018) might be useful in helping adults with CM experience by focusing on maintaining global and functional health. Moreover, psychotherapeutic approaches should target self-compassion and self-concept, secure attachment, emotional intelligence, PTSD and mood symptoms, and advance training to help individuals to cope with life stressors that may be preventing them from achieving or maintaining recovery.

### Strengths and limitations

This study builds on the well-established evidence base for the role of CM as a risk factor for adverse health and psychosocial outcomes and reinforces that experiences of CM could be related to impaired resilience in survivors. We performed a comprehensive and up-to-date systematic review, allowing the inclusion of a large number of studies. This is by far the first meta-analysis in the field of CM and resilience with a multi-domain approach. This study also benefitted from the wide range of pooled subjects, which constitutes a geographically diverse sample. Although there was some variability in which subtypes of CM were reported, most studies used the same standard and validated instrument to assess CM (CTQ). Other strengths of this study include the rigorous methodology of the systematic search, study selection, and data extraction performed by independent researchers.

Our work also includes some limitations. First, the number of studies available for some meta-analysis was small, meaning that analyses may not have been sufficiently powered for detecting small effects (Jackson & Turner, 2017). The capacity to identify heterogeneity and moderators was also substantially limited, and extra caution is needed for conclusions in meta-regressions when there are <10 studies. Second, it was impossible to account for all the possible variations across populations with different social environments, health conditions, and diagnoses, as well as variations across measurement instruments utilised (and conditions of administration) in the included studies, although most assessed resilience outcomes with robust tools. A sensitivity analysis confirmed that omitting one study at a time did not change the overall findings. Third, CM was retrospectively reported through assessments that may be biased, though retrospective self-reports of CM have shown sufficient reliability (Badenes-Ribera, Georgieva, Tomás, & Navarro-Pérez, 2024). Finally, we did not include unpublished work. However, the inclusion of data from unpublished studies could also introduce bias (Boutron et al., 2023).

## Conclusions

In conclusion, overall CM and its subtypes are linked to lower global/trait resilience and more resilience impairments across several domains, particularly coping, self-esteem, emotion regulation, self-efficacy, and well-being in adulthood. While the associations are weak, exploring socioeconomic status, education level, and the timing and severity of CM, as well as moderators such as attachment, mood symptoms, and personality features, may clarify these relationships. This knowledge may reduce the burden associated with negative health and psychosocial consequences in adulthood and increase the likelihood that maltreated individuals receive appropriate and/or optimal treatment.

Prospective and interventional studies are needed to address the limitations of the current evidence, which mainly comprises cross-sectional studies with retrospective reporting of CM. Our findings nonetheless support CM as a key predictor of resilient functioning in adulthood, underscoring the potential value of trauma-informed interventions and approaches founded on trauma resiliency models. Also, early interventions for at-risk children and adolescents may help improve resilience and quality of life outcomes long-term, including those with mental disorders.

## Supporting information

Fares-Otero et al. supplementary material Fares-Otero et al. supplementary material

## Data Availability

NEF-O has full access to all data in the study and takes responsibility for the integrity of the data and the accuracy of the data analyses. The data that support the findings of this study and/or codes are available on request.

## Supplementary material

The supplementary material for this article can be found at http://doi.org/10.1017/S0033291725001205.
