# Supplementary material for: Child maltreatment and resilience in adulthood: a systematic review and meta-analysis
Source: Psychol Med. 2025 Jun 2;55:e163. doi: 10.1017/S0033291725001205 (PMC12150341; doi:10.1017/S0033291725001205)
Supplement: Fares-Otero et al. supplementary material [file S0033291725001205sup001.docx]

Supplement

Child maltreatment and resilience in adulthood:

A systematic review and meta-analysis

[SA1. Glossary of terms 1](#_Toc190038103)

[SA2. Literature search 5](#_Toc190038104)

[SA3. Extracted variables 9](#_Toc190038105)

[SA4. Definition of exposure and outcome variables 10](#_Toc190038106)

[SA5. Full list of included studies 12](#_Toc190038107)

[SA6. Full list of excluded studies with reasons 29](#_Toc190038108)

[SA7. Qualitative synthesis 77](#_Toc190038109)

[ST1. PRISMA 2020 statement and checklist 85](#_Toc190038110)

[ST2. PRISMA 2020 for abstracts checklist 89](#_Toc190038111)

[ST3. MOOSE guidelines and checklist 90](#_Toc190038112)

[ST4. Description and measurement of global/trait resilience and domains 93](#_Toc190038113)

[ST5. Quality assessment of included studies 108](#_Toc190038114)

[**Newcastle-Ottawa scale (NOS) for non-randomised studies** 109](#_Toc190038115)

[**Results of the quality assessment of included studies** 111](#_Toc190038116)

[SF1. Forest plots investigating associations between CM and resilience in adulthood 118](#_Toc190038117)

[SF2. Meta-regressions for associations between CM and resilience in adulthood 151](#_Toc190038118)

[SF3. Subgroup analyses for associations between CM and resilience in adulthood 156](#_Toc190038119)

[SF4. One-study-removed sensitivity analyses 159](#_Toc190038120)

[SF5. Funnel plots for associations between CM and resilience in adulthood 161](#_Toc190038121)

[References 172](#_Toc190038122)

# SA1. Glossary of terms

AAQ-II: The Acceptance and Action Questionnaire-II

ABS: The Affect Balance Scale

ACE: Adverse Childhood Exposure

ACE-Q: Adverse Childhood Experiences Questionnaire

ADHD: Attention-Deficit/Hyperactivity Disorder

ALSPAC: Avon Longitudinal Study of Parents and Children

AMS: Academic Motivation Scale

AN: Anorexia Nervosa

AnxNOS: Anxiety Disorder Not Otherwise Specified

ARM: Adult Resilience Measure

ASD: Acute Stress Disorder

ATQ: Adult Temperament Questionnaire-Short Form

AUD: Alcohol Use Disorder

BD: Bipolar Disorder

BERQ: Behavioural Emotion Regulation Questionnaire

BFIS-9: Bullying and friendship interview schedule-9

BPD: Borderline Personality Disorder

BPNSS: Basic Psychological Needs Scale

Brief-COPE: The Brief Coping Orientation to Problems Experienced Inventory

Brief RCOPE: The Brief Religious Coping Activities Scale

BRS: The Brief Resilience Scale

BSCS: The Brief Self-Control Scale

BSE: The Beck Self-Esteem Scale

BSI: Brief Symptom Inventory

CAP: Child Abuse Potential Inventory

CAQ: Childhood Abuse Questionnaire

CAs: College Adjustment Scale

CAS: Childhood Abuse Scale

CASRS: The Child Abuse and Self Report Scale

CATS: The Child Abuse and Trauma Scale

CCHS-MH: Canadian Community Health Survey-Mental Health

CCMS: Comprehensive Child Maltreatment Scale

CD-RISC: The Connor–Davidson Resilience Scale

CECA-Q: Childhood Experiences of Care and Abuse Questionnaire

CEDV: Child Exposure to Domestic Violence

CERQ: Cognitive Emotion Regulation Questionnaire

CERQ-Short: The Cognitive Emotion Regulation Questionnaire-short version

CEVQ: Childhood Experiences of Violence Questionnaire

CFSEI-2: Culture-Free Self-Esteem Inventory

CIDI: Composite International Diagnostic Interview

CISS: Coping Inventory for Stressful Situation

CM: Childhood Maltreatment

CMIS: Childhood Maltreatment Interview Schedule

CMIS-SF: Child Maltreatment Interview Schedule—Short Form

COPE: Coping Orientations to the Problems Experienced

CSA: Child sexual abuse

CSAQ: Childhood Sexual Abuse Questionnaire

CSEI: Coopersmith Self-Esteem Inventory

CSI: Coping Strategies Inventory

CSI-SF: Coping Strategies Inventory–Short Form

CT: Childhood Trauma

CTI: Childhood Trauma Interview

CTQ: Childhood Trauma Questionnaire

CTQ-SF: Childhood Trauma Questionnaire-Short Form

CTs: Conflict Tactics Scale

CTS: Childhood Trauma Screener

CTS-33: Childhood Trauma Scale-33

CTs Form-R: The Conflict Tactics Scales Form R

CTs-PC: Parent-Child Conflict Tactics Scales

CW: The Coping Wheel

DD-NOS: Depressive Disorder Not Otherwise Specified

DEQ-SC: Depressive Experiences Questionnaire Self-Criticism

DERS: The Difficulties in Emotion Regulation Scale

DERS-SF: Difficulties in Emotion Regulation Scale – Short Form

DSM: Diagnostic and Statistical Manual of Mental Disorders

DSQ: The Defense Style Questionnaire

DTS: Distress Tolerance Scale

DUKE: The Duke Health Profile

DV: Domestic Violence

EA: Emotional Abuse

EAIA: The Child Abuse Scale for Adults

ED: Eating disorders

EDS: Emotional Dysregulation Scale

EN: Emotional Neglect

ERDS: Emotion Regulation Difficulty Scale-Short Form

ER: Emotion Regulation

ERPS: The Emotion Regulation Process Scale

ERQ: Emotional Regulation Questionnaire

ERQ-CA: Emotion Regulation Questionnaire modified version

ERS: The Emotion Regulation Scale

ETISR-SF: Early Trauma Inventory Self-Report - Short Form

FAM: Feelings and Me Questionnaire

FCVQ: Finkelhor Childhood Victimisation Questionnaire

FFMQ: The Five Facet Mindfulness Questionnaire

FSHQ: Family and Sexual History Questionnaire

GAD: Generalised Anxiety Disorder

GHQ: General Health Questionnaire

GSAD: Generalised Social Anxiety Disorder

GSES: General Self-Efficacy Scale

HFS: The Heartland Forgiveness Scale

HIV: Human Immunodeficiency Virus

HOPES: Hunter Opinions and Personal Expectations Scale

IBS: Impulsive Behaviour Scale

ICAST-R: The ISPCAN Child Abuse Screening Tools Retrospective version

ICD: International Statistical Classification of Diseases and Related Health Problems

ICES: Invalidating Childhood Environments Scale

IPV: Intimate Partner Violence

LOC: The Locus of Control of Behaviour

LOCS: Levels of Self Criticism Scale

LOT-R: Life Orientation Test-Revised

LSC-R: Life Stressor Checklist-Revised

MASQ: Mood and Symptoms Questionnaire

MDD: Major Depressive Disorder

MEMS: Multidimensional Existential Meaning Scale

MHC-SF: Mental Health Continuum-Short Form

MIDUS: Midlife in the United States study

MLQ: Meaning in Life Questionnaire

MOOSE: Meta-analysis Of Observational Studies in Epidemiology

MPLS: Meaning and Purpose of Life Scale

MPQ: Multidimensional Personality Questionnaire

NA: Not Available

NMR: General Expectancy for Negative Mood Regulation Scale

NS: Not Significant

OBVQ: Olweus Bully/Victim Questionnaire

OCD: Obsessive-Compulsive Disorder

OCPD: Obsessive-Compulsive Personality Disorder

OUD: Opioid Use Disorder

PANAS: The Positive and Negative Affect Schedule

PA: Physical Abuse

PD: Personality Disorder

PDS: Post-Traumatic Stress Diagnostic Scale–Part I

PECK: The Personal Experiences Checklist

PLEs: Psychotic-like experiences

PMQ: Psychological Maltreatment Questionnaire

PMR: The Psychological Maltreatment Review

PMS: Pearlin Mastery Scale

PN: Physical Neglect

PRISMA: Preferred Reporting Items for Systematic Reviews and Meta-Analyses

PSI: Personal Style Inventory

PTGI: Post-traumatic Growth Inventory

PTGI-SF: Post-traumatic Growth Inventory-Short Form

PTM: Prosocial Tendencies Measure

PTSD: Post-Traumatic Stress Disorder

PVS: The Personal View Survey

RBQ: Retrospective Bullying Questionnaire

RES: Resilience

RESE: The Regulatory Emotional Self-Efficacy Scale

RLOC: Rotter’s Locus of Control Scale

RPS: Religious Practice Scale

RS: Resilience Scale

RSA: The Resilience Scale for Adults

RSES: Rosenberg Self-Esteem Scale

RSQ: Response Style Questionnaire

3S: Self-Satisfaction Scale

SA: Sexual Abuse

SACQ: Student Adaptation to College Questionnaire

SAS: Severity of Abuse Scale

SCC: Self-Concept Clarity Scale

SCRS: Self-Critical Rumination Scale

SCS: Self-Compassion Scale

SCSQ: The Simplified Coping Style Questionnaire

SCS-SF: The Self-Compassion Scale Short Form

SD: Standard Deviation

S-DERS: State Difficulties in Emotion Regulation Scale

SDS-R: Self-Disgust Scale Revised

SE: Self-esteem

SEQ: Sexual Events Questionnaire

SES: Socioeconomic Status

SESBW: Self-Efficacy Scale for Battered Women

SES-SFV: The sexual Experiences Survey–Short Form Victimisation Revised

SHS: Subjective Happiness Scale

SLCS: The Self-Liking/Self-Competence Scale

SOCS: Sense of Coherence Scale

SPRS: Short Psychological Resilience Scale

SPSI-R: The Social Problem-Solving Inventory-Revised Short Form

SRI-25: Suicide Resilience Inventory-25

SRQ: Sibling Relations Questionnaire

SSHH: Stress, Spirituality, and Health Questionnaire

STI: Sexually Transmitted Infection

STS: The Spiritual Transcendence Scale

SUBI: Subjective well-being inventory

SUD: Substance Use Disorder

SVCQ: Sexually Victimised Children Questionnaire

SWBS: The Spiritual Well-Being Scale

SWLS: The Satisfaction with Life Scale

SWS: Subjective Well-being Scale

TADS: The Trauma Distress Scale

TCAQ: The Cognitive Avoidance Questionnaire

TEC: Traumatic Experience Checklist

THS: The Hope Scale

TRD: Treatment-Resistant Depression

TSCS: Tennessee Self-Concept Scale

TSEI: The Taylor Self-Esteem Inventory

TSES: The Self-Efficacy Scale

TSPWB: The Scales of Psychological Well-Being

TSS: The Self Scale

UPPS-P: Urgency, Premeditation, Perseverance, Sensation seeking, Positive urgency

USA: The United States of America

WB: Well-being

WCQ: Ways of Coping Questionnaire

WEMWBS: Warwick-Edinburgh Mental Well-Being Scale

WHO-5: The World Health Organisation-Five Well-Being Index

WSHQ: The Wyatt Sexual History Questionnaire

# SA2. Literature search

**Search string**

("resilience" OR "resilient” OR "resiliency" OR "self-regulation" OR "self-efficacy" OR "self-organisation" OR "self-reliance" OR "self-esteem" OR "self-confidence" OR "adaptive functioning" OR "adaptive coping" OR "competent coping" OR "successful coping" OR "social competence" OR "sense of mastery" OR "problem solving" OR "social adjustment" OR "motivation" OR "emotion regulation" OR "positive appraisal" OR "meaning in life" OR "sense of coherence" OR "regulatory flexibility" OR "optimism" OR "positive affect" OR "invulnerability" OR "hardiness" OR " buffering" OR "psychological well-being")

AND

("child* maltreatment" OR "child* trauma" OR "child* advers*" OR "early life adversity" OR "early life stress" OR "complex trauma" OR "child* victim*"OR "child* abuse" OR "child* neglect" OR "child* physical abuse" OR "child* emotional abuse" OR "child* psychological abuse" OR "domestic violence" OR "family violence" OR "bullying" OR "child* sexual abuse" OR "CTQ" OR "childhood trauma questionnaire" OR "CECA")

**Search strategies adapted for each database**

**PubMed/MEDLINE**

Initial search on 18.04.2023, 2534 articles retrieved, filter: humans and updated on 12.06.2024, 361 articles retrieved.

("resilience"[Title/Abstract] OR "resilient"[Title/Abstract] OR "resiliency"[Title/Abstract] OR "self-regulation"[Title/Abstract] OR "self-efficacy"[Title/Abstract] OR "self-organisation"[Title/Abstract] OR "self-reliance"[Title/Abstract] OR "self-esteem"[Title/Abstract] OR "self-confidence"[Title/Abstract] OR "adaptive functioning"[Title/Abstract] OR "adaptive coping"[Title/Abstract] OR "competent coping"[Title/Abstract] OR "successful coping"[Title/Abstract] OR "social competence"[Title/Abstract] OR "sense of mastery"[Title/Abstract] OR "problem solving"[Title/Abstract] OR "social adjustment"[Title/Abstract] OR "motivation"[Title/Abstract] OR "emotion regulation"[Title/Abstract] OR "positive appraisal"[Title/Abstract] OR "meaning in life"[Title/Abstract] OR "sense of coherence"[Title/Abstract] OR "regulatory flexibility"[Title/Abstract] OR "optimism"[Title/Abstract] OR "positive affect"[Title/Abstract] OR "invulnerability"[Title/Abstract] OR "hardiness"[Title/Abstract] OR "buffering"[Title/Abstract] OR "psychological well-being"[Title/Abstract]) AND ("child* maltreatment"[Title/Abstract] OR "child* trauma"[Title/Abstract] OR "child* advers*"[Title/Abstract] OR "early life adversity"[Title/Abstract] OR "early life stress"[Title/Abstract] OR "complex trauma"[Title/Abstract] OR "child* victim*"[Title/Abstract] OR "child* abuse"[Title/Abstract] OR "child* neglect"[Title/Abstract] OR "child* physical abuse"[Title/Abstract] OR "child* emotional abuse"[Title/Abstract] OR "child* psychological abuse"[Title/Abstract] OR "domestic violence"[Title/Abstract] OR "family violence"[Title/Abstract] OR "bullying"[Title/Abstract] OR "child* sexual abuse"[Title/Abstract] OR "CTQ"[Title/Abstract] OR "childhood trauma questionnaire"[Title/Abstract] OR "CECA"[Title/Abstract])

**Scopus**

Initial search on 18.04.2023, 3347 articles retrieved and updated on 12.06.2024, 1032 articles retrieved.

(TITLE-ABS(resilience) OR TITLE-ABS(resilient) OR TITLE-ABS(resiliency) OR TITLE-ABS("self-regulation") OR TITLE-ABS("self-efficacy") OR TITLE-ABS("self-organisation") OR TITLE-ABS("self-reliance") OR TITLE-ABS("self-esteem") OR TITLE-ABS("self-confidence") OR TITLE-ABS("adaptive functioning") OR TITLE-ABS("adaptive coping") OR TITLE-ABS("competent coping") OR TITLE-ABS("successful coping") OR TITLE-ABS("social competence") OR TITLE-ABS("sense of mastery") OR TITLE-ABS("problem solving") OR TITLE-ABS("social adjustment") OR TITLE-ABS(motivation) OR TITLE-ABS("emotion regulation") OR TITLE-ABS("positive appraisal") OR TITLE-ABS("meaning in life") OR TITLE-ABS("sense of coherence") OR TITLE-ABS("regulatory flexibility") OR TITLE-ABS(optimism) OR TITLE-ABS("positive affect") OR TITLE-ABS(invulnerability) OR TITLE-ABS(hardiness) OR TITLE-ABS(buffering) OR TITLE-ABS("psychological well-being")) AND (TITLE-ABS("child* maltreatment") OR TITLE-ABS("child* trauma") OR TITLE-ABS("child* advers*") OR TITLE-ABS("early life adversity") OR TITLE-ABS("early life stress") OR TITLE-ABS("complex trauma") OR TITLE-ABS("child* victim*") OR TITLE-ABS("child* abuse") OR TITLE-ABS("child* neglect") OR TITLE-ABS("child* physical abuse") OR TITLE-ABS("child* emotional abuse") OR TITLE-ABS("child* psychological abuse") OR TITLE-ABS("domestic violence") OR TITLE-ABS("family violence") OR TITLE-ABS(bullying) OR TITLE-ABS("child* sexual abuse") OR TITLE-ABS(CTQ) OR TITLE-ABS("childhood trauma questionnaire") OR TITLE-ABS(CECA))

**WoS (Web of Science Core Collection)**

Initial search on 18.04.2023, 787 articles retrieved and updated on 12.06.2024, 804 articles retrieved.

((TI=resilience OR AB=resilience) OR (TI=resilient OR AB=resilient) OR (TI=resiliency OR AB=resiliency) OR (TI="self-regulation" OR AB="self-regulation") OR (TI="self-efficacy" OR AB="self-efficacy") OR (TI="self-organisation" OR AB="self-organisation") OR (TI="self-reliance" OR AB="self-reliance") OR (TI="self-esteem" OR AB="self-esteem") OR (TI="self-confidence" OR AB="self-confidence") OR (TI="adaptive functioning" OR AB="adaptive functioning") OR (TI="adaptive coping" OR AB="adaptive coping") OR (TI="competent coping" OR AB="competent coping") OR (TI="successful coping" OR AB="successful coping") OR (TI="social competence" OR AB="social competence") OR (TI="sense of mastery" OR AB="sense of mastery") OR (TI="problem solving" OR AB="problem solving") OR (TI="social adjustment" OR AB="social adjustment") OR (TI=motivation OR AB=motivation) OR (TI="emotion regulation" OR AB="emotion regulation") OR (TI="positive appraisal" OR AB="positive appraisal") OR (TI="meaning in life" OR AB="meaning in life") OR (TI="sense of coherence" OR AB="sense of coherence") OR (TI="regulatory flexibility" OR AB="regulatory flexibility") OR (TI=optimism OR AB=optimism) OR (TI="positive affect" OR AB="positive affect") OR (TI=invulnerability OR AB=invulnerability) OR (TI=hardiness OR AB=hardiness) OR (TI=buffering OR AB=buffering) OR (TI="psychological well-being" OR AB="psychological well-being")) AND ((TI="child* maltreatment" OR AB="child* maltreatment") OR (TI="child* trauma" OR AB="child* trauma") OR (TI="child* advers*" OR AB="child* advers*") OR (TI="early life adversity" OR AB="early life adversity") OR (TI="early life stress" OR AB="early life stress") OR (TI="complex trauma" OR AB="complex trauma") OR (TI="child* victim*" OR AB="child* victim*") OR (TI="child* abuse" OR AB="child* abuse") OR (TI="child* neglect" OR AB="child* neglect") OR (TI="child* physical abuse" OR AB="child* physical abuse") OR (TI="child* emotional abuse" OR AB="child* emotional abuse") OR (TI="child* psychological abuse" OR AB="child* psychological abuse") OR (TI="domestic violence" OR AB="domestic violence") OR (TI="family violence" OR AB="family violence") OR (TI=bullying OR AB=bullying) OR (TI="child* sexual abuse" OR AB="child* sexual abuse") OR (TI=CTQ OR AB=CTQ) OR (TI="childhood trauma questionnaire" OR AB="childhood trauma questionnaire") OR (TI=CECA OR AB=CECA))

**PsycINFO**

Initial search on 18.04.2023, 5405 articles retrieved and updated on 12.06.2024, 426 articles retrieved.

((TI=resilience) OR AB(resilience) OR TI(resilient) OR AB(resilient) OR TI(resiliency) OR AB(resiliency) OR (TI(self-regulation) OR AB(self-regulation) OR TI(self-efficacy) OR AB(self-efficacy) OR (TI(self-organisation) OR AB(self-organisation) OR TI(self-reliance) OR AB(self-reliance) OR TI(self-esteem) OR AB(self-esteem) OR TI(self-confidence) OR AB(self-confidence) OR TI(adaptive functioning) OR AB(adaptive functioning) OR TI(adaptive coping) OR AB(adaptive coping) OR TI(competent coping) OR AB(competent coping) OR TI(successful coping) OR AB(successful coping) OR TI(social competence) OR AB(social competence) OR TI(sense of mastery) OR AB(sense of mastery) OR TI(problem solving) OR AB(problem solving) OR TI(social adjustment) OR AB(social adjustment) OR TI(motivation OR AB(motivation) OR TI(emotion regulation) OR AB(emotion regulation) OR TI(positive appraisal) OR AB(positive appraisal) OR TI(meaning in life) OR AB(meaning in life) OR TI(sense of coherence) OR AB(sense of coherence) OR TI(regulatory flexibility) OR AB(regulatory flexibility) OR TI(optimism OR AB(optimism) OR TI(positive affect) OR AB(positive affect) OR TI(invulnerability OR AB(invulnerability) OR TI(hardiness OR AB(hardiness) OR TI(buffering OR AB(buffering) OR TI(psychological well-being) OR AB(psychological well-being)) AND (TI(child maltreatment) OR AB(child maltreatment) OR TI(child trauma) OR AB(child trauma) OR TI(child advers*) OR AB(child advers*) OR TI(early life adversity) OR AB(early life adversity) OR TI(early life stress) OR AB(early life stress) OR TI(complex trauma) OR AB(complex trauma) OR TI(child victim*) OR AB(child victim*) OR TI(child abuse) OR AB(child abuse) OR TI(child neglect) OR AB(child neglect) OR TI(child physical abuse) OR AB(child physical abuse) OR TI(child emotional abuse) OR AB(child emotional abuse) OR TI(child psychological abuse) OR AB(child psychological abuse) OR TI(domestic violence) OR AB(domestic violence) OR TI(family violence) OR AB(family violence) OR TI(bullying OR AB(bullying) OR TI(child sexual abuse) OR AB(child sexual abuse) OR TI(CTQ OR AB(CTQ) OR TI(childhood trauma questionnaire) OR AB(childhood trauma questionnaire) OR TI(CECA) OR AB(CECA))

**Embase**

Initial search on 19.04.2023, 444 articles retrieved and updated on 12.06.2024, 100 articles retrieved.

#1 'resilience':ab,ti OR 'resilient':ab,ti OR 'resiliency':ab,ti OR 'self-regulation':ab,ti OR 'self-efficacy':ab,ti OR 'self-organisation':ab,ti OR 'self-reliance':ab,ti OR 'self-esteem':ab,ti OR 'self-confidence':ab,ti OR 'adaptive functioning':ab,ti OR 'adaptive coping':ab,ti OR 'competent coping':ab,ti OR 'successful coping':ab,ti OR 'social competence':ab,ti OR 'sense of mastery':ab,ti OR 'problem solving':ab,ti OR 'social adjustment':ab,ti OR 'motivation':ab,ti OR 'emotion regulation':ab,ti OR 'positive appraisal':ab,ti OR 'meaning in life':ab,ti OR 'sense of coherence':ab,ti OR 'regulatory flexibility':ab,ti OR 'optimism':ab,ti OR 'positive affect':ab,ti OR 'invulnerability':ab,ti OR 'hardiness':ab,ti OR 'buffering':ab,ti OR 'psychological well-being':ab,ti

#2 'child maltreatment':ab,ti OR 'child trauma':ab,ti OR 'child advers*':ab,ti OR 'early life adversity':ab,ti OR 'early life stress':ab,ti OR 'complex trauma':ab,ti OR 'child victim*':ab,ti OR 'child abuse':ab,ti OR 'child neglect':ab,ti OR 'child physical abuse':ab,ti OR 'child emotional abuse':ab,ti OR 'child psychological abuse':ab,ti OR 'domestic violence':ab,ti OR 'family violence':ab,ti OR 'bullying':ab,ti OR 'child sexual abuse':ab,ti OR 'ctq':ab,ti OR 'childhood trauma questionnaire':ab,ti OR 'ceca':ab,ti

#3 #1 AND #2

#4 #3 AND [embase]/lim NOT ([embase]/lim AND [medline]/lim)

#4 AND ('article'/it OR 'article in press'/it OR 'editorial'/it OR 'letter'/it OR 'note'/it OR 'review'/it OR 'short survey'/it)

#5 AND 'human'/de

# SA3. Extracted variables

Author and publication year, country and region, sample size, mean age (*SD*) and range in years, gender, sex (% male), socioeconomic status, education level (in years and/or higher education %), socioeconomic status (average/family household income), study design, mental health condition and diagnosis descriptive (*n* or % if reported), type and instrument for diagnosis (and criteria), physical condition and diagnosis descriptive (*n* or % if reported), exposure including type, severity and timing of CM, outcome including global/trait resilience and domains of resilience, variable measurement and instruments, valence of outcome and results used in meta-analysis (effect sizes, confidence intervals and *p* value), confounders, moderators, and mediators investigated in included studies.

# SA4. Definition of exposure and outcome variables

**Exposure variables**

Childhood maltreatment (CM) was defined and operationalised as: (a) ***Overall CM (or total CM):*** cumulative scores of abuse and neglect; all forms of maltreatment that caused harm to those with an age <18 years of age and that endangered their development (Fares-Otero & Seedat, 2024); (b) ***Physical abuse:*** defined as acts of violence causing physical harm or injury, including physical punishment; (c) ***Sexual abuse:*** defined as sexual acts including intercourse with or touching a child, one completed/attempted sexual act or exploitation of a child; any non-consensual, unwanted, or exploitative sexual activity that involved children or adolescents (<18 years of age); (d) ***Emotional (or psychological) abuse:*** defined as verbal or behavioural assaults toward a child that might result in trauma, including any humiliating name-calling by an adult or caregiver, or a repeated pattern of behaviours of caregivers, which conveys to a child that he/she is unwanted, worthless, unloved, and flawed; (e) ***Physical neglect:*** defined as caregivers’ failure to provide basic physical needs for the child including shelter, education, protection, food, clothing or health-care; and (f) ***Emotional (or psychological) neglect:*** defined as caregivers’ failure to meet the child’s fundamental emotional and psychological needs, including love, care, support, and belonging; (g) ***Domestic violence:*** defined as household dysfunction, parental discord, or witnessing parental/siblings victimisation; (f) ***Bullying:*** defined as emotional and/or physical peer victimisation (Goemans et al., 2023) (see CM assessment and measures in included studies in Table 1).

**Outcome variables**

Resilience was categorised into: **I) Global or trait resilience** (Connor & Davidson, 2003): defined as stable trait resilience or personal characteristics involving dimensions such as personal competence, trust, positive acceptance, control, and spiritual influence (Ye et al., 2022); and **II)** five separate domains of resilience, including: **1) Coping:** defined as conscious, volitional efforts to regulate emotion, cognition, behaviour, physiology, and the environment in response to stress (Bonanno et al., 2011, 2015); **2) Self-esteem:** defined as one’s overall sense of self-worth or personal value and represents one’s comprehensive evaluation of oneself, including positive and negative evaluations (Brown et al., 2001); **3) Emotion regulation:** defined as the process by which individuals influence the occurrence, timing, nature, experience, and expression of their emotions, and the ability to manage and regulate their emotions in a healthy and adaptive way (McRae & Gross, 2020); **5) Self-efficacy:** defined as a sense of perceived self-efficacy to cope with daily hassles and stressors and adapt after experiencing stressful life events. Self-efficacy heightens with successes and lowers with repeated failures (Caprara & Gerbino, 2001); **6) Well-being:** defined as the combination of feeling good and functioning well; the experience of positive emotions such as happiness and contentment as well as the development of one’s potential, having a sense of purpose, and experiencing positive relationships (Huppert, 2009).

# SA5. Full list of included studies

1. Allbaugh, L. J., Florez, I. A., Turmaud, D. R., Quyyum, N., Dunn, S. E., Kim, J., & Kaslow, N. J. (2017). Child Abuse - Suicide Resilience Link in African American Women: Interpersonal Psychological Mediators. *Journal of aggression, maltreatment & trauma*, *26*(10), 1055–1071. <https://doi.org/10.1080/10926771.2017.1350773>
2. Anctil, T. M., McCubbin, L. D., O'Brien, K., & Pecora, P. (2007). An evaluation of recovery factors for foster care alumni with physical or psychiatric impairments: Predictors of psychological outcomes. *Children and Youth Services Review, 29*(8), 1021–1034. [https://doi.org/10.1016/j.childyouth.2007.02.003](https://psycnet.apa.org/doi/10.1016/j.childyouth.2007.02.003)
3. Armitage, J. M., Wang, R. A. H., Davis, O. S. P., Bowes, L., & Haworth, C. M. A. (2021). Peer victimisation during adolescence and its impact on wellbeing in adulthood: a prospective cohort study. *BMC public health*, *21*(1), 148. <https://doi.org/10.1186/s12889-021-10198-w>
4. Arslan, G. (2015). Relationship between childhood psychological maltreatment, resilience, depression, and negative self-concept. *Neuropsychiatric Investigation*, *53*(4), 3–10. <https://doi.org/10.5455/NYS.20160328090400>
5. Arslan, G., & Genç, E. (2022). Psychological maltreatment and college student mental wellbeing: A uni and multi-dimensional effect of positive perception. *Children and Youth Services Review, 134*, 106371. <https://doi.org/10.1016/j.childyouth.2022.106371>
6. Artime, T. M., & Peterson, Z. D. (2012). The Relationships Among Childhood Maltreatment, Emotion Regulation, and Sexual Risk-Taking in Men from Urban STD Clinics. *Journal of aggression, maltreatment & trauma*, *21*(3), 277–299. <https://doi.org/10.1080/10926771.2012.659802>
7. Babad, S., Zwilling, A., Carson, K. W., Fairchild, V., & Nikulina, V. (2022). Childhood Environmental Instability and Social-Emotional Outcomes in Emerging Adults. *Journal of interpersonal violence*, *37*(7-8), NP3875–NP3904. <https://doi.org/10.1177/0886260520948147>
8. Berhe, O., Moessnang, C., Reichert, M., Ma, R., Höflich, A., Tesarz, J., Heim, C. M., Ebner-Priemer, U., Meyer-Lindenberg, A., & Tost, H. (2023). Dose-dependent changes in real-life affective well-being in healthy community-based individuals with mild to moderate childhood trauma exposure. *Borderline personality disorder and emotion dysregulation*, *10*(1), 14. <https://doi.org/10.1186/s40479-023-00220-5>
9. Berzenski S. R. (2019). Distinct emotion regulation skills explain psychopathology and problems in social relationships following childhood emotional abuse and neglect. *Development and psychopathology*, *31*(2), 483–496. <https://doi.org/10.1017/S0954579418000020>
10. Berzenski, S. R., & Yates, T. M. (2010). Research on intimate partner violence: A developmental process analysis of the contribution of childhood emotional abuse to relationship violence. *Journal of Aggression, Maltreatment & Trauma, 19*(2), 180–203. [https://doi.org/10.1080/10926770903539474](https://psycnet.apa.org/doi/10.1080/10926770903539474)
11. Billen, E., Garofalo, C., Schwabe, I., Jeandarme, I., & Bogaerts, S. (2022). Emotional, cognitive and behavioral self-regulation in forensic psychiatric patients: Changes over time and associations with childhood trauma, identity and personality pathology. *Psychology, Crime & Law.* Advance online publication. [https://doi.org/10.1080/1068316X.2022.2044813](https://psycnet.apa.org/doi/10.1080/1068316X.2022.2044813)
12. Blood, G. W., & Blood, I. M. (2016). Long-term Consequences of Childhood Bullying in Adults who Stutter: Social Anxiety, Fear of Negative Evaluation, Self-esteem, and Satisfaction with Life. *Journal of fluency disorders*, *50*, 72–84. <https://doi.org/10.1016/j.jfludis.2016.10.002>
13. Bouchard, G., & Sonier, N. A. (2021). Relationship between sibling bullying, family functioning, and problem solving: A structural equation modeling. *Current Psychology: A Journal for Diverse Perspectives on Diverse Psychological Issues.* Advance online publication. [https://doi.org/10.1007/s12144-021-02475-z](https://psycnet.apa.org/doi/10.1007/s12144-021-02475-z)
14. Bradley, R., Schwartz, A. C., & Kaslow, N. J. (2005). Posttraumatic stress disorder symptoms among low-income, African American women with a history of intimate partner violence and suicidal behaviors: self-esteem, social support, and religious coping. *Journal of traumatic stress*, *18*(6), 685–696. <https://doi.org/10.1002/jts.20077>
15. Brodski, S. K., & Hutz, C. S. (2012). The repercussions of emotional abuse and parenting styles on self-esteem, subjective well-being: A retrospective study with university students in Brazil. *Journal of Aggression, Maltreatment & Trauma, 21*(3), 256–276. [https://doi.org/10.1080/10926771.2012.666335](https://psycnet.apa.org/doi/10.1080/10926771.2012.666335)
16. Broekhof, R., Rius-Ottenheim, N., Spinhoven, P., van der Mast, R. C., Penninx, B. W., Zitman, F. G., & Giltay, E. J. (2015). Long-lasting effects of affective disorders and childhood trauma on dispositional optimism. *Journal of affective disorders*, *175*, 351–358. <https://doi.org/10.1016/j.jad.2015.01.022>
17. Bungert, M., Liebke, L., Thome, J., Haeussler, K., Bohus, M., & Lis, S. (2015). Rejection sensitivity and symptom severity in patients with borderline personality disorder: effects of childhood maltreatment and self-esteem. *Borderline personality disorder and emotion dysregulation*, *2*, 4. <https://doi.org/10.1186/s40479-015-0025-x>
18. Burns, E. E., Jackson, J. L., & Harding, H. G. (2010). Child maltreatment, emotion regulation, and posttraumatic stress: The impact of emotional abuse. *Journal of Aggression, Maltreatment & Trauma, 19*(8), 801–819. [https://doi.org/10.1080/10926771.2010.522947](https://psycnet.apa.org/doi/10.1080/10926771.2010.522947)
19. Cantón-Cortés, D., Cortés, M. R., & Cantón, J. (2012). The role of traumagenic dynamics on the psychological adjustment of survivors of child sexual abuse. *European Journal of Developmental Psychology, 9*(6), 665–680. [https://doi.org/10.1080/17405629.2012.660789](https://psycnet.apa.org/doi/10.1080/17405629.2012.660789)
20. Cao, H., Zhang, R., Li, L., & Yang, L. (2022). Coping Style and Resilience Mediate the Effect of Childhood Maltreatment on Mental Health Symptomology. *Children (Basel, Switzerland)*, *9*(8), 1118. https://doi.org/10.3390/children9081118
21. Cao, Q., Zhang, Q., Chen, Y., He, Z., Xiang, Z., Guan, H., Yan, N., Qiang, Y., & Li, M. (2023). The relationship between non-suicidal self-injury and childhood abuse in transgender people: a cross-sectional cohort study. *Frontiers in psychology*, *14*, 1062601. <https://doi.org/10.3389/fpsyg.2023.1062601>
22. Carvalho Fernando, S., Beblo, T., Schlosser, N., Terfehr, K., Otte, C., Löwe, B., Wolf, O. T., Spitzer, C., Driessen, M., & Wingenfeld, K. (2014). The impact of self-reported childhood trauma on emotion regulation in borderline personality disorder and major depression. *Journal of trauma & dissociation : the official journal of the International Society for the Study of Dissociation (ISSD)*, *15*(4), 384–401. <https://doi.org/10.1080/15299732.2013.863262>
23. Cecen, A. R., & Gümüş, Z. (2024). The underlying mechanism for childhood psychological maltreatment and self-satisfaction: The serial mediating roles of self-critical rumination and self-compassion. *International Journal of Mental Health and Addiction.* Advance online publication. [https://doi.org/10.1007/s11469-023-01228-2](https://psycnet.apa.org/doi/10.1007/s11469-023-01228-2)
24. Çelik, Ç. B., & Odacı, H. (2020). Does child abuse have an impact on self-esteem, depression, anxiety and stress conditions of individuals?. *The International journal of social psychiatry*, *66*(2), 171–178. <https://doi.org/10.1177/0020764019894618>
25. Chang, Y. H., Yang, M. H., Yao, Z. F., Tsai, M. C., & Hsieh, S. (2023). The Mediating Role of Brain Structural Imaging Markers in Connecting Adverse Childhood Experiences and Psychological Resilience. *Children (Basel, Switzerland)*, *10*(2), 365. <https://doi.org/10.3390/children10020365>
26. Chaturvedi S., & Arya B. (2023). Mediating Role of Self-esteem and Trust in the Relationship Between Childhood Trauma and Romantic Attachment in Indian Adults. *Journal of Research & Health, 13*(5), 313-324. <http://dx.doi.org/10.32598/JRH.13.5.2296.1>
27. Chen, Z., Shen, S., & Dai, Q. (2023). Long-term and short-term psycho-social predictors of early-adulthood depression: role of childhood trauma, neuroticism, social-support, resilience, and life-events. *Current Psychology* *42*, 3904–3916. <https://doi.org/10.1007/s12144-021-01570-5>
28. Cheng, P., & Langevin, R. (2023). Difficulties with emotion regulation moderate the relationship between child maltreatment and emotion recognition. *Child abuse & neglect*, *139*, 106094. <https://doi.org/10.1016/j.chiabu.2023.106094>
29. Chi, X. L., Huang, Q. M., Liu, X. F., Huang, L. Y., Hu, M. J., Chen, Z. J., Jiao, C., Stubbs, B., Hossain, M. M., & Zou, L. Y. (2021). Self-compassion and resilience mediate the relationship between childhood exposure to domestic violence and posttraumatic growth/stress disorder during COVID-19 pandemic. *World journal of psychiatry*, *11*(11), 1106–1115. <https://doi.org/10.5498/wjp.v11.i11.1106>
30. Choe, E., Srisarajivakul, E., & Davis, D. E. (2021). Protecting Victims of Bullying: The Protective Roles Self-Esteem and Self-Forgiveness Play between past Victimization and Current Depressive Symptoms. *Journal of School Violence*, *20*(4), 417–429. <https://doi.org/10.1080/15388220.2021.1930015>
31. Choi, J. Y., Choi, Y. M., Gim, M. S., Park, J. H., & Park, S. H. (2014). The effects of childhood abuse on symptom complexity in a clinical sample: mediating effects of emotion regulation difficulties. *Child abuse & neglect*, *38*(8), 1313–1319. <https://doi.org/10.1016/j.chiabu.2014.04.016>
32. Christ, C., de Waal, M. M., Dekker, J. J. M., van Kuijk, I., van Schaik, D. J. F., Kikkert, M. J., Goudriaan, A. E., Beekman, A. T. F., & Messman-Moore, T. L. (2019). Linking childhood emotional abuse and depressive symptoms: The role of emotion dysregulation and interpersonal problems. *PloS one*, *14*(2), e0211882. <https://doi.org/10.1371/journal.pone.0211882>
33. Clark, S. M., Immelman, T. D., Hart, A. R., & Kaslow, N. J. (2021). Childhood maltreatment and resource acquisition in African American women: The role of self-esteem. *Psychological trauma : theory, research, practice and policy*, *13*(5), 603–610. <https://doi.org/10.1037/tra0001003>
34. Cloitre, M., Stovall-McClough, C., Zorbas, P., & Charuvastra, A. (2008). Attachment organisation, emotion regulation, and expectations of support in a clinical sample of women with childhood abuse histories. *Journal of traumatic stress*, *21*(3), 282–289. <https://doi.org/10.1002/jts.20339>
35. Crosta, M. L., De Simone, C., Di Pietro, S., Acanfora, M., Caldarola, G., Moccia, L., Callea, A., Panaccione, I., Peris, K., Rinaldi, L., Janiri, L., & Di Nicola, M. (2018). Childhood trauma and resilience in psoriatic patients: A preliminary report. *Journal of psychosomatic research*, *106*, 25–28. <https://doi.org/10.1016/j.jpsychores.2018.01.002>
36. C. V. Costa, E., Simões, J., Correia, P., McIntyre, T., & Graça Pereira, M. (2024). The impact of interpersonal traumas in Portuguese women’s psychological wellbeing: predictors of depression and post traumatic stress symptoms. *Journal of Sexual Aggression*, 1–16. https://doi.org/10.1080/13552600.2023.2300478
37. Daniels, J. K., Hegadoren, K. M., Coupland, N. J., Rowe, B. H., Densmore, M., Neufeld, R. W., & Lanius, R. A. (2012). Neural correlates and predictive power of trait resilience in an acutely traumatized sample: a pilot investigation. *The Journal of clinical psychiatry*, *73*(3), 327–332. <https://doi.org/10.4088/JCP.10m06293>
38. Daruy-Filho, L., Brietzke, E., Kluwe-Schiavon, B., Fabres, C. d. S., & Grassi-Oliveira, R. (2013). Childhood maltreatment and coping in bipolar disorder. *Psychology & Neuroscience, 6*(3), 271–277. <https://doi.org/10.3922/j.psns.2013.3.05>
39. Davies, C. A., DiLillo, D., & Martinez, I. G. (2004). Isolating Adult Psychological Correlates of Witnessing Parental Violence: Findings from a Predominantly Latina Sample. *Journal of Family Violence, 19*(6), 377–385. [https://doi.org/10.1007/s10896-004-0682-9](https://psycnet.apa.org/doi/10.1007/s10896-004-0682-9)
40. Dawson, D., Strodl, E., & Kitamura, H. (2022). Childhood maltreatment and disordered eating: The mediating role of emotion regulation. *Appetite*, *172*, 105952. <https://doi.org/10.1016/j.appet.2022.105952>
41. Demir, Z., Böge, K., Fan, Y., Hartling, C., Harb, M. R., Hahn, E., Seybold, J., & Bajbouj, M. (2020). The role of emotion regulation as a mediator between early life stress and posttraumatic stress disorder, depression and anxiety in Syrian refugees. *Translational psychiatry*, *10*(1), 371. <https://doi.org/10.1038/s41398-020-01062-3>
42. Dereboy, Ç., Şahin Demirkapı, E., Şakiroğlu, M., & Şafak Öztürk, C. (2018). The Relationship Between Childhood Traumas, Identity Development, Difficulties in Emotion Regulation and Psychopathology. *Turkish journal of psychiatry*, *29*(4), 269–278. <https://www.doi.org/10.5080/u20463>
43. Di Nicola, M., Pepe, M., Montanari, S., Marcelli, I., Panaccione, I., Janiri, D., Janiri, L., & Sani, G. (2024). Childhood sexual abuse and suicide attempts in patients with substance use disorders: The mediating role of emotion dysregulation. *Child abuse & neglect*, *151*, 106731. <https://doi.org/10.1016/j.chiabu.2024.106731>
44. Ekinci, S., & Kandemir, H. (2015). Childhood trauma in the lives of substance-dependent patients: The relationship between depression, anxiety and self-esteem. *Nordic journal of psychiatry*, *69*(4), 249–253. <https://doi.org/10.3109/08039488.2014.981856>
45. ElBarazi, A. S. (2023). Childhood Maltreatment and its Mental Health Consequences among University’s Students. *The Family Journal*, *0*(0). <https://doi.org/10.1177/10664807231157022>
46. Endo, M., Ono, M., Deguchi, A., Iwata, Y., Tamada, Y., Masuya, J., Tanabe, H., Hashimoto, N., Inoue, T., & Honyashiki, M. (2024). Effects of Subjective Social Status and Self-Esteem in the Association Between Childhood Abuse and Adulthood Anxiety. *Neuropsychiatric disease and treatment*, *20*, 877–884. <https://doi.org/10.2147/NDT.S440616>
47. Erol, Y., & Inozu, M. (2024). An Investigation of the Mediating Roles of Emotion Regulation Difficulties, Distress Tolerance, Self-Compassion, and Self-Disgust in the Association Between Childhood Trauma and Nonsuicidal Self-Injury. *Archives of suicide research : official journal of the International Academy for Suicide Research*, *28*(3), 815–829. <https://doi.org/10.1080/13811118.2023.2237083>
48. Feinauer, L. L., Mitchell, J., Harper, J. M., & Dane, S. (1996). The impact of hardiness and severity of childhood sexual abuse on adult adjustment. *American Journal of Family Therapy, 24*(3), 206–214. [https://doi.org/10.1080/01926189608251034](https://psycnet.apa.org/doi/10.1080/01926189608251034)
49. Fereidooni, F., Daniels, J. K., D Krause-Utz, A., Hagenaars, M. A., Smeets, T., Heins, J., Dorahy, M. J., Emmerik, A. A. P. V., de Jong, P. J., Hoekstra, S., Warrens, M. J., & Lommen, M. J. J. (2023). Childhood maltreatment and adulthood victimization: An evidence-based model. *Journal of psychiatric research*, *167*, 46–62. <https://doi.org/10.1016/j.jpsychires.2023.10.007>
50. Festinger, T., & Baker, A. (2010). Prevalence of recalled childhood emotional abuse among child welfare staff and related well-being factors. *Children and Youth Services Review, 32*(4), 520–526. [https://doi.org/10.1016/j.childyouth.2009.11.004](https://psycnet.apa.org/doi/10.1016/j.childyouth.2009.11.004)
51. Fitzgerald, M., & Barton, C. (2022). Self-qualities and self-leadership as pathways linking childhood maltreatment to depression and relationship quality. *Contemporary Family Therapy: An International Journal, 44*(2), 156–166. [https://doi.org/10.1007/s10591-021-09577-7](https://psycnet.apa.org/doi/10.1007/s10591-021-09577-7)
52. Fitzgerald, M., & Esplin, J. A. (2023). Marital quality as a mechanism linking childhood abuse to mental health. *Journal of Family Issues, 44*(6), 1488–1507. [https://doi.org/10.1177/0192513X211059831](https://psycnet.apa.org/doi/10.1177/0192513X211059831)
53. Fleming, J., Mullen, P. E., Sibthorpe, B., & Bammer, G. (1999). The long-term impact of childhood sexual abuse in Australian women. *Child abuse & neglect*, *23*(2), 145–159. <https://doi.org/10.1016/s0145-2134(98)00118-5>
54. Fossati, A., Gratz, K. L., Somma, A., Maffei, C., & Borroni, S. (2016). The Mediating Role of Emotion Dysregulation in the Relations Between Childhood Trauma History and Adult Attachment and Borderline Personality Disorder Features: A Study of Italian Nonclinical Participants. *Journal of personality disorders*, *30*(5), 653–676. <https://doi.org/10.1521/pedi_2015_29_222>
55. Fosse, G. K., & Holen, A. (2007). Reported maltreatment in childhood in relation to the personality features of Norwegian adult psychiatric outpatients. *The Journal of nervous and mental disease*, *195*(1), 79–82. <https://doi.org/10.1097/01.nmd.0000252312.98109.d4>
56. Fox, K. M., & Gilbert, B. O. (1994). The interpersonal and psychological functioning of women who experienced childhood physical abuse, incest, and parental alcoholism. *Child abuse & neglect*, *18*(10), 849–858. <https://doi.org/10.1016/0145-2134(94)90064-7>
57. Galea, M., Ciarrocchi, J. W., Piedmont, R. L., & Wicks, R. J. (2007). Child abuse, personality, and spirituality as predictors of happiness in Maltese college students. *Research in the Social Scientific Study of Religion, 18,* 141–154. [https://doi.org/10.1163/ej.9789004158511.i-301.57](https://psycnet.apa.org/doi/10.1163/ej.9789004158511.i-301.57)
58. Gambaro, E., Mastrangelo, M., Sarchiapone, M., Marangon, D., Gramaglia, C., Vecchi, C., Airoldi, C., Mirisola, C., Costanzo, G., Bartollino, S., Baralla, F., & Zeppegno, P. (2020). Resilience, trauma, and hopelessness: protective or triggering factor for the development of psychopathology among migrants?. *BMC psychiatry*, *20*(1), 358. <https://doi.org/10.1186/s12888-020-02729-3>
59. Garcia, C. O., & Berzenski, S. R. (2023). The importance of perception and personality on the association between childhood neglect and adult social competence. *Journal of Aggression, Maltreatment & Trauma, 32*(10), 1337–1352. [https://doi.org/10.1080/10926771.2023.2189045](https://awspntest.apa.org/doi/10.1080/10926771.2023.2189045)
60. Garofalo, C., Delvecchio, E., Bogaerts, S., Sellbom, M., & Mazzeschi, C. (2024). Childhood trauma and psychopathy: The moderating role of resilience. *Psychological trauma : theory, research, practice and policy*, 10.1037/tra0001687. Advance online publication. <https://doi.org/10.1037/tra0001687>
61. Goldbach, R. E., Neukel, C., Panizza, A., Reinken, A., & Krause-Utz, A. (2023). Differentiating between intrapsychic symptoms and behavioral expressions of borderline personality disorder in relation to childhood emotional maltreatment and emotion dysregulation: an exploratory investigation. *European journal of psychotraumatology*, *14*(2), 2263317. <https://doi.org/10.1080/20008066.2023.2263317>
62. Goldstein, A. L., Faulkner, B., & Wekerle, C. (2013). The relationship among internal resilience, smoking, alcohol use, and depression symptoms in emerging adults transitioning out of child welfare. *Child abuse & neglect*, *37*(1), 22–32. <https://doi.org/10.1016/j.chiabu.2012.08.007>
63. Goodboy, A. K., Martin, M. M., & Goldman, Z. W. (2016). Students’ experiences of bullying in high school and their adjustment and motivation during the first semester of college. *Western Journal of Communication, 80*(1), 60–78. [https://doi.org/10.1080/10570314.2015.1078494](https://psycnet.apa.org/doi/10.1080/10570314.2015.1078494)
64. Griffing, S., Lewis, C. S., Chu, M., Sage, R., Jospitre, T., Madry, L., & Primm, B. J. (2006). The process of coping with domestic violence in adult survivors of childhood sexual abuse. *Journal of Child Sexual Abuse: Research, Treatment, & Program Innovations for Victims, Survivors, & Offenders, 15*(2), 23–41. [https://doi.org/10.1300/J070v15n02_02](https://psycnet.apa.org/doi/10.1300/J070v15n02_02)
65. Güler, K., Gümüş Demir, Z., Yurtseven, C. S. (2023). Investigation of the relationship between childhood traumas, psychological resilience, cognitive flexibility and emotion regulation skills in adults. *The European Research Journal, 10*(2), 166-177. <https://doi.org/10.18621/eurj.1279884>
66. Guo, X., Huang, J., & Yang, Y. (2022). The Association between Differentiation of Self and Life Satisfaction among Chinese Emerging Adults: The Mediating Effect of Hope and Coping Strategies and the Moderating Effect of Child Maltreatment History. *International journal of environmental research and public health*, *19*(12), 7106. <https://doi.org/10.3390/ijerph19127106>
67. Haj-Yahia, M. M., Hassan-Abbas, N., Malka, M., & Sokar, S. (2021). Exposure to Family Violence in Childhood, Self-Efficacy, and Posttraumatic Stress Symptoms in Young Adulthood. *Journal of interpersonal violence*, *36*(17-18), NP9548–NP9575. https://doi.org/10.1177/0886260519860080
68. He, J., Yan, X., Wang, R., Zhao, J., Liu, J., Zhou, C., & Zeng, Y. (2022). Does Childhood Adversity Lead to Drug Addiction in Adulthood? A Study of Serial Mediators Based on Resilience and Depression. *Frontiers in psychiatry*, *13*, 871459. <https://doi.org/10.3389/fpsyt.2022.871459>
69. Hengartner, M. P., Müller, M., Rodgers, S., Rössler, W., & Ajdacic-Gross, V. (2013). Can protective factors moderate the detrimental effects of child maltreatment on personality functioning?. *Journal of psychiatric research*, *47*(9), 1180–1186. <https://doi.org/10.1016/j.jpsychires.2013.05.005>
70. Herrenkohl, T. I., Klika, J. B., Herrenkohl, R. C., Russo, M. J., & Dee, T. (2012). A prospective investigation of the relationship between child maltreatment and indicators of adult psychological well-being. *Violence and victims*, *27*(5), 764–776. <https://doi.org/10.1891/0886-6708.27.5.764>
71. Heshmati, R., Haghi, N., & Brownridge, D. A. (2021). Pathways Linking Childhood Maltreatment to Impulsivity: The Mediating Role of Affect Balance. *Journal of Aggression, Maltreatment & Trauma*, *30*(8), 991–1006. <https://doi.org/10.1080/10926771.2021.1933289>
72. Higgins, D. J., & McCabe, M. P. (1994). The relationship of child sexual abuse and family violence to adult adjustment: Toward an integrated risk-sequelae model. *Journal of Sex Research, 31*(4), 255–266. [https://doi.org/10.1080/00224499409551761](https://psycnet.apa.org/doi/10.1080/00224499409551761)
73. Hu, H., Chen, C., Xu, B., & Wang, D. (2024). Moderating and mediating effects of resilience between childhood trauma and psychotic-like experiences among college students. *BMC psychiatry*, *24*(1), 273. <https://doi.org/10.1186/s12888-024-05719-x>
74. Ion, A., Bîlc, M. I., Pițur, S., Pop, C. F., Szentágotai-Tătar, A., & Miu, A. C. (2023). Childhood maltreatment and emotion regulation in everyday life: an experience sampling study. *Scientific reports*, *13*(1), 7214. https://doi.org/10.1038/s41598-023-34302-9
75. Janiri, D., Moccia, L., Dattoli, L., Pepe, M., Molinaro, M., De Martin, V., Chieffo, D., Di Nicola, M., Fiorillo, A., Janiri, L., & Sani, G. (2021). Emotional dysregulation mediates the impact of childhood trauma on psychological distress: First Italian data during the early phase of COVID-19 outbreak. *The Australian and New Zealand journal of psychiatry*, *55*(11), 1071–1078. <https://doi.org/10.1177/0004867421998802>
76. Jennissen, S., Holl, J., Mai, H., Wolff, S., & Barnow, S. (2016). Emotion dysregulation mediates the relationship between child maltreatment and psychopathology: A structural equation model. *Child abuse & neglect*, *62*, 51–62. <https://doi.org/10.1016/j.chiabu.2016.10.015>
77. Johnson, P. (2001). In their own voices: Report of a study on the later effects of child sexual abuse. *Journal of Sexual Aggression, 7*(2), 41–56. [https://doi.org/10.1080/13552600108416166](https://psycnet.apa.org/doi/10.1080/13552600108416166)
78. Jones, E. J., Marsland, A. L., & Gianaros, P. J. (2023). Do trait-level emotion regulation strategies moderate associations between retrospective reports of childhood trauma and prospective changes in systemic inflammation?. *Stress and health : journal of the International Society for the Investigation of Stress*, *39*(3), 525–538. <https://doi.org/10.1002/smi.3205>
79. Jonzon, E., & Lindblad, F. (2006). Risk factors and protective factors in relation to subjective health among adult female victims of child sexual abuse. *Child Abuse & Neglect, 30*(2), 127–143. [https://doi.org/10.1016/j.chiabu.2005.08.014](https://psycnet.apa.org/doi/10.1016/j.chiabu.2005.08.014)
80. Kanai, Y., Takaesu, Y., Nakai, Y., Ichiki, M., Sato, M., Matsumoto, Y., Ishikawa, J., Ono, Y., Murakoshi, A., Tanabe, H., Kusumi, I., & Inoue, T. (2016). The influence of childhood abuse, adult life events, and affective temperaments on the well-being of the general, nonclinical adult population. *Neuropsychiatric disease and treatment*, *12*, 823–832. <https://doi.org/10.2147/NDT.S100474>
81. Kanj, G., Hallit, S., & Obeid, S. (2023). The relationship between childhood emotional abuse and borderline personality disorder: the mediating role of difficulties in emotion regulation among Lebanese adults. *Borderline personality disorder and emotion dysregulation*, *10*(1), 34. <https://doi.org/10.1186/s40479-023-00241-0>
82. Kapoor, S., Domingue, H. K., Watson-Singleton, N. N., Are, F., Elmore, C. A., Crooks, C. L., Madden, A., Mack, S. A., Peifer, J. S., & Kaslow, N. J. (2018). Childhood abuse, intrapersonal strength, and suicide resilience in African American females who attempted suicide. *Journal of Family Violence, 33*(1), 53–64. [https://doi.org/10.1007/s10896-017-9943-2](https://psycnet.apa.org/doi/10.1007/s10896-017-9943-2)
83. Karagöz, B., & Dağ, İ. (2015). The Relationship between Childhood Maltreatment and Emotional Dysregulation in Self Mutilation: An Investigation among Substance Dependent Patients. *Noro psikiyatri arsivi*, *52*(1), 8–14. <https://doi.org/10.5152/npa.2015.6769>
84. Karakaş, M., & Çingöl, N. (2022). The relationship of childhood trauma experiences with cognitive distortions and sense of coherence in nursing students. *Perspectives in psychiatric care*, *58*(4), 1546–1553. <https://doi.org/10.1111/ppc.12962>
85. Kazan Kızılkurt, Ö., Demirkan, A. K., Gıynaş, F. E., & Güleç, H. (2021). Effect of childhood trauma on disease severity in patients with fibromyalgia: The mediating role of psychological resilience. *Archives of rheumatology*, *36*(4), 538–547. <https://doi.org/10.46497/ArchRheumatol.2021.8477>
86. Kesebir, S., Ünübol, B., Tatlıdil Yaylacı, E., Gündoğar, D., & Ünübol, H. (2015). Impact of childhood trauma and affective temperament on resilience in bipolar disorder. *International journal of bipolar disorders*, *3*, 3. <https://doi.org/10.1186/s40345-015-0023-3>
87. Khosravani, V., Samimi Ardestani, S. M., Sharifi Bastan, F., Mohammadzadeh, A., & Amirinezhad, A. (2019). Childhood maltreatment, cognitive emotion regulation strategies, and alcohol craving and dependence in alcohol-dependent males: Direct and indirect pathways. *Child abuse & neglect*, *98*, 104197. <https://doi.org/10.1016/j.chiabu.2019.104197>
88. Kim, E. Y., Park, J., & Kim, B. (2016). Type of childhood maltreatment and the risk of criminal recidivism in adult probationers: a cross-sectional study. *BMC psychiatry*, *16*, 294. <https://doi.org/10.1186/s12888-016-1001-8>
89. Kim, M., Hong, G., Kim, R. Y., Song, Y., Lee, H., Joo, Y., Kim, J., & Yoon, S. (2021). Severity of post-traumatic stress disorder and childhood abuse in adult crime victims as mediated by low resilience and dysfunctional coping strategies. *Child abuse & neglect*, *118*, 105154. <https://doi.org/10.1016/j.chiabu.2021.105154>
90. Kızıltepe, R., Ebeoğlu-Duman, M., Sağel-Çetiner, E., & Hecker, T. (2023). The unique contribution of childhood maltreatment types to risk-taking behavior and self-esteem. *Current Psychology: A Journal for Diverse Perspectives on Diverse Psychological Issues.* Advance online publication. [https://doi.org/10.1007/s12144-023-04300-1](https://psycnet.apa.org/doi/10.1007/s12144-023-04300-1)
91. Koçak, Z., & Çağatay, S. E. (2024). Childhood traumas and emotional eating: The mediating role of self-esteem, and emotion dysregulation. *Current Psychology: A Journal for Diverse Perspectives on Diverse Psychological Issues, 43*(25), 21783–21791. [https://doi.org/10.1007/s12144-024-05953-2](https://psycnet.apa.org/doi/10.1007/s12144-024-05953-2)
92. Kong, J., Homan, K. J., & Goldberg, J. (2024). Longitudinal trajectories of adult sibling relationship quality and psychological well‐being: The effect of childhood maltreatment. *Family Relations: An Interdisciplinary Journal of Applied Family Studies, 73*(2), 891–904. [https://doi.org/10.1111/fare.12945](https://psycnet.apa.org/doi/10.1111/fare.12945)
93. Krause-Utz, A., Černáková, R., Hoogenboom, W., Schulze, A., Büttner, S., Demirelli, Z., Mouthaan, J., van Schie, C. C., Garnefski, N., & Kraaij, V. (2023). Psychological Factors Linked to Intimate Partner Violence and Childhood Maltreatment: On Dissociation as a Possible Bridge Symptom. *Journal of interpersonal violence*, *38*(21-22), 11400–11428. <https://doi.org/10.1177/08862605231181377>
94. Krvavac, S., & Jansson, B. (2021). The role of emotion dysregulation and alexithymia in the link between types of child abuse and neglect and psychopathology: A moderated mediation model. *European Journal of Trauma & Dissociation, 5*(3), Article 100213. [https://doi.org/10.1016/j.ejtd.2021.100213](https://psycnet.apa.org/doi/10.1016/j.ejtd.2021.100213)
95. Kumar, S. A., Brockdorf, A. N., Jaffe, A. E., Church, H. R., Messman, T. L., & DiLillo, D. (2022). Mindful awareness promotes resilience: Buffered links among childhood sexual abuse severity, goal-directed emotion dysregulation, and psychopathology. *Mindfulness, 13*(4), 993–1006. [https://doi.org/10.1007/s12671-022-01854-2](https://psycnet.apa.org/doi/10.1007/s12671-022-01854-2)
96. Kuo, J. R., Khoury, J. E., Metcalfe, R., Fitzpatrick, S., & Goodwill, A. (2015). An examination of the relationship between childhood emotional abuse and borderline personality disorder features: the role of difficulties with emotion regulation. *Child abuse & neglect*, *39*, 147–155. <https://doi.org/10.1016/j.chiabu.2014.08.008>
97. Kurtuluş, C., & Elemo, A. S. (2023). Childhood emotional neglect and risks of substance misuse: Meaning and purpose of life as a mediator. *International Journal of Mental Health and Addiction.* Advance online publication. [https://doi.org/10.1007/s11469-023-01096-w](https://psycnet.apa.org/doi/10.1007/s11469-023-01096-w)
98. Lacelle, C., Hébert, M., Lavoie, F., Vitaro, F., & Tremblay, R. E. (2012). Sexual health in women reporting a history of child sexual abuse. *Child abuse & neglect*, *36*(3), 247–259. <https://doi.org/10.1016/j.chiabu.2011.10.011>
99. Laghaei, M., Mehrabizadeh Honarmand, M., Jobson, L., Abdollahpour Ranjbar, H., & Habibi Asgarabad, M. (2023). Pathways from childhood trauma to suicidal ideation: mediating through difficulties in emotion regulation and depressive symptoms. *BMC psychiatry*, *23*(1), 295. <https://doi.org/10.1186/s12888-023-04699-8>
100. Lassri, D., Bregman-Hai, N., Soffer-Dudek, N., & Shahar, G. (2023). The Interplay Between Childhood Sexual Abuse, Self-Concept Clarity, and Dissociation: A Resilience-Based Perspective. *Journal of interpersonal violence*, *38*(3-4), 2313–2336. <https://doi.org/10.1177/08862605221101182>
101. Latzer, Y., Rozenstain-Hason, M., Kabakov, O., Givon, M., Mizrachi, S., Alon, S., & Tzischinsky, O. (2020). Childhood maltreatment in patients with binge eating disorder with and without night eating syndrome vs. control. *Psychiatry research*, *293*, 113451. <https://doi.org/10.1016/j.psychres.2020.113451>
102. Lewis, C. S., Griffing, S., Chu, M., Jospitre, T., Sage, R. E., Madry, L., & Primm, B. J. (2006). Coping and violence exposure as predictors of psychological functioning in domestic violence survivors. *Violence against women*, *12*(4), 340–354. <https://doi.org/10.1177/1077801206287285>
103. Li, B.; Pan, Y.; Liu, G.; Chen, W.; Lu, J.; Li, X. (2020). Perceived social support and self-esteem mediate the relationship between childhood maltreatment and psychosocial flourishing in Chinese undergraduate students. *Children and Youth Services Review, 117*, 105303. <https://doi.org/10.1016/j.childyouth.2020.105303>
104. Li, C., Fu, P., Wang, M., Xia, Y., Hu, C., Liu, M., Zhang, H., Sheng, X., & Yang, Y. (2023). The role of self-esteem and emotion regulation in the associations between childhood trauma and mental health in adulthood: a moderated mediation model. *BMC psychiatry*, *23*(1), 241. <https://doi.org/10.1186/s12888-023-04719-7>
105. Li, C., Lv, G., Liu, B., Ju, Y., Wang, M., Dong, Q., Sun, J., Lu, X., Zhang, L., Wan, P., Guo, H., Zhao, F., Liao, M., Zhang, Y., Li, L., & Liu, J. (2023). Impact of childhood maltreatment on adult resilience. *BMC psychiatry*, *23*(1), 637. <https://doi.org/10.1186/s12888-023-05124-w>
106. Li, C., Zhu, N., Zhang, L., Li, W., & Kong, F. (2023). The relation between childhood maltreatment and hedonic and eudaimonic well-being in emerging adults: A daily diary study. *Child abuse & neglect*, *138*, 106057. <https://doi.org/10.1016/j.chiabu.2023.106057>
107. Li, W., Zhang, S., Lin, H., Zhang, K., Zhang, X., Chen, J., Xu, F., & Liu, C. (2023). Childhood Maltreatment and Creativity among Chinese College Students: A Serial Mediation Model. *Journal of Intelligence*, *11*(4), 58. <https://doi.org/10.3390/jintelligence11040058>
108. Liu, J., Tan, R. H. S., Chang, S. S. H., Teh, W. L., Shahwan, S., Lee, Y. W., Chandwani, N., Chan, C. Y. W., Tor, P. C., & Subramaniam, M. (2024). Pathological personality explains individual differences in global emotion dysregulation within the pathway between child maltreatment and severe depressive symptoms. *Psychological trauma : theory, research, practice and policy*, *16*(Suppl 1), S233–S241. <https://doi.org/10.1037/tra0001581>
109. Liu, S., Ahemaitijiang, N., Xu, J., Liu, Y., Chen, L., & Han, Z. R. (2023). Patterns of childhood maltreatment influence sleep quality: The role of emotion regulation. *Development and psychopathology*, 1–11. Advance online publication. <https://doi.org/10.1017/S0954579423000597>
110. Lu, F. Y., Wen, S., Deng, G., & Tang, Y. L. (2017). Self-concept mediate the relationship between childhood maltreatment and abstinence motivation as well as self-efficacy among drug addicts. *Addictive behaviors*, *68*, 52–58. <https://doi.org/10.1016/j.addbeh.2017.01.017>
111. Maftei, A., & Nițu, Ș. (2022). Does childhood maltreatment make us more morally disengaged? The indirect effect of expressive suppression. *Ethics & Behavior.* Advance online publication. [https://doi.org/10.1080/10508422.2022.2162522](https://psycnet.apa.org/doi/10.1080/10508422.2022.2162522)
112. Mandavia, A., Robinson, G. G., Bradley, B., Ressler, K. J., & Powers, A. (2016). Exposure to Childhood Abuse and Later Substance Use: Indirect Effects of Emotion Dysregulation and Exposure to Trauma. *Journal of traumatic stress*, *29*(5), 422–429. <https://doi.org/10.1002/jts.22131>
113. Martin, L. N., Renshaw, K. D., Mauro, K. L., Curby, T. W., Ansell, E., & Chaplin, T. (2023). Intergenerational effects of childhood maltreatment: Role of emotion dysregulation and emotion socialization. *Journal of Child and Family Studies, 32*(7), 2187–2197. [https://doi.org/10.1007/s10826-023-02608-x](https://psycnet.apa.org/doi/10.1007/s10826-023-02608-x)
114. Martínez, P., Gloger, S., Dagnino, P., & de Medina, D. D. (2023). Early adverse stress and depression severity: A pilot exploration of mediating psychological mechanisms. *Development and psychopathology*, 1–10. Advance online publication. <https://doi.org/10.1017/S0954579423000688>
115. Martxueta, A., & Etxeberria, J. (2014). Análisis diferencial retrospectivo de las variables de salud mental en lesbianas, gais y bisexuales (LGB) víctimas de bullying homofóbico en la escuela [Retrospective differential analysis of mental health variables in lesbians, gays and bisexuals (LGB) who suffered homophobic bullying at school]. *Revista de Psicopatología y Psicología Clínica, 19*(1), 23–35. [https://doi.org/10.5944/rppc.vol.19.num.1.2014.12980](https://psycnet.apa.org/doi/10.5944/rppc.vol.19.num.1.2014.12980)
116. Maxwell, K., & Huprich, S. (2014). Retrospective reports of attachment disruptions, parental abuse and neglect mediate the relationship between pathological narcissism and self-esteem. *Personality and mental health*, *8*(4), 290–305. <https://doi.org/10.1002/pmh.1269>
117. Merians, A.N., Frazier, P. (2024). Adaptive Functioning in College Students Following Childhood Maltreatment. *Adversity and Resilience Science,* *5*, 283–293 <https://doi.org/10.1007/s42844-023-00124-x>
118. Mohammadpanah Ardakan, A., Khosravani, V., Kamali, Z., & Dabiri, S. (2024). The experience of being emotionally maltreated and self-regulatory strategies in obsessive-compulsive disorder: Pathways to depressive symptoms. *Journal of Rational-Emotive & Cognitive-Behavior Therapy.* Advance online publication. [https://doi.org/10.1007/s10942-024-00552-3](https://psycnet.apa.org/doi/10.1007/s10942-024-00552-3)
119. Mohammadzadeh, A., Ganji, Z., Khosravani, V., Mohammadpanah Ardakan, A., & Amirinezhad, A. (2019). Direct and indirect associations between perception of childhood trauma and suicidal ideation through emotion dysregulation in males who use heroin. *Addictive behaviors*, *98*, 106011. https://doi.org/10.1016/j.addbeh.2019.05.035
120. Mondolin, V., Karlsson, H., Tuulari, J. J., Pelto, J., Karlsson, L., Nordenswan, E., & Kataja, E. L. (2024). Childhood maltreatment, trait resilience and prenatal distress among expecting mothers and fathers in the FinnBrain Birth Cohort Study. *Journal of affective disorders*, *344*, 41–47. <https://doi.org/10.1016/j.jad.2023.10.026>
121. Moreira, D., Silva, C., Moreira, P., Pinto, T. M., Costa, R., Lamela, D., Jongenelen, I., & Pasion, R. (2024). Addressing the Complex Links between Psychopathy and Childhood Maltreatment, Emotion Regulation, and Aggression-A Network Analysis in Adults. *Behavioral sciences (Basel, Switzerland)*, *14*(2), 115. <https://doi.org/10.3390/bs14020115>
122. Musella, K. E., DiFonte, M. C., Michel, R., Stamates, A., & Flannery-Schroeder, E. (2024). Emotion regulation as a mediator in the relationship between childhood maltreatment and symptoms of social anxiety among college students. *Journal of American college health : J of ACH*, 1–8. Advance online publication. <https://doi.org/10.1080/07448481.2024.2325926>
123. Naderzadeh, S., Khoran, Z., Khanjani, M., & Wiesmann, U. (2023). Childhood maltreatment, late-life depression, and sense of coherence: a structural equation modeling. *Aging & mental health*, *27*(5), 965–972. <https://doi.org/10.1080/13607863.2022.2076203>
124. Naughton, C. M., O'Donnell, A. T., & Muldoon, O. T. (2020). Exposure to Domestic Violence and Abuse: Evidence of Distinct Physical and Psychological Dimensions. *Journal of interpersonal violence*, *35*(15-16), 3102–3123. <https://doi.org/10.1177/0886260517706763>
125. Newman, M. L., Holden, G. W., & Delville, Y. (2011). Coping with the stress of being bullied: Consequences of coping strategies among college students. *Social Psychological and Personality Science, 2*(2), 205–211. [https://doi.org/10.1177/1948550610386388](https://psycnet.apa.org/doi/10.1177/1948550610386388)
126. Nimphy, C. A., Kullberg, M. J., Pittner, K., Buisman, R., van den Berg, L., Alink, L., Bakermans-Kranenburg, M., Elzinga, B. M., & Tollenaar, M. (2024). The Role of Psychopathology and Emotion Regulation in the Intergenerational Transmission of Childhood Abuse: A Family Study. *Child maltreatment*, 10775595231223657. Advance online publication. <https://doi.org/10.1177/10775595231223657>
127. Ozakar Akca, S., Oztas, G., Karadere, M. E., & Yazla Asafov, E. (2022). Childhood trauma and its relationship with suicide probability and Self-Esteem: A case study in a university in Turkey. *Perspectives in psychiatric care*, *58*(4), 1839–1846. <https://doi.org/10.1111/ppc.12997>
128. Pabian, S., Dehue, F., Völlink, T., & Vandebosch, H. (2022). Exploring the perceived negative and positive long-term impact of adolescent bullying victimization: A cross-national investigation. *Aggressive behavior*, *48*(2), 205–218. <https://doi.org/10.1002/ab.22006>
129. Park, J. Y., Lee, C. W., Jang, Y., Lee, W., Yu, H., Yoon, J., Oh, S., Park, Y. S., Ryoo, H. A., Lee, J., Cho, N., Lee, C. H., Lee, Y. C., Won, H. H., Kang, H. S., Ha, T. H., & Myung, W. (2023). Relationship between childhood trauma and resilience in patients with mood disorders. *Journal of affective disorders*, *323*, 162–170. <https://doi.org/10.1016/j.jad.2022.11.003>
130. Peng, W., Liu, Z., Liu, Q., Chu, J., Zheng, K., Wang, J., Wei, H., Zhong, M., Ling, Y., & Yi, J. (2021). Insecure attachment and maladaptive emotion regulation mediating the relationship between childhood trauma and borderline personality features. *Depression and anxiety*, *38*(1), 28–39. <https://doi.org/10.1002/da.23082>
131. Pourshahriar, H., Alizade, H., & Rajaeinia, K. (2018). Childhood emotional abuse and borderline personality disorder features: The mediating roles of attachment style and emotion regulation. *Iranian Journal of Psychiatry and Clinical Psychology, 24*(2), 148–162. <http://dx.doi.org/10.32598/ijpcp.24.2.148>
132. Qin, Q., Jiang, Y., Mei, X., Zhu, Y., Li, H., Li, S., Chen, K., Zhang, C., Wang, X., Bai, H., Zhang, J., & Wang, W. (2024). The effect of childhood trauma on depression in college students: A moderated mediation model. *Journal of affective disorders*, *352*, 490–497. <https://doi.org/10.1016/j.jad.2024.02.067>
133. Racine, S. E., & Wildes, J. E. (2015). Emotion dysregulation and anorexia nervosa: an exploration of the role of childhood abuse. *The International journal of eating disorders*, *48*(1), 55–58. <https://doi.org/10.1002/eat.22364>
134. Richardson, T., Egglishaw, A., & Sood, M. (2024). Does Childhood Trauma Predict Impulsive Spending in Later Life? An Analysis of the Mediating Roles of Impulsivity and Emotion Regulation. *Journal of child & adolescent trauma*, *17*(2), 275–281. <https://doi.org/10.1007/s40653-023-00600-7>
135. Rodriguez, V. J., Are, F., Madden, A., Shaffer, A., & Suveg, C. (2021). Intergenerational transmission of childhood maltreatment mediated by maternal emotion dysregulation. *Journal of Child and Family Studies, 30*(8), 2068–2075. [https://doi.org/10.1007/s10826-021-02020-3](https://psycnet.apa.org/doi/10.1007/s10826-021-02020-3)
136. Romans, S. E., Martin, J. L., Anderson, J. C., O'Shea, M. L., & Mullen, P. E. (1995). Factors that mediate between child sexual abuse and adult psychological outcome. *Psychological Medicine, 25*(1), 127–142. [https://doi.org/10.1017/S0033291700028154](https://psycnet.apa.org/doi/10.1017/S0033291700028154)
137. Rong, F., Kang, C., Peng, C., Wang, M., Cheng, J., Ding, H., & Yu, Y. (2022). Childhood maltreatment and nonsuicidal self-injury among younger chinese prisoners: The mediating role of self-esteem. *Current Psychology: A Journal for Diverse Perspectives on Diverse Psychological Issues.* Advance online publication. [https://doi.org/10.1007/s12144-022-03647-1](https://psycnet.apa.org/doi/10.1007/s12144-022-03647-1)
138. Rostami, M., Sabbagh, & Shirkhani, M. (2023). Investigating the mediating role of emotion regulation difficulty in the relationship between traumatic childhood experiences and self-criticism in adulthood. *International Journal of Behavioral Sciences, 17*(1), 16-23. <https://doi.org/10.30491/IJBS.2023.376907.1883>
139. Sachs-Ericsson, N., Medley, A. N., Kendall-Tackett, K., & Taylor, J. (2011). Childhood Abuse and Current Health Problems among Older Adults: The Mediating Role of Self-Efficacy. *Psychology of violence*, *1*(2), 106–120. <https://doi.org/10.1037/a0023139>
140. Salles, J., Stephan, F., Molière, F., Bennabi, D., Haffen, E., Bouvard, A., Walter, M., Allauze, E., Llorca, P. M., Genty, J. B., Leboyer, M., Holtzmann, J., Nguon, A. S., D'Amato, T., Rey, R., Horn, M., Vaiva, G., Fond, G., Richieri, R., Hennion, V., … Yrondi, A. (2024). Indirect effect of impulsivity on suicide risk through self-esteem and depressive symptoms in a population with treatment-resistant depression: A FACE-DR study. *Journal of affective disorders*, *347*, 306–313. <https://doi.org/10.1016/j.jad.2023.11.063>
141. Schulz, A., Becker, M., Van der Auwera, S., Barnow, S., Appel, K., Mahler, J., Schmidt, C. O., John, U., Freyberger, H. J., & Grabe, H. J. (2014). The impact of childhood trauma on depression: does resilience matter? Population-based results from the Study of Health in Pomerania. *Journal of psychosomatic research*, *77*(2), 97–103. <https://doi.org/10.1016/j.jpsychores.2014.06.008>
142. See Mey, L., Khairudin, R., Tengku Muda, T. E. A., Abdullah Mohd Nor, H., & Kamaluddin, M. R. (2022). The Mediating Role of Forgiveness and Self-Efficacy in the Relationship Between Childhood Maltreatment and Treatment Motivation Among Malaysian Male Drug Addicts. *Frontiers in psychology*, *13*, 816373. <https://doi.org/10.3389/fpsyg.2022.816373>
143. Sehlikoğlu, Ş., Sehlikoğlu, K., & Eğilmez, O. B. (2023). Examination of childhood trauma and self-esteem of individuals who applied to the probation office due to substance use. *Journal of ethnicity in substance abuse*, *22*(4), 858–878. <https://doi.org/10.1080/15332640.2022.2115435>
144. Sexton, M. B., Hamilton, L., McGinnis, E. W., Rosenblum, K. L., & Muzik, M. (2015). The roles of resilience and childhood trauma history: main and moderating effects on postpartum maternal mental health and functioning. *Journal of affective disorders*, *174*, 562–568. <https://doi.org/10.1016/j.jad.2014.12.036>
145. Sezer Katar, K., Kurtoğlu, M. B., Zengin İspir, G., & Danışman, M. (2023). Resilience and traumatic childhood experiences of patients with opioid use disorder. *Journal of ethnicity in substance abuse*, 1–13. Advance online publication. <https://doi.org/10.1080/15332640.2023.2278469>
146. Shen A. C. (2009). Self-esteem of young adults experiencing interparental violence and child physical maltreatment: parental and peer relationships as mediators. *Journal of interpersonal violence*, *24*(5), 770–794. https://doi.org/10.1177/0886260508317188
147. Shen, F., & Soloski, K. L. (2022). Examining the moderating role of childhood attachment for the relationship between child sexual abuse and adult attachment. *Journal of Family Violence.* Advance online publication. [https://doi.org/10.1007/s10896-022-00456-9](https://psycnet.apa.org/doi/10.1007/s10896-022-00456-9)
148. Shin, M., & Brunton, R. (2024). Early life stress and mental health—Attentional bias, executive function and resilience as moderating and mediating factors. *Personality and Individual Differences, 221,* 1–9. [https://doi.org/10.1016/j.paid.2024.112565](https://psycnet.apa.org/doi/10.1016/j.paid.2024.112565)
149. Simeon, D., Yehuda, R., Cunill, R., Knutelska, M., Putnam, F. W., & Smith, L. M. (2007). Factors associated with resilience in healthy adults. *Psychoneuroendocrinology*, *32*(8-10), 1149–1152. <https://doi.org/10.1016/j.psyneuen.2007.08.005>
150. Simon, N. M., Herlands, N. N., Marks, E. H., Mancini, C., Letamendi, A., Li, Z., Pollack, M. H., Van Ameringen, M., & Stein, M. B. (2009). Childhood maltreatment linked to greater symptom severity and poorer quality of life and function in social anxiety disorder. *Depression and anxiety*, *26*(11), 1027–1032. <https://doi.org/10.1002/da.20604>
151. Sistad, R. E., Simons, R. M., Mojallal, M., & Simons, J. S. (2021). The indirect effect from childhood maltreatment to PTSD symptoms via thought suppression and cognitive reappraisal. *Child abuse & neglect*, *114*, 104939. <https://doi.org/10.1016/j.chiabu.2021.104939>
152. Soffer, N., Gilboa-Schechtman, E., & Shahar, G. (2008). The relationship of childhood emotional abuse and neglect to depressive vulnerability and low self-efficacy. *International Journal of Cognitive Therapy, 1*(2), 151–162. [https://doi.org/10.1521/ijct.2008.1.2.151](https://psycnet.apa.org/doi/10.1521/ijct.2008.1.2.151)
153. Șoflău, R., Szentágotai-Tătar, A. & Oltean, LE. (2024). Childhood Adversity, Resilience, and Paranoia During the COVID-19 Outbreak. The Mediating Role of Irrational Beliefs and Affective Disturbance. *Journal of Rational-Emotive & Cognitive-Behavior Therapy,* *42*, 263–278. <https://doi.org/10.1007/s10942-023-00511-4>
154. Somers, J. A., Ibrahim, M. H., & Luecken, L. J. (2017). Biological Sensitivity to the Effects of Childhood Family Adversity on Psychological Well-Being in Young Adulthood. *Child maltreatment*, *22*(3), 236–244. <https://doi.org/10.1177/1077559517711041>
155. Stevens, N. R., Gerhart, J., Goldsmith, R. E., Heath, N. M., Chesney, S. A., & Hobfoll, S. E. (2013). Emotion regulation difficulties, low social support, and interpersonal violence mediate the link between childhood abuse and posttraumatic stress symptoms. *Behavior therapy*, *44*(1), 152–161. <https://doi.org/10.1016/j.beth.2012.09.003>
156. Su, Y., Meng, X., Yang, G., & D'Arcy, C. (2022). The relationship between childhood maltreatment and mental health problems: coping strategies and social support act as mediators. *BMC psychiatry*, *22*(1), 359. <https://doi.org/10.1186/s12888-022-04001-2>
157. Sun, Y., Zhang, J., Yu, H., Zhou, Y., Jiang, W. & Jia, Y. (2023). Effect of Childhood Trauma on Aggressive Behavior in Stable Schizophrenia Patients: the Chain Mediating Effect of Self-esteem and Resilience. *Chinese General Practice, 26*(23): 2899-2906. [DOI: 10.12114/j.issn.1007-9572.2023.0030](https://doi.org/10.12114/j.issn.1007-9572.2023.0030).
158. Suresh, S. & Tipandjan, A. (2012). School bullying victimization and college adjustment. *Journal of the Indian Academy of Applied Psychology, 38* (1), 68-73. <https://doi.org/10.4135/36.753117>
159. Švecová, J., Furstova, J., Kaščáková, N., Hašto, J., & Tavel, P. (2023). The effect of childhood trauma and resilience on psychopathology in adulthood: Does bullying moderate the associations?. *BMC psychology*, *11*(1), 230. <https://doi.org/10.1186/s40359-023-01270-8>
160. Swaminath, S., Sistad, R. E., Simons, R. M., & Simons, J. S. (2023). The role of thought suppression and negative urgency in eating disorder symptoms and alcohol-related problems among survivors of childhood maltreatment. *Clinical Psychologist, 27*(3), 316–327. [https://doi.org/10.1080/13284207.2023.2221783](https://psycnet.apa.org/doi/10.1080/13284207.2023.2221783)
161. Talmon, A., Cohen, N., Raif, Y., & Ginzburg, K. (2022). Sense of mastery among older adults and its relation to invalidating childhood experiences. *Aging & mental health*, *26*(11), 2186–2194. <https://doi.org/10.1080/13607863.2021.1993130>
162. Tarber, D. N., Cohn, T. J., Casazza, S., Hastings, S. L., & Steele, J. (2016). The role of self-compassion in psychological well-being for male survivors of childhood maltreatment. *Mindfulness, 7*(5), 1193–1202. [https://doi.org/10.1007/s12671-016-0562-4](https://psycnet.apa.org/doi/10.1007/s12671-016-0562-4)
163. Theran, S. A., & Han, S. C. (2013). Authenticity as a mediator of the relation between child maltreatment and negative outcomes for college women. *Journal of Aggression, Maltreatment & Trauma, 22*(10), 1096–1116. [https://doi.org/10.1080/10926771.2013.845277](https://psycnet.apa.org/doi/10.1080/10926771.2013.845277)
164. Thoma, M. V., Bernays, F., Eising, C. M., Maercker, A., & Rohner, S. L. (2021). Child maltreatment, lifetime trauma, and mental health in Swiss older survivors of enforced child welfare practices: Investigating the mediating role of self-esteem and self-compassion. *Child abuse & neglect*, *113*, 104925. <https://doi.org/10.1016/j.chiabu.2020.104925>
165. Tinajero, R., Williams, P. G., Cribbet, M. R., Rau, H. K., Silver, M. A., Bride, D. L., & Suchy, Y. (2020). Reported history of childhood trauma and stress-related vulnerability: Associations with emotion regulation, executive functioning, daily hassles and pre-sleep arousal. *Stress and health : journal of the International Society for the Investigation of Stress*, *36*(4), 405–418. <https://doi.org/10.1002/smi.2938>
166. Toker, T., Tiryaki, A., Özçürümez, G., & Iskender, B. (2011). The relationship between traumatic childhood experiences and proclivities towards substance abuse, self-esteem and coping strategies. *Turkish journal of psychiatry*, *22*(2), 83–92.
167. Ustuner Top, F., & Cam, H. H. (2021). Childhood maltreatment among university students in Turkey: prevalence, demographic factors, and health-related quality of life consequences. *Psychology, health & medicine*, *26*(5), 543–554. <https://doi.org/10.1080/13548506.2020.1768274>
168. Upenieks, L., Kent, B. V., Nagaswami, M., Gu, Y., Kanaya, A. M., & Shields, A. E. (2024). Do Religion and Spirituality Buffer the Effect of Childhood Trauma on Depressive Symptoms? Examination of a South Asian Cohort from the USA. *Journal of religion and health*, *63*(4), 2998–3026. <https://doi.org/10.1007/s10943-024-02040-5>
169. Valencia, P. D., & De la Rosa-Gómez, A. (2024). Emotion regulation mediates the association between child abuse and adult suicidal ideation: an exploratory study. *Revista Colombiana de Psiquiatría*. Advance online publication: <https://doi.org/10.1016/j.rcp.2024.01.003>
170. van Schie, C., Gallagher, R., & Krause-Utz, A. (2024). Exploring the complex relationship between childhood trauma and self-harm. *Journal of Aggression, Maltreatment & Trauma, 33*(6), 685–703. [https://doi.org/10.1080/10926771.2024.2303525](https://psycnet.apa.org/doi/10.1080/10926771.2024.2303525)
171. Vancappel, A., Hingray, C., Reveillere, C., & El-Hage, W. (2024). Disentangling the Link Between Mindfulness and Dissociation in PTSD: The Mediating Role of Attention and Emotional Acceptance. *Journal of trauma & dissociation : the official journal of the International Society for the Study of Dissociation (ISSD)*, *25*(1), 30–44. <https://doi.org/10.1080/15299732.2023.2231907>
172. Vettese, L.C., Dyer, C.E., Li, W.L., Wekerle, C. (2011). Does Self-Compassion Mitigate the Association Between Childhood Maltreatment and Later Emotion Regulation Difficulties? A Preliminary Investigation. *International Journal of Mental Health and Addiction,  9*, 480–491. <https://doi.org/10.1007/s11469-011-9340-7>
173. Volgenau, K. M., Hokes, K. E., Hacker, N., & Adams, L. M. (2023). A Network Analysis Approach to Understanding the Relationship Between Childhood Trauma and Wellbeing Later in Life. *Child psychiatry and human development*, *54*(4), 1127–1140. <https://doi.org/10.1007/s10578-022-01321-y>
174. Wadji, D. L., Oe, M., Bartoli, E., Martin-Soelch, C., Pfaltz, M. C., & Langevin, R. (2023). How are experiences and acceptability of child maltreatment related to resilience and posttraumatic growth: a cross cultural study. *European journal of psychotraumatology*, *14*(2), 2264119. <https://doi.org/10.1080/20008066.2023.2264119>
175. Walker, H. E., Thomsen, K. N., Jamison, L. E., Wamser-Nanney, R., & Howell, K. H. (2023). The Role of Dimensions of Emotion Dysregulation Following Exposure to Maltreatment and Adult Victimization. *Journal of interpersonal violence*, *38*(7-8), 5661–5681. <https://doi.org/10.1177/08862605221127188>
176. Walsh, K., DiLillo, D., & Scalora, M. J. (2011). The cumulative impact of sexual revictimization on emotion regulation difficulties: an examination of female inmates. *Violence against women*, *17*(8), 1103–1118. <https://doi.org/10.1177/1077801211414165>
177. Wang, Z., Wang, X., Peng, Y., Liu, C., & He, J. (2022). Recalled childhood maltreatment and suicide risk in chinese college students: The mediating role of psychache and the moderating role of meaning in life. *Journal of Adult Development.* Advance online publication. [https://doi.org/10.1007/s10804-022-09422-7](https://psycnet.apa.org/doi/10.1007/s10804-022-09422-7)
178. Wang, Z., Qiu, H., & Han, W. (2023). Childhood abuse and depression among Chinese male prisoners: Self-esteem and coping style as mediators. *Social Behavior and Personality: An international journal, 51*(12), e12690. <https://doi.org/10.2224/sbp.12690>
179. Whittington D. (2023). Disorganized attachment in emerging adulthood: Measurement comparisons and relations to childhood maltreatment and emotion dysregulation. *Family process*, *63*(1), 348–363. <https://doi.org/10.1111/famp.12866>
180. Wind, T. W., & Silvern, L. (1994). Parenting and family stress as mediators of the long-term effects of child abuse. *Child abuse & neglect*, *18*(5), 439–453. <https://doi.org/10.1016/0145-2134(94)90029-9>
181. Wolff, S., Holl, J., Stopsack, M., Arens, E. A., Höcker, A., Staben, K. A., Hiller, P., Klein, M., Schäfer, I., Barnow, S., & CANSAS Study Group (2016). Does Emotion Dysregulation Mediate the Relationship between Early Maltreatment and Later Substance Dependence? Findings of the CANSAS Study. *European addiction research*, *22*(6), 292–300. <https://doi.org/10.1159/000447397>
182. Wong, N. E., Hagan, M. J., & Holley, S. R. (2024). Childhood Maltreatment and Suicidal Thoughts and Behaviors in Young Adults. *Crisis*, *45*(1), 26–32. <https://doi.org/10.1027/0227-5910/a000909>
183. Wu, C. Z., Zong, Z. Y., Huang, T. T., Yu, L. X., & Sun, Q. W. (2023). Childhood maltreatment influences suicidal behavior: Rumination mediates and regulatory emotional self-efficacy moderates. *Death studies*, *47*(7), 784–791. <https://doi.org/10.1080/07481187.2022.2132319>
184. Wu, Q., Cao, H., Lin, X., Zhou, N., & Chi, P. (2022). Child Maltreatment and Subjective Well-being in Chinese Emerging Adults: A Process Model Involving Self-esteem and Self-compassion. *Journal of interpersonal violence*, *37*(15-16), NP13685–NP13706. <https://doi.org/10.1177/0886260521993924>
185. Xiang, Y., Wang, W., & Guan, F. (2018). The Relationship Between Child Maltreatment and Dispositional Envy and the Mediating Effect of Self-Esteem and Social Support in Young Adults. *Frontiers in psychology*, *9*, 1054. <https://doi.org/10.3389/fpsyg.2018.01054>
186. Xiang, Y., Chen, Z., & Zhao, J. (2020). How childhood maltreatment impacts aggression from perspectives of social comparison and resilience framework theory. *Journal of Aggression, Maltreatment & Trauma, 29*(9), 1113–1124. [https://doi.org/10.1080/10926771.2019.1685046](https://psycnet.apa.org/doi/10.1080/10926771.2019.1685046)
187. Xiang, Y., Yuan, R., & Zhao, J. (2021). Childhood maltreatment and life satisfaction in adulthood: The mediating effect of emotional intelligence, positive affect and negative affect. *Journal of health psychology*, *26*(13), 2460–2469. <https://doi.org/10.1177/1359105320914381>
188. Xiao, Z., Obsuth, I., Meinck, F., & Murray, A. L. (2023). Latent profiles of childhood psychological maltreatment and their links to adult mental health in China and the UK. *Child and adolescent psychiatry and mental health*, *17*(1), 30. <https://doi.org/10.1186/s13034-023-00572-4>
189. Xie, Y., Shen, Y., & Wu, J. (2023). Cumulative childhood trauma and mobile phone addiction among chinese college students: role of self-esteem and self-concept clarity as serial mediators. *Current psychology (New Brunswick, N.J.)*, 1–9. Advance online publication. <https://doi.org/10.1007/s12144-023-04734-7>
190. Xu, B., Wei, S., Yin, X., Jin, X., Yan, S., & Jia, L. (2023). The relationship between childhood emotional neglect experience and depressive symptoms and prefrontal resting functional connections in college students: The mediating role of reappraisal strategy. *Frontiers in behavioral neuroscience*, *17*, 927389. <https://doi.org/10.3389/fnbeh.2023.927389>
191. Xu, W., & Zheng, S. (2022). Childhood emotional abuse and cyberbullying perpetration among Chinese university students: The chain mediating effects of self-esteem and problematic social media use. *Frontiers in psychology*, *13*, 1036128. <https://doi.org/10.3389/fpsyg.2022.1036128>
192. Yao, K., Chen, P., Zhou, H., Ruan, J., Chen, D., Yang, X., & Zhou, Y. (2023). The effect of childhood trauma on suicide risk: the chain mediating effects of resilience and mental distress. *BMC psychiatry*, *23*(1), 865. <https://doi.org/10.1186/s12888-023-05348-w>
193. Yaroslavsky, I., Bush, A. H., & France, C. M. (2022). Emotion regulation deficits mediate childhood sexual abuse effects on stress sensitization and depression outcomes. *Development and psychopathology*, *34*(1), 157–170. <https://doi.org/10.1017/S095457942000098X>
194. Yilmaz, F. B., & Satici, S. A. (2024). Childhood Maltreatment and Spiritual Well-Being: Intolerance of Uncertainty and Emotion Regulation as Mediators in Turkish Sample. *Journal of religion and health*, *63*(3), 2380–2396. <https://doi.org/10.1007/s10943-023-01965-7>
195. Yöyen, E., & Bozacı, U. (2023). Childhood traumas, emotional differences and resilience as predictives of interpersonal problems. *Kibris Türk Psikiyatri ve Psikoloji Dergisi, 5*(2), 129–139. <https://doi.org/10.35365/ctjpp.23.2.05>
196. Yöyen, E., & Çaylak, İ. A. (2023). Investigation of childhood traumas, emotion regulation processes and dissociation as predictives of social anxiety. *Kibris Türk Psikiyatri ve Psikoloji Dergisi, 5*(4), 303–312. <https://doi.org/10.35365/ctjpp.23.4.02>
197. Yrondi, A., Arbus, C., Bennabi, D., D'Amato, T., Bellivier, F., Bougerol, T., Camus, V., Courtet, P., Doumy, O., Genty, J. B., Holtzmann, J., Horn, M., Lancon, C., Leboyer, M., Llorca, P. M., Maruani, J., Moirand, R., Molière, F., Petrucci, J., Richieri, R., … El-Hage, W. (2021). Relationship between childhood physical abuse and clinical severity of treatment-resistant depression in a geriatric population. *PloS one*, *16*(4), e0250148. <https://doi.org/10.1371/journal.pone.0250148>
198. Yubero, S., de las Heras, M., Navarro, R., & Larrañaga, E. (2021). Relations among chronic bullying victimization, subjective well-being and resilience in university students: A preliminary study. *Current Psychology: A Journal for Diverse Perspectives on Diverse Psychological Issues.* Advance online publication. [https://doi.org/10.1007/s12144-021-01489-x](https://psycnet.apa.org/doi/10.1007/s12144-021-01489-x)
199. Zaorska, J., Kopera, M., Trucco, E. M., Suszek, H., Kobyliński, P., & Jakubczyk, A. (2020). Childhood Trauma, Emotion Regulation, and Pain in Individuals With Alcohol Use Disorder. *Frontiers in psychiatry*, *11*, 554150. <https://doi.org/10.3389/fpsyt.2020.554150>
200. Zhang, Q., Zhang, Q., Ran, G., & Liang, Y. (2024). Childhood abuse and depression in emerging adults: The mediating role of regulatory emotional self-efficacy and the moderating role of subjective social status. *Journal of Adult Development, 31*(3), 206–216. [https://doi.org/10.1007/s10804-023-09463-6](https://psycnet.apa.org/doi/10.1007/s10804-023-09463-6)
201. Zhou, H., Ruan, J., Xie, J., Wang, Y., & Yang, X*.* (2024)*.* The effect of childhood maltreatment on the prosocial behavior of Chinese university students: a chain mediation analysis. *Current Psychology* *43*, 20722–20731. <https://doi.org/10.1007/s12144-024-05846-4>
202. Zhou, J., Hu, T., Xue, S., Dong, Z., & Tang, W. (2024). The association of childhood trauma with suicidality in adult psychiatric patients: The mediating role of NSSI and the moderating role of self-esteem. *Journal of clinical psychology*, *80*(3), 664–677. <https://doi.org/10.1002/jclp.23646>
203. Zhou, G., & Li, Y. (2024). Effect of childhood maltreatment on internet addiction among college students: The mediators of social support and self-esteem. *Social Behavior and Personality: An International Journal, 52*(2), 1–9. [https://doi.org/10.2224/sbp.12889](https://psycnet.apa.org/doi/10.2224/sbp.12889)

# SA6. Full list of excluded studies with reasons

| **Excluded studies with reasons** | |
| --- | --- |
| **Authors** | **Reason** |
| Aas et al., 2021 | Wrong outcome |
| Agudelo Hernández et al., 2023 | No valid CM assessment |
| Antons et al., 2023 | Wrong design |
| Arslan, 2023 | Overlapping dataset \| Duplicate |
| Ashaba et al., 2022 | Wrong outcome |
| Ashy et al., 2020 | Wrong design |
| Badr et al., 2018 | No valid CM assessment |
| Bakouni et al., 2023 | Wrong design |
| V. Banyard et al., 2017 | Wrong population |
| V. L. Banyard et al., 2002 | Wrong outcome |
| Beduna, 2018 | Wrong publication type |
| Beduna & Perrone-McGovern, 2019 | Wrong design |
| Benner et al., 2023 | Wrong exposure |
| Berent et al., 2017 | Wrong exposure |
| Berent et al., 2020 | Wrong exposure |
| Berent et al., 2021 | Duplicate |
| Berglund et al., 2023 | Wrong outcome and wrong design |
| Berke et al., 2023 | Wrong design |
| Berzenski & Yates, 2010 | Duplicate |
| Bhattarai et al., 2023 | Wrong design |
| Biggam & Power, 1999 | Wrong exposure |
| Billen et al., 2023 | Duplicate |
| Binder et al., 1996 | Wrong outcome |
| Bishop et al., 2024 | Wrong exposure |
| Bogar & Hulse‐Killacky, 2006 | No valid CM assessment |
| Bouchard & Sonier, 2023 | Wrong outcome |
| Bremer-Hoeve et al., 2023 | Wrong design |
| Brockie et al., 2018 | Wrong outcome |
| Bruefach et al., 2023 | Wrong outcome |
| Brunton & Dryer, 2024 | Wrong design |
| Campbell-Sills et al., 2022 | Wrong outcome |
| Cărnuţă et al., 2015 | Wrong exposure |
| Carlson, 2011 | Wrong exposure |
| Carlton et al., 2021 | Wrong outcome |
| Carr et al., 2023 | Wrong exposure |
| Chakrapani et al., 2022 | Wrong exposure, outcome and population |
| Chang et al., 2023 | Duplicate |
| Charak et al., 2018 | Wrong design |
| Chau et al., 2023 | Wrong outcome |
| J. Chen et al., 2004 | Wrong design |
| Z. Chen et al., 2023 | Duplicate |
| F. Cheng et al., 2020 | Wrong outcome |
| F. Cheng et al., 2023 | Wrong population |
| P. Cheng & Langevin, 2023 | Overlapping dataset \| Duplicate |
| Cirillo, 2000 | Wrong publication type |
| Clarke et al., 2024 | Wrong exposure |
| Coelho & Sousa, 2021 | Wrong population |
| Collings, 1997 | Wrong design |
| Collishaw et al., 2007 | Wrong outcome |
| Colter, 2020 | Wrong publication type |
| Cortés & Justicia, 2008 | No valid CM assessment |
| Cortés Arboleda et al., 2011 | No valid CM assessment |
| Coyle et al., 2014 | No valid CM assessment |
| Daemen et al., 2023 | Wrong design |
| Daníelsdóttir et al., 2022 | Wrong exposure |
| Darawsheh, 2023 | No valid CM assessment |
| Das et al., 2011 | Wrong exposure |
| Debowska & Boduszek, 2017 | Wrong design |
| Dehghan Manshadi et al., 2024 | No valid resilience assessment |
| Delhalle & Blavier, 2023 | Wrong outcome |
| Demers et al., 2018 | Wrong outcome |
| Denny et al., 2004 | Wrong population |
| Dhungana et al., 2022 | Wrong design |
| Ding et al., 2019 | Wrong exposure |
| Dion et al., 2019 | Wrong population |
| Dixon et al., 2024 | Wrong exposure |
| Doba et al., 2022 | Wrong outcome |
| Dokuz et al., 2022 | Wrong outcome |
| Dong et al., 2024 | Wrong design |
| Dugal et al., 2018 | No valid CM assessment |
| Duprey et al., 2023 | Wrong population \| Duplicate |
| Easterbrooks et al., 2024 | Wrong exposure and design |
| Elton et al., 2023 | Wrong design |
| Fan et al., 2024 | Wrong design |
| Fava et al., 2024 | Wrong design |
| Fergusson et al., 2013 | Wrong design |
| Floyd, 2024 | Wrong exposure |
| Foster et al., 2023 | Wrong exposure |
| Freer et al., 2017 | Wrong exposure |
| Freitas et al., 2022 | Wrong population |
| Frick et al., 2021 | Wrong outcome |
| Fuller-Thomson et al., 2023 | No valid CM assessment \| Duplicate |
| Gathier et al., 2024 | Wrong design |
| Gross & Keller, 1992 | Wrong design |
| Gündoğar et al., 2014 | Wrong exposure \| Duplicate |
| Guo et al., 2021 | Wrong design |
| Gusler & Jackson, 2023 | Wrong exposure |
| Gwandure, 2007 | Wrong design |
| Haidl et al., 2021 | Wrong outcome |
| Ham-Rowbottom et al., 2005 | Wrong outcome |
| M. A. Harris et al., 2016 | Wrong exposure |
| L. S. Harris et al., 2016 | Wrong population |
| Q.-M. He et al., 2008 | Not retrieved and wrong design by title |
| J. He et al., 2023 | Duplicate |
| Hegelstad et al., 2021 | Wrong design |
| Heretick, 2012 | Wrong publication type |
| Heselton et al., 2022 | Wrong design |
| Hoell et al., 2023 | No valid CM assessment |
| Holdren, 2020 | Wrong publication type |
| Hu et al., 2021 | Wrong outcome |
| Huh et al., 2016 | Wrong design |
| Hunter, 1991 | Wrong design |
| Hyman & Williams, 2001 | No valid resilience assessment |
| Ibrahim et al., 2024 | Wrong exposure |
| Islam et al., 2022 | No valid CM assessment |
| Iwanaga et al., 2024 | No valid resilience assessment |
| Jenkins et al., 2022 | Wrong exposure |
| Jenness et al., 2021 | Wrong population |
| Jiang et al., 2010 | Not retrieved and wrong design by title |
| John, 2021 | Wrong publication type |
| John-Henderson et al., 2024 | Wrong exposure |
| Juárez-García et al., 2024 | Wrong design |
| Kabadayi & Sari, 2018 | Wrong population |
| Kahraman & Çankaya, 2020 | Wrong design |
| Kalia & Knauft, 2020 | Wrong exposure |
| Karaman, 2023 | Wrong population |
| Karska et al., 2024 | Wrong design |
| Kaščáková et al., 2022 | Wrong design |
| Katz & Gurtovenko, 2015 | Wrong exposure |
| Kelifa et al., 2021 | Wrong exposure |
| Kiefer et al., 2023 | Wrong outcome |
| M.-K. Kim et al., 2018 | Wrong design |
| Y. Kim et al., 2022 | Wrong population |
| J. Kim et al., 2015 | Wrong design |
| Kızıltepe et al., 2023 | Duplicate |
| Klanecky, 2011 | Wrong publication type |
| Kohrt et al., 2016 | Wrong exposure and no valid CM assessment |
| Kong, 2018 | Wrong design |
| Korkeila et al., 2004 | Wrong design |
| Kowalski et al., 2023 | Wrong exposure |
| Krakau et al., 2021 | Wrong outcome |
| Kronström et al., 2021 | Wrong exposure |
| Labella et al., 2024 | No valid CM assessment |
| Larsen et al., 2019 | Wrong outcome |
| Lassri et al., 2023 | Duplicate |
| H. Lee, 2021 | Wrong publication type |
| E. E. Lee et al., 2018 | Wrong design |
| Lehmann et al., 2014 | Wrong exposure and outcome |
| Lei et al., 2020 | Wrong outcome |
| Leitenberg et al., 2004 | Wrong exposure |
| Y. Li et al., 2023 | Wrong outcome |
| S. Li et al., 2023 | Wrong exposure |
| Y. Li & Liang, 2023 | Wrong exposure |
| D. Li et al., 2023 | Wrong design |
| C. Li et al., 2023 | Duplicate |
| Liem et al., 1997 | Wrong design |
| Logan-Greene et al., 2014 | Wrong outcome |
| LoSavio et al., 2021 | Wrong design |
| Lunding et al., 2023 | Wrong design |
| Lyle, 2006 | Wrong publication type |
| Mansour et al., 2013 | No valid resilience assessment |
| Marcy, 1998 | Wrong publication type |
| Mathur et al., 2018 | Wrong exposure |
| Matthews et al., 2017 | No valid CM assessment |
| McLafferty et al., 2021 | Wrong exposure |
| McNeal, 2020 | Wrong publication type |
| Meddeb et al., 2023 | Wrong exposure |
| Merians et al., 2024 | Wrong exposure |
| Mizutani & Amemiya, 2015 | Wrong design |
| Mohammadi et al., 2023 | Wrong publication type |
| Moon & Han, 2022 | Wrong outcome |
| Moore & Woodcock, 2017 | Wrong population |
| Morstead & DeLongis, 2023 | No valid CM assessment |
| Mullins & Panlilio, 2023 | Wrong population |
| Múzquiz et al., 2021 | Wrong population |
| Nam et al., 2016 | Wrong exposure |
| Newcomb et al., 2019 | No valid CM assessment |
| Nisu et al., 2023 | Wrong exposure |
| Nomura et al., 2006 | Wrong outcome |
| Oshri et al., 2017 | No valid CM assessment |
| Palagini et al., 2022 | Wrong exposure |
| Pardo, 2008 | Wrong publication type |
| Park et al., 2023 | Duplicate |
| Pelcovitz, 2015 | Wrong publication type |
| Pereda & Sicilia, 2017 | Wrong outcome |
| Petros et al., 2013 | Wrong exposure |
| Picci et al., 2023 | Wrong population |
| Polepally Ashok, 2017 | Wrong publication type |
| Proskynitopoulos et al., 2021 | Wrong outcome |
| Rajalin et al., 2020 | Wrong outcome |
| Ramos Salazar, 2021 | Wrong population |
| Reed, 2017 | Wrong publication type |
| Rhoden-Neita et al., 2024 | No valid CM assessment |
| Richter et al., 2019 | Wrong design |
| Ridder, 2018 | Wrong publication type |
| Roberts et al., 2004 | No valid CM assessment |
| Robinson et al., 2021 | Wrong outcome |
| Roche-Miranda et al., 2023 | Wrong design |
| Rogosch et al., 2010 | Wrong population |
| Rohner et al., 2022 | Wrong exposure |
| Romera et al., 2022 | Wrong population |
| Rompilla et al., 2023 | Wrong exposure and outcome |
| Rose et al., 2023 | Wrong exposure |
| Roy et al., 2011 | Wrong design |
| Russo et al., 2023a | Wrong design |
| Russo et al., 2023b | Wrong exposure |
| Saintil, 2017 | Wrong publication type |
| Sappington et al., 1997 | No valid CM assessment |
| Sassoon et al., 2023 | Wrong design |
| Sauceda et al., 2016 | Wrong design |
| Savani et al., 2023 | Wrong population |
| Schiele et al., 2020 | Wrong design |
| Schiele et al., 2016 | Wrong design |
| Schumm et al., 2006 | Wrong outcome |
| Shafiq & Batool, 2022 | Wrong population |
| Shaheen et al., 2023 | Wrong exposure |
| Sheffler et al., 2022 | Wrong outcome |
| Shen & Soloski, 2024 | Duplicate |
| Sheridan et al., 2020 | Wrong population |
| Shi et al., 2024 | Wrong design |
| G. C. Smith et al., 2023 | Wrong design |
| S. Smith & Chesin, 2023 | Wrong publication type |
| Snow et al., 2022 | Wrong design |
| Snyder et al., 2024 | Wrong exposure |
| Sölva et al., 2023 | Wrong publication type |
| Somefun et al., 2023 | Wrong population |
| Stanislawski et al., 2023 | Wrong design |
| Stanton et al., 2022 | Wrong exposure |
| Steensma & Van Dijke, 2006 | Wrong exposure |
| Stein et al., 2002 | No valid CM assessment |
| Strøm et al., 2018 | Wrong outcome |
| Su et al., 2023 | Wrong design |
| Sullivan et al., 2024 | Wrong outcome |
| Švecová et al., 2023 | Duplicate |
| Tanacioğlu Aydin & Pekşen Süslü, 2023 | Wrong exposure |
| Terock et al., 2019 | Wrong design |
| Theodora et al., 2023 | Wrong outcome and design |
| Thoma et al., 2020 | No valid CM assessment |
| Titelius et al., 2018 | Wrong population |
| Tonmyr et al., 2011 | Wrong publication type |
| Topitzes et al., 2010 | Wrong outcome |
| Tung et al., 2022 | Wrong exposure |
| Uchida et al., 2018 | Wrong outcome |
| Upenieks, 2021 | Wrong outcome |
| Upenieks & Ford-Robertson, 2023 | No valid CM assessment |
| Vaillancourt-Morel et al., 2021 | Wrong outcome |
| Van Der Werff et al., 2013 | Wrong outcome |
| Walker et al., 2021 | No valid CM assessment |
| Walker-Williams & Fouché, 2018 | Wrong outcome |
| S. S. Wang et al., 2024 | Wrong population, outcome and design \| Duplicate |
| W. Wang et al., 2023 | Wrong design |
| Y.-C. Wang et al., 2019 | Wrong exposure |
| W. Wang et al., 2023 | Wrong design |
| Warmingham et al., 2023 | Wrong exposure |
| Watt et al., 1995 | Wrong exposure |
| Weindl et al., 2020 | Wrong design |
| Wen et al., 2023 | Wrong design |
| Wild & Paivio, 2004 | Wrong exposure \| Duplicate |
| Wind & Silvern, 1992 | Wrong outcome |
| Wingo et al., 2010 | Wrong design |
| Wolf & Elklit, 2020 | No valid CM assessment |
| Wong & Chung, 2023 | Wrong design \| Duplicate |
| C.-Z. Wu et al., 2023 | Wrong publication type |
| Q. Wu et al., 2018 | Duplicate |
| Z. Xu et al., 2022 | Wrong exposure |
| J. Xu et al., 2024 | Wrong exposure |
| X. Xu et al., 2024 | Wrong exposure |
| B. Xu et al., 2023 | Wrong design |
| Yagci & Inaltekin, 2023 | Wrong design |
| Ye et al., 2024 | Wrong exposure |
| Yetim, 2022 | Wrong population |
| Young-Wolff et al., 2019 | Wrong exposure |
| Youssef et al., 2017 | Wrong exposure |
| Youssef et al., 2013 | Wrong design |
| Yu et al., 2022 | Wrong exposure and no valid resilience assessment |
| Yundt, 2019 | Wrong publication type |
| Zhou et al., 2022 | Wrong exposure |
| Zilioli et al., 2016 | Wrong exposure |

**References of excluded articles**

Aas, M., Ueland, T., Mørch, R. H., Laskemoen, J. F., Lunding, S. H., Reponen, E. J., Cattaneo, A., Agartz, I., Melle, I., Steen, N. E., & Andreassen, O. A. (2021). Physical activity and childhood trauma experiences in patients with schizophrenia or bipolar disorders. *The World Journal of Biological Psychiatry*, *22*(8), 637–645. https://doi.org/10.1080/15622975.2021.1907707

Agudelo Hernández, F., Benavides Bastidas, M., & Arango Gómez, F. (2023). Resilience, adverse childhood experiences, and mental health in Health Science students during the COVID-19 pandemic. *Salud mental*, *46*(2), 111–119. https://doi.org/10.17711/SM.0185-3325.2023.015

Antons, S., Büsche, K., Mallon, L., Wolf, O. T., Diers, M., & Brand, M. (2023). Stress Susceptibility, Affective Responses toward Acute Stressors and Emotion Regulation Strategies in the Context of Problematic Pornography Use. *Sexual Health & Compulsivity*, *30*(3), 231–251. https://doi.org/10.1080/26929953.2023.2220003

Arslan, G. (2023). Childhood psychological maltreatment, optimism, aversion to happiness, and psychological adjustment among college students. *Current Psychology*, *42*(29), 25142–25150. https://doi.org/10.1007/s12144-022-03538-5

Ashaba, S., Kakuhikire, B., Baguma, C., Satinsky, E. N., Perkins, J. M., Rasmussen, J. D., Cooper-Vince, C. E., Ahereza, P., Gumisiriza, P., Kananura, J., Bangsberg, D. R., & Tsai, A. C. (2022). Adverse childhood experiences, alcohol consumption, and the modifying role of social participation: Population-based study of adults in southwestern Uganda. *SSM - Mental Health*, *2*, 100062. https://doi.org/10.1016/j.ssmmh.2022.100062

Ashy, M., Yu, B., Gutowski, E., Samkavitz, A., & Malley-Morrison, K. (2020). Childhood Maltreatment, Limbic Dysfunction, Resilience, and Psychiatric Symptoms. *Journal of Interpersonal Violence*, *35*(1–2), 426–452. https://doi.org/10.1177/0886260516683174

Badr, H. E., Naser, J., Al-Zaabi, A., Al-Saeedi, A., Al-Munefi, K., Al-Houli, S., & Al-Rashidi, D. (2018). Childhood maltreatment: A predictor of mental health problems among adolescents and young adults. *Child Abuse & Neglect*, *80*, 161–171. https://doi.org/10.1016/j.chiabu.2018.03.011

Bakouni, H., Ouimet, M. C., Desjardins, S., Forget, H., & Vasiliadis, H.-M. (2023). Childhood abuse/neglect and temporal patterns in late-life anxiety. *Aging & Mental Health*, *27*(5), 973–982. https://doi.org/10.1080/13607863.2022.2076204

Banyard, V., Hamby, S., & Grych, J. (2017). Health effects of adverse childhood events: Identifying promising protective factors at the intersection of mental and physical well-being. *Child Abuse & Neglect*, *65*, 88–98. https://doi.org/10.1016/j.chiabu.2017.01.011

Banyard, V. L., Williams, L. M., Siegel, J. A., & West, C. M. (2002). Childhood Sexual Abuse in the Lives of Black Women: Risk and Resilience in a Longitudinal Study. *Women & Therapy*, *25*(3–4), 45–58. https://doi.org/10.1300/J015v25n03_04

Beduna, K. N. (2018). *Factors promoting shame resilience in adult survivors of childhood bullying*. Ball State University.

Beduna, K. N., & Perrone-McGovern, K. M. (2019). Recalled childhood bullying victimization and shame in adulthood: The influence of attachment security, self-compassion, and emotion regulation. *Traumatology*, *25*(1), 21–32. https://doi.org/10.1037/trm0000162

Benner, G. J., Strycker, L. A., Berry, L. A., Logan, A. J., & Lee, E. O. (2023). Associations between childhood trauma, perceived resilience, and teacher burnout. *Teachers and Teaching*, *29*(3), 291–309. https://doi.org/10.1080/13540602.2023.2173167

Berent, D., Emilien, G., Podgórski, M., Kusideł, E., Kulczycka-Wojdala, D., Szymańska, B., Macander, M., & Pawłowska, Z. (2017). SSTR4, childhood adversity, self-efficacy and suicide risk in alcoholics. *Translational Neuroscience*, *8*(1). https://doi.org/10.1515/tnsci-2017-0013

Berent, D., Szymańska, B., Kulczycka-Wojdala, D., Macander, M., Pawłowska, Z., & Wojnar, M. (2020). The role of childhood adversities, FKBP5, BDNF, NRN1, and generalized self-efficacy in suicide attempts in alcohol-dependent patients. *Pharmacological Reports*, *72*(3), 730–743. https://doi.org/10.1007/s43440-020-00080-8

Berent, D., Szymańska, B., Kulczycka-Wojdala, D., Macander, M., Pawłowska, Z., & Wojnar, M. (2021). Correction to: The role of childhood adversities, FKBP5, BDNF, NRN1, and generalized self-efficacy in suicide attempts in alcohol-dependent patients. *Pharmacological Reports*, *73*(5), 1482–1482. https://doi.org/10.1007/s43440-021-00277-5

Berglund, K. J., Alborn, S.-E., & Wennberg, P. (2023). Can a History of Childhood Trauma Impact the Effectiveness of Treatment in Individuals with Alcohol Use Disorders? A 2.5- and 5-Year Follow-up. *International Journal of Mental Health and Addiction*. https://doi.org/10.1007/s11469-023-01158-z

Berke, D. S., Moody, R. L., Grov, C., & Rendina, H. J. (2023). Psychosocial Risk Pathways from Childhood Sexual Abuse to Intimate Partner Violence among Sexual Minority Men: A Test of the Psychological Mediation Framework of Minority Stress. *Journal of Interpersonal Violence*, *38*(3–4), 3321–3343. https://doi.org/10.1177/08862605221106145

Berzenski, S. R., & Yates, T. M. (2010). A Developmental Process Analysis of the Contribution of Childhood Emotional Abuse to Relationship Violence. *Journal of Aggression, Maltreatment & Trauma*, *19*(2), 180–203. https://doi.org/10.1080/10926770903539474

Bhattarai, A., King, N., Adhikari, K., Dimitropoulos, G., Devoe, D., Byun, J., Li, M., Rivera, D., Cunningham, S., Bulloch, A. G. M., Patten, S. B., & Duffy, A. (2023). Childhood Adversity and Mental Health Outcomes Among University Students: A Longitudinal Study. *The Canadian Journal of Psychiatry*, *68*(7), 510–520. https://doi.org/10.1177/07067437221111368

Biggam, F. H., & Power, K. G. (1999). Social Problem-Solving Skills and Psychological Distress Among Incarcerated Young Offenders: The Issue of Bullying and Victimization. *Cognitive Therapy and Research*, *23*(3), 307–326. https://doi.org/10.1023/A:1018795632067

Billen, E., Garofalo, C., Schwabe, I., Jeandarme, I., & Bogaerts, S. (2023). Emotional, cognitive and behavioral self-regulation in forensic psychiatric patients: Changes over time and associations with childhood trauma, identity and personality pathology. *Psychology, Crime & Law*, *29*(10), 1080–1106. https://doi.org/10.1080/1068316X.2022.2044813

Binder, R. L., McNiel, D. E., & Goldstone, R. L. (1996). Is adaptive coping possible for adult survivors of childhood sexual abuse? *Psychiatric Services*, *47*(2), 186–188. https://doi.org/10.1176/ps.47.2.186

Bishop, J. D., VanDeusen, K. M., Sherwood, D. A., & Williams-Hecksel, C. (2024). Adverse Childhood Experiences, Trauma Exposure, and Stress Among MSW Students: Promoting Well-Being Through Perceived Adequacy of Self-Care. *Journal of Teaching in Social Work*, *44*(2), 171–186. https://doi.org/10.1080/08841233.2024.2317367

Bogar, C. B., & Hulse‐Killacky, D. (2006). Resiliency Determinants and Resiliency Processes Among Female Adult Survivors of Childhood Sexual Abuse. *Journal of Counseling & Development*, *84*(3), 318–327. https://doi.org/10.1002/j.1556-6678.2006.tb00411.x

Bouchard, G., & Sonier, N. A. (2023). Relationship between sibling bullying, family functioning, and problem solving: A structural equation modeling. *Current Psychology*, *42*(14), 11565–11574. https://doi.org/10.1007/s12144-021-02475-z

Bremer-Hoeve, S., Van Vliet, N. I., Van Bronswijk, S. C., Huntjens, R. J. C., De Jongh, A., & Van Dijk, M. K. (2023). Predictors of treatment dropout in patients with posttraumatic stress disorder due to childhood abuse1. *Frontiers in Psychiatry*, *14*, 1194669. https://doi.org/10.3389/fpsyt.2023.1194669

Brockie, T. N., Elm, J. H. L., & Walls, M. L. (2018). Examining protective and buffering associations between sociocultural factors and adverse childhood experiences among American Indian adults with type 2 diabetes: A quantitative, community-based participatory research approach. *BMJ Open*, *8*(9), e022265. https://doi.org/10.1136/bmjopen-2018-022265

Bruefach, T., Carr, D. C., & Sachs-Ericsson, N. (2023). History of childhood mistreatment and the psychological health consequences of COVID-19 for older adults. *Aging & Mental Health*, *27*(3), 563–571. https://doi.org/10.1080/13607863.2022.2084713

Brunton, R. J., & Dryer, R. (2024). Intimate Partner Violence Risk Factors: A Vulnerability-Adaptation Stress Model Approach. *Journal of Interpersonal Violence*, *39*(15–16), 3738–3763. https://doi.org/10.1177/08862605241234352

Campbell-Sills, L., Sun, X., Choi, K. W., He, F., Ursano, R. J., Kessler, R. C., Levey, D. F., Smoller, J. W., Gelernter, J., Jain, S., & Stein, M. B. (2022). Dissecting the heterogeneity of posttraumatic stress disorder: Differences in polygenic risk, stress exposures, and course of PTSD subtypes. *Psychological Medicine*, *52*(15), 3646. https://doi.org/10.1017/S0033291721000428

Carlson, B. E. (2011). Sibling Incest: Adjustment in Adult Women Survivors. *Families in Society: The Journal of Contemporary Social Services*, *92*(1), 77–83. https://doi.org/10.1606/1044-3894.4067

Carlton, C. N., Garcia, K. M., Sullivan-Toole, H., Stanton, K., McDonnell, C. G., & Richey, J. A. (2021). From childhood maltreatment to adult inflammation: Evidence for the mediational status of social anxiety and low positive affect. *Brain, Behavior, & Immunity - Health*, *18*, 100366. https://doi.org/10.1016/j.bbih.2021.100366

Cărnuţă, M., Crişan, L. G., Vulturar, R., Opre, A., & Miu, A. C. (2015). Emotional non-acceptance links early life stress and blunted cortisol reactivity to social threat. *Psychoneuroendocrinology*, *51*, 176–187. https://doi.org/10.1016/j.psyneuen.2014.09.026

Carr, M. M., Ellis, J. D., Saules, K. K., & Ledgerwood, D. M. (2023). Childhood Adversity, Addiction Severity, and Comorbidity Among Patients Receiving Opioid Use Disorder Treatment: The Role of Emotion Regulation. *Journal of Nervous & Mental Disease*, *211*(2), 150–156. https://doi.org/10.1097/NMD.0000000000001581

Chakrapani, V., Scheim, A. I., Newman, P. A., Shunmugam, M., Rawat, S., Baruah, D., Bhatter, A., Nelson, R., Jaya, A., & Kaur, M. (2022). Affirming and negotiating gender in family and social spaces: Stigma, mental health and resilience among transmasculine people in India. *Culture, Health & Sexuality*, *24*(7), 951–967. https://doi.org/10.1080/13691058.2021.1901991

Chang, Y.-H., Yang, M.-H., Yao, Z.-F., Tsai, M.-C., & Hsieh, S. (2023). The Mediating Role of Brain Structural Imaging Markers in Connecting Adverse Childhood Experiences and Psychological Resilience. *Children*, *10*(2), 365. https://doi.org/10.3390/children10020365

Charak, R., DiLillo, D., Messman-Moore, T. L., & Gratz, K. L. (2018). Latent classes of lifetime sexual victimization characteristics in women in emerging adulthood: Differential relations with emotion dysregulation. *Psychology of Violence*, *8*(5), 570–579. https://doi.org/10.1037/vio0000154

Chau, V., Dryer, R., & Brunton, R. (2023). Examining the relationship between maternal childhood abuse history and mother-infant bonding: The mediating roles of postpartum depression and maternal self-efficacy. *Child Abuse & Neglect*, *145*, 106439. https://doi.org/10.1016/j.chiabu.2023.106439

Chen, J., Han, P., & Dunne, M. P. (2004). [Child sexual abuse: A study among 892 female students of a medical school]. *Zhonghua Er Ke Za Zhi = Chinese Journal of Pediatrics*, *42*(1), 39–43.

Chen, Z., Shen, S., & Dai, Q. (2023). Long-term and short-term psycho-social predictors of early-adulthood depression: Role of childhood trauma, neuroticism, social-support, resilience, and life-events. *Current Psychology*, *42*(5), 3904–3916. https://doi.org/10.1007/s12144-021-01570-5

Cheng, F., Cui, S., Zhang, C., Zhang, L., Wang, L., Yuan, Q., Huang, C., Zhang, K., & Zhou, X. (2020). Association Between Cognitive Function and Early Life Experiences in Patients with Alcohol Use Disorder. *Frontiers in Psychiatry*, *11*, 792. https://doi.org/10.3389/fpsyt.2020.00792

Cheng, F., Shi, L., Wang, S., Jin, Q., Xie, H., Wang, B., & Zhang, W. (2023). The relationship between childhood traumatic experience and suicidal tendency in non-suicidal self-injury behavior patients. *BMC Psychiatry*, *23*(1), 401. https://doi.org/10.1186/s12888-023-04863-0

Cheng, P., & Langevin, R. (2023). Unpacking the effects of child maltreatment subtypes on emotional competence in emerging adults. *Psychological Trauma: Theory, Research, Practice, and Policy*, *15*(Suppl 1), S102–S111. https://doi.org/10.1037/tra0001322

Cirillo, I. (2000). *The relationship of constructive aggression to resilience in adults who were abused as children*. Smith College School for Social Work.

Clarke, S. D., Riser, D. K., & Schmidt, M. S. (2024). Electrocortical Correlates of Emotion Processing and Resilience in Individuals with Adverse Childhood Experiences. *Journal of Child & Adolescent Trauma*, *17*(3), 1–16. https://doi.org/10.1007/s40653-024-00621-w

Coelho, V. A., & Sousa, V. (2021). A Multilevel Analysis of the Relation Between Bullying Roles and Social and Emotional Competencies. *Journal of Interpersonal Violence*, *36*(11–12), 5122–5144. https://doi.org/10.1177/0886260518801943

Collings, S. J. (1997). Child sexual abuse in a sample of South African women students: Prevalence, characteristics, and long-term effects. *South African Journal of Psychology*, *27*(1), 37–42.

Collishaw, S., Pickles, A., Messer, J., Rutter, M., Shearer, C., & Maughan, B. (2007). Resilience to adult psychopathology following childhood maltreatment: Evidence from a community sample. *Child Abuse & Neglect*, *31*(3), 211–229. https://doi.org/10.1016/j.chiabu.2007.02.004

Colter, K. S. (2020). *The relationship between previous cybervictimization and current psychological well-being of College students*. Miami University.

Cortés Arboleda, M. R., Canton-Cortés, D., & Duarte, J. C. (2011). Consecuencias a largo plazo del abuso sexual infantil: Papel de la naturaleza y continuidad del abuso y del ambiente familiar. [Long term consequences of child sexual abuse: The role of the nature and continuity of abuse and family environment.]. *Behavioral Psychology*, *19*(1), 41–56.

Cortés, D. C., & Justicia, F. J. (2008). Afrontamiento del abuso sexual infantil y ajuste psicológico a largo plazo. [Child sexual abuse coping and long term psychological adjustment.]. *Psicothema*, *20*(4), 509–515.

Coyle, E., Karatzias, T., Summers, A., & Power, M. (2014). Emotions and emotion regulation in survivors of childhood sexual abuse: The importance of “disgust” in traumatic stress and psychopathology. *European Journal of Psychotraumatology*, *5*(1), 23306. https://doi.org/10.3402/ejpt.v5.23306

Daemen, M., Van Amelsvoort, T., GROUP investigators, & Reininghaus, U. (2023). Momentary Self-esteem as a Process Underlying the Association Between Childhood Trauma and Psychosis: Experience Sampling Study. *JMIR Mental Health*, *10*, e34147. https://doi.org/10.2196/34147

Daníelsdóttir, H. B., Aspelund, T., Thordardottir, E. B., Fall, K., Fang, F., Tómasson, G., Rúnarsdóttir, H., Yang, Q., Choi, K. W., Kennedy, B., Halldorsdottir, T., Lu, D., Song, H., Jakobsdóttir, J., Hauksdóttir, A., & Valdimarsdóttir, U. A. (2022). Adverse childhood experiences and resilience among adult women: A population-based study. *eLife*, *11*, e71770. https://doi.org/10.7554/eLife.71770

Darawsheh, N. A. A. (2023). The Impact of Cyber Bullying on the Psychological Well-being of University Students: A Study in Jordanian Universities,. *Information Sciences Letters*, *12*(8), 2757–2768. https://doi.org/10.18576/isl/120826

Das, D., Cherbuin, N., Tan, X., Anstey, K. J., & Easteal, S. (2011). DRD4-exonIII-VNTR Moderates the Effect of Childhood Adversities on Emotional Resilience in Young-Adults. *PLoS ONE*, *6*(5), e20177. https://doi.org/10.1371/journal.pone.0020177

Debowska, A., & Boduszek, D. (2017). Child abuse and neglect profiles and their psychosocial consequences in a large sample of incarcerated males. *Child Abuse & Neglect*, *65*, 266–277. https://doi.org/10.1016/j.chiabu.2016.12.003

Dehghan Manshadi, Z., Neshat-Doost, H. T., & Jobson, L. (2024). Cognitive factors as mediators of the relationship between childhood trauma and depression symptoms: The mediating roles of cognitive overgeneralisation, rumination, and social problem-solving. *European Journal of Psychotraumatology*, *15*(1), 2320041. https://doi.org/10.1080/20008066.2024.2320041

Delhalle, M., & Blavier, A. (2023). Le sentiment de compétence parentale des mères victimes d’abus sexuel(s) infantile(s): Un modèle structural. *Neuropsychiatrie de l’Enfance et de l’Adolescence*, *71*(5), 249–258. https://doi.org/10.1016/j.neurenf.2023.05.003

Demers, L. A., McKenzie, K. J., Hunt, R. H., Cicchetti, D., Cowell, R. A., Rogosch, F. A., Toth, S. L., & Thomas, K. M. (2018). Separable Effects of Childhood Maltreatment and Adult Adaptive Functioning on Amygdala Connectivity During Emotion Processing. *Biological Psychiatry: Cognitive Neuroscience and Neuroimaging*, *3*(2), 116–124. https://doi.org/10.1016/j.bpsc.2017.08.010

Denny, S., Clark, T. C., Fleming, T., & Wall, M. (2004). Emotional Resilience: Risk and Protective Factors for Depression Among Alternative Education Students in New Zealand. *American Journal of Orthopsychiatry*, *74*(2), 137–149. https://doi.org/10.1037/0002-9432.74.2.137

Dhungana, S., Koirala, R., Ojha, S. P., & Thapa, S. B. (2022). Association of childhood trauma, and resilience, with quality of life in patients seeking treatment at a psychiatry outpatient: A cross-sectional study from Nepal. *PLOS ONE*, *17*(10), e0275637. https://doi.org/10.1371/journal.pone.0275637

Ding, C., Wang, T., Chen, X., Li, J., Wang, W., Huang, D., Yan, H., & Li, S. (2019). Association of Adverse Childhood Experience and Attention Deficit Hyperactivity Disorder with depressive symptoms among men who have sex with men in China: Moderated mediation effect of resilience. *BMC Public Health*, *19*(1), 1706. https://doi.org/10.1186/s12889-019-8016-2

Dion, J., Gervais, J., Bigras, N., Blackburn, M.-E., & Godbout, N. (2019). A Longitudinal Study of the Mediating Role of Romantic Attachment in the Relation Between Child Maltreatment and Psychological Adaptation in Emerging Adults. *Journal of Youth and Adolescence*, *48*(12), 2391–2402. https://doi.org/10.1007/s10964-019-01073-4

Dixon, K. E., Bindbeutel, K. M., Daugherty, Y. T., Robertson, A. C., Lee, M., Galano, M. M., & Mahoney, C. T. (2024). The differential impact of childhood trauma on adult impulsivity and impulse control. *Traumatology*, *30*(3), 468–476. https://doi.org/10.1037/trm0000440

Doba, K., Saloppé, X., Choukri, F., & Nandrino, J.-L. (2022). Childhood trauma and posttraumatic stress symptoms in adolescents and young adults: The mediating role of mentalizing and emotion regulation strategies. *Child Abuse & Neglect*, *132*, 105815. https://doi.org/10.1016/j.chiabu.2022.105815

Dokuz, G., Kani, A. S., Uysal, Ö., & Kuşcu, M. K. (2022). The impact of childhood trauma and daily life experiences on emotional and psychotic symptom intensity in psychosis: An experience sampling study. *Psychiatry Research*, *317*, 114872. https://doi.org/10.1016/j.psychres.2022.114872

Dong, C., Jiang, H., Chen, X., Tao, J., Xu, Q., & Zhang, Y. (2024). Mental health associated with the cluster of childhood trauma and resilience among nursing students: A cross-sectional study. *Psychological Trauma: Theory, Research, Practice, and Policy*, *16*(Suppl 1), S224–S232. https://doi.org/10.1037/tra0001527

Dugal, C., Godbout, N., Bélanger, C., Hébert, M., & Goulet, M. (2018). Cumulative Childhood Maltreatment and Subsequent Psychological Violence in Intimate Relationships: The Role of Emotion Dysregulation. *Partner Abuse*, *9*(1), 18–40. https://doi.org/10.1891/1946-6560.9.1.18

Duprey, E. B., Handley, E. D., Russotti, J., Manly, J. T., & Cicchetti, D. (2023). A Longitudinal Examination of Child Maltreatment Dimensions, Emotion Regulation, and Comorbid Psychopathology. *Research on Child and Adolescent Psychopathology*, *51*(1), 71–85. https://doi.org/10.1007/s10802-022-00913-5

Easterbrooks, M. A., Katz, R. C., Stargel, L. E., & Rohrs, R. (2024). Parenting in the Context of Adversity: Investigating Buffering Effects of Positive Relationships and Community Connections. *Adversity and Resilience Science*, *5*(1), 11–25. https://doi.org/10.1007/s42844-022-00085-7

Elton, A., Allen, J. H., Yorke, M., Khan, F., Xu, P., & Boettiger, C. A. (2023). Sex moderates family history of alcohol use disorder and childhood maltreatment effects on an fMRI stop‐signal task. *Human Brain Mapping*, *44*(6), 2436–2450. https://doi.org/10.1002/hbm.26221

Fan, N., Fan, H., Luo, R., Wang, Y., Yan, Y., Yang, X., Wang, M., Dou, Y., Ni, R., Wei, J., Yang, W., & Ma, X. (2024). The impact of childhood trauma on emotional distress and the moderating role of sense of coherence among college students in China. *Scientific Reports*, *14*(1), 9797. https://doi.org/10.1038/s41598-024-60537-1

Fava, N. M., Coxe, S. J., Fortenberry, J. D., & Bay-Cheng, L. Y. (2024). Sexual Self-Concept After Child Maltreatment: The Role of Resilient Coping and Sexual Experience Among U.S. Young Adults. *Archives of Sexual Behavior*, *53*(1), 359–373. https://doi.org/10.1007/s10508-023-02706-3

Fergusson, D. M., McLeod, G. F. H., & Horwood, L. J. (2013). Childhood sexual abuse and adult developmental outcomes: Findings from a 30-year longitudinal study in New Zealand. *Child Abuse & Neglect*, *37*(9), 664–674. https://doi.org/10.1016/j.chiabu.2013.03.013

Floyd, K. (2024). Affectionate Communication Moderates the Effect of Adverse Childhood Experience on Mental Well-Being. *Western Journal of Communication*, *88*(3), 700–716. https://doi.org/10.1080/10570314.2023.2168505

Foster, J. C., Cohodes, E. M., Brieant, A. E., McCauley, S., Odriozola, P., Zacharek, S. J., Pierre, J. C., Hodges, H. R., Kribakaran, S., Haberman, J. T., Holt-Gosselin, B., & Gee, D. G. (2023). Associations Between Early-Life Stress Exposure and Internalizing Symptomatology During the COVID-19 Pandemic: Assessing the Role of Neurobehavioral Mediators. *Biological Psychiatry Global Open Science*, *3*(3), 362–373. https://doi.org/10.1016/j.bpsgos.2022.07.006

Freer, B. D., Sprang, G., Katz, D., Belle, C., & Sprang, K. (2017). The Impact of Child Abuse Potential on Adaptive Functioning: Early Identification of Risk. *Journal of Family Violence*, *32*(2), 189–196. https://doi.org/10.1007/s10896-016-9863-6

Freitas, D. F. D., Mendonça, M., Wolke, D., Marturano, E. M., Fontaine, A. M., & Coimbra, S. (2022). Resilience in the face of peer victimization and perceived discrimination: The role of individual and familial factors. *Child Abuse & Neglect*, *125*, 105492. https://doi.org/10.1016/j.chiabu.2022.105492

Frick, A., Thinnes, I., Hofmann, S. G., Windmann, S., & Stangier, U. (2021). Reduced Social Connectedness and Compassion Toward Close Others in Patients With Chronic Depression Compared to a Non-clinical Sample. *Frontiers in Psychiatry*, *12*, 608607. https://doi.org/10.3389/fpsyt.2021.608607

Fuller-Thomson, E., Ryan-Morissette, D., Attar-Schwartz, S., & Brennenstuhl, S. (2023). Achieving Optimal Mental Health Despite Exposure to Chronic Parental Domestic Violence: What Pathways are Associated with Resilience in Adulthood? *Journal of Family Violence*, *38*(4), 703–712. https://doi.org/10.1007/s10896-022-00390-w

Gathier, A. W., Van Tuijl, L. A., Penninx, B. W. J. H., De Jong, P. J., Van Oppen, P. C., Vinkers, C. H., & Verhoeven, J. E. (2024). The role of explicit and implicit self-esteem in the relationship between childhood trauma and adult depression and anxiety. *Journal of Affective Disorders*, *354*, 443–450. https://doi.org/10.1016/j.jad.2024.03.036

Gross, A. B., & Keller, H. R. (1992). Long-term consequences of childhood physical and psychological maltreatment. *Aggressive Behavior*, *18*(3), 171–185. https://doi.org/10.1002/1098-2337(1992)18:3<171::AID-AB2480180302>3.0.CO;2-I

Gündoğar, D., Kesebir, S., Demirkan, A. K., & Yaylacı, E. T. (2014). Is the relationship between affective temperament and resilience different in depression cases with and without childhood trauma? *Comprehensive Psychiatry*, *55*(4), 870–875. https://doi.org/10.1016/j.comppsych.2014.01.016

Guo, R., Sun, M., Zhang, C., Fan, Z., Liu, Z., & Tao, H. (2021). The Role of Military Training in Improving Psychological Resilience and Reducing Depression Among College Freshmen. *Frontiers in Psychiatry*, *12*, 641396. https://doi.org/10.3389/fpsyt.2021.641396

Gusler, S., & Jackson, Y. (2023). Adversity’s Impact on Adults’ Self-Report and Physiological Difficulties with Emotion Regulation: Appraisal as a Moderating Mechanism. *Journal of Aggression, Maltreatment & Trauma*, *32*(4), 611–630. https://doi.org/10.1080/10926771.2023.2179446

Gwandure, C. (2007). Sexual assault in childhood: Risk HIV and AIDS behaviours in adulthood. *AIDS Care*, *19*(10), 1313–1315. https://doi.org/10.1080/09540120701426508

Haidl, T. K., Gruen, M., Dizinger, J., Rosen, M., Doll, C. M., Penzel, N., Daum, L., Große Hokamp, N., Klosterkötter, J., Ruhrmann, S., Vogeley, K., Schultze-Lutter, F., & Kambeitz, J. (2021). Is there a diagnosis-specific influence of childhood trauma on later educational attainment? A machine learning analysis in a large help-seeking sample. *Journal of Psychiatric Research*, *138*, 591–597. https://doi.org/10.1016/j.jpsychires.2021.04.040

Ham-Rowbottom, K. A., Gordon, E. E., Jarvis, K. L., & Novaco, R. W. (2005). Life Constraints and Psychological Well-Being of Domestic Violence Shelter Graduates. *Journal of Family Violence*, *20*(2), 109–121. https://doi.org/10.1007/s10896-005-3174-7

Harris, L. S., Block, S. D., Ogle, C. M., Goodman, G. S., Augusti, E.-M., Larson, R. P., Culver, M. A., Pineda, A. R., Timmer, S. G., & Urquiza, A. (2016). Coping style and memory specificity in adolescents and adults with histories of child sexual abuse. *Memory*, *24*(8), 1078–1090. https://doi.org/10.1080/09658211.2015.1068812

Harris, M. A., Brett, C. E., Starr, J. M., Deary, I. J., & McIntosh, A. M. (2016). Early-life predictors of resilience and related outcomes up to 66 years later in the 6-day sample of the 1947 Scottish mental survey. *Social Psychiatry and Psychiatric Epidemiology*, *51*(5), 659–668. https://doi.org/10.1007/s00127-016-1189-4

He, J., Wang, R., Liu, J., & Yip, P. (2023). Effects of Adverse Childhood Experiences on Sleep Quality in People With Drug Addiction and Non-Addicts and the Role of Resilience Between Them. *Psychological Reports*, *126*(5), 2158–2171. https://doi.org/10.1177/00332941221076776

He, Q.-M., Pan, R.-D., & Meng, X.-Z. (2008). Relationship of social anxiety disorder and child abuse and trauma. *Chinese Journal of Clinical Psychology*, *16*(1), 40–42.

Hegelstad, W. T. V., Berg, A. O., Bjornestad, J., Gismervik, K., Johannessen, J. O., Melle, I., Stain, H. J., & Joa, I. (2021). Childhood interpersonal trauma and premorbid social adjustment as predictors of symptom remission in first episode psychosis. *Schizophrenia Research*, *232*, 87–94. https://doi.org/10.1016/j.schres.2021.05.015

Heretick, J. A. (2012). *The relationship between type of bullying experienced in childhood and psychosocial functioning in young adulthood*. University of Florida.

Heselton, G. A., Rempel, G. R., & Nicholas, D. B. (2022). “Realizing the problem wasn’t necessarily me”: The meaning of childhood adversity and resilience in the lives of autistic adults. *International Journal of Qualitative Studies on Health and Well-Being*, *17*(1), 2051237. https://doi.org/10.1080/17482631.2022.2051237

Hoell, A., Kourmpeli, E., Dölling, D., Horten, B., Meyer-Lindenberg, A., & Dreßing, H. (2023). Ein Schritt ins Dunkle: Pilotstudie zu Prävalenz, situativem Kontext und Folgen sexualisierter Gewalt gegen Kinder und Jugendliche in Deutschland. *Psychiatrische Praxis*, *50*(04), 182–188. https://doi.org/10.1055/a-1960-4795

Holdren, A. (2020). *Examining the Impact of Adolescent Cyberbullying on Early Adulthood Self-Esteem and Self-Efficacy*. Palo Alto University.

Hu, W., Liu, Y., Li, J., Zhao, X., & Yang, J. (2021). Early life stress moderated the influence of reward anticipation on acute psychosocial stress responses. *Psychophysiology*, *58*(10), e13892. https://doi.org/10.1111/psyp.13892

Huh, H. J., Baek, K., Kwon, J.-H., Jeong, J., & Chae, J.-H. (2016). Impact of childhood trauma and cognitive emotion regulation strategies on risk-aversive and loss-aversive patterns of decision-making in patients with depression. *Cognitive Neuropsychiatry*, *21*(6), 447–461. https://doi.org/10.1080/13546805.2016.1230053

Hunter, J. A. (1991). A Comparison of the Psychosocial Maladjustment of Adult Males and Females Sexually Molested as Children. *Journal of Interpersonal Violence*, *6*(2), 205–217. https://doi.org/10.1177/088626091006002005

Hyman, B., & Williams, L. (2001). Resilience Among Women Survivors of Child Sexual Abuse. *Affilia*, *16*(2), 198–219. https://doi.org/10.1177/08861090122094226

Ibrahim, F. M., Rashad Dabou, E. A., AbdelSamad, S., & Abuijlan, I. A. M. (2024). Prevalence of bullying and its impact on self-esteem, anxiety and depression among medical and health sciences university students in RAS Al Khaimah, UAE. *Heliyon*, *10*(3), e25063. https://doi.org/10.1016/j.heliyon.2024.e25063

Islam, M. J., Broidy, L., Eriksson, L., Rahman, M., & Mazumder, N. (2022). Childhood maltreatment and decision-making autonomy in adulthood: The mediating roles of self-esteem and social support. *Child Abuse & Neglect*, *129*, 105665. https://doi.org/10.1016/j.chiabu.2022.105665

Iwanaga, M., Nishi, D., Obikane, E., & Kawakami, N. (2024). Age of victimization and moderating role of social support for the relationship between school-age bullying and life satisfaction in middle-age. *Scandinavian Journal of Public Health*, *52*(2), 225–233. https://doi.org/10.1177/14034948221148788

Jenkins, L. N., McNeal, T., Eftaxas, D., Howell, J., & Wang, Q. (2022). Childhood Trauma and College Sexual Harassment: Coping and Resilience as Moderators. *Journal of Child & Adolescent Trauma*, *15*(2), 461–469. https://doi.org/10.1007/s40653-021-00382-w

Jenness, J. L., Peverill, M., Miller, A. B., Heleniak, C., Robertson, M. M., Sambrook, K. A., Sheridan, M. A., & McLaughlin, K. A. (2021). Alterations in neural circuits underlying emotion regulation following child maltreatment: A mechanism underlying trauma-related psychopathology. *Psychological Medicine*, *51*(11), 1880–1889. https://doi.org/10.1017/S0033291720000641

Jiang, H.-J., Deng, Y.-L., Pan, C., & Huang, L. (2010). Childhood psychological abuse and neglect to vocational college freshmen mental health: Mediating roles of self-esteem and neuroticism. *Chinese Mental Health Journal*, *24*(9), 705–710.

John, K. L. (2021). *Resilience and intimacy for adult lesbian survivors of child sexual abuse*. Walden University.

John-Henderson, N. A., Counts, C. J., Strong, N. C. B., Larsen, J. M., & Jeffs, M. (2024). Investigating the role of emotion regulation in the relationship between childhood trauma and alcohol problems in American Indian adults. *Journal of Affective Disorders*, *344*, 440–445. https://doi.org/10.1016/j.jad.2023.10.043

Juárez-García, D. M., Téllez, A., Sánchez-Jáuregui, T., Almaraz Castruita, D. A., García-Fuentes, K., & Valdez, A. (2024). Influence of Adverse Childhood Experiences and Resilience on Symptoms of Anxiety and Depression in Mexican Psychology Students. *Journal of Aggression, Maltreatment & Trauma*, *33*(8), 938–954. https://doi.org/10.1080/10926771.2024.2342507

Kabadayi, F., & Sari, S. V. (2018). What is the Role of Resilience in Predicting Cyber Bullying Perpetrators and Their Victims? *Journal of Psychologists and Counsellors in Schools*, *28*(1), 102–117. https://doi.org/10.1017/jgc.2017.20

Kahraman, B., & Çankaya, P. (2020). Childhood traumas, difficulty in emotion regulation and coping strategies in adult patients with nonsuicidal self-injury. *Anatolian Journal of Psychiatry*, *20*(0), 1. https://doi.org/10.5455/apd.70047

Kalia, V., & Knauft, K. (2020). Emotion regulation strategies modulate the effect of adverse childhood experiences on perceived chronic stress with implications for cognitive flexibility. *PLOS ONE*, *15*(6), e0235412. https://doi.org/10.1371/journal.pone.0235412

Karaman, M. A. (2023). Comparison of levels of bullying, achievement motivation, and resilience among Syrian refugee students and Turkish students. *Journal of Psychologists and Counsellors in Schools*, *33*(1), 62–77. https://doi.org/10.1017/jgc.2021.22

Karska, J., Rejek, M., & Misiak, B. (2024). Resilience buffers the association between psychotic-like experiences and suicide risk: A prospective study from a non-clinical sample. *BMC Psychiatry*, *24*(1), 32. https://doi.org/10.1186/s12888-024-05491-y

Kaščáková, N., Petríková, M., Hašto, J., & Tavel, P. (2022). Long-term anxiety and clinically diagnosed disorders: Associations with childhood trauma and insecure attachment in adulthood. *Ceska a Slovenska Psychiatrie*, *118*(5), 189–197.

Katz, L. F., & Gurtovenko, K. (2015). Posttraumatic stress and emotion regulation in survivors of intimate partner violence. *Journal of Family Psychology*, *29*(4), 528–536. https://doi.org/10.1037/fam0000128

Kelifa, M. O., Yang, Y., Carly, H., Bo, W., & Wang, P. (2021). How Adverse Childhood Experiences Relate to Subjective Wellbeing in College Students: The Role of Resilience and Depression. *Journal of Happiness Studies*, *22*(5), 2103–2123. https://doi.org/10.1007/s10902-020-00308-7

Kiefer, R., Goncharenko, S., Forkus, S. R., Contractor, A. A., LeBlanc, N., & Weiss, N. H. (2023). Role of positive emotion regulation strategies in the association between childhood trauma and posttraumatic stress disorder among trauma-exposed individuals who use substances. *Anxiety, Stress, & Coping*, *36*(3), 366–381. https://doi.org/10.1080/10615806.2022.2079636

Kim, J., Seok, J.-H., Choi, K., Jon, D.-I., Hong, H. J., Hong, N., & Lee, E. (2015). The Protective Role of Resilience in Attenuating Emotional Distress and Aggression Associated with Early-life Stress in Young Enlisted Military Service Candidates. *Journal of Korean Medical Science*, *30*(11), 1667. https://doi.org/10.3346/jkms.2015.30.11.1667

Kim, M.-K., Kim, J.-S., Park, H.-I., Choi, S.-W., Oh, W.-J., & Seok, J.-H. (2018). Early life stress, resilience and emotional dysregulation in major depressive disorder with comorbid borderline personality disorder. *Journal of Affective Disorders*, *236*, 113–119. https://doi.org/10.1016/j.jad.2018.04.119

Kim, Y., Lee, H., & Park, A. (2022). Patterns of adverse childhood experiences and depressive symptoms: Self-esteem as a mediating mechanism. *Social Psychiatry and Psychiatric Epidemiology*, *57*(2), 331–341. https://doi.org/10.1007/s00127-021-02129-2

Kızıltepe, R., Ebeoğlu-Duman, M., Sağel-Çetiner, E., & Hecker, T. (2023). The unique contribution of childhood maltreatment types to risk-taking behavior and self-esteem. *Current Psychology*, *42*(10), 8478–8488. https://doi.org/10.1007/s12144-023-04300-1

Klanecky, A. K. (2011). *Child/Adolescent Sexual Abuse and Alcohol: Proposed Pathways to Problematic Drinking in College via PTSD Symptoms, Emotion Dysregulation, and Dissociative Tendencies*. University of Nebraska - Lincoln.

Kohrt, B. A., Worthman, C. M., Adhikari, R. P., Luitel, N. P., Arevalo, J. M. G., Ma, J., McCreath, H., Seeman, T. E., Crimmins, E. M., & Cole, S. W. (2016). Psychological resilience and the gene regulatory impact of posttraumatic stress in Nepali child soldiers. *Proceedings of the National Academy of Sciences*, *113*(29), 8156–8161. https://doi.org/10.1073/pnas.1601301113

Kong, J. (2018). Childhood Maltreatment and Psychological Well-Being in Later Life: The Mediating Effect of Contemporary Relationships with the Abusive Parent. *The Journals of Gerontology: Series B*, *73*(5), e39–e48. https://doi.org/10.1093/geronb/gbx039

Korkeila, K., Kivel�, S.-L., Suominen, S., Vahtera, J., Kivim�ki, M., Sundell, J., Helenius, H., & Koskenvuo, M. (2004). Childhood adversities, parent-child relationships and dispositional optimism in adulthood. *Social Psychiatry and Psychiatric Epidemiology*, *39*(4), 286–292. https://doi.org/10.1007/s00127-004-0740-x

Kowalski, R. M., Giumetti, G. W., & Feinn, R. S. (2023). Is Cyberbullying An Extension of Traditional Bullying or a Unique Phenomenon? A Longitudinal Investigation Among College Students. *International Journal of Bullying Prevention*, *5*(3), 227–244. https://doi.org/10.1007/s42380-022-00154-6

Krakau, L., Tibubos, A. N., Beutel, M. E., Ehrenthal, J. C., Gieler, U., & Brähler, E. (2021). Personality functioning as a mediator of adult mental health following child maltreatment. *Journal of Affective Disorders*, *291*, 126–134. https://doi.org/10.1016/j.jad.2021.05.006

Kronström, K., Multimäki, P., Ristkari, T., Parkkola, K., Sillanmäki, L., & Sourander, A. (2021). Ten-year changes in the psychosocial well-being, psychopathology, substance use, suicidality, bullying, and sense of coherence of 18-year-old males: A Finnish population-based time-trend study. *European Child & Adolescent Psychiatry*, *30*(2), 313–325. https://doi.org/10.1007/s00787-020-01517-4

Labella, M. H., Klein, N. D., Yeboah, G., Bailey, C., Doane, A. N., Kaminer, D., Bravo, A. J., & Cross‐Cultural Addictions Study Team. (2024). Childhood bullying victimization, emotion regulation, rumination, distress tolerance, and depressive symptoms: A cross‐national examination among young adults in seven countries. *Aggressive Behavior*, *50*(1), e22111. https://doi.org/10.1002/ab.22111

Larsen, E. M., Ospina, L. H., Cuesta-Diaz, A., Vian-Lains, A., Nitzburg, G. C., Mulaimovic, S., Latifoglu, A., Clari, R., & Burdick, K. E. (2019). Effects of childhood trauma on adult moral decision-making: Clinical correlates and insights from bipolar disorder. *Journal of Affective Disorders*, *244*, 180–186. https://doi.org/10.1016/j.jad.2018.10.002

Lassri, D., Bregman-Hai, N., Soffer-Dudek, N., & Shahar, G. (2023). The Interplay Between Childhood Sexual Abuse, Self-Concept Clarity, and Dissociation: A Resilience-Based Perspective. *Journal of Interpersonal Violence*, *38*(3–4), 2313–2336. https://doi.org/10.1177/08862605221101182

Lee, E. E., Martin, A. S., Tu, X., Palmer, B. W., & Jeste, D. V. (2018). Childhood Adversity and Schizophrenia: The Protective Role of Resilience in Mental and Physical Health and Metabolic Markers. *The Journal of Clinical Psychiatry*, *79*(3). https://doi.org/10.4088/JCP.17m11776

Lee, H. (2021). *Is emotion regulation the key to breaking the cycle of violence? The roles of emotion regulation in buffering the effects of a childhood history of maltreatment on intimate partner violence in emerging adulthood*. University of Pittsburgh.

Lehmann, R. J. B., Goodwill, A. M., Hanson, R. K., & Dahle, K.-P. (2014). Crime Scene Behaviors Indicate Risk-Relevant Propensities of Child Molesters. *Criminal Justice and Behavior*, *41*(8), 1008–1028. https://doi.org/10.1177/0093854814521807

Lei, M.-K., Berg, M. T., Simons, R. L., Simons, L. G., & Beach, S. R. H. (2020). Childhood adversity and cardiovascular disease risk: An appraisal of recall methods with a focus on stress-buffering processes in childhood and adulthood. *Social Science & Medicine*, *246*, 112794. https://doi.org/10.1016/j.socscimed.2020.112794

Leitenberg, H., Gibson, L. E., & Novy, P. L. (2004). Individual differences among undergraduate women in methods of coping with stressful events: The impact of cumulative childhood stressors and abuse. *Child Abuse & Neglect*, *28*(2), 181–192. https://doi.org/10.1016/j.chiabu.2003.08.005

Li, C., Lv, G., Liu, B., Ju, Y., Wang, M., Dong, Q., Sun, J., Lu, X., Zhang, L., Wan, P., Guo, H., Zhao, F., Liao, M., Zhang, Y., Li, L., & Liu, J. (2023). Impact of childhood maltreatment on adult resilience. *BMC Psychiatry*, *23*(1), 637. https://doi.org/10.1186/s12888-023-05124-w

Li, D., Luo, J., Yan, X., & Liang, Y. (2023). Complex Posttraumatic Stress Disorder (CPTSD) as an Independent Diagnosis: Differences in Hedonic and Eudaimonic Well-Being between CPTSD and PTSD. *Healthcare*, *11*(8), 1188. https://doi.org/10.3390/healthcare11081188

Li, S., Yin, Y., Cui, G., Zhang, C., Zhu, H., & Yao, Y. (2023). The mediating and moderating effects of resilience between childhood trauma and geriatric depressive symptoms among Chinese community-dwelling older adults. *Frontiers in Public Health*, *11*, 1137600. https://doi.org/10.3389/fpubh.2023.1137600

Li, Y., & Liang, Y. (2023). The effect of childhood trauma on complex posttraumatic stress disorder: The role of self-esteem. *European Journal of Psychotraumatology*, *14*(2), 2272478. https://doi.org/10.1080/20008066.2023.2272478

Li, Y., Yuan, M., Chang, J., Zhang, T., Chen, L., Xie, G., Chen, S., He, Y., & Su, P. (2023). Association of Child Sexual Abuse Victimization and Murderous Behaviors and the Mediating Role of Psychological Adjustment among College Students in China. *Journal of Interpersonal Violence*, *38*(1–2), 1842–1867. https://doi.org/10.1177/08862605221093684

Liem, J. H., James, J. B., O’Toole, J. G., & Boudewyn, A. C. (1997). Assessing resilience in adults with histories of childhood sexual abuse. *American Journal of Orthopsychiatry*, *67*(4), 594–606. https://doi.org/10.1037/h0080257

Logan-Greene, P., Green, S., Nurius, P. S., & Longhi, D. (2014). Distinct Contributions of Adverse Childhood Experiences and Resilience Resources: A Cohort Analysis of Adult Physical and Mental Health. *Social Work in Health Care*, *53*(8), 776–797. https://doi.org/10.1080/00981389.2014.944251

LoSavio, S. T., Hale, W. J., Moring, J. C., Blankenship, A. E., Dondanville, K. A., Wachen, J. S., Mintz, J., Peterson, A. L., Litz, B. T., Young-McCaughan, S., Yarvis, J. S., & Resick, P. A. (2021). Efficacy of individual and group cognitive processing therapy for military personnel with and without child abuse histories. *Journal of Consulting and Clinical Psychology*, *89*(5), 476–482. https://doi.org/10.1037/ccp0000641

Lunding, S. H., Ueland, T., Aas, M., Høegh, M. C., Werner, M. C. F., Rødevand, L., Johansen, I. T., Hjell, G., Ormerod, M. B. E. G., Ringen, P. A., Ottesen, A., Lagerberg, T. V., Melle, I., Andreassen, O. A., Simonsen, C., & Steen, N. E. (2023). Tobacco smoking related to childhood trauma mediated by cognitive control and impulsiveness in severe mental disorders. *Schizophrenia Research*, *261*, 236–244. https://doi.org/10.1016/j.schres.2023.09.041

Lyle, P. N. (2006). *Adult males with childhood sexual experiences: The role of attachment and coping in psychological adjustment*. Auburn University.

Mansour, K., Roshdy, E., Langdon, P. E., Daoud, O. A., El-Saadawy, M., Al-Zahrani, A., & Khashaba, A. (2013). Child abuse and its long-term consequences: An exploratory study on Egyptian university students. *The International Psychiatry and Behavioral Neurosciences Yearbook*, *2*, 177–197.

Marcy, S. N. (1998). *Childhood maltreatment and negative sequelae in young adults: A comparison between outcomes of psychological, physical, and sexual maltreatment*. Boston University.

Mathur, A., Graham-Engeland, J. E., Slavish, D. C., Smyth, J. M., Lipton, R. B., Katz, M. J., & Sliwinski, M. J. (2018). Recalled early life adversity and pain: The role of mood, sleep, optimism, and control. *Journal of Behavioral Medicine*, *41*(4), 504–515. https://doi.org/10.1007/s10865-018-9917-8

Matthews, K. A., Jennings, J. R., Lee, L., & Pardini, D. A. (2017). Bullying and Being Bullied in Childhood Are Associated With Different Psychosocial Risk Factors for Poor Physical Health in Men. *Psychological Science*, *28*(6), 808–821. https://doi.org/10.1177/0956797617697700

McLafferty, M., McGlinchey, E., Travers, A., & Armour, C. (2021). The mediating role of resilience on psychopathology following childhood adversities among UK armed forces veterans residing in Northern Ireland. *European Journal of Psychotraumatology*, *12*(1), 1978176. https://doi.org/10.1080/20008198.2021.1978176

McNeal, T. A. (2020). *Relations between childhood maltreatment and adult psychological well-being: The influence of age at maltreatment onset*. Florida State University.

Meddeb, A., Garofalo, C., Karlén, M. H., & Wallinius, M. (2023). Emotion dysregulation – A bridge between ACE and aggressive antisocial behavior. *Journal of Criminal Justice*, *88*, 102110. https://doi.org/10.1016/j.jcrimjus.2023.102110

Merians, A. N., Mischel, E., Frazier, P., & Lust, K. (2024). Relationships between childhood adversity and life functioning in US college students: Risk and resilience. *Journal of American College Health*, *72*(1), 71–81. https://doi.org/10.1080/07448481.2021.2024205

Mizutani, S., & Amemiya, T. (2015). Impact of Having Been Bullied in Elementary, Junior High, and High School on University Students’ Self-Esteem and Well-Being. *The Japanese Journal of Educational Psychology*, *63*(2), 102–110. https://doi.org/10.5926/jjep.63.102

Mohammadi, F., Oshvandi, K., Shamsaei, F., Khodaveisi, M., Khazaei, S., & Masoumi, S. Z. (2023). Correction to: Child exposure to domestic violence, substance dependence and suicide resilience in child laborers. *BMC Public Health*, *23*(1), 1842. https://doi.org/10.1186/s12889-023-16175-9

Moon, I., & Han, J. (2022). Moderating Effects of Physical Activity on the Relationship between Adverse Childhood Experiences and Health-Related Quality of Life. *International Journal of Environmental Research and Public Health*, *19*(2), 668. https://doi.org/10.3390/ijerph19020668

Moore, B., & Woodcock, S. (2017). RESILIENCE, BULLYING, AND MENTAL HEALTH: FACTORS ASSOCIATED WITH IMPROVED OUTCOMES: Resilience, Bullying, and Mental Health. *Psychology in the Schools*, *54*(7), 689–702. https://doi.org/10.1002/pits.22028

Morstead, T., & DeLongis, A. (2023). Searching for secrets, searching for self: Childhood adversity, self‐concept clarity, and the motivation to uncover family secrets through direct‐to‐consumer genetic testing. *Journal of Genetic Counseling*, *32*(3), 698–705. https://doi.org/10.1002/jgc4.1679

Mullins, C., & Panlilio, C. C. (2023). Adversity, engagement, and later achievement: The role of emotion regulation and parent-child relationship quality. *Children and Youth Services Review*, *148*, 106862. https://doi.org/10.1016/j.childyouth.2023.106862

Múzquiz, J., Perez-García, A. M., & Bermúdez, J. (2021). Autoestima, autocompasión y afecto positivo y negativo en víctimas y agresores de bullying: Estudio comparativo con medidas autoinformadas e informadas por pares. *Revista de Psicopatología y Psicología Clínica*, *26*(1), 23. https://doi.org/10.5944/rppc.28156

Nam, B., Kim, J. Y., DeVylder, J. E., & Song, A. (2016). Family functioning, resilience, and depression among North Korean refugees. *Psychiatry Research*, *245*, 451–457. https://doi.org/10.1016/j.psychres.2016.08.063

Newcomb, M., Burton, J., & Edwards, N. (2019). Student Constructions of Resilience: Understanding the Role of Childhood Adversity. *Australian Social Work*, *72*(2), 166–178. https://doi.org/10.1080/0312407X.2018.1550521

Nisu, S., Anuroj, K., Wannaiampikul, S., Pangsorn, A., Chiencharoenthanakij, R., & Chongbanyatcharoen, S. (2023). Moderation Effects of Positive Core Belief and Social-Emotional Responsiveness on the Relationship Between Cyberbullying Victimization and Affective Symptoms. *Psychology Research and Behavior Management*, *Volume 16*, 2545–2551. https://doi.org/10.2147/PRBM.S420771

Nomura, Y., Chemtob, C. M., Fifer, W. P., Newcorn, J. H., & Brooks‐Gunn, J. (2006). Additive Interaction of Child Abuse and Perinatal Risk as Signs of Resiliency in Adulthood. *Annals of the New York Academy of Sciences*, *1094*(1), 330–334. https://doi.org/10.1196/annals.1376.044

Oshri, A., Carlson, M. W., Kwon, J. A., Zeichner, A., & Wickrama, K. K. A. S. (2017). Developmental Growth Trajectories of Self-Esteem in Adolescence: Associations with Child Neglect and Drug Use and Abuse in Young Adulthood. *Journal of Youth and Adolescence*, *46*(1), 151–164. https://doi.org/10.1007/s10964-016-0483-5

Palagini, L., Miniati, M., Marazziti, D., Franceschini, C., Zerbinati, L., Grassi, L., Sharma, V., & Riemann, D. (2022). Insomnia symptoms are associated with impaired resilience in bipolar disorder: Potential links with early life stressors may affect mood features and suicidal risk. *Journal of Affective Disorders*, *299*, 596–603. https://doi.org/10.1016/j.jad.2021.12.042

Pardo, A. (2008). *Positive religious coping as a moderator of the relationship of physical abuse to generalized self-efficacy and dissociation*. Pacific Graduate School of Psychology.

Park, J. Y., Lee, C. W., Jang, Y., Lee, W., Yu, H., Yoon, J., Oh, S., Park, Y. S., Ryoo, H. A., Lee, J., Cho, N., Lee, C. H., Lee, Y. C., Won, H.-H., Kang, H. S., Ha, T. H., & Myung, W. (2023). Relationship between childhood trauma and resilience in patients with mood disorders. *Journal of Affective Disorders*, *323*, 162–170. https://doi.org/10.1016/j.jad.2022.11.003

Pelcovitz, M. (2015). *Familial support as a protective factor of long-term psychological well-being in physically abused adolescents*. St John’s University.

Pereda, N., & Sicilia, L. (2017). Reacciones sociales ante la revelación de abuso sexual infantil y malestar psicológico en mujeres víctimas. *Psychosocial Intervention*, *26*(3), 131–138. https://doi.org/10.1016/j.psi.2017.02.002

Petros, N., Opacka-Juffry, J., & Huber, J. H. (2013). Psychometric and neurobiological assessment of resilience in a non-clinical sample of adults. *Psychoneuroendocrinology*, *38*(10), 2099–2108. https://doi.org/10.1016/j.psyneuen.2013.03.022

Picci, G., Linden-Carmichael, A. N., & Rose, E. J. (2023). Resilience profiles predict polysubstance use in adolescents with a history of childhood maltreatment. *Addiction Research & Theory*, *31*(2), 137–147. https://doi.org/10.1080/16066359.2022.2132237

Polepally Ashok, G. (2017). *Childhood trauma, BDSM, and self-esteem: An exploration of the impact of childhood trauma on sexual behavior and the effects to self-esteem*. The Chicago School of Professional Psychology.

Proskynitopoulos, P. J., Heitland, I., Glahn, A., Bauersachs, J., Westhoff-Bleck, M., & Kahl, K. G. (2021). Prevalence of Child Maltreatment in Adults With Congenital Heart Disease and Its Relationship With Psychological Well-Being, Health Behavior, and Current Cardiac Function. *Frontiers in Psychiatry*, *12*, 686169. https://doi.org/10.3389/fpsyt.2021.686169

Rajalin, M., Hirvikoski, T., Renberg, E. S., Åsberg, M., & Jokinen, J. (2020). Exposure to Early Life Adversity and Interpersonal Functioning in Attempted Suicide. *Frontiers in Psychiatry*, *11*, 552514. https://doi.org/10.3389/fpsyt.2020.552514

Ramos Salazar, L. (2021). Cyberbullying Victimization as a Predictor of Cyberbullying Perpetration, Body Image Dissatisfaction, Healthy Eating and Dieting Behaviors, and Life Satisfaction. *Journal of Interpersonal Violence*, *36*(1–2), 354–380. https://doi.org/10.1177/0886260517725737

Reed, S. (2017). *Parenting, attachment, and resiliency among women who were bullied in high school*. Capella University.

Rhoden-Neita, M.-A., Huang, H., & Fava, N. M. (2024). The Effects of Childhood Adversity and Self-Regulation on the Well-Being of Adults with Delinquency Histories: A Mediation Model. *Journal of Evidence-Based Social Work*, *21*(1), 117–139. https://doi.org/10.1080/26408066.2023.2265923

Richter, A., Krämer, B., Diekhof, E. K., & Gruber, O. (2019). Resilience to adversity is associated with increased activity and connectivity in the VTA and hippocampus. *NeuroImage: Clinical*, *23*, 101920. https://doi.org/10.1016/j.nicl.2019.101920

Ridder, K. A. (2018). *Investigating social information processing and emotion regulation as mediating links between childhood abuse and adult aggression*. Rosalink Franklin University of Medicine and Science.

Roberts, R., O’Connor, T., Dunn, J., & Golding, J. (2004). The effects of child sexual abuse in later family life; mental health, parenting and adjustment of offspring. *Child Abuse & Neglect*, *28*(5), 525–545. https://doi.org/10.1016/j.chiabu.2003.07.006

Robinson, M., Ross, J., Fletcher, S., Burns, C. R., Lagdon, S., & Armour, C. (2021). The Mediating Role of Distress Tolerance in the Relationship Between Childhood Maltreatment and Mental Health Outcomes Among University Students. *Journal of Interpersonal Violence*, *36*(15–16), 7249–7273. https://doi.org/10.1177/0886260519835002

Roche-Miranda, M. I., Subervi-Vázquez, A. M., & Martinez, K. G. (2023). Ataque de nervios: The impact of sociodemographic, health history, and psychological dimensions on Puerto Rican adults. *Frontiers in Psychiatry*, *14*, 1013314. https://doi.org/10.3389/fpsyt.2023.1013314

Rogosch, F. A., Oshri, A., & Cicchetti, D. (2010). From child maltreatment to adolescent cannabis abuse and dependence: A developmental cascade model. *Development and Psychopathology*, *22*(4), 883–897. https://doi.org/10.1017/S0954579410000520

Rohner, S. L., Bernays, F., Maercker, A., & Thoma, M. V. (2022). Salutary mechanisms in the relationship between stress and health: The mediating and moderating roles of Sense of Coherence—Revised. *Stress and Health*, *38*(2), 388–401. https://doi.org/10.1002/smi.3093

Romera, E. M., Luque-González, R., García-Fernández, C. M., & Ortega-Ruiz, R. (2022). Competencia social y bullying: El papel de la edad y el sexo. *Educación XX1*, *25*(1), 309–333. https://doi.org/10.5944/educxx1.30461

Rompilla, D. B., Stephens, J. E., Martinez, M., Mikels, J. A., & Haase, C. M. (2023). Can emotional acceptance buffer the link between executive functioning and mental health in late life? *Emotion*, *23*(8), 2286–2299. https://doi.org/10.1037/emo0001236

Rose, H., Womick, J., & King, L. A. (2023). Purpose maintained: Adverse childhood experiences and meaning in life. *Journal of Personality*, *91*(6), 1425–1441. https://doi.org/10.1111/jopy.12820

Roy, A., Carli, V., & Sarchiapone, M. (2011). Resilience mitigates the suicide risk associated with childhood trauma. *Journal of Affective Disorders*, *133*(3), 591–594. https://doi.org/10.1016/j.jad.2011.05.006

Russo, J. E., Dhruve, D. M., & Oliveros, A. D. (2023a). Childhood Trauma and PTSD Symptoms: Disentangling the Roles of Emotion Regulation and Distress Tolerance. *Research on Child and Adolescent Psychopathology*, *51*(9), 1273–1287. https://doi.org/10.1007/s10802-023-01048-x

Russo, J. E., Dhruve, D. M., & Oliveros, A. D. (2023b). Role of Developmental Timing of Childhood Adversity in Nonsuicidal Self-Injury Persistence or Desistance. *Research on Child and Adolescent Psychopathology*, *51*(12), 1895–1908. https://doi.org/10.1007/s10802-023-01037-0

Saintil, M. (2017). *Long-term effects of peer victimization: Examining the link among early experiences with victimization, social support, and current well-being in honors college students*. Florida State University.

Sappington, A. A., Pharr, R., Tunstall, A., & Rickert, E. (1997). Relationships among child abuse, date abuse, and psychological problems. *Journal of Clinical Psychology*, *53*(4), 319–329. https://doi.org/10.1002/(SICI)1097-4679(199706)53:4<319::AID-JCLP4>3.0.CO;2-K

Sassoon, S. A., Fama, R., Le Berre, A.-P., Müller-Oehring, E. M., Zahr, N. M., Pfefferbaum, A., & Sullivan, E. V. (2023). Influence of childhood trauma, HIV infection, alcohol use disorder, and resilience on health-related quality of life in adulthood. *Journal of Psychiatric Research*, *163*, 230–239. https://doi.org/10.1016/j.jpsychires.2023.05.033

Sauceda, J. A., Wiebe, J. S., & Simoni, J. M. (2016). Childhood sexual abuse and depression in Latino men who have sex with men: Does resilience protect against nonadherence to antiretroviral therapy? *Journal of Health Psychology*, *21*(6), 1096–1106. https://doi.org/10.1177/1359105314546341

Savani, C. D., Jani, M. P., Patel, A. H., Modi, P. R., & Odedara, V. K. (2023). Cyberbullying Victimisation and Psychological Well-being: A Cross-sectional Study among Medical Students in Western India. *JOURNAL OF CLINICAL AND DIAGNOSTIC RESEARCH*. https://doi.org/10.7860/JCDR/2023/61372.17913

Schiele, M. A., Herzog, K., Kollert, L., Schartner, C., Leehr, E. J., Böhnlein, J., Repple, J., Rosenkranz, K., Lonsdorf, T. B., Dannlowski, U., Zwanzger, P., Reif, A., Pauli, P., Deckert, J., & Domschke, K. (2020). Extending the vulnerability–stress model of mental disorders: Three-dimensional *NPSR1* × environment × coping interaction study in anxiety. *The British Journal of Psychiatry*, *217*(5), 645–650. https://doi.org/10.1192/bjp.2020.73

Schiele, M. A., Ziegler, C., Holitschke, K., Schartner, C., Schmidt, B., Weber, H., Reif, A., Romanos, M., Pauli, P., Zwanzger, P., Deckert, J., & Domschke, K. (2016). Influence of 5-HTT variation, childhood trauma and self-efficacy on anxiety traits: A gene-environment-coping interaction study. *Journal of Neural Transmission*, *123*(8), 895–904. https://doi.org/10.1007/s00702-016-1564-z

Schumm, J. A., Briggs‐Phillips, M., & Hobfoll, S. E. (2006). Cumulative interpersonal traumas and social support as risk and resiliency factors in predicting PTSD and depression among inner‐city women. *Journal of Traumatic Stress*, *19*(6), 825–836. https://doi.org/10.1002/jts.20159

Shafiq, S., & Batool, S. (2022). Bullying, Victimization, Rejection Sensitivity, and Self- regulation in Positive Development of Adolescents. *Journal of Liaquat University of Medical & Health Sciences*, *21*(01), 55–59. https://doi.org/10.22442/jlumhs.2022.00883

Shaheen, H., Rashid, S., & Aftab, N. (2023). Dealing with feelings: Moderating role of cognitive emotion regulation strategies on the relationship between cyber-bullying victimization and psychological distress among students. *Current Psychology*, *42*(34), 29745–29753. https://doi.org/10.1007/s12144-023-04934-1

Sheffler, J., Meyer, C., & Puga, F. (2022). Multi-sample assessment of stress reactivity as a mediator between childhood adversity and mid- to late-life outcomes. *Aging & Mental Health*, *26*(6), 1207–1216. https://doi.org/10.1080/13607863.2021.1910787

Shen, F., & Soloski, K. L. (2024). Examining the Moderating Role of Childhood Attachment for the Relationship between Child Sexual Abuse and Adult Attachment. *Journal of Family Violence*, *39*(3), 347–357. https://doi.org/10.1007/s10896-022-00456-9

Sheridan, M. A., Shi, F., Miller, A. B., Salhi, C., & McLaughlin, K. A. (2020). Network structure reveals clusters of associations between childhood adversities and development outcomes. *Developmental Science*, *23*(5), e12934. https://doi.org/10.1111/desc.12934

Shi, J., Chen, Y., Jiang, Y., Li, Y., Wang, W., Zhao, H., Guo, L., Liao, Y., Zhang, H., Gao, C., McIntyre, R. S., Zhang, W.-H., Han, X., & Lu, C. (2024). Stigma and its associations with medication adherence in major depressive disorder. *Psychiatry Research*, *331*, 115664. https://doi.org/10.1016/j.psychres.2023.115664

Smith, G. C., Dolbin-MacNab, M., Infurna, F., Webster, B., Musil, C., Castro, S., & Crowley, D. M. (2023). Self-reported Adverse Childhood Experiences Among Custodial Grandmothers: Frequencies, Patterns, and Correlates. *The International Journal of Aging and Human Development*, *97*(1), 81–110. https://doi.org/10.1177/00914150221106096

Smith, S., & Chesin, M. (2023). Belief in a just world mediates the relationship between childhood abuse and self-esteem in emerging adults. *Journal of Family Trauma, Child Custody & Child Development*, *20*(1), 81–98. https://doi.org/10.1080/26904586.2022.2049463

Snow, J., Moorman, J., & Romano, E. (2022). Emotion Regulation and Mental Health among Men with Childhood Sexual Abuse Histories. *Journal of Child Sexual Abuse*, *31*(4), 412–430. https://doi.org/10.1080/10538712.2021.1970677

Snyder, K. S., Luchner, A. F., & Tantleff-Dunn, S. (2024). Adverse childhood experiences and insecure attachment: The indirect effects of dissociation and emotion regulation difficulties. *Psychological Trauma: Theory, Research, Practice, and Policy*, *16*(Suppl 1), S20–S27. https://doi.org/10.1037/tra0001532

Sölva, K., Haselgruber, A., & Lueger-Schuster, B. (2023). Resilience in the face of adversity: Classes of positive adaptation in trauma-exposed children and adolescents in residential care. *BMC Psychology*, *11*(1), 30. https://doi.org/10.1186/s40359-023-01049-x

Somefun, O., Theron, L., & Ungar, M. (2023). The association between family adversity and youth mental health outcomes. *Journal of Adolescence*, *95*(7), 1333–1347. https://doi.org/10.1002/jad.12205

Stanislawski, E. R., Saali, A., Magill, E. B., Deshpande, R., Kumar, V., Chan, C., Hurtado, A., Charney, D. S., Ripp, J., & Katz, C. L. (2023). Longitudinal Mental Health Outcomes of Third-year Medical Students Rotating Through the Wards During COVID-19. *Psychiatry Research*, *320*, 115030. https://doi.org/10.1016/j.psychres.2022.115030

Stanton, M. V., Jones, A., & Shahani, D. (2022). Relationship among interpersonal stressors in adolescence, social support buffers, and obesity in adulthood 14 years later. *Health Psychology*, *41*(12), 904–911. https://doi.org/10.1037/hea0001223

Steensma, H., & Van Dijke, R. (2006). Attributional Styles, Self-Esteem, and Just World Belief of Victims of Bullying in Dutch Organisations. *International Quarterly of Community Health Education*, *25*(4), 381–392. https://doi.org/10.2190/9312-064K-67Q7-004Q

Stein, J. A., Leslie, M. B., & Nyamathi, A. (2002). Relative contributions of parent substance use and childhood maltreatment to chronic homelessness, depression, and substance abuse problems among homeless women: Mediating roles of self-esteem and abuse in adulthood. *Child Abuse & Neglect*, *26*(10), 1011–1027. https://doi.org/10.1016/S0145-2134(02)00382-4

Strøm, I. F., Aakvaag, H. F., Birkeland, M. S., Felix, E., & Thoresen, S. (2018). The mediating role of shame in the relationship between childhood bullying victimization and adult psychosocial adjustment. *European Journal of Psychotraumatology*, *9*(1), 1418570. https://doi.org/10.1080/20008198.2017.1418570

Su, Y., Li, M., D’Arcy, C., Caron, J., & Meng, X. (2023). Childhood maltreatment and major depressive disorder in well-being: A network analysis of a longitudinal community-based cohort. *Psychological Medicine*, *53*(15), 7180–7188. https://doi.org/10.1017/S0033291723000673

Sullivan, K. S., Park, Y., Richardson, S., Stander, V., & Jaccard, J. (2024). The role of problem solving appraisal and support in the relationship between stress exposure and posttraumatic stress symptoms of military spouses and service member partners. *Stress and Health*, *40*(4), e3371. https://doi.org/10.1002/smi.3371

Švecová, J., Furstova, J., Kaščáková, N., Hašto, J., & Tavel, P. (2023). The effect of childhood trauma and resilience on psychopathology in adulthood: Does bullying moderate the associations? *BMC Psychology*, *11*(1), 230. https://doi.org/10.1186/s40359-023-01270-8

Tanacioğlu Aydin, B., & Pekşen Süslü, D. (2023). The mediating role of difficulties in emotion regulation in the relationship between childhood trauma and resilience among university students. *Türk Psikolojik Danışma ve Rehberlik Dergisi*, *13*(70), 315–329. https://doi.org/10.17066/tpdrd.1265528.4

Terock, J., Van der Auwera, S., Hannemann, A., Janowitz, D., Homuth, G., Teumer, A., & Grabe, H. J. (2019). Interaction of childhood trauma with rs1360780 of the FKBP5 gene on trait resilience in a general population sample. *Journal of Psychiatric Research*, *116*, 104–111. https://doi.org/10.1016/j.jpsychires.2019.06.008

Theodora, M., Sahrani, R., & Roswiyani, R. (2023). The mediating effect of forgiveness on the relationship between spirituality and psychological well-being in adults with history of childhood bullying. *Psikohumaniora: Jurnal Penelitian Psikologi*, *8*(2), 241–258. https://doi.org/10.21580/pjpp.v8i2.17829

Thoma, M. V., Höltge, J., Eising, C. M., Pfluger, V., & Rohner, S. L. (2020). Resilience and Stress in Later Life: A Network Analysis Approach Depicting Complex Interactions of Resilience Resources and Stress-Related Risk Factors in Older Adults. *Frontiers in Behavioral Neuroscience*, *14*, 580969. https://doi.org/10.3389/fnbeh.2020.580969

Titelius, E. N., Cook, E., Spas, J., Orchowski, L., Kivisto, K., O’Brien, K., Frazier, E., Wolff, J. C., Dickstein, D. P., Kim, K. L., & Seymour, K. E. (2018). Emotion Dysregulation Mediates the Relationship Between Child Maltreatment and Non-Suicidal Self-Injury. *Journal of Aggression, Maltreatment & Trauma*, *27*(3), 323–331. https://doi.org/10.1080/10926771.2017.1338814

Tonmyr, L., Wekerle, C., Zangeneh, M., & Fallon, B. (2011). Childhood Maltreatment, Risk and Resilience. *International Journal of Mental Health and Addiction*, *9*(4), 343–346. https://doi.org/10.1007/s11469-011-9344-3

Topitzes, J., Mersky, J. P., & Reynolds, A. J. (2010). Child Maltreatment and Adult Cigarette Smoking: A Long-term Developmental Model. *Journal of Pediatric Psychology*, *35*(5), 484–498. https://doi.org/10.1093/jpepsy/jsp119

Tung, N. Y. C., Yap, Y., Bei, B., Luecken, L. J., & Wiley, J. F. (2022). Resilience to early family risk moderates stress-affect associations: A 14-day ecological momentary assessment study. *Journal of Affective Disorders*, *311*, 134–142. https://doi.org/10.1016/j.jad.2022.05.075

Uchida, Y., Takahashi, T., Katayama, S., Masuya, J., Ichiki, M., Tanabe, H., Kusumi, I., & Inoue, T. (2018). Influence of trait anxiety, child maltreatment, and adulthood life events on depressive symptoms. *Neuropsychiatric Disease and Treatment*, *Volume 14*, 3279–3287. https://doi.org/10.2147/NDT.S182783

Upenieks, L. (2021). Resilience in the Aftermath of Childhood Abuse? Changes in Religiosity and Adulthood Psychological Distress. *Journal of Religion and Health*, *60*(4), 2677–2701. https://doi.org/10.1007/s10943-020-01155-9

Upenieks, L., & Ford-Robertson, J. (2023). Childhood Abuse, Goal-Striving Stress and Self-Esteem: An Explanatory Role for Perceptions of Divine Control? *Journal of Religion and Health*, *62*(2), 906–931. https://doi.org/10.1007/s10943-022-01682-7

Vaillancourt-Morel, M.-P., Byers, E. S., Péloquin, K., & Bergeron, S. (2021). A Dyadic Longitudinal Study of Child Maltreatment and Sexual Well-Being in Adult Couples: The Buffering Effect of a Satisfying Relationship. *The Journal of Sex Research*, *58*(2), 248–260. https://doi.org/10.1080/00224499.2020.1792396

Van Der Werff, S. J. A., Pannekoek, J. N., Veer, I. M., Van Tol, M.-J., Aleman, A., Veltman, D. J., Zitman, F. G., Rombouts, S. A. R. B., Elzinga, B. M., & Van Der Wee, N. J. A. (2013). Resilience to childhood maltreatment is associated with increased resting-state functional connectivity of the salience network with the lingual gyrus. *Child Abuse & Neglect*, *37*(11), 1021–1029. https://doi.org/10.1016/j.chiabu.2013.07.008

Walker, H. E., Wamser-Nanney, R., & Howell, K. H. (2021). Child Sexual Abuse and Adult Sexual Assault among Emerging Adults: Exploring the Roles of Posttraumatic Stress Symptoms, Emotion Regulation, and Anger. *Journal of Child Sexual Abuse*, *30*(4), 407–426. https://doi.org/10.1080/10538712.2021.1890295

Walker-Williams, H. J., & Fouché, A. (2018). Resilience enabling processes and posttraumatic growth outcomes in a group of women survivors of childhood sexual abuse. *Health SA Gesondheid*, *23*. https://doi.org/10.4102/hsag.v23i0.1134

Wang, S. S., Walsh, K., & Li, J. J. (2024). A prospective longitudinal study of multidomain resilience among youths with and without maltreatment histories. *Development and Psychopathology*, *36*(2), 750–764. https://doi.org/10.1017/S0954579423000032

Wang, W., Zhang, J., Zheng, X., Li, G., & Zhou, Y. (2023). Heterogeneity of resilience and the curvilinear relationship between childhood trauma and resilience among people with schizophrenia. *Frontiers in Psychiatry*, *14*, 1082000. https://doi.org/10.3389/fpsyt.2023.1082000

Wang, Y.-C., Moya Guerola, M., Lin, Y.-C., Hsieh, Y.-P., Strong, C., Tsai, M.-C., & Lin, C.-Y. (2019). Effects of childhood adversity and resilience on Taiwanese youth health behaviors. *Pediatrics & Neonatology*, *60*(4), 368–376. https://doi.org/10.1016/j.pedneo.2018.08.004

Warmingham, J. M., Duprey, E. B., Handley, E. D., Rogosch, F. A., & Cicchetti, D. (2023). Patterns of childhood maltreatment predict emotion processing and regulation in emerging adulthood. *Development and Psychopathology*, *35*(2), 766–781. https://doi.org/10.1017/S0954579422000025

Watt, N. F., David, J. P., Ladd, K. L., & Shamos, S. (1995). The life course of psychological resilience: A phenomenological perspective on deflecting life’s slings and arrows. *The Journal of Primary Prevention*, *15*(3), 209–246. https://doi.org/10.1007/BF02197473

Weindl, D., Knefel, M., Glück, T., & Lueger-Schuster, B. (2020). Emotion regulation strategies, self-esteem, and anger in adult survivors of childhood maltreatment in foster care settings. *European Journal of Trauma & Dissociation*, *4*(4), 100163. https://doi.org/10.1016/j.ejtd.2020.100163

Wen, X., Li, Y., Li, Y. H., Chang, J. J., Yuan, M. Y., Cao, L. L., Wang, S. J., Su, P. Y., & Wang, G. F. (2023). Childhood Sexual Abuse and Quality of Life in Chinese Undergraduates: The Mediating Role of Resilience. *Biomedical and Environmental Sciences: BES*, *36*(7), 658–662. https://doi.org/10.3967/bes2023.095

Wild, N. D., & Paivio, S. C. (2004). Psychological Adjustment, Coping, and Emotion Regulation as Predictors of Posttraumatic Growth. *Journal of Aggression, Maltreatment & Trauma*, *8*(4), 97–122. https://doi.org/10.1300/J146v08n04_05

Wind, T. W., & Silvern, L. (1992). Type and extent of child abuse as predictors of adult functioning. *Journal of Family Violence*, *7*(4), 261–281. https://doi.org/10.1007/BF00994618

Wingo, A. P., Wrenn, G., Pelletier, T., Gutman, A. R., Bradley, B., & Ressler, K. J. (2010). Moderating effects of resilience on depression in individuals with a history of childhood abuse or trauma exposure. *Journal of Affective Disorders*, *126*(3), 411–414. https://doi.org/10.1016/j.jad.2010.04.009

Wolf, N. M., & Elklit, A. (2020). Child Maltreatment and Disordered Eating in Adulthood: A Mediating Role of PTSD and Self-Esteem? *Journal of Child & Adolescent Trauma*, *13*(1), 21–32. https://doi.org/10.1007/s40653-018-0224-x

Wong, S. L., & Chung, M. C. (2023). Child abuse and non-suicidal self-injury among Chinese university students: The role of emotion dysregulation and attachment style. *Current Psychology*, *42*(6), 4862–4872. https://doi.org/10.1007/s12144-021-01832-2

Wu, C.-Z., Zong, Z.-Y., Huang, T.-T., Yu, L.-X., & Sun, Q.-W. (2023). Childhood maltreatment influences suicidal behavior: Rumination mediates and regulatory emotional self-efficacy moderates. *Death Studies*, *47*(7), 784–791. https://doi.org/10.1080/07481187.2022.2132319

Wu, Q., Chi, P., Lin, X., & Du, H. (2018). Child maltreatment and adult depressive symptoms: Roles of self-compassion and gratitude. *Child Abuse & Neglect*, *80*, 62–69. https://doi.org/10.1016/j.chiabu.2018.03.013

Xu, B., Wei, S., Yin, X., Jin, X., Yan, S., & Jia, L. (2023). The relationship between childhood emotional neglect experience and depressive symptoms and prefrontal resting functional connections in college students: The mediating role of reappraisal strategy. *Frontiers in Behavioral Neuroscience*, *17*, 927389. https://doi.org/10.3389/fnbeh.2023.927389

Xu, J., Tully, L. A., & Dadds, M. R. (2024). Generation time-out grows up: Young adults’ reports about childhood time-out use and their mental health, attachment, and emotion regulation. *European Child & Adolescent Psychiatry*, *33*(10), 3471–3479. https://doi.org/10.1007/s00787-024-02408-8

Xu, X., Li, Y., Liu, S., & Wang, W. (2024). Longitudinal Relationships Between Bullying Victimization and Dual Social Behaviors: The Roles of Self-Compassion and Trauma-Related Shame. *Psychology Research and Behavior Management*, *Volume 17*, 1463–1475. https://doi.org/10.2147/PRBM.S450013

Xu, Z., Zhang, D., Ding, H., Zheng, X., Lee, R. C.-M., Yang, Z., Mo, P. K.-H., Lee, E. K.-P., & Wong, S. Y.-S. (2022). Association of positive and adverse childhood experiences with risky behaviours and mental health indicators among Chinese university students in Hong Kong: An exploratory study. *European Journal of Psychotraumatology*, *13*(1), 2065429. https://doi.org/10.1080/20008198.2022.2065429

Yagci, I., & Inaltekin, A. (2023). Social Phobia, Traumatic Experiences and Other Psychosocial Factors Accompanying Vaginismus. *Eurasian Journal of Family Medicine*, *12*(2), 63–70. https://doi.org/10.33880/ejfm.2023120203

Ye, Z., Wei, X., Zhang, J., Li, H., & Cao, J. (2024). The impact of adverse childhood experiences on depression: The role of insecure attachment styles and emotion dysregulation strategies. *Current Psychology*, *43*(5), 4016–4026. https://doi.org/10.1007/s12144-023-04613-1

Yetim, O. (2022). Examining the relationships between stressful life event, resilience, self-esteem, trauma, and psychiatric symptoms in Syrian migrant adolescents living in Turkey. *International Journal of Adolescence and Youth*, *27*(1), 221–234. https://doi.org/10.1080/02673843.2022.2072749

Young-Wolff, K. C., Alabaster, A., McCaw, B., Stoller, N., Watson, C., Sterling, S., Ridout, K. K., & Flanagan, T. (2019). Adverse Childhood Experiences and Mental and Behavioral Health Conditions During Pregnancy: The Role of Resilience. *Journal of Women’s Health (2002)*, *28*(4), 452–461. https://doi.org/10.1089/jwh.2018.7108

Youssef, N. A., Belew, D., Hao, G., Wang, X., Treiber, F. A., Stefanek, M., Yassa, M., Boswell, E., McCall, W. V., & Su, S. (2017). Racial/ethnic differences in the association of childhood adversities with depression and the role of resilience. *Journal of Affective Disorders*, *208*, 577–581. https://doi.org/10.1016/j.jad.2016.10.024

Youssef, N. A., Green, K. T., Dedert, E. A., Hertzberg, J. S., Calhoun, P. S., Dennis, M. F., Research Education And Clinical Cen, M.-A. M. I., & Beckham, J. C. (2013). Exploration of the Influence of Childhood Trauma, Combat Exposure, and the Resilience Construct on Depression and Suicidal Ideation Among U.S. Iraq/Afghanistan Era Military Personnel and Veterans. *Archives of Suicide Research*, *17*(2), 106–122. https://doi.org/10.1080/13811118.2013.776445

Yu, W., Zhu, F., Foo, M. D., & Wiklund, J. (2022). What does not kill you makes you stronger: Entrepreneurs’ childhood adversity, resilience, and career success. *Journal of Business Research*, *151*, 40–55. https://doi.org/10.1016/j.jbusres.2022.06.035

Yundt, G. C. (2019). *The effect of adverse childhood experiences on psychosocial wellbeing*. George Fox University.

Zhou, S., Chen, J., Lin, H., Ye, Y., Xiao, Y., Ouyang, N., Pan, S., Feng, S., Xie, M., & Li, B. (2022). Associations Among Workplace Bullying, Resilience, Insomnia Severity, and Subjective Wellbeing in Chinese Resident Doctors. *Frontiers in Psychiatry*, *13*, 840945. https://doi.org/10.3389/fpsyt.2022.840945

Zilioli, S., Slatcher, R. B., Chi, P., Li, X., Zhao, J., & Zhao, G. (2016). Childhood Adversity, Self-Esteem, and Diurnal Cortisol Profiles Across the Life Span. *Psychological Science*, *27*(9), 1249–1265. https://doi.org/10.1177/0956797616658287

# SA7. Qualitative synthesis

**Synthesis of studies not included in meta-analysis but fulfilling inclusion criteria and included in the systematic review**

Twenty of the 203 included studies were not included in the meta-analyses because though they explored specific CM subtypes or resilience domains, they did not report sufficient data (*n* and/or *k <* 5) to be pooled.

***Bullying***

Five studies (Armitage et al., 2021; Bouchard & Sonier, 2021; Goodboy et al., 2016; Newman et al., 2011; Yubero et al., 2021) explored associations between bullying or peer victimisation and resilience outcomes.

In an online survey, tapping into (a) experiences as a victim of *bullying* during adolescence, (b) typical strategies for coping with stress, and (c) current levels of stress among 1339 college students, authors found that victimisation history was associated with both increased stress and an increased use of *avoidant coping strategies*. In addition, avoidant coping partially mediated the link between victimisation and stress, suggesting that avoidant coping may develop as an adaptive response to uncontrollable stress but that, in the long term, these strategies are a maladaptive approach to coping that acts to prolong stress (Newman et al., 2011).

Another study found a negative association between high school victimisation experiences of *bullying* (relational-verbal bullying, cyberbullying, physical bullying, culture-based bullying) and current *motivation* for attending college (intrinsic motivation, extrinsic motivation, amotivation) with first-semester *adjustment* (academic adjustment, social adjustment, personal-emotional adjustment, institutional attachment) in 149 college students (Goodboy et al., 2016).

In a prospective cohort study based in the UK, over 15% of victims of frequent *bullying* had a diagnosis of depression at age 18. Victimisation also had a significant impact on well-being, with a one-point increase in frequent victimisation associated with a 2.71-point decrease in well-being scores after adjustment for any mediating/moderating effects of depression. This suggests that the burden of victimisation extends beyond depression to impact well-being. Results therefore show that individuals who remain partially resilient by avoiding a diagnosis of depression after victimisation have significantly poorer well-being than their non-victimised counterparts (Armitage et al., 2021).

Another study found that chronic *bullying* victimisation (before and at university) was associated with impairments in emotional *well-being* in 1122 university students*,* and that resilience moderated this association (Yubero et al., 2021).

In a study using structural equation modelling in a sample of 200 young adults and their mothers, intrusive parental style during conflicts between siblings was related to higher levels of *sibling bullying* (including both perpetration and victimisation) in childhood. Furthermore, young adults who were involved in sibling bullying as a child displayed less *positive social problem-solving behaviours* (Bouchard & Sonier, 2021).

***Domestic violence***

Three studies (Chi et al., 2021; Naughton et al., 2020; Wind & Silvern, 1994) explored domestic violence exposure and resilience outcomes.

A study examined whether variation in perceived parental warmth and non-abusive family stressors influenced the strength of relationships between psychological difficulties and childhood sexual and/or physical abuse in 259 working women, and found that perceived parental warmth, childhood stress, and abuse were each separately associated with current functioning. Parental warmth strongly influenced or mediated the relationship of intrafamilial child abuse to depression and *self-esteem* levels; abuse was associated with PTSD independently of variation in perceived parenting; and parenting mediated initial relationships of childhood stress to each of the adjustment measures (Wind & Silvern, 1994).

Using confirmatory factor analysis, a study verified the presence of a two-factor model (physical and psychological) for *domestic violence* and found that exposure to psychological (but not physical) domestic violence was related to reduced *psychological well-being* in 465 young adults. However, mediation analysis suggested the presence of a suppression effect; there was a magnification of the negative relationship between exposure to psychological domestic violence and social support satisfaction when exposure to physical domestic violence was accounted for (Naughton et al., 2020).

In a nationwide cross-sectional online survey conducted in China during the COVID-19 pandemic, exposure to *domestic violence* was significantly associated with *post-traumatic growth* and PTSD via a one-step indirect path of self-compassion and via a two-step indirect path from self-compassion to resilience. However, resilience did not mediate the relationship between exposure to domestic violence and post-traumatic growth and PTSD among 2038 university students (Chi et al., 2021).

***Abuse and Neglect***

Five studies investigated associations between total scores of abuse or neglect and resilience outcomes (Feinauer et al., 1996; Kapoor et al., 2018; Kim et al., 2021; Sachs-Ericsson et al., 2011; Shin & Brunton, 2024).

One study explored the relationship of hardiness, severity of abuse, frequency and duration of abuse, and the identity of the perpetrator on *adjustment* in a non-clinical sample of 255 women survivors. Using a path analysis, results indicated that 1) longer duration and more frequent abuse resulted in higher severity scores; 2) women who showed higher levels of commitment, control, and challenge were better adjusted; 3) women whose abuse was more frequent and of longer duration demonstrated more emotional trauma symptoms; and 4) father/ stepfather abuse was related to poorer adjustment (Feinauer et al., 1996).

Using structural equation modelling, a study examined the relations among *childhood abuse* (measured via physical, sexual, and emotional abuse), *intrapersonal strengths* (assessed by self-efficacy and spiritual well-being), and *suicide resilience* (operationalised via the three components of suicide resilience—internal protective, external protective, and emotional stability) in African American females who attempted suicide. Results indicated that childhood abuse was negatively associated with intrapersonal strengths and suicide resilience, intrapersonal strengths were positively associated with suicide resilience, and intrapersonal strengths fully mediated the association between childhood abuse and suicide resilience. This suggests a positive and protective influence of intrapersonal strengths on suicide resilience in the face of childhood abuse (Kapoor et al., 2018).

In a study with a multiethnic sample of older adults, *child abuse* was associated with the number of current medical problems, as well as disability. Child abuse was also related to lower *self-efficacy*, and self-efficacy explained the relationship between abuse and the number of health problems (Sachs-Ericsson et al., 2011).

Another study found that resilience and dysfunctional *coping strategies* mediated the association between *childhood abuse* and lifetime PTSD severity in adulthood, after covarying for the number of repeated trauma exposures, suggesting that resilience and coping strategies mediate the detrimental effects of childhood abuse on lifetime PTSD severity (Kim et al., 2021).

A study investigated the effects of attentional bias, executive function and *resilience* between *early life stress-threat* (abuse) and *early life stress-deprivation* (neglect) and mental health. Across two studies, authors found no association between early life stress-threat and attentional bias. Early life stress-threat predicted poor mental health. Early life stress-deprivation and early life stress-threat were associated with poorer executive function. Importantly, the effect of early life stress-deprivation on resilience was buffered by higher executive function in 176 adult participants (Shin & Brunton, 2024).

***Other resilience outcomes***

For other resilience outcomes not included in meta-analysis, seven studies explored sense of mastery, sense of coherence, qualities of self, and self-concept. Of them, one study using structural equation modelling explored associations between CM experiences and *sense of mastery*, which is the faith individuals have in their ability to influence the course and outcomes of meaningful life events (a well-recognised resilience resource in late adulthood) and found that the association between parental invalidating childhood experiences and sense of mastery in 316 older adults was mediated by both self-objectification and by disrupted body boundaries (Talmon et al., 2022).

Another study found a positive correlation between CM and cognitive distortions and a negative correlation between CM and *sense of coherence* (SOC; a resource that strengthens resilience and promotes health), and found that sexual abuse had a significant effect on SOC meaningfulness in 359 nursing students (Karakaş & Çingöl, 2022).

CM was positively associated with depression while negatively correlated with the SOC in older adults in a further study. A low level of SOC was also correlated with symptoms of depression. In addition, SOC partially mediated the association between CM and late-life depression, yet no moderation effect of SOC was found (Naderzadeh et al., 2023).

A study using a sample of adults in a romantic relationship examined Self, specifically *the qualities of Self* (e.g., compassion) and *Self-Leadership* (e.g., staying calm in the face of distress) as pathways linking CM to depression and relationship quality, and found that the indirect (mediating) effects from CM to depressive symptoms and relationship quality were significant through Self-Qualities, but not Self-Leadership, suggesting that CM was associated with less frequent access to the qualities of Self, such as compassion and confidence which, in turn, were associated with fewer depressive symptoms and higher levels of relationship quality (Fitzgerald & Barton, 2022).

A study explored whether *self-concept clarity* moderated the relationship between *childhood sexual abuse* and dissociation (Model 1), and whether childhood sexual abuse moderated the relationship between self-concept clarity and dissociation (Model 2) among 65 well-functioning young women with (*n* = 35) and without exposure to sexual trauma (*n* = 30). Results revealed that childhood sexual abuse was related to depersonalisation-derealisation only under low self-concept clarity levels (Model 1), and self-concept clarity was negatively related to depersonalisation-derealisation in the childhood sexual abuse group only (Model 2), suggesting that self-concept clarity is a protective factor, buffering the association between childhood sexual abuse and detachment (depersonalisation-derealisation) symptoms (Lassri et al., 2023).

A study examined the roles of *distress tolerance, self-compassion,* and *self-disgust* in the association between CM types and emotion regulation difficulty, which was expected to predict non-suicidal self-injury in 397 university students and found that low distress tolerance, low self-compassion, high self-disgust, and resulting high emotion regulation difficulty mediated the indirect effect of emotional neglect on non-suicidal self-injury. However, the results for physical, sexual, emotional abuse, and physical neglect experiences were not significant (Erol & Inozu, 2024).

In a study, *sociability* and *locus of control* were examined as protective factors in two separate three-way interaction models of the effects of childhood physical and emotional neglect on adult *social competence* in 405 introductory psychology students who completed a computerised battery of assessments examining multidomain regulation. In the physical neglect model, those with an internal locus of control, sociability was a protective factor, as evidenced by a strong negative relationship between physical neglect and social competence only when sociability was lower. However, for externally controlled individuals, higher sociability acted as a risk factor, strengthening the association between physical neglect and social competence (Garcia & Berzenski, 2023).

**Synthesis of studies included in meta-analyses that explored other resilience domains but did not provide enough data to be pooled**

Four studies (Arslan, 2015; Lu et al., 2017; Martxueta & Etxeberria, 2014; Wadji et al., 2023) included in meta-analysis explored CM subtypes (bullying) and resilience domains (i.e., self-concept, posttraumatic growth) but could not be pooled in meta-analyses due to insufficient data (*n* and/or *k <* 5).

Two studies (Arslan, G., 2015; Lu et al., 2017) explored associations between CM and *self-concept*. One indicated that childhood psychological maltreatment directly predicted resilience, negative self-concept and depression in adulthood, and that childhood psychological maltreatment indirectly predicted negative self-concept and depression by mediating resilience (Arslan, 2015). The study by Lu et al., (2017), which used structural equation model analysis, showed that CM was negatively associated with self-concept, self-efficacy, and abstinence motivation in 816 individuals with drug addiction recruited from compulsory detoxification units. Self-concept was positively associated with self-efficacy and abstinence motivation. Conversely, a significant association between self-efficacy and abstinence motivation was not found. An indirect analysis showed that self-concept mediated the relationship between CM and self-efficacy and arbitrated the relationship between CM and abstinence motivation. The indirect effect of self-concept between CM and abstinence motivation still existed when the total scores of CM were replaced by the scores of specific forms of CM (Lu et al., 2017).

One study investigated how experiences and the perceived acceptability of CM were related to resilience and *posttraumatic growth* in countries with different cultures, living standards, and gross national income, and included Cameroon (*n* = 111), Canada (*n* = 137), Japan (*n* = 108), and Germany (n = 122). The results indicated that while experiences of physical abuse and emotional maltreatment were negatively associated with resilience, experiences of emotional maltreatment were positively associated with posttraumatic growth (Wadji et al., 2023).

Finally, a study in a Spanish sample of 119 gay and bisexual individuals, bullying experienced in childhood and/or adolescence affected their psychological well-being and, specifically, depression and anxiety levels, self-esteem and affective balance. In addition, the instrumentality and expressiveness dimensions of gender identity were identified as factors that may influence psychological well-being (Martxueta & Etxeberria, 2014).

# ST1. PRISMA 2020 statement and checklist

| **Section and Topic** | **Item #** | **Checklist item** | **Location where item is reported** |
| --- | --- | --- | --- |
| **TITLE** | | |  |
| Title | 1 | Identify the report as a systematic review. | Cover page |
| **ABSTRACT** | | |  |
| Abstract | 2 | See the PRISMA 2020 for Abstracts checklist. | Abstract |
| **INTRODUCTION** | | |  |
| Rationale | 3 | Describe the rationale for the review in the context of existing knowledge. | Introduction |
| Objectives | 4 | Provide an explicit statement of the objective(s) or question(s) the review addresses. | Introduction |
| **METHODS** | | |  |
| Eligibility criteria | 5 | Specify the inclusion and exclusion criteria for the review and how studies were grouped for the syntheses. | Methods |
| Information sources | 6 | Specify all databases, registers, websites, organisations, reference lists and other sources searched or consulted to identify studies. Specify the date when each source was last searched or consulted. | Methods |
| Search strategy | 7 | Present the full search strategies for all databases, registers, and websites, including any filters and limits used. | Methods  Supplement |
| Selection process | 8 | Specify the methods used to decide whether a study met the inclusion criteria of the review, including how many reviewers screened each record and each report retrieved, whether they worked independently, and if applicable, details of automation tools used in the process. | Methods |
| Data collection process | 9 | Specify the methods used to collect data from reports, including how many reviewers collected data from each report, whether they worked independently, any processes for obtaining or confirming data from study investigators, and if applicable, details of automation tools used in the process. | Methods |
| Data items | 10a | List and define all outcomes for which data were sought. Specify whether all results that were compatible with each outcome domain in each study were sought (e.g., for all measures, time points, analyses), and if not, the methods used to decide which results to collect. | Methods Supplement |
|  | 10b | List and define all other variables for which data were sought (e.g., participant and intervention characteristics, funding sources). Describe any assumptions made about any missing or unclear information. | Methods Supplement |
| Study risk of bias assessment | 11 | Specify the methods used to assess risk of bias in the included studies, including details of the tool(s) used, how many reviewers assessed each study and whether they worked independently, and if applicable, details of automation tools used in the process. | Methods Supplement |
| Effect measures | 12 | Specify for each outcome the effect measure(s) (e.g., risk ratio, mean difference) used in the synthesis or presentation of results. | Methods |
| Synthesis methods | 13a | Describe the processes used to decide which studies were eligible for each synthesis (e.g., tabulating the study intervention characteristics and comparing against the planned groups for each synthesis (item #5)). | Methods |
|  | 13b | Describe any methods required to prepare the data for presentation or synthesis, such as handling of missing summary statistics, or data conversions. | Methods |
|  | 13c | Describe any methods used to tabulate or visually display results of individual studies and syntheses. | Methods |
|  | 13d | Describe any methods used to synthesise results and provide a rationale for the choice(s). If meta-analysis was performed, describe the model(s), method(s) to identify the presence and extent of statistical heterogeneity, and software package(s) used. | Methods |
|  | 13e | Describe any methods used to explore possible causes of heterogeneity among study results (e.g., subgroup analysis, meta-regression). | Methods |
|  | 13f | Describe any sensitivity analyses conducted to assess robustness of the synthesised results. | Methods |
| Reporting bias assessment | 14 | Describe any methods used to assess risk of bias due to missing results in a synthesis (arising from reporting biases). | Methods |
| Certainty assessment | 15 | Describe any methods used to assess certainty (or confidence) in the body of evidence for an outcome. | Methods |
| **RESULTS** | | |  |
| Study selection | 16a | Describe the results of the search and selection process, from the number of records identified in the search to the number of studies included in the review, ideally using a flow diagram. | Figure 1 Results |
|  | 16b | Cite studies that might appear to meet the inclusion criteria, but which were excluded, and explain why they were excluded. | Results  Supplement |
| Study characteristics | 17 | Cite each included study and present its characteristics. | Results  Supplement |
| Risk of bias in studies | 18 | Present assessments of risk of bias for each included study. | Results Supplement |
| Results of individual studies | 19 | For all outcomes, present, for each study: (a) summary statistics for each group (where appropriate) and (b) an effect estimate and its precision (e.g., confidence/credible interval), ideally using structured tables or plots. | Results  Table 2  Supplement |
| Results of syntheses | 20a | For each synthesis, briefly summarise the characteristics and risk of bias among contributing studies. | Results  Table 1  Supplement |
|  | 20b | Present results of all statistical syntheses conducted. If meta-analysis was done, present for each the summary estimate and its precision (e.g., confidence/credible interval) and measures of statistical heterogeneity. If comparing groups, describe the direction of the effect. | Results  Table 2  Figure 2 Supplement |
|  | 20c | Present results of all investigations of possible causes of heterogeneity among study results. | Results Supplement |
|  | 20d | Present results of all sensitivity analyses conducted to assess the robustness of the synthesised results. | Results Supplement |
| Reporting biases | 21 | Present assessments of risk of bias due to missing results (arising from reporting biases) for each synthesis assessed. | Results Supplement |
| Certainty of evidence | 22 | Present assessments of certainty (or confidence) in the body of evidence for each outcome assessed. | Results |
| **DISCUSSION** | | |  |
| Discussion | 23a | Provide a general interpretation of the results in the context of other evidence. | Discussion |
|  | 23b | Discuss any limitations of the evidence included in the review. | Discussion |
|  | 23c | Discuss any limitations of the review processes used. | Discussion |
|  | 23d | Discuss implications of the results for practice, policy, and future research. | Discussion |
| **OTHER INFORMATION** | | |  |
| Registration and protocol | 24a | Provide registration information for the review, including register name and registration number, or state that the review was not registered. | Abstract Methods |
|  | 24b | Indicate where the review protocol can be accessed, or state that a protocol was not prepared. | Abstract Methods |
|  | 24c | Describe and explain any amendments to information provided at registration or in the protocol. | Methods |
| Support | 25 | Describe sources of financial or non-financial support for the review, and the role of the funders or sponsors in the review. | Funding |
| Competing interests | 26 | Declare any competing interests of review authors. | Competing interest declaration |
| Availability of data, code and other materials | 27 | Report which of the following are publicly available and where they can be found: template data collection forms; data extracted from included studies; data used for all analyses; analytic code; any other materials used in the review. | Article information |

# ST2. PRISMA 2020 for abstracts checklist

| **Section and Topic** | **Item #** | **Checklist item** | **Reported (Yes/No)** |
| --- | --- | --- | --- |
| **TITLE** | | |  |
| Title | 1 | Identify the report as a systematic review. | Yes |
| **BACKGROUND** | | |  |
| Objectives | 2 | Provide an explicit statement of the main objective(s) or question(s) the review addresses. | Yes |
| **METHODS** | | |  |
| Eligibility criteria | 3 | Specify the inclusion and exclusion criteria for the review. | Yes |
| Information sources | 4 | Specify the information sources (e.g., databases, registers) used to identify studies and the date when each was last searched. | Yes |
| Risk of bias | 5 | Specify the methods used to assess risk of bias in the included studies. | Yes |
| Synthesis of results | 6 | Specify the methods used to present and synthesise results. | Yes |
| **RESULTS** | | |  |
| Included studies | 7 | Give the total number of included studies and participants and summarise relevant characteristics of studies. | Yes |
| Synthesis of results | 8 | Present results for main outcomes, preferably indicating the number of included studies and participants for each. If meta-analysis was done, report the summary estimate and confidence/credible interval. If comparing groups, indicate the direction of the effect (i.e. which group is favoured). | Yes |
| **DISCUSSION** | | |  |
| Limitations of evidence | 9 | Provide a brief summary of the limitations of the evidence included in the review (e.g. study risk of bias, inconsistency and imprecision). | Yes |
| Interpretation | 10 | Provide a general interpretation of the results and important implications. | Yes |
| **OTHER** | | |  |
| Funding | 11 | Specify the primary source of funding for the review. | NA |
| Registration | 12 | Provide the register name and registration number. | Yes |

# ST3. MOOSE guidelines and checklist

| **Criteria** | | **Brief description of how criteria were applied in the meta-analysis** |
| --- | --- | --- |
| **Reporting of background should include:** | |  |
| √ | Problem definition | To study the magnitude and consistency of associations between overall childhood maltreatment and its subtypes and global/trait resilience and its domains in adults, and to examine mediators and moderators of these relationships. |
| √ | Hypothesis statement | We hypothesised that there is a negative association between childhood maltreatment and resilience (domains), in that childhood maltreatment would be related to poorer resilience outcomes in adulthood. |
| √ | Description of study outcomes | Resilience: global/trait resilience capacity; Resilience domains: coping, self-esteem, emotion regulation, self-efficacy, well-being. |
| √ | Type of exposure or intervention used | Studies included were original articles investigating adults (≥ 18 years) exposed to maltreatment during childhood (≤18 years) (no intervention studies). |
| √ | Type of study designs used | Cross-sectional and longitudinal studies (only with baseline data). |
| √ | Study population | Individuals with or without mental conditions (clinical or non-clinical populations) |
| **Reporting of search strategy should include** | |  |
| √ | Qualifications of searchers | The credentials of the investigators/reviewers are indicated in the author list and acknowledgements. |
| √ | Search strategy, including time period included in the synthesis and keywords | We used specific keywords related to the type of diverse resilience domains, and childhood maltreatment. A second step involved a manual search of the reference lists of retrieved articles. |
| √ | Databases and registries searched | Embase, PubMed (Medline), Web of Science (Core collection), PsycINFO, Scopus. |
| √ | Search software used, name and version, including special features | Zotero 5.0.96.3, Rayyan (https://www.rayyan.com), Comprehensive Meta-analysis version 4.0, R version 4.1.2. |
| √ | Use of hand searching | A snow-balling approach was applied to identify additional studies in the reference lists of retrieved studies. We hand-searched bibliographies of retrieved papers for additional references. |
| √ | List of citations located and those excluded, including justifications | Details of the literature search process are outlined in the PRISMA flowchart. |
| √ | Method of addressing articles published in languages other than English | The search included abstracts without language restrictions. |
| √ | Method of handling abstracts and unpublished studies | Abstracts and unpublished studies (grey literature) were excluded. |
| √ | Description of any contact with authors | Where necessary, we contacted corresponding authors to request additional data for the meta-analysis. |
| **Reporting of methods should include** | |  |
| √ | Description of relevance or appropriateness of studies assembled for assessing the hypothesis to be tested | Detailed inclusion and exclusion criteria are described in the methods section. |
| √ | Rationale for the selection and coding of data | Data extracted from each of the studies were related to the population characteristics, study design, exposure, outcome, and effect of confounders, mediators and moderators reported in included studies. |
| √ | Assessment of confounding | Meta-regressions were used to examine the quantitative influence of several pre-defined variables and the quality of studies. Additionally, we qualitatively estimate the influence of confounders in the association between childhood maltreatment and resilience domains. |
| √ | Assessment of study quality, including blinding of quality assessors; stratification or regression on possible predictors of study results | We adapted the Newcastle-Ottawa Scale for the evaluation of non-randomised studies. This tool has been adopted in recent meta-analyses. |
| √ | Assessment of heterogeneity | Heterogeneity was assessed using the Q-test, *I^2^* index, and Tau square. Confidence intervals and prediction intervals were also reported. |
| √ | Description of statistical methods in sufficient detail to be replicated | Random effect meta-analyses. Description of methods of meta-analyses, sensitivity analyses, meta-regressions, subgroup analyses, and assessment of publication bias are fully detailed in the methods. |
| √ | Provision of appropriate tables and graphics | We included the PRISMA flow-chart and several tables to describe the literature search and results. Several tables and forest/funnel plots, and figures were used to describe the main findings of the analyses. |
| **Reporting of results should include** | |  |
| √ | Graph summarising individual study estimates and overall estimate | We have appended an illustrative table (Table 2) in the main text. Additional tables were presented as supplementary material to fully describe the results. |
| √ | Table giving descriptive information for each study included | Table 1. |
| √ | Results of sensitivity testing | Sensitivity analyses (one-study-removal) are reported in the main text and plots are appended in the supplementary results. |
| √ | Indication of statistical uncertainty of findings | We report mean estimates for the main outcome, pooled 95% CIs, and prediction intervals. |
| **Reporting of discussion should include** | |  |
| √ | Quantitative assessment of bias | Descriptions of quantitative assessment of bias are detailed in the methods; results are described in the main text, and funnel plots are appended in the supplementary materials. |
| √ | Justification for exclusion | Exclusion criteria regarding publication type, measures for childhood maltreatment and resilience, the presence of treatment outcomes, a relationship between childhood maltreatment and resilience in adults (or descriptive statistics that allow calculation of associations), or any meta-analytical data provided, are detailed in methods, and presented in the discussion section. |
| √ | Assessment of quality of included studies | The assessment of quality of included studies, using the Newcastle-Ottawa Quality Assessment Scale (NOS) is detailed in the supplement, and results of the quality assessment entered in meta-regression analyses are presented in the discussion section. |
| **Reporting of conclusions should include** | |  |
| √ | Consideration of alternative explanations for observed results | We discussed alternative explanations for our findings, specifically considering potential methodological shortcomings. |
| √ | Generalisation of the conclusions | We have clearly addressed the generalisation of the conclusions in the discussion section. |
| √ | Guidelines for future research | We have suggested possible streams of future research in the discussion. |
| √ | Disclosure of funding source | We added a funding disclosure for the undertaking of this systematic review and meta-analysis. |

# ST4. Description and measurement of global/trait resilience and domains

| **Global/trait resilience** | |
| --- | --- |
| ***Definition*** Resilience describes successful adaptation in the face of adversity, commonly inferred from trajectories of well-being following major life events. Alternatively, resilience is conceptualised as a psychological trait, facilitating adaptation through stable individual characteristics. As a personal trait, resilience is a relatively stable, innate characteristic that is featured by psychological hardiness, ego resilience, and coping efficacy (Blanke et al., 2023; Connor & Davidson, 2003). | |
| **Instrument / Measurement** | **Description** |
| Adult Resilience Measure (ARM) | The ARM is a self-report measure of resilience, a 28-item scale that seeks to measure protective resources across individual, relational, and contextual subscales. |
| Brief Resilience Scale (BRS-6-item) | The BRS is a self-report measure of resilience, a 6-item scale designed to measure resilience as the ability to bounce back from stress (Smith et al., 2008). |
| Connor-Davidson Resilience Scale (CD-RISC) | The CD-RISC is a self-administered test that measures resilience or how well one is equipped to bounce back after stressful events, tragedy, or trauma, including the ability to adapt to change, the ability to deal with what comes along, the ability to cope with stress, the ability to stay focused and think clearly, the ability to not get discouraged in the face of failure, and the ability to handle unpleasant feelings such as anger, pain or sadness. The CD-RISC comprises of 25 items, each rated on a 5-point scale (0-4), with higher scores reflecting greater resilience(Connor & Davidson, 2003). |
| Psychological Resilience Scale-Short form (RS-15) | The Psychological Resilience scale was developed based on the theory that an overarching construct unites personality traits indicating psychological resilience, protecting individuals in the face of adversity and leading to positive adaptive behaviour. This self-report instrument is used to measure psychological resilience. The 15 items are answered using a Likert scale ranging from 1 (agree) to 7 (disagree) (Neill & Dias, 2016). |
| Suicide Resilience Inventory (SRI-25) | The SRI-25 is a self-report to assesses factors that help defend against suicidal thoughts and behaviours (Osman et al., 2004). |
| Resilience Scale for Adults (RSA) | The RSA measures the presence of protective resources that promote adult resilience. The RSA consists of 45 items covering five dimensions: personal competence, social competence, family coherence, social support, and personal structure (Friborg et al., 2003). |
| Resilience scale (RS-25) | The RS-25 is a standardised 25-item self-report assessment tool that measures the degree of individual resilience, focusing on positive psychological characteristics instead of deficits. Participants are required to rate, using a 7-point Likert item, how much they agree or disagree with the statements and how much they identify with them. Higher scores reflect higher levels of resilience (Wagnild & Young, 1993). |
| The Defense Style Questionnaire (DSQ) | The Defense Style Questionnaire is the first questionnaire to reliably describe defense styles. The 72-item DSQ is a self-report instrument designed to measure defensive functioning and coping styles (Andrews et al., 1993). |
| **Resilience domains** | |
| **1) Coping** | |
| **Definition:** Coping is defined broadly as an effort used to minimise distress associated with negative life experiences, involving conscious, volitional efforts to regulate emotion, cognition, behaviour, physiology, and the environment in response to stress (Bonanno et al., 2011, 2015). | |
| **Instrument / Measurement** | **Description** |
| The Coping Wheel | The Coping Wheel is a semi-projective open-ended instrument consisting of a circle with 12 equal-sized segments that captures the extent to which the individual feels that they can control different factors of their life, in relation to their perceived importance, and positive or negative evaluation. The investigator gives general, non-detailed instructions and guides the individual through the test. The scores range from −36 to +36, and a higher score indicates better coping (Blomkvist et al., 2002; Shalit & Carlstedt, 1984). |
| Coping Inventory for Stressful Situation (CISS) | The CISS is a trait-like measure of general coping styles. This self-report scale measures the multidimensional aspects of coping: task-oriented strategies (16 items), emotion-oriented strategies (16 items), and avoidance strategies (16 items). Respondents indicate how often, on a 5-point scale (1 = not at all, to 5 = a lot), they use each of the coping strategies presented. Higher scores indicate greater use of emotion-oriented and avoidant coping strategies (Endler & Parker, 1994; Rafnsson et al., 2006). |
| Coping Orientation for Problem Experiences (COPE) | The COPE is a 60-item self-report multidimensional coping inventory to assess the different ways in which people respond to stress. Five scales measure conceptually distinct aspects of problem-focused coping; five scales measure aspects of emotional-focused coping; and three scales measure ineffective coping responses. Response options for the 60 items include: 1= I never do such a thing; 2=I rarely do such a thing; 3=I occasionally do such a thing; 4=I often  do such a thing. Each of the fifteen subscales consist of four items inquiring about a different coping attitude. COPE subscales include: 1. Positive reinterpretation and growth (positively review, assess the behaviour which has been displayed against stress), 2. Mental disengagement (giving up thinking about the problem), 3. Focus on and venting of emotions (expressing feelings), 4. Seeking social support for instrumental reasons (calling for suggestions, help or information), 5. Active coping (concentrating efforts on doing something about the stress), 6. Denial (denying the existence of stress), 7. Turning  to religion, 8. Humour (mocking/joking about the situation), 9. Behavioural disengagement (reduction of coping efforts), 10. Restraint coping (waiting for the appropriate opportunity to act), 11. Seeking emotional social support (receiving moral support), 12. Substance use, 13. Acceptance, 14. Suppression  of competing activities (putting aside other activities in order to concentrate on the problem), and 15. Planning (thinking hard about coping with the stress). Higher scores obtained from the subscales provide the opportunity to comment on which coping attitude is used more frequently by the individual (Carver et al., 1989). |
| Brief Coping Orientation for Problem Experiences (COPE) | The Brief-COPE is a shortened self-report version of the instrument above, consisting of 28 items designed to measure effective and ineffective ways to cope with a stressful life event. The instrument measures a total of 14 coping strategies when faced with adversity or stressful situations. The items are classified into the following categories: problem-focused coping strategies (active coping, instrumental support, and plan); emotion-focused coping strategies (acceptance, emotional support, humour, positive reframing, and religion); and dysfunctional coping strategies (venting, behavioural disengagement, self-distraction, self-blame, substance use, and denial). Higher scores obtained from the subscales provide the opportunity to determine which coping attitude is used more frequently by the individual (Carver, 1997). |
| Ways of Coping Questionnaire (WCQ) | The Ways of Coping Questionnaire (WCQ) is a widely used self-report measure of coping processes. It comprises a set of 45 items, in which the frequency of use of different coping strategies is presented in a Likert format, from 1 (“I never do this”) to 5 (“I always do this”). The participant is asked to select a  specific stress-related event and, using a 5-point Likert type scale, indicate how they would respond to that event. The coping strategies are divided into four factors: (1) focused on the problem (i.e., active efforts to manage, cope, solve, or reappraise the problem), (2) focused on emotion (i.e., efforts to regulate the emotional states associated with the stressor as a way to reduce emotional discomfort without the objective to solve the problem; these include emotional reactions, such as rage, anxiety, guilt, avoidance, and passive behaviour), (3) religious or fantastic thought (i.e., religious behaviour, thoughts, and faith that help when coping with problems), and (4) search for social support (i.e., actively search for information or emotional support). Higher scores indicate greater use of a particular coping strategy (Folkman et al., 1986). |
| The Brief Religious Coping Activities Scale (Brief RCOPE) | The Brief Religious Coping Activities Scale (RCOPE) is a 14-item self-report instrument that measures how people use religion to cope with major life stressors. Items assess two styles of religious coping: positive and negative. Positive coping items include items such as “I looked for a stronger connection with God.” Negative religious coping methods include items such as “I wondered whether God had abandoned me.” The possible score range is 3–14, with 3 indicating low religious coping and 14 indicating high religious coping (Pargament et al., 1998). |
| Simplified Coping Style Questionnaire (SCSQ) | The 20-item Simplified Coping Style Questionnaire is a self-report measure of stress responses based on the Ways of Coping Questionnaire (Folkman et al., 1986). It includes two dimensions: problem-oriented/positive coping and emotion-oriented/negative coping. The SCSQ has 20 items on a 4-point Likert scale. Higher scores indicate greater use of a particular coping strategy (Xie, 1998). |
| 2012 Canadian Community Health Survey-Mental Health (CCHS-MH 2012) | The purpose of the Canadian Community Health Survey - Mental Health (CCHS - Mental Health) is to collect information about mental health status, access to and perceived need for formal and informal services and supports, functioning and disability, and covariates. It is a national cross-sectional population-based survey conducted by Statistics Canada (Arslan & Genç, 2022). |
| Spirituality Survey (SS-1) from the Study on Stress, Spirituality, and Health (SSSH) | The SS-1 consists of 82 R/S items assessing the following areas: Religious activities; Closeness to God; Religious coping (positive religious coping and negative religious coping/spiritual struggles); Gratitude and Non-theistic daily spiritual experiences. Religious service attendance was coded “never” =0 to “several times per week” =6. Positive religious coping was an average of 8 items (e.g., “I saw my situation as part of God’s plan”), and negative religious coping was an average of six items (e.g., “I wondered what I did for God to punish me”) (Pargament et al., 2000). Responses ranged from “not at all” =1 to “a great deal” =4. Gratitude was an average of two items (“I have so much in life to be thankful for” and “If I had to list everything, I felt thankful for, it would be a very long list”) (McCullough et al., 2002). Response options ranged from “strongly disagree” = 1 to “strongly agree” =5. Non-theistic daily spiritual experiences (NTDSE) consisted of an average of four items (e.g., “I experience a connection to all of life”) and theistic daily spiritual experiences (TDSE) consisted of an average of two items (e.g., “I desire to be closer to God”). Response options ranged from “never”=0 to “many times a day”=5 for NTDSE and from “definitely not true of me”=0 to “definitely true of me”=5 for TDSE (Warner et al., 2021). |
| **2) Self-esteem** | |
| **Definition:** Self-esteem is defined as one’s overall sense of self-worth or personal value and represents one’s comprehensive evaluation of oneself, including positive and negative evaluations (Brown et al., 2001). | |
| **Instrument / Measurement** | **Description** |
| Beck Self-Esteem Scale (BSE) | The BSE is an 18-item self-report instrument that measures an individual's self-esteem by evaluating their beliefs and attitudes about themselves, including their sense of self-worth and self-acceptance. The BSE uses a 4-point Likert scale, with response options ranging from 0 to 3. Higher scores indicate greater self-esteem (Beck et al., 2001, 2013). |
| Culture-Free Self-Esteem Inventory (CFSEI-2) | The CFSEI-2 is a self-report instrument designed to measure self-esteem in children and adults. The items are formatted as Yes and No questions (dichotomous response format), and the inventory consists of 60 items. The scales assess various aspects of self-esteem, including general self-esteem, social self-esteem, and academic self-esteem. It includes parent-related self-esteem and a lie scale to identify socially desirable response tendencies. Higher scores on the respective scales indicate a higher level of self-esteem in those areas (Holaday et al., 1996). |
| Coopersmith Self-Esteem Inventory (CSEI) | The CSEI is a self-report questionnaire consisting of 25 items. It uses a dichotomous response format (Yes or No). The results can be divided into various dimensions of self-esteem, including general self-esteem, social self-esteem, academic self-esteem, and parent- or family-related self-esteem. Higher scores in the respective dimensions indicate higher levels of self-esteem (Coopersmith, 2012). |
| Rosenberg Self-Esteem Scale (RSES) | The RSES measures an individual's overall self-esteem, evaluating their positive and negative perceptions of themselves through a series of statements rated on a four-point Likert scale. The scale consists of 63 items divided into 12 subgroups and is a self-report questionnaire. Respondents indicate the degree to which they agree with each statement, with response options ranging from strongly disagree (1) to strongly agree (4). The scale includes both positively and negatively worded items to minimise response biases. Scores are calculated by summing the responses. Positively worded items are scored directly. Negatively worded items are reverse scored. Higher scores indicate a greater level of self-esteem (Gnambs et al., 2018). |
| Tennessee Self Concept Scale (TSCS) | The TSCS is composed of 70 self-descriptive statements that include three subscales: self-identity, satisfaction, and behaviour. It uses a 5-point Likert scale, where respondents indicate the extent to which they agree with a statement, ranging from "strongly disagree" to "strongly agree." The statements are designed to measure various aspects of self-concept, including both positive and negative self-perceptions. The TSCS is a self-report questionnaire to assesses a global self-concept and specific dimensions, such as physical self-concept, moral self-concept, social self-concept, emotional self-perception, and academic as well as intellectual self-concept. Higher scores indicate a more positive self-concept in the respective dimension (Marsh & Richards, 1988). |
| Self-esteem Scale (SES) | The SES, based on the TSCS, is an instrument specifically designed to measure self-esteem as a subset of self-concept. While the TSCS covers a broader range of self-concept dimensions, the SES focuses on assessing general and specific self-esteem. A 5-point Likert scale is used to measure the intensity of agreement with statements. The SES is a self-report questionnaire evaluating global self-esteem. Higher scores indicate a higher level of self-esteem, whereas lower scores may suggest insecurity or low self-esteem (Robson, 1988, 1989). |
| Taylor Self-Esteem Inventory (TSEI) | The TSEI is a psychological instrument designed to assess self-esteem. It typically uses an 8-point Likert scale, where respondents indicate the extent to which they agree or disagree with specific statements. It is a self-report questionnaire consisting of 16 items. Higher scores indicate a higher level of self-esteem, while lower scores may suggest lower self-esteem. The TSEI measures an individual's self-esteem by assessing their self-perception and self-worth through a series of statements, providing insights into overall self-esteem levels (Jones, 1996). |
| **3) Emotion Regulation** | |
| **Definition:** the process by which individuals influence the occurrence, timing, nature, experience, and expression of their emotions, involving conscious or unconscious strategies or processes to monitor, evaluate, modulate, modify and manage the intensity, form, duration, physiological states and behaviours associated with emotional arousal and expression (Kok, 2020). | |
| **Instrument / Measurement** | **Description** |
| Brief Self Control Scale (BSCS) | The Brief Self Control Scale (BSCS) is an abbreviated 13-item self-report questionnaire based on the 36-item Self Control Scale. The BSCS uses a five-point scale to assess self-control (self-discipline, deliberative/no impulsive action, healthy habits, work ethic, reliability) and yields scores ranging from 13–65 (Tangney et al., 2004). |
| Cognitive Emotion Regulation Questionnaire (CERQ) | The cognitive Emotion Regulation Questionnaire (CERQ) is a 36-item self-report questionnaire assessing nine cognitive strategies of emotion regulation (self-blame, blaming others, acceptance, refocus on planning, positive refocusing, rumination or focus on thought, positive reappraisal, putting into perspective, catastrophising) and generating scores from 36–180 (Garnefski et al., 2001). |
| Difficulties in Emotion Regulation Scale (DERS) | The Difficulties in Emotion Regulation Scale (DERS) is a 36-item self-report questionnaire to assess problems in adaptively using appropriate emotion regulation strategies. The six subscales comprise nonacceptance of emotional responses, difficulty engaging in goal-directed behaviour, impulse control difficulties, lack of emotional awareness, limited access to emotion regulation strategies and lack of emotional clarity. Scores range from 36–180, with higher scores indicating greater emotion regulation problems (Gratz & Roemer, 2004). |
| Emotion Dysregulation Scale – Short (EDS) | The Emotion Dysregulation Scale – Short (EDS) is a 12-item self-report questionnaire assessing emotional experiencing, cognition and behaviour. Scores range from 12–84, with higher scores indicating greater emotion regulation difficulties (Powers et al., 2015). |
| Emotion Regulation Questionnaire (ERQ) | The Emotion Regulation Questionnaire (ERQ) is a 10-item questionnaire assessing two domains of emotion regulation (suppression and reappraisal). A 7-item scale is used and generates scores ranging from 10–70 (Gross & John, 2003). |
| Feelings and Me (FAM) | The Feeling and Me (FAM) questionnaire is a 54-item self-report survey assessing both adaptive and maladaptive cognitive, behavioural/instrumental, interpersonal and somatosensory responses employed to alleviate sadness and distress. Items are rated on a scale from 0–2 with scores for the 30 items measuring adaptive regulatory responses ranging from 0–60 and the 24 items measuring maladaptive regulatory responses ranging from 0–48 (Kovacs et al., 2009). |
| Negative Mood Regulation Scale (NMR) | The Negative Mood Regulation Scale (NMR) is a 30-item self-report questionnaire that assesses personal expectations of the ability to regulate negative mood via behaviour or cognition mood regulation ability. Items assessing negative mood regulation expectancies and mood regulation strategies are scored on a 5-point scale yielding total scores ranging from 30–150 (Catanzaro & Mearns, 1990). |
| Urgency, Premeditation, Perseverance, Sensation seeking, and Positive urgency Impulsive Behaviour Scale (UPPS-P) | The Urgency, Premeditation, Perseverance, Sensation seeking and Positive urgency Impulsive Behaviour Scale (UPPS-P) is a 59-item measure of impulsive behaviour (positive urgency, negative urgency, sensation seeking, lack of premeditation, lack of perseverance). Items are scored on a scale from 1–4, yielding a total score ranging from 59–236 (Lynam et al., 2006). |
| **4) Self-efficacy** | |
| **Definition** Self-efficacy is one’s overall sense of self-worth or personal value that represents one’s comprehensive evaluation of oneself, including positive and negative evaluations, and a person's belief in their ability to complete a task or achieve a goal (Bandura, 1982). | |
| **Instrument / Measurement** | **Description** |
| The General Self-Efficacy Scale (GSES) | The General Self-Efficacy Scale (GSE) is a 10-item self-report instrument created to assess a general sense of perceived self-efficacy with the aim in mind to predict coping with daily hassles as well as adaptation after experiencing all kinds of stressful life events. Participant responses range from 1 (Always false) to 4 (Always true). Higher scores are indicative of high level of self-efficacy (Caikang, 2001; Schwarzer, R. & Jerusalem, M., 1995; Zhang & Schwarzer, 1995). |
| The Regulatory Emotional Self-Efficacy Scale (RESE) | The RESE is a 12-item scale developed to assess perceived self-efficacy in managing negative (NEG) and in expressing positive (POS) affect. The Chinese version has 17 items and supports a second-order five-factor model including perceived self-efficacy in expressing positive affect (POS-RESE) and perceived self-efficacy in managing negative affect (NEG-RESE). Ratings are a 5-point scale from 1 (not at all like me) to 5 (completely like me). Higher scores indicate greater self-efficacy (Caprara & Gerbino, 2001; Shufeng et al., 2009). |
| Pearlin Mastery (PM) Scale | The PM scale is a self-report instrument to measure the extent to which an individual regards their life chances as being under their personal control rather than fatalistically ruled. Respondents are presented with seven items that included statements such as “You have little control over the things that happen to you” and “What happens to you in the future mostly depends on you.” Response categories range from “strongly agree” to “strongly disagree” on a 5-point scale. All responses are coded such that higher values equate to greater self-efficacy (Pearlin & Schooler, 1978). |
| 12-item Self-Efficacy Scale for Battered Women (SESBW) | The SESBW is a 27-item 100-mm visual analogue scale designed to measure an self-efficacy in women exposed to abuse. The scale taps engagement in adaptive help-seeking behaviours and adaptive living skills. Items are ranked from 0 (*couldn’t do it at all*) to 100 (*completely sure I could do it*) and summed to get a rating of self-efficacy, with higher scores indicative of greater self-efficacy (May & Limandri, 2004). |
| The Self-Efficacy Scale | The Self-Efficacy Scale includes 23 self-report statements, 17 of which assess general self-efficacy (e.g., “when I make plans, I am certain they will be executed”) and six of which assess social self-efficacy (e.g., “I find it difficult to make new friends”). In addition, the scale includes seven filler statements that are not calculated in the score for the self-efficacy variable (e.g., “there’s something good in everyone”). Responses are based on a scale ranging from 1(*completely disagree*) to 5 (*completely agree*). The overall score is calculated as the mean score on all 23 items. A high score indicates a high degree of self-efficacy (Sherer et al., 1982). |
| CD-RISC Tenacity and Personal Competence sub-scale | The CD-RISC is a self-administered test that measures resilience or how well one is equipped to bounce back after stressful events, tragedy, or trauma, including the ability to adapt to change, the ability to deal with what comes along, the ability to cope with stress, the ability to stay focused and think clearly, the ability to not get discouraged in the face of failure, and the ability to handle unpleasant feelings such as anger, pain or sadness. The CD-RISC comprises 25 items and participants rate each item on a 5-point Likert-type scale (0–4). The scale consists of three factors: tenacity and personal competence, tolerance of negative affect, and spiritual influences. Scores range from 0–100, with higher scores indicative of higher psychological resilience (Connor & Davidson, 2003). |
| **5) Well-being** | |
| **Definition:** A broad psychological and social construct that encompasses life satisfaction, higher positive affect and lower negative affect (Diener, 2000) and biological and psychological qualities that enable successful adaptation or swift recovery from life adversity, such as sense of mastery, optimism, or sense of coherence, the experience of positive emotions, and having a purpose in life (Rutten et al., 2013). | |
| **Instrument / Measurement** | **Description** |
| General Health Questionnaire -12 (GHQ-12) | The General Health Questionnaire (GHQ-12) is a 12-item screening measure for common mental disorders, as well as general psychiatric well-being. Items are rated on a 4-point scale with total scores varying based on whether bimodal, Likert or C-GHQ scoring approaches are used (Goldberg et al., 1997). |
| Meaning and Purpose in Life Scale (MPLS) | The Meaning and Purpose in Life Scale (MPLS) is a 17-item scale measuring the degree to which individuals attach meaning, based on subjective experience and purpose, to their lives. The items are scored on a 5-point scale yielding total scores ranging from 17–85 (Aydin et al., 2015). |
| Meaning in Life Questionnaire (MLQ) | The Meaning in Life Questionnaire (MLQ) is a 10-item questionnaire measuring the sense made of one’s being and existence, as well as the perceived significance of this i.e., the presence of and search for meaning. The items are scored on a 7-point scale yielding total scores ranging from 10–70 (Steger et al., 2006). |
| Mental Health Continuum Short Form (MHC-SF) | The Mental Health Continuum Short Form (MHC-SF) is a 14-item measure of emotional (happiness, interest in life, satisfaction), psychological (autonomy, environmental mastery, personal growth, positive relations with others, purpose in life, self-acceptance) and social well-being (social coherence, social integration, social contribution, social actualisation, social acceptance). The items are rated on a 6-point scale yielding total scores ranging from 0–70 (Keyes, 2009). |
| Scales of Psychological Well-Being (SPWB) | The Ryff’s Scales of Psychological Well-Being comprised 20 items measuring six dimensions of psychological well-being (autonomy, environmental mastery, personal growth, positive relations with others, purpose in life, self-acceptances). Items are rated on a scale of 1–6 to yield total scores ranging from 20–120 (Ryff & Keyes, 1995). |
| Scales of Psychological Well-Being – Medium Form (SPWB – 54) | The Ryff’s Scales of Psychological Well-Being – Medium Form comprises 54 items measuring six dimensions of psychological well-being (autonomy, environmental mastery, personal growth, positive relations with others, purpose in life, self-acceptances). Items are rated on a scale of 1–6 to yield total scores ranging from 54–324 (Ryff, 1989). |
| Satisfaction with Life Scale (SWLS) | The Satisfaction with Life Scale (SWLS) is a 5-item measure that measures global satisfaction with life as an aspect of subjective well-being. Items are rated on a 7-point scale to yield total scores ranging from 7–35 (Diener et al., 1985). |
| Sense of Coherence Scale – 13 (SOC-13) | The Sense of Coherence Scale (SOC-13) is a 13-item measure of the perceptions of the world and stressors that facilitate coping (comprehensibility, manageability and meaning of life). The items are scored on a 7-point scale to yield total scores ranging from 13–91 (Antonovsky, 1993). |
| Spiritual Well-Being Scale (SWBS) | The Spiritual Well-Being Scale is a 20-item instrument designed to assess religious and existential well-being in both groups and individuals. Each item is scored on a 6-point Likert scale to yield total scores ranging from 20–120 (Paloutzian & Ellison, 1991). |
| Subjective Well-being Inventory (SUBI) | The Subjective Well-being Inventory (SUBI) is a 40-item measure that covers eleven factors related to feelings of well-being and ill-being (general well-being/positive affect, expectation-achievement congruence, confidence in coping, transcendence, family group support, social support, primary group concern, inadequate mental mastery, perceived ill-health, deficiency in social contacts, general well-being/negative affect). Items are rated on a 3-point scale to yield total scores ranging from 40–120 (World Health Organisation, 1992). |
| Warwick-Edinburgh Mental Well-Being Scale (WEMWBS) | The Warwick-Edinburgh Mental Well-Being Scale (WEMBS) is a 14-item self-report measure that examines positive mental health (positive affect, interpersonal relationship satisfaction and positive functioning). Each item is scored on a 5-point scale to yield total scores ranging from 14–70 (Tennant et al., 2007). |
| World Health Organisation-Five Well-Being Index (WHO-5) | The World Health Organisation-Five Well-Being Index (WHO-5) is a 5-item self-report measure of mental well-being within the prior two weeks. Items are scored on a 0–5 scale yielding total scores ranging from 0–25 (World Health Organisation, 2024). |

# ST5. Quality assessment of included studies

The adapted version of the Newcastle-Ottawa Scale (NOS) for non-randomised studies was used to assess the quality of included studies. Details on this scale can be found in the NOS scoring section in ST7. Studies were assessed based on three broad domains: 1) Selection of participants (representativeness of exposed samples; sample size, non-response rate or non-responders compared, and ascertainment of exposure; 2) Comparability involving appropriate control for confounders; 3) Assessment of outcome based on validated scales, and statistical analysis clearly described and allowing associations to be calculated.

The maximum number of scores for each domain was 4 for Selection, 2 for Comparability, and 2 for Outcome. Total quality scores ranged from 0 to 8, with a higher score representing better quality. We rated the overall quality according to specific combinations of results across the three domains, according to the scale adaptation for cross-sectional studies (Modesti et al., 2016). Studies scoring 5 points, that is, 2 points in selection, 1 point in comparability, and 2 points in outcome were considered as “fair” quality. Studies scoring 6, that is, 3 points in Selection, 1 point in Comparability, and 2 points in Outcome were classified as “good” quality. “High” quality studies scored > 6, that is, 3 or 4 points in Selection, 1 or 2 points in Comparability, and 2 points in Outcome (see definitions of each domain and scoring of the NOS assessment in SA5 and ST5, and the quality assessment of the included studies below in ST7).

The representativeness of samples was mixed, and most included studies did not report on non-response rates, or *a priori* or *post hoc* power analyses or other sample size justifications. A wide range of confounders were considered in 62 (30.54%) included studies, such as age, sex or gender, race/ethnicity, education level, income, marital/employment status, family risks, drug use, mood symptoms, affective temperament, and/or adulthood trauma (see Table 1). Many studies did not fully report results from statistical analyses conducted, e.g., omitting named effect estimates, *p* values, or measures of precision, if appropriate (such as standard errors or confidence intervals).

## **Newcastle-Ottawa scale (NOS) for non-randomised studies**

***Selection (maximum 4 stars)***

**1. Representativeness of the sample**

a. Truly representative of the average in the target population (all subjects or whole population, random sampling). *

b. Somewhat representative of the average in the target population (evidence that the sample is representative of the source population, non-random sampling). *

c. Selected group of users.

d. No description of the sampling strategy.

**2. Sample size**

a. Justified or satisfactory. *

b. Not justified.

**3. Non-respondents**

a. Comparability between respondents and non-respondents characteristics is established, or the response rate is satisfactory (> 60%) *

b. The response rate is unsatisfactory, or the comparability between respondents and non-respondents is unsatisfactory.

c. No description of the response rate or the characteristics of the responders and non-responders.

**4. Ascertainment of the exposure** (childhood maltreatment)

a. Validated measurement tool. *

b. Non-validated measurement tool, or not described.

***Comparability (maximum 2 stars)***

**5. The subjects in different outcome groups are comparable,**

**based on the study design** **or analysis** (confounding factors are controlled).

a. The study controls for sex/gender (or analysis separated by sex/gender). *

b. Study controls for any additional factor. *

c. The study does not adjust for any confounding factor.

***Outcome (maximum 2 stars)***

**6. Assessment of the outcome** (resilience)

a. The study uses reliable and validated instruments as confirmed by reported psychometric measures (questionnaires). *

b. No description.

**7. Statistical test**

a. The statistical test used to analyse the data is clearly described and appropriate, and the measurement of the association is presented, including confidence intervals and the probability level (*p* value). *

b. Study reports descriptive statistics that allows calculating associations. *

c. The statistical test is not appropriate, not described or incomplete.

***Note:***  In the Newcastle-Ottawa Scale (NOS) (Wells et al., 2014) adapted and validated (Epstein et al., 2018; Herzog et al., 2013; Mertz et al., 2013; Wickersham et al., 2020) for non-randomised studies (cross-sectional and longitudinal)*,* a study can be awarded a maximum of one point (star) for each numbered item within the Selection and Exposure categories. A maximum of two points (stars) can be given for Comparability.

| ***NOS scoring*** | **Maximum score = 8** |
| --- | --- |
| **Selection** | **4** |
| Sample representative of target sample (e.g., all eligible or random sample)? | 1 |
| Sample size justified or satisfactory? | 1 |
| Non-response rate is defined satisfactory, and characteristics of responders/non-responders compared? | 1 |
| Ascertainment of exposure (i.e., childhood maltreatment) is valid and/or well described? | 1 |
| **Comparability** | **2** |
| Controls for sex/gender | 1 |
| Controls for any additional factor | 1 |
| **Outcome** | **2** |
| Assessment of outcome with validated tool? | 1 |
| Statistical test clearly described and appropriate, and/or descriptive statistics that allows calculating associations? | 1 |

## **Results of the quality assessment of included studies**

| **Study** | **Selection** | **Comparability** | **Outcome** | **NOS Total** |
| --- | --- | --- | --- | --- |
| Allbaugh et al., 2017 | ** |  | ** | 4 |
| Anctil et al., 2007 | ** | ** | ** | 6 |
| Armitage et al., 2021 | *** | * | ** | 6 |
| Arslan & Genç, 2022 | *** |  | ** | 5 |
| Arslan et al., 2015 | * |  | ** | 3 |
| Artime et al., 2012 | ** |  | ** | 4 |
| Babad et al., 2022 | *** | ** | ** | 7 |
| Berhe et al., 2023 | **** | ** | ** | 8 |
| Berzenski & Yates, 2010 | ** | * | ** | 5 |
| Berzenski, 2019 | **** | * | ** | 7 |
| Billen et al., 2022 | ** | * | ** | 5 |
| Blood & Blood, 2016 | ** | ** | ** | 6 |
| Bouchard & Sonnier, 2023 | *** | ** | ** | 7 |
| Bradley et al., 2005 | ** | * | ** | 5 |
| Brodski & Hutz, 2012 | * | * | ** | 4 |
| Broekhof et al., 2015 | **** | ** | ** | 8 |
| Bungert et al., 2015 | ** | * | ** | 5 |
| Burns et al., 2010 | *** |  | ** | 5 |
| Cantón-Cortés et al., 2012 | ** |  | ** | 4 |
| Cao et al., 2022 | ** | * | ** | 5 |
| Cao et al., 2023 | *** |  | ** | 5 |
| Carvalho et al., 2014 | * | ** | ** | 5 |
| Cecen & Gümüş, 2024 | *** | ** | ** | 7 |
| Çelik & Odaci, 2020 | *** |  | ** | 5 |
| Chang et al., 2023 | *** |  | ** | 5 |
| Chaturvedi & Arya, 2023 | **** | * | ** | 7 |
| Chen et al., 2023 | *** |  | ** | 5 |
| Cheng & Langevin, 2023 | **** | ** | ** | 8 |
| Chi et al., 2021 | **** | ** | ** | 8 |
| Choe et al., 2021 | ** | ** | ** | 6 |
| Choi et al., 2014 | * | * | ** | 4 |
| Christ et al., 2019 | **** | * | ** | 7 |
| Clark et al., 2021 | ** | * | ** | 5 |
| Cloitre et al., 2008 | * | * | ** | 4 |
| Costa et al., 2024 | * | * | ** | 4 |
| Crosta et al., 2018 | **** |  | ** | 6 |
| Daniels et al., 2012 | * |  | ** | 3 |
| Daruy-Filho et al., 2013 | * | * | ** | 4 |
| Davies et al., 2004 | * | * | ** | 4 |
| Dawson et al., 2022 | *** | ** | ** | 7 |
| Demir et al., 2020 | * | ** | ** | 5 |
| Dereboy et al., 2018 | **** | * | ** | 7 |
| Di Nicola et al., 2024 | ** | ** | ** | 6 |
| Ekinci & Kandemir, 2015 | *** | ** | ** | 7 |
| ElBarazi, 2023 | ** |  | ** | 4 |
| Endo et al., 2024 | ** |  | ** | 4 |
| Erol & Inozu, 2023 | *** |  | ** | 5 |
| Feinauer et al., 1996 | ** |  | * | 3 |
| Fereidooni et al., 2023 | **** |  | ** | 6 |
| Festinger & Baker, 2009 | ** | ** | ** | 6 |
| Fitzgerald & Barton, 2022 | **** | ** | ** | 8 |
| Fitzgerlad & Esplin, 2023 | *** | ** | ** | 7 |
| Fleming et al., 1999 | *** | * | ** | 6 |
| Fossati et al., 2015 | *** | ** | ** | 7 |
| Fosse & Holen, 2007 | *** | ** | ** | 7 |
| Fox et al., 1994 | ** | * | ** | 5 |
| Galea et al., 2007 | ** |  | ** | 4 |
| Gambaro et al., 2020 | *** |  | ** | 5 |
| Garcia & Berzenski, 2023 | *** |  | ** | 5 |
| Garofalo et al., 2024 | **** |  | ** | 6 |
| Goldbach et al., 2023 | *** |  | ** | 5 |
| Goldstein et al., 2013 | * | ** | ** | 5 |
| Goodboy et al., 2016 | * |  | ** | 3 |
| Griffing et al., 2006 | * |  | ** | 3 |
| Güler et al., 2023 | *** |  | ** | 5 |
| Guo et al., 2022 | *** | ** | ** | 7 |
| Haj-Yahia et al., 2021 | *** |  | ** | 5 |
| He et al., 2022 | ** |  | ** | 4 |
| Hengartner et al., 2013 | ** | * | ** | 5 |
| Herrenkohl et al., 2012 | *** | ** | ** | 7 |
| Heshmati et al., 2021 | *** |  | ** | 5 |
| Higgins et al., 1994 | *** |  | ** | 5 |
| Hu et al., 2024 | *** |  | ** | 5 |
| Ion et al., 2023 | **** | * | ** | 7 |
| Janiri et al., 2021 | *** | ** | ** | 7 |
| Jennissen et al., 2016 | **** | ** | ** | 8 |
| Johnson, 2001 | * |  | ** | 3 |
| Jones et al., 2023 | *** | ** | ** | 7 |
| Jonzon & Lindblad, 2006 | * | * | ** | 4 |
| Kanai et al., 2016 | * |  | ** | 3 |
| Kanj et al., 2023 | *** | * | ** | 6 |
| Kapoor et al., 2018 | ** |  | ** | 4 |
| Karagöz & Dağ, 2015 | * | * | ** | 4 |
| Karakaş & Çingol, 2022 | **** |  | ** | 6 |
| Kazan Kizilkurt et al., 2021 | *** |  | ** | 5 |
| Kesebir et al., 2015 | ** |  | * | 3 |
| Khosravani et al., 2019 | *** | * | ** | 6 |
| Kim et al., 2016 | ** | ** | ** | 6 |
| Kim et al., 2021 | *** |  | ** | 5 |
| Kiziltepe et al., 2023 | *** | ** | ** | 7 |
| Koçak & Çağatay, 2024 | *** |  | ** | 5 |
| Kong et al., 2024 | *** |  | ** | 5 |
| Krause-Utz et al., 2023 | **** | * | ** | 7 |
| Krvavac & Jansson, 2021 | *** | * | ** | 6 |
| Kumar et al., 2022 | *** | ** | ** | 7 |
| Kuo et al., 2015 | ** |  | ** | 4 |
| Kurtuluş & Elemo, 2023 | *** |  | ** | 5 |
| Lacelle et al., 2012 | *** | * | ** | 6 |
| Laghaei et al., 2023 | **** |  | ** | 6 |
| Lassri et al., 2023 | ** |  | ** | 4 |
| Latzer et al., 2020 | *** | * | ** | 6 |
| Lewis et al., 2006 | * |  | ** | 3 |
| Li et al., 2020 | **** | ** | ** | 8 |
| Li et al., 2023 | *** | ** | ** | 7 |
| Li et al., 2023 | **** | ** | ** | 8 |
| Li et al., 2023 | ** |  | ** | 4 |
| Li et al., 2023 | **** | ** | ** | 8 |
| Liu et al., 2023 | **** |  | ** | 6 |
| Liu et al., 2024 | **** | ** | ** | 8 |
| Lu et al., 2017 | *** | ** | ** | 7 |
| Maftei & Nițu, 2024 | **** |  | ** | 6 |
| Mandavia et al., 2016 | **** | ** | ** | 8 |
| Martin et al., 2023 | **** |  | ** | 6 |
| Martínez et al., 2023 | *** | ** | ** | 7 |
| Martxueta et al., 2014 | * |  | ** | 3 |
| Maxwell & Huprich, 2014 | ** | * | ** | 5 |
| Merians & Frazier, 2023 | ** |  | ** | 4 |
| Mohammadpanah Ardakan et al., 2024 | ** |  | ** | 4 |
| Mohammadzadeh et al., 2019 | *** | * | ** | 6 |
| Mondolin et al., 2024 | *** | * | ** | 6 |
| Moreira et al., 2024 | * |  | ** | 3 |
| Musella et al., 2024 | ** |  | ** | 4 |
| Naderzadeh et al., 2023 | *** | ** | ** | 7 |
| Naughton et al., 2020 | **** | ** | ** | 8 |
| Newman et al., 2011 | ** | * | ** | 5 |
| Nimphy et al., 2024 | ** | ** | ** | 6 |
| Özakar Akça et al., 2021 | **** |  | ** | 6 |
| Pabian et al., 2022 | **** |  | ** | 6 |
| Park et al., 2023 | **** | * | * | 7 |
| Peng et al., 2020 | *** | ** | ** | 7 |
| Pourshahriar et al., 2018 | **** |  | ** | 6 |
| Qin et al., 2024 | ** |  | ** | 4 |
| Racine & Wildes, 2015 | * | * | ** | 4 |
| Richardson et al., 2023 | * |  | ** | 3 |
| Rodriguez et al., 2021 | * | * | ** | 4 |
| Romans et al., 1995 | ** |  | ** | 4 |
| Rong et al., 2023 | *** | ** | ** | 7 |
| Rostami et al., 2023 | *** |  | ** | 5 |
| Sachs-Ericsson et al., 2011 | *** | ** | ** | 7 |
| Salles et al., 2023 | ** |  | ** | 4 |
| Schulz et al., 2014 | ** | ** | ** | 6 |
| See Mey et al., 2022 | **** |  | ** | 6 |
| Sehlikoğlu et al., 2022 | ** |  | ** | 4 |
| Sexton et al., 2015 | *** | * | * | 5 |
| Sezer Katar et al., 2023 | ** |  | ** | 4 |
| Shen & Soloski, 2022 | *** | ** | ** | 7 |
| Shen, 2009 | **** | ** | ** | 8 |
| Shin & Brunton, 2024 | * |  | ** | 3 |
| Simeon et al., 2007 | * | ** | ** | 5 |
| Simon et al., 2009 | * | ** | ** | 5 |
| Sistad et al., 2021 | *** | ** | ** | 7 |
| Soffer et al., 2008 | *** |  | ** | 5 |
| Șoflău et al., 2023 | **** |  | * | 5 |
| Somers et al., 2017 | **** | ** | ** | 8 |
| Stevens et al., 2013 | ** | * | ** | 5 |
| Su et al., 2022 | *** |  | ** | 5 |
| Suresh & Tipandjan, 2012 | * | * | ** | 4 |
| Švecová et al., 2023 | **** | ** | ** | 8 |
| Swaminath et al., 2023 | *** | * | ** | 6 |
| Talmon et al., 2022 | *** | ** | ** | 7 |
| Tarber et al., 2016 | **** | * | ** | 7 |
| Theran & Han, 2013 | ** | * | ** | 5 |
| Thoma et al., 2021 | ** | ** | ** | 6 |
| Tinajero et al., 2020 | * | ** | ** | 5 |
| Toker et al., 2011 | ** | * | ** | 5 |
| Top & Cam, 2021 | **** |  | ** | 6 |
| Upenieks et al., 2024 | **** | ** | ** | 8 |
| Valencia & Rosa-Gómez, 2024 | ** |  | ** | 4 |
| Van Schie et al., 2024 | ** | ** | ** | 6 |
| Vancappel et al., 2023 | * |  | ** | 3 |
| Vetesse et al., 2011 | ** | ** | ** | 6 |
| Volgenau et al., 2022 | *** |  | * | 4 |
| Wadji et al., 2023 | *** | ** | ** | 8 |
| Walker et al., 2023 | ** | ** | ** | 6 |
| Walsh et al., 2011 | * | * | ** | 4 |
| Wang et al., 2022 | *** |  | ** | 5 |
| Wang et al., 2023 | **** |  | ** | 6 |
| Whittington (2023 | ** |  | ** | 4 |
| Wind et al., 1994 | ** | * | ** | 5 |
| Wolff et al., 2016 | ** | ** | ** | 6 |
| Wong et al., 2024 | *** | * | ** | 6 |
| Wu et al., 2022 | *** | ** | ** | 7 |
| Wu et al., 2023 | *** | * | ** | 6 |
| Xiang et al., 2018 | *** | * | ** | 6 |
| Xiang et al., 2020 | ** | * | ** | 5 |
| Xiang et al., 2021 | ** |  | ** | 4 |
| Xiao et al., 2023 | ** | * | ** | 5 |
| Xie et al. (2023 | ** | * | ** | 5 |
| Xu & Zeng, 2022 | **** | * | ** | 7 |
| Xu et al., 2023 | * | ** | ** | 5 |
| Yao et al., 2023 | **** |  | ** | 6 |
| Yaroslavsky et al., 2022 | ** | * | ** | 5 |
| Yilmaz & Satici, 2023 | ** | * | ** | 5 |
| Yöyen & Akyüz, 2023 | ** |  | ** | 4 |
| Yöyen & Bozacı, 2023 | *** |  | ** | 5 |
| Yrondi et al., 2021 | ** | ** | ** | 6 |
| Yubero et al., 2021 | *** |  | ** | 5 |
| Yujing et al., 2023 | *** | * | ** | 6 |
| Zaorska et al., 2020 | * | * | ** | 4 |
| Zhang et al., 2023 | *** |  | ** | 5 |
| Zhou & Li (2024 | **** |  | ** | 6 |
| Zhou et al., 2024 | ** | * | ** | 5 |
| Zhou et al., 2024 | **** |  | ** | 6 |

# SF1. Forest plots investigating associations between CM and resilience in adulthood

**I- Global/trait resilience**

**II- Resilience domains**

1. **Coping**

**2) Self-esteem**

**3) Emotion Regulation**

**4) Self-efficacy**

**5) Well-being**

# SF2. Meta-regressions for associations between CM and resilience in adulthood

Meta-regressions for associations between CM and resilience in adulthood were conducted by overall CM and subtypes. The following variables were explored as potential moderating (continuous) variables: 1) mean age; 2) proportion of males; 3) sample size; 4) study quality score (NOS).

***Global/trait resilience:*** The magnitude of the association between sexual abuse and global/trait resilience decreased with sample size (*n* = 12, *k* = 12, *B* = -0.000, 95% CI [-0.021; 0.002], *p* = 0.018) and increased with study quality (*n* = 12, *k* = 12, *B* = 0.161, 95% CI [0.073; 0.249], *p* < 0.001).

***Resilience domains:*** Coping: the magnitude of the association between overall CM and coping increased with sample size (*n* = 7, *k* = 7, *B* = 0.001, 95% CI [0.000; 0.001], *p* = 0.000) and decreased with age (*n* = 7, *k* = 7, *B* = -0.001, 95% CI [-0.000; -0.000], *p* = 0.003 and study quality (*n* = 7, *k* = 7, *B* = -0.091, 95% CI [-0.164; -0.018], *p* = 0.014). The association between physical abuse and coping decreased with age (*n* = 7, *k* = 7, *B* = -0.000, 95% CI [-0.000; -0.000], *p* = 0.002). Emotion regulation: the association between sexual abuse and emotion regulation decreased with study quality (*n* = 33, *k* = 33, *B* = -0.034, 95% CI [-0.063; 0.005], *p* = 0.021). The association between emotional neglect and emotion regulation increased with age (*n* = 20, *k* = 20, *B* = 0.014, 95% CI [0.005; -0.022], *p* = 0.002) and sample size (*n* = 20, *k* = 20, *B* = -0.000, 95% CI [-0.000; -0.000], *p* = 0.003). The association between physical neglect and emotion regulation increased with age (*n* = 6, *k* = 6, *B* = 0.010, 95% CI [0.000; -0.095], *p* = 0.040). No moderation effects of mean age, percentage of males, sample size, or study quality were found on the associations between overall or any subtype of CM and self-esteem, self-efficacy or well-being.

| **Outcome/Exposure** | **Moderators** | **n *(k)*** | ***B*** | **SE** | **95% CI** | ***Z-*value** | ***p-*value** |
| --- | --- | --- | --- | --- | --- | --- | --- |
| **Global/trait resilience** |  |  |  |  |  |  |  |
| **Overall CM** | Mean age | 26 (*26*) | -0.005 | 0.003 | -0.002; 0.001 | -1.64 | 0.100 |
|  | Sex (% male) | 26 (*26*) | -0.006 | 0.001 | -0.002; 0.001 | -0.82 | 0.410 |
|  | Sample size | 26 (*26*) | -0.000 | 0.000 | -0.001; 0.000 | -0.32 | 0.746 |
|  | Study quality (NOS rating) | 26 (*26*) | 0.042 | 0.026 | -0.009; 0.093 | 1.63 | 0.103 |
| **Emotional abuse** | Mean age | 14 *(14)* | -0.009 | 0.006 | -0.021; 0.002 | -1.64 | 0.100 |
|  | Sex (% male) | 14 *(14)* | 0.002 | 0.002 | -0.001; 0.005 | 1.11 | 0.269 |
|  | Sample size | 14 *(14)* | -0.001 | 0.000 | -0.001; 0.000 | -0.41 | 0.678 |
|  | Study quality (NOS rating) | 14 *(14)* | 0.086 | 0.047 | -0.007; 0.178 | 1.81 | 0.697 |
| **Physical abuse** | Mean age | 13 *(13)* | -0.007 | 0.006 | -0.019; 0.005 | -1.20 | 0.231 |
|  | Sex (% male) | 13 *(13)* | -0.001 | 0.002 | -0.002; 0.005 | 0.73 | 0.464 |
|  | Sample size | 13 *(13)* | 0.002 | 0.003 | -0.000; -0.001 | 0.60 | 0.549 |
|  | Study quality (NOS rating) | 13 *(13)* | 0.037 | 0.063 | -0.086; 0.159 | 0.59 | 0.556 |
| **Sexual abuse** | Mean age | 12 *(12)* | -0.006 | 0.004 | -0.014; 0.002 | -1.49 | 0.137 |
|  | Sex (% male) | 12 *(12)* | 0.001 | 0.001 | -0.001; 0.004 | 1.14 | 0.255 |
|  | **Sample size** | **12 *(12)*** | **-0.000** | **0.001** | **-0.001; -0.000** | **-2.37** | **0.018** |
|  | **Study quality (NOS rating)** | **12 *(12)*** | **0.161** | **0.045** | **0.073; 0.249** | **3.59** | **<0.001** |
| **Emotional neglect** | Mean age | 12 *(12)* | -0.005 | 0.016 | -0.006; 0.005 | -1.07 | 0.283 |
|  | Sex (% male) | 12 *(12)* | -0.001 | 0.002 | -0.004; 0.003 | -0.43 | 0.669 |
|  | Sample size | 12 *(12)* | 0.000 | 0.000 | -0.000; 0.007 | 1.25 | 0.209 |
|  | Study quality (NOS rating) | 12 *(12)* | -0.017 | 0.061 | -0.137; 0.103 | -0.28 | 0.779 |
| **Physical neglect** | Mean age | 11 *(11)* | 0.004 | 0.008 | -0.016; 0.016 | 0.04 | 0.966 |
|  | Sex (% male) | 11 *(11)* | 0.002 | 0.002 | -0.004; 0.004 | 0.09 | 0.093 |
|  | Sample size | 11 *(11)* | 0.000 | 0.000 | -0.000; 0.000 | 0.94 | 0.346 |
|  | Study quality (NOS rating) | 11 *(11)* | 0.003 | 0.794 | -0.153; 0.159 | 0.04 | 0.970 |
| **Coping** |  |  |  |  |  |  |  |
| **Overall CM** | **Mean age** | **7 *(7)*** | **-0.001** | **0.000** | **-0.000; -0.000** | **-3.01** | **0.003** |
|  | Sex (% male) | 7 *(7)* | 0.007 | 0.001 | -0.000; 0.002 | 1.46 | 0.146 |
|  | **Sample size** | **7 *(7)*** | **0.001** | **0.000** | **0.000; 0.001** | **5.42** | **0.000** |
|  | **Study quality (NOS rating)** | **7 *(7)*** | **-0.091** | **0.037** | **-0.164; -0.018** | **-2.45** | **0.014** |
| **Physical abuse** | **Mean age** | **5 *(5)*** | **-0.000** | **0.000** | **-0.000; -0.000** | **-3.15** | **0.002** |
|  | Sex (% male) | 5 *(5)* | 0.003 | 0.002 | -0.001; 0.008 | 1.36 | 0.173 |
|  | Sample size | 5 *(5)* | 0.000 | 0.000 | -0.001; 0.001 | 0.25 | 0.806 |
|  | Study quality (NOS rating) | 5 *(5)* | 0.082 | 0.087 | -0.089; 0.025 | 0.94 | 0.348 |
| **Sexual abuse** | Mean age | 7 *(7)* | -0.000 | 0.000 | -0.000; 0.000 | -0.21 | 0.834 |
|  | Sex (% male) | 7 *(7)* | 0.001 | 0.004 | -0.007; 0.009 | 0.32 | 0.751 |
|  | Sample size | 7 *(7)* | -0.000 | 0.000 | -0.002; 0.001 | -0.36 | 0.719 |
|  | Study quality (NOS rating) | 7 *(7)* | -0.053 | 0.199 | -0.443; 0.336 | -0.27 | 0.789 |
| **Self-esteem** |  |  |  |  |  |  |  |
| **Overall CM** | Mean age | 25 *(25)* | 0.001 | 0.003 | -0.006; 0.007 | 0.25 | 0.805 |
|  | Sex (% male) | 25 *(25)* | 0.001 | 0.001 | -0.002; 0.003 | 0.51 | 0.612 |
|  | Sample size | 25 *(25)* | -0.000 | 0.000 | -0.000; 0.000 | -0.76 | 0.449 |
|  | Study quality (NOS rating) | 25 *(25)* | -0.009 | 0.036 | -0.078; 0.062 | -0.24 | 0.809 |
| **Emotional abuse** | Mean age | 19 *(19)* | 0.005 | 0.003 | -0.001; 0.011 | 1.67 | 0.096 |
|  | Sex (% male) | 19 *(19)* | -0.001 | 0.002 | -0.004; 0.020 | -0.56 | 0.577 |
|  | Sample size | 19 *(19)* | -0.000 | 0.000 | -0.001; 0.000 | -0.52 | 0.601 |
|  | Study quality (NOS rating) | 19 *(19)* | 0.046 | 0.043 | -0.038; -0.131 | 1.08 | 0.282 |
| **Physical abuse** | Mean age | 23 *(23)* | -0.005 | 0.006 | -0.016; 0.007 | -0.81 | 0.419 |
|  | Sex (% male) | 23 *(23)* | 0.001 | 0.002 | -0.003; 0.006 | 0.55 | 0.582 |
|  | Sample size | 23 *(23)* | -0.001 | 0.000 | -0.001; 0.000 | -0.36 | 0.717 |
|  | Study quality (NOS rating) | 23 *(23)* | 0.034 | 0.087 | -0.138; 0.205 | 0.38 | 0.700 |
| **Sexual abuse** | Mean age | 23 *(23)* | -0.002 | 0.003 | -0.008; 0.004 | -0.72 | 0.471 |
|  | Sex (% male) | 23 *(23)* | 0.000 | 0.001 | -0.002; 0.003 | 0.37 | 0.711 |
|  | Sample size | 23 *(23)* | -0.000 | 0.000 | -0.000; 0.000 | -0.49 | 0.623 |
|  | Study quality (NOS rating) | 23 *(23)* | 0.006 | 0.031 | -0.054; 0.067 | 0.21 | 0.837 |
| **Emotional neglect** | Mean age | 16 *(16)* | -0.000 | 0.003 | -0.006; 0.006 | -0.04 | 0.967 |
|  | Sex (% male) | 16 *(16)* | 0.003 | 0.002 | -0.000; 0.006 | 1.82 | 0.069 |
|  | Sample size | 16 *(16)* | -0.000 | 0.000 | -0.001; 0.000 | -1.96 | 0.050 |
|  | Study quality (NOS rating) | 16 *(16)* | -0.026 | 0.451 | -0.114; 0.063 | -0.57 | 0.569 |
| **Physical neglect** | Mean age | 14 *(14)* | -0.004 | 0.007 | -0.017; 0.009 | -0.52 | 0.599 |
|  | Sex (% male) | 14 *(14)* | 0.003 | 0.004 | -0.007; 0.007 | 0.07 | 0.944 |
|  | Sample size | 14 *(14)* | -0.000 | 0.001 | -0.001; 0.001 | -0.49 | 0.626 |
|  | Study quality (NOS rating) | 14 *(14)* | 0.026 | 0.124 | -0.217; 0.269 | 0.21 | 0.834 |
| **Bullying** | Mean age | 7 *(7)* | 0.008 | 0.284 | -0.047; 0.064 | 0.29 | 0.774 |
|  | Sex (% male) | 7 *(7)* | -0.006 | 0.007 | -0.021; 0.009 | -0.79 | 0.431 |
|  | Sample size | 7 *(7)* | 0.000 | 0.001 | -0.001; 0.001 | 0.23 | 0.822 |
|  | Study quality (NOS rating) | 7 *(7)* | -0.069 | 0.143 | -0.349; 0.210 | -0.49 | 0.625 |
| **Emotion Regulation** |  |  |  |  |  |  |  |
| **Overall CM** | Mean age | 30 *(30)* | -0.002 | 0.004 | -0.009; 0.006 | -0.43 | 0.669 |
|  | Sex (% male) | 30 *(30)* | -0.001 | 0.001 | -0.003; 0.001 | -0.87 | 0.384 |
|  | Sample size | 30 *(30)* | 0.000 | 0.000 | -0.000; 0.000 | 0.66 | 0.510 |
|  | Study quality (NOS rating) | 30 *(30)* | -0.012 | 0.020 | -0.052; 0.281 | -0.58 | 0.560 |
| **Emotional abuse** | Mean age | 32 *(32)* | -0.001 | 0.003 | -0.007; 0.005 | -0.36 | 0.719 |
|  | Sex (% male) | 32 *(32)* | 0.003 | 0.002 | -0.008; 0.006 | 1.49 | 0.135 |
|  | Sample size | 32 *(32)* | -0.000 | 0.000 | -0.000; 0.000 | -0.08 | 0.938 |
|  | Study quality (NOS rating) | 32 *(32)* | -0.022 | 0.018 | -0.057; 0.014 | -1.20 | 0.229 |
| **Physical abuse** | Mean age | 27 *(27)* | -0.002 | 0.003 | -0.008; 0.003 | -0.85 | 0.394 |
|  | Sex (% male) | 27 *(27)* | -0.000 | 0.001 | -0.003; 0.002 | -0.20 | 0.839 |
|  | Sample size | 27 *(27)* | 0.000 | 0.000 | -0.000; 0.001 | 0.67 | 0.505 |
|  | Study quality (NOS rating) | 27 *(27)* | -0.015 | 0.016 | -0.046; 0.016 | -0.95 | 0.343 |
| **Sexual abuse** | Mean age | 33 *(33)* | -0.002 | 0.003 | -0.007; 0.004 | -0.58 | 0.565 |
|  | Sex (% male) | 33 *(33)* | -0.000 | 0.001 | -0.002; 0.002 | -0.00 | 0.997 |
|  | Sample size | 33 *(33)* | 0.000 | 0.000 | -0.000; 0.001 | 0.35 | 0.729 |
|  | **Study quality (NOS rating)** | **33 *(33)*** | **-0.034** | **0.015** | **-0.063; 0.005** | **-2.31** | **0.021** |
| **Emotional neglect** | **Mean age** | **20 *(20)*** | **0.014** | **0.005** | **0.005; 0.022** | **3.06** | **0.002** |
|  | Sex (% male) | 20 *(20)* | 0.001 | 0.002 | -0.003; 0.006 | 0.58 | 0.560 |
|  | **Sample size** | **20 *(20)*** | **0.000** | **0.000** | **0.000; 0.000** | **2.97** | **0.003** |
|  | Study quality (NOS rating) | 20 *(20)* | -0.010 | 0.025 | -0.059; 0.040 | -0.38 | 0.703 |
| **Physical neglect** | **Mean age** | **6 *(6)*** | **0.010** | **0.005** | **0.000; 0.095** | **2.05** | **0.040** |
|  | Sex (% male) | 6 *(6)* | -0.005 | 0.008 | -0.020; 0.011 | -0.61 | 0.545 |
|  | Sample size | 6 *(6)* | 0.000 | 0.000 | -0.000; 0.000 | 1.87 | 0.062 |
|  | Study quality (NOS rating) | 6 *(6)* | -0.019 | 0.025 | -0.067; 0.031 | -0.74 | 0.460 |
| **Self-efficacy** |  |  |  |  |  |  |  |
| **Overall CM** | Mean age | 6 *(6)* | 0.010 | 0.217 | -0.033; 0.053 | 0.46 | 0.646 |
|  | Sex (% male) | 6 *(6)* | -0.003 | 0.009 | -0.021; 0.014 | -0.36 | 0.717 |
|  | Sample size | 6 *(6)* | 0.000 | 0.000 | -0.001; 0.001 | 0.14 | 0.887 |
|  | Study quality (NOS rating) | 6 *(6)* | 0.155 | 0.083 | -0.008; 0.319 | 1.86 | 0.063 |
| **Emotional abuse** | Mean age | 6 *(6)* | -0.001 | 0.014 | -0.034; 0.021 | -0.47 | 0.635 |
|  | Sex (% male) | 6 *(6)* | -0.003 | 0.002 | -0.008; 0.002 | -1.36 | 0.174 |
|  | Sample size | 6 *(6)* | 0.000 | 0.000 | -0.000; 0.001 | 0.83 | 0.409 |
|  | Study quality (NOS rating) | 6 *(6)* | 0.043 | 0.100 | -0.153; 0.239 | 0.43 | 0.668 |
| **Physical abuse** | Mean age | 6 *(6)* | -0.001 | 0.015 | -0.040; 0.018 | -0.76 | 0.447 |
|  | Sex (% male) | 6 *(6)* | -0.005 | 0.002 | -0.009; 0.000 | -1.86 | 0.063 |
|  | Sample size | 6 *(6)* | 0.000 | 0.000 | -0.000; 0.001 | 0.39 | 0.696 |
|  | Study quality (NOS rating) | 6 *(6)* | 0.003 | 0.111 | -0.215; 0.220 | 0.02 | 0.981 |
| **Sexual abuse** | Mean age | 5 *(5)* | -0.002 | 0.008 | -0.017; 0.013 | -0.23 | 0.817 |
|  | Sex (% male) | 5 *(5)* | -0.001 | 0.002 | -0.005; 0.002 | -0.76 | 0.445 |
|  | Sample size | 5 *(5)* | -0.000 | 0.000 | -0.000; 0.000 | -0.18 | 0.855 |
|  | Study quality (NOS rating) | 5 *(5)* | 0.034 | 0.054 | -0.071; 0.139 | 0.64 | 0.522 |
| **Emotional neglect** | Mean age | 5 *(5)* | -0.001 | 0.019 | -0.039; -0.037 | -0.06 | 0.949 |
|  | Sex (% male) | 5 *(5)* | -0.002 | 0.004 | -0.011; 0.005 | -0.63 | 0.529 |
|  | Sample size | 5 *(5)* | 0.001 | 0.000 | -0.000; 0.001 | 0.48 | 0.631 |
|  | Study quality (NOS rating) | 5 *(5)* | 0.132 | 0.110 | -0.084; 0.348 | 1.20 | 0.232 |
| **Physical neglect** | Mean age | 5 *(5)* | -0.002 | 0.016 | -0.032; 0.028 | -0.14 | 0.893 |
|  | Sex (% male) | 5 *(5)* | -0.001 | 0.003 | -0.007; 0.006 | -0.16 | 0.869 |
|  | Sample size | 5 *(5)* | 0.000 | 0.000 | -0.000; 0.001 | 0.31 | 0.755 |
|  | Study quality (NOS rating) | 5 *(5)* | 0.146 | 0.069 | 0.010; 0.282 | 2.11 | 0.035 |
| **Well-being** |  |  |  |  |  |  |  |
| **Overall CM** | Mean age | 16 *(16)* | -0.007 | 0.006 | -0.019; 0.005 | -1.21 | 0.225 |
|  | Sex (% male) | 16 *(16)* | 0.002 | 0.002 | -0.002; 0.067 | 0.98 | 0.328 |
|  | Sample size | 16 *(16)* | 0.000 | 0.000 | -0.000; 0.000 | 1.82 | 0.069 |
|  | Study quality (NOS rating) | 16 *(16)* | -0.137 | 0.028 | -0.068; 0.040 | -0.50 | 0.619 |
| **Emotional abuse** | Mean age | 11 *(11)* | -0.002 | 0.005 | -0.012; 0.008 | -0.34 | 0.735 |
|  | Sex (% male) | 11 *(11)* | 0.002 | 0.004 | -0.006; 0.011 | 0.52 | 0.604 |
|  | Sample size | 11 *(11)* | 0.000 | 0.000 | -0.000; 0.000 | 0.67 | 0.505 |
|  | Study quality (NOS rating) | 11 *(11)* | 0.029 | 0.037 | -0.043; 0.101 | 0.79 | 0.427 |
| **Physical abuse** | Mean age | 5 *(5)* | -0.002 | 0.001 | -0.005; 0.001 | -1.29 | 0.197 |
|  | Sex (% male) | 5 *(5)* | -0.002 | 0.003 | -0.007; 0.004 | -0.63 | 0.531 |
|  | Sample size | 5 *(5)* | -0.000 | 0.000 | -0.000; 0.000 | -0.90 | 0.366 |
|  | Study quality (NOS rating) | 5 *(5)* | 0.009 | 0.012 | -0.014; 0.034 | 0.81 | 0.418 |
| **Emotional neglect** | Mean age | 5 *(5)* | -0.000 | 0.002 | -0.005; 0.005 | -0.05 | 0.964 |
|  | Sex (% male) | 5 *(5)* | -0.001 | 0.003 | -0.005; 0.004 | -0.22 | 0.825 |
|  | Sample size | 5 *(5)* | -0.000 | 0.000 | -0.000; 0.000 | -1.35 | 0.178 |
|  | Study quality (NOS rating) | 5 *(5)* | -0.000 | 0.026 | -0.052; 0.051 | -0.01 | 0.991 |

***Note.*** As a general rule, estimates of heterogeneity based on less than 10 studies are not likely to be reliable (Borenstein, 2022, 2024; Borenstein et al., 2017).

# SF3. Subgroup analyses for associations between CM and resilience in adulthood

Subgroup analyses for associations between CM and resilience in adulthood were conducted by overall CM and subtypes. The following variables were explored as potential moderating (categorical) variables: 1) western *vs.* non-western countries; 2) clinical vs. non-clinical samples.

***Global/trait resilience:*** No differences were found in the association between overall or any subtype of CM and global/trait resilience in western *vs.* non-western countries, or in samples with mental disorders *vs.* without mental disorders.

***Resilience domains:*** The association between emotional abuse and emotion regulation was higher in western countries (*n* = 21, *r* = -0.321, [-0.364; -0.277]) *vs.* non-western countries (*n* = 16, *r* = -0.215, [-0.282; -0.1545), *p* = 0.010 (see figure a). The association between emotional abuse and self-esteem was lower in western countries (*n* = 9, *r* = -0.213, [-0.321; -0.098]) *vs.* non-western countries (*n* = 15, *r* = -0.352, [-0.407; -0.296]), *p* = 0.025 (see figure b).

No differences were found in the association between overall or any subtype of CM and any resilience domains in clinical vs. non-clinical samples.

**a.**

**b.**

***Note***. 1 = western country; 2 = non-western country.

# SF4. One-study-removed sensitivity analyses

To further assess possible causes of heterogeneity and robustness of findings, one-study-removed sensitivity analyses (Borenstein et al., 2022) were conducted. Removal of single effect sizes did not change the patterns of results with a few exceptions:

For the association between sexual abuse and coping, the removal of Lacelle et al. (*r =* -0.081 [95% CI = -0.134, -0.027], *p* = 0.003) led to a significant negative association, which was not observed with the inclusion of this study.

For the association between physical abuse and self-esteem, the removal of Babad et al. (*r =* -0.118 [95% CI = -0.224, -0.008], *p* = 0.035), or Toker et al. (*r =* -0.157 [95% CI = -0.222, -0.090], *p* = 0.028) led to a significant negative association, which was not observed with the inclusion of these studies. For the association between physical neglect and self-esteem, the removal of Zhou & Li (*r =* -0.183 [95% CI = -0.255, -0.110], *p* < 0.001) led to a significant negative association, which was not observed with the inclusion of this study.

For the association between overall CM and self-efficacy, the removal of Güler et al. (*r =* -0.300 [95% CI = -0.588, -0.035], *p* = 0.030), Li et al. (*r =* -0.256 [95% CI = -0.509, 0.038], *p* = 0.087), See Mey et al. (*r =* -0.239 [95% CI = -0.494, 0.055], *p* = 0.110), and Wu et al. (*r =* -0.313 [95% CI = -0.603, 0.051], *p* = 0.091), resulted in the previous significant negative association becoming not significant. For the association between physical abuse and self-efficacy, the removal of Güler et al. (*r =* -0.300 [95% CI = -0.588, -0.035], *p* = 0.030), Li et al. (*r =* -0.256 [95% CI = -0.509, 0.038], *p* = 0.087), See Mey et al. (*r =* -0.239 [95% CI = -0.494, 0.055], *p* = 0.110), and Wu et al. (*r =* -0.313 [95% CI = -0.603, 0.051], *p* = 0.091), led to a non-significant association, which was significant (negative) with the inclusion of these studies. For the association between sexual abuse and self-efficacy, the removal of See Mey et al. (*r =* -0.063 [95% CI = -0.157, 0.031], *p* = 0.187), and Wu et al. (*r =* -0.068 [95% CI = -0.177, 0.041], *p* = 0.222) led to non-significant association, which was significant (negative) with the inclusion of these studies. For the association between physical neglect and self-efficacy, the removal of Güler et al. (*r =* -0.151 [95% CI = -0.301, 0.006], *p* < 0.059), See Mey et al. (*r =* -0.185 [95% CI = -0.361, 0.003], *p* = 0.054), and Wu et al. (*r =* -0.198 [95% CI = -0.406, 0.028], *p* = 0.085) led to non-significant association, which was significant (negative) with the inclusion of these studies.

# SF5. Funnel plots for associations between CM and resilience in adulthood

**I. Global trait/resilience**

***Note.*** The funnel plots investigating publication bias for the association between CM and global/trait resilience are displayed in this order: overall CM, emotional abuse, physical abuse, sexual abuse, emotional neglect, physical neglect. The plot observed is represented in blue and the plot imputed is represented in red.

**II. Resilience domains**

**Coping**

***Note.*** Funnel plot investigating publication bias for the association between overall CM and coping. The plot observed is represented in blue and the plot imputed is represented in red.

**Self-esteem**

******

******

******

******

******

***Note.*** Funnel plots investigating publication bias for the association between CM and self-esteem displayed in order: overall CM, emotional abuse, physical abuse, sexual abuse, emotional neglect, physical neglect. The plot observed is represented in blue and the plot imputed is represented in red.

**Emotion Regulation**

***Note.*** Funnel plots investigating publication bias for the association between CM and emotion regulation displayed in order: overall CM, emotional abuse, physical abuse, sexual abuse, emotional neglect, physical neglect. The plot observed is represented in blue and the plot imputed is represented in red.

**Well-being**

***Note.*** Funnel plots investigating publication bias for the association between CM and well-being displayed in order: overall CM, emotional abuse. The plot observed is represented in blue and the plot imputed is represented in red.

# References

Andrews, G., Singh, M., & Bond, M. (1993). The Defense Style Questionnaire. *The Journal of Nervous and Mental Disease*, *181*(4), 246.

Antonovsky, A. (1993). The structure and properties of the sense of coherence scale. *Social Science & Medicine*, *36*(6), 725–733. https://doi.org/10.1016/0277-9536(93)90033-Z

Armitage, J. M., Wang, R. A. H., Davis, O. S. P., Bowes, L., & Haworth, C. M. A. (2021). Peer victimisation during adolescence and its impact on wellbeing in adulthood: A prospective cohort study. *BMC Public Health*, *21*(1), 148. https://doi.org/10.1186/s12889-021-10198-w

Arslan, G. (2015). Relationship between Childhood Psychological Maltreatment, Resilience, Depression, and Negative Self-concept. *Neuropsychiatric Investigation*, *53*, 3–10. https://doi.org/10.5455/NYS.20160328090400

Aydin, C., Kaya, M., & Peker, H. (2015). Hayatın Anlam ve Amacı Ölçeği: Geçerlik ve Güvenirlik Çalışması. *Ondokuz Mayıs Üniversitesi İlahiyat Fakültesi Dergisi*, *0*(38), 39. https://doi.org/10.17120/omuifd.80248

Bandura, A. (1982). Self-efficacy mechanism in human agency. *American Psychologist*, *37*(2), 122–147. https://doi.org/10.1037/0003-066X.37.2.122

Beck, A. T., Brown, G. K., Steer, R. A., Kuyken, W., & Grisham, J. (2001). Psychometric properties of the Beck Self-Esteem Scales. *Behaviour Research and Therapy*, *39*(1), 115–124. https://doi.org/10.1016/S0005-7967(00)00028-0

Beck, A. T., Brown, G. K., Steer, R. A., Kuyken, W., & Grisham, J. (2013). *Beck Self-Esteem Scales* [Dataset]. https://doi.org/10.1037/t16045-000

Blanke, E. S., Schmiedek, F., Siebert, S., Richter, D., & Brose, A. (2023). Perspectives on resilience: Trait resilience, correlates of resilience in daily life, and longer-term change in affective distress. *Stress and Health: Journal of the International Society for the Investigation of Stress*, *39*(1), 59–73. https://doi.org/10.1002/smi.3164

Blomkvist, V., Hannerz, J., Katz, L., & Theorell, T. (2002). Coping style and social support in men and women suffering from cluster headache or migraine. *Headache*, *42*(3), 178–184. https://doi.org/10.1046/j.1526-4610.2002.02049.x

Bonanno, G. A., Romero, S. A., & Klein, S. I. (2015). The temporal elements of psychological resilience: An integrative framework for the study of individuals, families, and communities. *Psychological Inquiry*, *26*(2), 139–169. https://doi.org/10.1080/1047840X.2015.992677

Bonanno, G. A., Westphal, M., & Mancini, A. D. (2011). Resilience to loss and potential trauma. *Annual Review of Clinical Psychology*, *7*, 511–535. https://doi.org/10.1146/annurev-clinpsy-032210-104526

Borenstein, M. (2022). In a meta-analysis, the I-squared statistic does not tell us how much the effect size varies. *Journal of Clinical Epidemiology*. https://doi.org/10.1016/j.jclinepi.2022.10.003

Borenstein, M. (2024). Avoiding common mistakes in meta-analysis: Understanding the distinct roles of Q, I-squared, tau-squared, and the prediction interval in reporting heterogeneity. *Research Synthesis Methods*, *15*(2), 354–368. https://doi.org/10.1002/jrsm.1678

Borenstein, M., Higgins, J. P. T., Hedges, L. V., & Rothstein, H. R. (2017). Basics of meta-analysis: I2 is not an absolute measure of heterogeneity. *Research Synthesis Methods*, *8*(1), 5–18. https://doi.org/10.1002/jrsm.1230

Bouchard, G., & Sonier, N. A. (2021). Relationship between sibling bullying, family functioning, and problem solving: A structural equation modeling. *Current Psychology: A Journal for Diverse Perspectives on Diverse Psychological Issues*, No Pagination Specified-No Pagination Specified. https://doi.org/10.1007/s12144-021-02475-z

Brown, J. D., Dutton, K. A., & Cook, K. E. (2001). From the top down: Self-esteem and self-evaluation. *Cognition and Emotion*, *15*(5), 615–631. https://doi.org/10.1080/02699930126063

Caikang, W. (2001). Evidences for Reliability and Validity of the Chinese Version of General SelfEfficacy Scale. *Chinese Journal of Applied Psychology*. https://www.semanticscholar.org/paper/Evidences-for-Reliability-and-Validity-of-the-of-Caikang/1acea108f7176c2b00654a2401cfe3b49517aaaf

Caprara, G.V. & Gerbino, M. (2001). Affective perceived self-efficacy: The capacity to regulate negative affect and to express positive affect. In *In: Caprara GV, editor. Self-efficacy assessment* (pp. 35–50). Edizioni Erickson.

Carver, C. S. (1997). You want to measure coping but your protocol’s too long: Consider the brief COPE. *International Journal of Behavioral Medicine*, *4*(1), 92–100. https://doi.org/10.1207/s15327558ijbm0401_6

Carver, C. S., Scheier, M. F., & Weintraub, J. K. (1989). Assessing coping strategies: A theoretically based approach. *Journal of Personality and Social Psychology*, *56*(2), 267–283. https://doi.org/10.1037//0022-3514.56.2.267

Catanzaro, S. J., & Mearns, J. (1990). Measuring generalized expectancies for negative mood regulation: Initial scale development and implications. *Journal of Personality Assessment*, *54*(3–4), 546–563. https://doi.org/10.1080/00223891.1990.9674019

Chi, X.-L., Huang, Q.-M., Liu, X.-F., Huang, L.-Y., Hu, M.-J., Chen, Z.-J., Jiao, C., Stubbs, B., Hossain, M. M., & Zou, L.-Y. (2021). Self-compassion and resilience mediate the relationship between childhood exposure to domestic violence and posttraumatic growth/stress disorder during COVID-19 pandemic. *World Journal of Psychiatry*, *11*(11), 1106–1115. https://doi.org/10.5498/wjp.v11.i11.1106

Connor, K. M., & Davidson, J. R. T. (2003). Development of a new resilience scale: The Connor-Davidson Resilience Scale (CD-RISC). *Depression and Anxiety*, *18*(2), 76–82. https://doi.org/10.1002/da.10113

Coopersmith, S. (2012). *Coopersmith Self-Esteem Inventories* [Dataset]. https://doi.org/10.1037/t06456-000

Diener, E. (2000). Subjective well-being: The science of happiness and a proposal for a national index. *American Psychologist*, *55*(1), 34–43. https://doi.org/10.1037/0003-066X.55.1.34

Diener, E., Emmons, R. A., Larsen, R. J., & Griffin, S. (1985). The Satisfaction With Life Scale. *Journal of Personality Assessment*, *49*(1), 71–75. https://doi.org/10.1207/s15327752jpa4901_13

Endler, N. S., & Parker, J. D. A. (1994). Assessment of multidimensional coping: Task, emotion, and avoidance strategies. *Psychological Assessment*, *6*(1), 50–60. https://doi.org/10.1037/1040-3590.6.1.50

Epstein, S., Roberts, E., Sedgwick, R., Finning, K., Ford, T., Dutta, R., & Downs, J. (2018). Poor school attendance and exclusion: A systematic review protocol on educational risk factors for self-harm and suicidal behaviours. *BMJ Open*, *8*(12), e023953. https://doi.org/10.1136/bmjopen-2018-023953

Erol, Y., & Inozu, M. (2024). An Investigation of the Mediating Roles of Emotion Regulation Difficulties, Distress Tolerance, Self-Compassion, and Self-Disgust in the Association Between Childhood Trauma and Nonsuicidal Self-Injury. *Archives of Suicide Research: Official Journal of the International Academy for Suicide Research*, *28*(3), 815–829. https://doi.org/10.1080/13811118.2023.2237083

Fares-Otero, N. E., & Seedat, S. (2024). Childhood maltreatment: A call for a standardised definition and applied framework. *European Neuropsychopharmacology: The Journal of the European College of Neuropsychopharmacology*, *87*, 24–26. https://doi.org/10.1016/j.euroneuro.2024.07.002

Feinauer, L. L., Mitchell, J., Harper, J. M., & Dane, S. (1996). The impact of hardiness and severity of childhood sexual abuse on adult adjustment. *American Journal of Family Therapy*, *24*(3), 206–214. https://doi.org/10.1080/01926189608251034

Fitzgerald, M., & Barton, C. (2022). Self-qualities and self-leadership as pathways linking childhood maltreatment to depression and relationship quality. *Contemporary Family Therapy: An International Journal*, *44*(2), 156–166. https://doi.org/10.1007/s10591-021-09577-7

Folkman, S., Lazarus, R. S., Dunkel-Schetter, C., DeLongis, A., & Gruen, R. J. (1986). Dynamics of a stressful encounter: Cognitive appraisal, coping, and encounter outcomes. *Journal of Personality and Social Psychology*, *50*(5), 992–1003. https://doi.org/10.1037//0022-3514.50.5.992

Friborg, O., Hjemdal, O., Rosenvinge, J. H., & Martinussen, M. (2003). A new rating scale for adult resilience: What are the central protective resources behind healthy adjustment? *International Journal of Methods in Psychiatric Research*, *12*(2), 65–76. https://doi.org/10.1002/mpr.143

Garcia, C. O., & Berzenski, S. R. (2023). The Importance of Perception and Personality on the Association Between Childhood Neglect and Adult Social Competence. *Journal of Aggression, Maltreatment & Trauma*, *32*(10), 1337–1352. https://doi.org/10.1080/10926771.2023.2189045

Garnefski, N., Kraaij, V., & Spinhoven, P. (2001). Negative life events, cognitive emotion regulation and emotional problems. *Personality and Individual Differences*, *30*(8), 1311–1327. https://doi.org/10.1016/S0191-8869(00)00113-6

Gnambs, T., Scharl, A., & Schroeders, U. (2018). The structure of the Rosenberg Self-Esteem Scale: A cross-cultural meta-analysis. *Zeitschrift Für Psychologie*, *226*(1), 14–29. https://doi.org/10.1027/2151-2604/a000317

Goemans, A., Viding, E., & McCrory, E. (2023). Child Maltreatment, Peer Victimization, and Mental Health: Neurocognitive Perspectives on the Cycle of Victimization. *Trauma, Violence & Abuse*, *24*(2), 530–548. https://doi.org/10.1177/15248380211036393

Goldberg, D. P., Gater, R., Sartorius, N., Ustun, T. B., Piccinelli, M., Gureje, O., & Rutter, C. (1997). The validity of two versions of the GHQ in the WHO study of mental illness in general health care. *Psychological Medicine*, *27*(1), 191–197. https://doi.org/10.1017/S0033291796004242

Goodboy, A. K., Martin, M. M., & Goldman, Z. W. (2016). Students’ experiences of bullying in high school and their adjustment and motivation during the first semester of college. *Western Journal of Communication*, *80*(1), 60–78. https://doi.org/10.1080/10570314.2015.1078494

Gratz, K. L., & Roemer, L. (2004). Multidimensional Assessment of Emotion Regulation and Dysregulation: Development, Factor Structure, and Initial Validation of the Difficulties in Emotion Regulation Scale. *Journal of Psychopathology and Behavioral Assessment*, *26*(1), 41–54. https://doi.org/10.1023/B:JOBA.0000007455.08539.94

Gross, J. J., & John, O. P. (2003). Individual differences in two emotion regulation processes: Implications for affect, relationships, and well-being. *Journal of Personality and Social Psychology*, *85*(2), 348–362. https://doi.org/10.1037/0022-3514.85.2.348

Herzog, R., Álvarez-Pasquin, M. J., Díaz, C., Del Barrio, J. L., Estrada, J. M., & Gil, Á. (2013). Are healthcare workers’ intentions to vaccinate related to their knowledge, beliefs and attitudes? A systematic review. *BMC Public Health*, *13*(1), 154. https://doi.org/10.1186/1471-2458-13-154

Holaday, M., Callahan, K., Fabre, L., Hall, C., MacDonald, N., Mundy, M. A., Owens, B., & Plappert, H. (1996). A comparison of Culture-Free Self-Esteem Scale means from different child and adolescent groups. *Journal of Personality Assessment*, *66*(3), 540–554. https://doi.org/10.1207/s15327752jpa6603_5

Huppert, F. A. (2009). Psychological well‐being: Evidence regarding its causes and consequences. *Applied Psychology: Health and Well-Being*, *1*(2), 137–164. https://doi.org/10.1111/j.1758-0854.2009.01008.x

Jones, R. L. (Ed.). (1996). *Handbook of tests and measurements for black populations*. Cobb & Henry Publishers.

Kapoor, S., Domingue, H. K., Watson-Singleton, N. N., Are, F., Elmore, C. A., Crooks, C. L., Madden, A., Mack, S. A., Peifer, J. S., & Kaslow, N. J. (2018). Childhood Abuse, Intrapersonal Strength, and Suicide Resilience in African American Females who Attempted Suicide. *Journal of Family Violence*, *33*(1), 53–64. https://doi.org/10.1007/s10896-017-9943-2

Karakaş, M., & Çingöl, N. (2022). The relationship of childhood trauma experiences with cognitive distortions and sense of coherence in nursing students. *Perspectives in Psychiatric Care*, *58*(4), 1546–1553. https://doi.org/10.1111/ppc.12962

Keyes, C. L. M. (2009). *Atlanta: Brief description of the mental health continuum short form (MHC-SF)*. le: http://www.sociology.emory.edu/ckeyes/.

Kim, M., Hong, G., Kim, R. Y., Song, Y., Lee, H., Joo, Y., Kim, J., & Yoon, S. (2021). Severity of post-traumatic stress disorder and childhood abuse in adult crime victims as mediated by low resilience and dysfunctional coping strategies. *Child Abuse & Neglect*, *118*, 105154. https://doi.org/10.1016/j.chiabu.2021.105154

Kok, R. (2020). Emotion Regulation. In V. Zeigler-Hill & T. K. Shackelford (Eds.), *Encyclopedia of Personality and Individual Differences* (pp. 1281–1290). Springer International Publishing. https://doi.org/10.1007/978-3-319-24612-3_811

Kovacs, M., Rottenberg, J., & George, C. (2009). Maladaptive mood repair responses distinguish young adults with early-onset depressive disorders and predict future depression outcomes. *Psychological Medicine*, *39*(11), 1841–1854. https://doi.org/10.1017/S0033291709005789

Lassri, D., Bregman-Hai, N., Soffer-Dudek, N., & Shahar, G. (2023). The Interplay Between Childhood Sexual Abuse, Self-Concept Clarity, and Dissociation: A Resilience-Based Perspective. *Journal of Interpersonal Violence*, *38*(3–4), 2313–2336. https://doi.org/10.1177/08862605221101182

Lu, F.-Y., Wen, S., Deng, G., & Tang, Y.-L. (2017). Self-concept mediate the relationship between childhood maltreatment and abstinence motivation as well as self-efficacy among drug addicts. *Addictive Behaviors*, *68*, 52–58. https://doi.org/10.1016/j.addbeh.2017.01.017

Lynam, D. R., Smith, G. T., Whiteside, S. P., & Cyders, M. A. (2006). *The UPPS-P: Assessing five personality pathways to impulsive behavior* (Technical 10). Purdue University.

Marsh, H. W., & Richards, G. E. (1988). Tennessee Self Concept Scale: Reliability, internal structure, and construct validity. *Journal of Personality and Social Psychology*, *55*(4), 612–624. https://doi.org/10.1037/0022-3514.55.4.612

Martxueta, A., & Etxeberria, J. (2014). Análisis diferencial retrospectivo de las variables de salud mental en lesbianas, gais y bisexuales (LGB) víctimas de bullying homofóbico en la escuela. [Retrospective differential analysis of mental health variables in lesbians, gays and bisexuals (LGB) who suffered homophobic bullying at school.]. *Revista de Psicopatología y Psicología Clínica*, *19*(1), 23–35. https://doi.org/10.5944/rppc.vol.19.num.1.2014.12980

May, B. A., & Limandri, B. J. (2004). Instrument development of the Self-Efficacy Scale for Abused Women. *Research in Nursing & Health*, *27*(3), 208–214. https://doi.org/10.1002/nur.20018

McRae, K., & Gross, J. J. (2020). Emotion regulation. *Emotion (Washington, D.C.)*, *20*(1), 1–9. https://doi.org/10.1037/emo0000703

Mertz, D., Kim, T. H., Johnstone, J., Lam, P.-P., Science, M., Kuster, S. P., Fadel, S. A., Tran, D., Fernandez, E., Bhatnagar, N., & Loeb, M. (2013). Populations at risk for severe or complicated influenza illness: Systematic review and meta-analysis. *BMJ (Clinical Research Ed.)*, *347*, f5061. https://doi.org/10.1136/bmj.f5061

Modesti, P. A., Reboldi, G., Cappuccio, F. P., Agyemang, C., Remuzzi, G., Rapi, S., Perruolo, E., Parati, G., & Settings, E. W. G. on C. R. in L. R. (2016). Panethnic Differences in Blood Pressure in Europe: A Systematic Review and Meta-Analysis. *PLOS ONE*, *11*(1), e0147601. https://doi.org/10.1371/journal.pone.0147601

Naderzadeh, S., Khoran, Z., Khanjani, M., & Wiesmann, U. (2023). Childhood maltreatment, late-life depression, and sense of coherence: A structural equation modeling. *Aging & Mental Health*, *27*(5), 965–972. https://doi.org/10.1080/13607863.2022.2076203

Naughton, C. M., O’Donnell, A. T., & Muldoon, O. T. (2020). Exposure to Domestic Violence and Abuse: Evidence of Distinct Physical and Psychological Dimensions. *Journal of Interpersonal Violence*, *35*(15–16), 3102–3123. https://doi.org/10.1177/0886260517706763

Neill, J. T., & Dias, K. L. (2016). *Resilience Scale—Short Version* [Dataset]. https://doi.org/10.1037/t24805-000

Newman, M. L., Holden, G. W., & Delville, Y. (2011). Coping with the stress of being bullied: Consequences of coping strategies among college students. *Social Psychological and Personality Science*, *2*(2), 205–211. https://doi.org/10.1177/1948550610386388

Osman, A., Gutierrez, P. M., Muehlenkamp, J. J., Dix-Richardson, F., Barrios, F. X., & Kopper, B. A. (2004). Suicide Resilience Inventory-25: Development and preliminary psychometric properties. *Psychological Reports*, *94*(3 Pt 2), 1349–1360. https://doi.org/10.2466/pr0.94.3c.1349-1360

Paloutzian, R. E., & Ellison, C. W. (1991). *Manual for the spiritual well-being scale. Navack: Life Advance.* Life Advance.

Pargament, K. I., Smith, B. W., Koenig, H. G., & Perez, L. (1998). Patterns of positive and negative religious coping with major life stressors. *Journal for the Scientific Study of Religion*, *37*(4), 710–724. https://doi.org/10.2307/1388152

Pearlin, L. I., & Schooler, C. (1978). The Structure of Coping. *Journal of Health and Social Behavior*, *19*(1), 2–21. https://doi.org/10.2307/2136319

Powers, A., Stevens, J., Fani, N., & Bradley, B. (2015). Construct validity of a short, self report instrument assessing emotional dysregulation. *Psychiatry Research*, *225*(1–2), 85–92. https://doi.org/10.1016/j.psychres.2014.10.020

Rafnsson, F. D., Smari, J., Windle, M., Mears, S. A., & Endler, N. S. (2006). Factor structure and psychometric characteristics of the Icelandic version of the Coping Inventory for Stressful Situations (CISS). *Personality and Individual Differences*, *40*(6), 1247–1258. https://doi.org/10.1016/j.paid.2005.11.011

Robson, P. (1989). Development of a new self-report questionnaire to measure self esteem. *Psychological Medicine*, *19*(2), 513–518. https://doi.org/10.1017/s003329170001254x

Robson, P. J. (1988). Self-esteem—A psychiatric view. *The British Journal of Psychiatry: The Journal of Mental Science*, *153*, 6–15. https://doi.org/10.1192/bjp.153.1.6

Rutten, B., Hammels, C., Geschwind, N., Menne-Lothmann, C., Pishva, E., Schruers, K., Schruers, K., Hove, D. V. D., Kenis, G., Os, J., & Wichers, M. (2013). Resilience in mental health: Linking psychological and neurobiological perspectives. *Acta Psychiatrica Scandinavica*. https://www.semanticscholar.org/paper/Resilience-in-mental-health%3A-linking-psychological-Rutten-Hammels/d5b332e9686659538fc9bf068d58ae63d46b30b8

Ryff, C. D. (1989). Happiness is everything, or is it? Explorations on the meaning of psychological well-being. *Journal of Personality and Social Psychology*, *57*(6), 1069–1081. https://doi.org/10.1037/0022-3514.57.6.1069

Ryff, C. D., & Keyes, C. L. M. (1995). The structure of psychological well-being revisited. *Journal of Personality and Social Psychology*, *69*(4), 719–727. https://doi.org/10.1037/0022-3514.69.4.719

Sachs-Ericsson, N., Medley, A. N., Kendall – Tackett, K., & Taylor, J. (2011). Childhood Abuse and Current Health Problems among Older Adults: The Mediating Role of Self-Efficacy. *Psychology of Violence*, *1*(2), 106–120. https://doi.org/10.1037/a0023139

Schwarzer, R. & Jerusalem, M. (1995). Generalized Self-Efficacy scale. In *J. Weinman, S. Wright, & M. Johnston. Measures in health psychology: A user’s portfolio. Causal and control beliefs.* (pp. 35–37). NFER-NELSON.

Shalit, B. & Carlstedt, L. (1984). *The Perception of Enemy Threat. A Method for Assessing the Coping Potentials* (Försvarets Forskningsanstalt Rapport: C 55063-H3).

Sherer, M., Maddux, J. E., Mercandante, B., Prentice-Dunn, S., Jacobs, B., & Rogers, R. W. (1982). The Self-Efficacy Scale: Construction and Validation. *Psychological Reports*, *51*(2), 663–671. https://doi.org/10.2466/pr0.1982.51.2.663

Shin, M., & Brunton, R. (2024). Early life stress and mental health—Attentional bias, executive function and resilience as moderating and mediating factors. *Personality and Individual Differences*, *221*, 1–9. APA PsycInfo. https://doi.org/10.1016/j.paid.2024.112565

Shufeng, W., Dangling, T., & Guoliang, Y. (2009). The characteristics of regulatory emotional self-efficacy in Chinese graduate students. *Psychological Science (China)*, *32*(3), 666–668.

Smith, B. W., Dalen, J., Wiggins, K., Tooley, E., Christopher, P., & Bernard, J. (2008). The brief resilience scale: Assessing the ability to bounce back. *International Journal of Behavioral Medicine*, *15*(3), 194–200. https://doi.org/10.1080/10705500802222972

Steger, M. F., Frazier, P., Oishi, S., & Kaler, M. (2006). The meaning in life questionnaire: Assessing the presence of and search for meaning in life. *Journal of Counseling Psychology*, *53*(1), 80–93. https://doi.org/10.1037/0022-0167.53.1.80

Talmon, A., Cohen, N., Raif, Y., & Ginzburg, K. (2022). Sense of mastery among older adults and its relation to invalidating childhood experiences. *Aging & Mental Health*, *26*(11), 2186–2194. https://doi.org/10.1080/13607863.2021.1993130

Tangney, J. P., Baumeister, R. F., & Boone, A. L. (2004). High Self-Control Predicts Good Adjustment, Less Pathology, Better Grades, and Interpersonal Success. *Journal of Personality*, *72*(2), 271–324. https://doi.org/10.1111/j.0022-3506.2004.00263.x

Tennant, R., Hiller, L., Fishwick, R., Platt, S., Joseph, S., Weich, S., Parkinson, J., Secker, J., & Stewart-Brown, S. (2007). The Warwick-Edinburgh Mental Well-being Scale (WEMWBS): Development and UK validation. *Health and Quality of Life Outcomes*, *5*(1), 63. https://doi.org/10.1186/1477-7525-5-63

Wadji, D. L., Oe, M., Bartoli, E., Martin-Soelch, C., Pfaltz, M. C., & Langevin, R. (2023). How are experiences and acceptability of child maltreatment related to resilience and posttraumatic growth: A cross cultural study. *European Journal of Psychotraumatology*, *14*(2), 2264119. https://doi.org/10.1080/20008066.2023.2264119

Wagnild, G. M., & Young, H. M. (1993). Development and psychometric evaluation of the Resilience Scale. *Journal of Nursing Measurement*, *1*(2), 165–178.

Warner, E. T., Kent, B. V., Zhang, Y., Argentieri, M. A., Rowatt, W. C., Pargament, K., Koenig, H. G., Underwood, L., Cole, S. A., Daviglus, M. L., Kanaya, A. M., Palmer, J. R., Huang, T., Blais, M. A., & Shields, A. E. (2021). The Study on Stress, Spirituality, and Health (SSSH): Psychometric Evaluation and Initial Validation of the SSSH Baseline Spirituality Survey. *Religions*, *12*(3), 150. https://doi.org/10.3390/rel12030150

Wells, G., Wells, G., Shea, B., Shea, B., O’Connell, D., Peterson, J., Welch, Losos, M., Tugwell, P., Ga, S. W., Zello, G., & Petersen, J. (2014). *The Newcastle-Ottawa Scale (NOS) for Assessing the Quality of Nonrandomised Studies in Meta-Analyses*. https://www.semanticscholar.org/paper/The-Newcastle-Ottawa-Scale-(NOS)-for-Assessing-the-Wells-Wells/c293fb316b6176154c3fdbb8340a107d9c8c82bf

Wickersham, A., Leightley, D., Archer, M., & Fear, N. T. (2020). The association between paternal psychopathology and adolescent depression and anxiety: A systematic review. *Journal of Adolescence*, *79*, 232–246. https://doi.org/10.1016/j.adolescence.2020.01.007

Wind, T. W., & Silvern, L. (1994). Parenting and family stress as mediators of the long-term effects of child abuse. *Child Abuse & Neglect*, *18*(5), 439–453. https://doi.org/10.1016/0145-2134(94)90029-9

World Health Organization. (1992). *Assessment of Subjective Well-Being: The Subjective Well-Being lnventory*. World Health Organization: WHO Regional Office for South-East Asia.

World Health Organization,. (2024). *The World Health Organization-Five Well-Being Index (WHO-5)*. World Health Organization.

Xie, Y. (1998). Reliability and validity of the simplified Coping Style Questionnaire. *Chinese Journal of Clinical Psychology*, *6*(2), 114–115.

Ye, Y.-C., Wu, C.-H., Huang, T.-Y., & Yang, C.-T. (2022). The difference between the Connor-Davidson Resilience Scale and the Brief Resilience Scale when assessing resilience: Confirmatory factor analysis and predictive effects. *Global Mental Health (Cambridge, England)*, *9*, 339–346. https://doi.org/10.1017/gmh.2022.38

Yubero, S., de las Heras, M., Navarro, R., & Larrañaga, E. (2021). Relations among chronic bullying victimization, subjective well-being and resilience in university students: A preliminary study. *Current Psychology: A Journal for Diverse Perspectives on Diverse Psychological Issues*, No Pagination Specified-No Pagination Specified. https://doi.org/10.1007/s12144-021-01489-x

Zhang, J. X., & Schwarzer, R. (1995). Measuring optimistic self-beliefs: A Chinese adaptation of the General Self-Efficacy Scale. *Psychologia: An International Journal of Psychology in the Orient*, *38*(3), 174–181.
